# Epileptogenic lesions in the Australian epilepsy project: A harmonized 3‐T magnetic resonance imaging protocol and its diagnostic yield

**DOI:** 10.1002/epi.70381

**Published:** 2026-07-07

**Authors:** David N. Vaughan, David F. Abbott, Amanda Anderson, Greg Fitt, Suyi Ooi, Heath Pardoe, Donna Parker, Yuliya Perchyonok, Eric Pierre, Chris Kokkinos, Robert E. Smith, Michael Stewart, Bahman Tahayori, Chris Tailby, Graeme D. Jackson, the Australian Epilepsy Project Investigators, Zanfina Ademi, Patrick Kwan, Karen Oliver, Mangor Pedersen

**Affiliations:** ^1^ Florey Institute for Neuroscience and Mental Health Melbourne Victoria Australia; ^2^ Department of Neurology Austin Health Melbourne Victoria Australia; ^3^ University of Melbourne Melbourne Victoria Australia; ^4^ Department of Radiology Austin Health Melbourne Victoria Australia; ^5^ Department of Clinical Psychology Austin Health Melbourne Victoria Australia

**Keywords:** encephalocele, first seizure, focal cortical dysplasia, hippocampal sclerosis, magnetic resonance imaging, newly diagnosed epilepsy

## Abstract

**Objective:**

Detection of epilepsy‐causing structural brain lesions on magnetic resonance imaging (MRI) is critical for diagnosis, prognosis, and treatment planning in people with epilepsy. We aimed to establish an epilepsy‐directed multisite harmonized 3‐T MRI acquisition protocol for the Australian Epilepsy Project (AEP) and describe the clinical structural brain findings in the initial participant cohort.

**Methods:**

The AEP MRI protocol was designed to meet clinical diagnostic and research needs. It includes HARNESS‐MRI sequences plus targeted views (temporal pole), susceptibility and diffusion imaging, quantitative measurements (T1, T2), and selected functional MRI tasks. A total of 1330 adults were enrolled and completed imaging assessment at seven sites. Diagnoses at referral were 146 people with first unprovoked seizure only (FUS), 325 with newly diagnosed epilepsy (NDE), 468 with drug‐resistant focal epilepsy (DRE), and 391 healthy controls. Imaging data were curated via a centralized platform, relevant sequences were clinically reported by subspecialist epilepsy neuroradiologists, and the report findings were classified. Findings were compared to prior reports from standard clinical MRI.

**Results:**

A structural brain lesion considered to be epileptogenic was detected in 12% in FUS, 18% in NDE, and 39% in DRE, compared with 2% in healthy controls. The most common epileptogenic lesions included hippocampal sclerosis, acquired cortical injury, malformations of cortical development, long‐term epilepsy associated tumors, and vascular malformations. Potentially epileptogenic lesions included temporal pole encephaloceles, which were detected in 12% of epilepsy participants versus 7% of healthy controls. In 682 participants with an available prior clinical MRI report, 62 (9.1%) had a new epileptogenic lesion detected on the AEP scan, indicating greater sensitivity of the AEP imaging and reporting process for detection of epileptogenic lesions over standard care (*p* < .001).

**Significance:**

The AEP 3‐T MRI research protocol is feasible at multisite national scale, and sensitive for detection of subtle lesions when read by neuroradiologists with expertise in epilepsy. When added to current Australian clinical practice, this strategy yields significantly more epileptogenic findings.

**Key points**

- The Australian Epilepsy Project is a prospective national‐scale observational study to improve epilepsy diagnostics and support clinical decision‐making.
- We deployed a harmonized 3‐T MRI protocol at seven Australian research imaging centers.
- Potentially epileptogenic lesions were detected in 12% with first unprovoked seizures, 18% with newly diagnosed epilepsy, and 39% with drug‐resistant focal epilepsy.
- In people who had a prior clinical MRI, the project found 9% more potentially epileptogenic lesions, showing higher sensitivity for detection than standard care.

## 1 INTRODUCTION

Magnetic resonance imaging (MRI) is a cornerstone of modern epilepsy diagnosis and management.^1^ It can provide visualization of the anatomical substrates of epilepsy, either developmental or acquired, and is fundamental for determining why seizures occur, establishing prognosis, and guiding treatment decisions^2^ including suitability for surgical intervention.^3^ In many cases, MRI findings can directly influence the classification of epilepsy syndromes and help distinguish between self‐limited forms and those likely to become drug‐resistant.^4^, ^5^

Despite advances in MRI technology, detecting epileptogenic lesions remains challenging, particularly for subtle abnormalities.^6^ As MRI hardware and sequences have evolved, so too has the clinical focus, shifting from overt abnormalities such as large infarcts or tumors to more subtle findings. Hippocampal sclerosis was an important early target for detection on MRI, and its recognition has improved surgical outcomes in temporal lobe epilepsy.^7^ Subsequently, the importance of focal cortical dysplasia has been increasingly appreciated, prompting refinement of acquisition protocols and the adoption of higher resolution imaging to visualize cortical architecture.^8^ More recently, temporal pole encephaloceles have emerged as a potentially relevant finding for epilepsy,^9^ requiring optimized imaging protocols and expert interpretation to determine their clinical significance.^10^

The Australian Epilepsy Project (AEP) is a national‐scale government‐funded initiative aiming to improve the lives of people with epilepsy through access to advanced diagnostics within a research framework.^11^, ^12^ It implements a platform model for prospective data collection and analysis, integrating information from advanced MRI, neuropsychological assessment, genomic sequencing, and clinical phenotyping. The project objectives are to (1) provide population access to advanced neuroimaging and diagnostic tools, (2) create a national resource of high‐quality curated data to support discovery and translational research, and (3) stimulate growth in the medical technology sector by fostering innovation in imaging, data science, and diagnostic software.

The goal of this study is to develop a harmonized 3‐T MRI protocol, applicable for both research and individual clinical use. Its purpose is both to detect epileptogenic lesions and to provide quantitative biomarkers for prediction of epilepsy outcomes. We present the complete protocol to document the full AEP imaging dataset, although analysis here is restricted to the subset of radiologist‐reported structural sequences. With the protocol established, we first ask what the imaging findings in this large‐scale cohort are. We identify which abnormalities are associated with drug‐resistant epilepsy, compared to first seizure or newly diagnosed epilepsy cohorts, to inform the prediction of pharmacoresistance. Then we identify lesions that occur commonly in healthy controls to help avoid overinterpretation of common population variants.

Using the clinical report from expert neuroradiologist review, we classify findings into three broad categories: (1) “epileptogenic” abnormalities highly likely to cause epilepsy, (2) “potentially epileptogenic” findings that can cause epilepsy but may be of uncertain significance in a given individual, and (3) “other findings” on MRI that may be unrelated to the cause of epilepsy in this person. Finally, we compare the radiological yield of the AEP protocol to that of standard‐of‐care MRI, previously performed in the same participants, to investigate the added value of advanced standardized imaging and dedicated neuroradiologist review.

## 2 MATERIALS AND METHODS

### 2.1 MRI protocol design and rationale

The AEP MRI protocol was designed to deliver high‐quality, harmonized 3‐T neuroimaging suitable for large‐scale deployment for a heterogeneous adult seizure cohort. The clinical aim was to maximize detection of epilepsy‐causing structural lesions, including subtle abnormalities, whereas the research aim was to support quantitative imaging and connectomics for biomarker development and use in models for outcome prediction.

Feasibility and participant tolerability are critical, given the prevalence of fatigue, anxiety, and cognitive impairment in people with epilepsy. Based on pilot testing, a single 90‐min session was chosen in preference to multiple split sessions. Individual sequences were limited to <6 min to reduce participant head motion and maintain comfort. Total image acquisition time was 62 min, with remaining session time allocated for setup, task instructions, and repeat acquisitions if required (Table 1).

**TABLE 1:** AEP MRI acquisition protocol.

Sequence	Orientation	Acquisition type	Resolution	Notes	Time	Radiologist reported
T1w MPRAGE^a^	Sagittal	3D	.9 × .9 × .9 mm	—	4:32	Y^b^
T2w FLAIR^a^	Sagittal	3D	.9 × .9 × .9 mm	—	5:32	Y
T2w TSE	Axial	2D	.2 × .2 × 3.0 mm	—	3:38	Y
T2w TSE “hippocampi”^a^	Coronal oblique	2D	.4 × .4 × 2.0 mm	Partial FOV: hippocampi	3:17	Y
T2w SPACE “encephalocele”	Axial oblique	3D	.25 × .25 × .5 mm	Partial FOV: temporal poles	5:38	Y
DWI^c^	Axial	2D	1.8 × 1.8 × 1.8 mm	103 shells *b* = 0, 300, 1000, 3000 mm/s^2^	2 × 2:58	Y (trace, ADC)
Multiecho GRE (SWI/QSM)	Axial	3D	1.2 × 1.2 × 1.2 mm	5 echoes	3:39	Y (SWI)
T1 map MP2RAGE^c^	Sagittal	3D	1.0 × 1.0 × 1.0 mm	‐	4:03	—
T2 map GRAPPATINI^c^	Coronal oblique	2D	.9 × .9 × 4.0 mm	‐	3:17	—
Functional MRI^c^, ^d^: language task	Axial oblique	2D	3.0 × 3.0 × 3.0 mm	3 echoes (13.4, 33, 52.5 ms) TR = 900 ms	3:40	—
Functional MRI^c^, ^d^: working memory task	Axial oblique	2D	3.0 × 3.0 × 3.0 mm	3 echoes TR = 900 ms	5:13	—
Functional MRI^c^, ^d^: movie‐watching task	Axial oblique	2D	3.0 × 3.0 × 3.0 mm	3 echoes TR = 900 ms	14:14	—
				TOTAL	62:39	

Abbreviations: 3D, three‐dimensional; ADC, apparent diffusion coefficient; AEP, Australian Epilepsy Project; DWI, diffusion‐weighted imaging; FLAIR, fluid‐attenuated inversion recovery; FOV, field of view; GRE, gradient‐recalled echo; MP2RAGE, Magnetization‐Prepared 2 RApid Gradient Echoes; MPRAGE, magnetization‐prepared rapid acquisition gradient echo; MRI, magnetic resonance imaging; QSM, quantitative susceptibility mapping; SPACE, sampling perfection with application‐optimized contrast using different flip angle evolution; SWI, susceptibility‐weighted imaging; T1w, T1‐weighted; TR, repetition time; TSE, turbo spin echo; Y, yes.

^a^ Sequences recommended by the HARNESS‐MRI guidelines.

^b^ Sequences marked “Y” (radiologist reported) are applied in AEP for clinical use. Three protocol deployment options are suggested: clinical set (27 min), clinical set plus encephalocele‐SPACE (32 min), or full research protocol (63 min).

^c^ Sequences for T1 and T2 mapping, not analyzed here, are “work in progress” packages used under research agreement with Siemens Healthineers. Functional MRI and diffusion imaging used the University of Minnesota CMRR sequence under the C2P (Collaborative to Proprietary) agreement. All other sequences are clinically available.

^d^ Each functional task is followed by acquisition of a pair of “blip‐up”/“blip‐down” spin‐echo sequences, acquisition time = 17 s, to perform susceptibility distortion correction for the preceding functional MRI data. Single‐band reference images are also acquired to enhance registrations.

For clinical lesion detection, the protocol adopts the International League Against Epilepsy (ILAE) Harmonized Neuroimaging of Epilepsy Structural Sequences (HARNESS‐MRI) recommendations,^13^ including high‐resolution whole‐brain three‐dimensional (3D) T1‐weighted and T2‐weighted fluid‐attenuated inversion recovery imaging, and hippocampal‐directed coronal T2‐weighted imaging. Whole‐brain 3D acquisitions were performed at .9‐mm isotropic resolution to achieve submillimetric resolution and maintain excellent signal‐to‐noise. Additional sequences were included to improve characterization of specific lesion types: axial 2D whole‐brain T2‐weighted imaging, susceptibility‐weighted imaging calculated from multiecho gradient‐echo data, diffusion imaging to generate apparent diffusion coefficient (ADC) and trace images, and a targeted high‐resolution 3D T2‐weighted sampling perfection with application‐optimized contrast using different flip angle evolution (SPACE) acquisition optimized for detection of temporal pole encephaloceles (Figure 1). These sequences were each optimized to balance acquisition time, spatial resolution, and signal‐to‐noise. To support clinical adoption, they can be deployed as a *clinical set* (HARNESS‐MRI plus axial T2‐weighted, susceptibility, and diffusion imaging), the *clinical set plus* (further adding the encephalocele‐directed SPACE sequence), or the *full research protocol*.

To achieve research objectives, the protocol incorporates multishell high‐angular‐resolution diffusion imaging, quantitative T1 and T2 mapping, and multiband multiecho functional MRI. The functional tasks performed are language lateralization, working memory, and a naturalistic movie‐watching paradigm. Full acquisition parameters are provided in Supplementary Information (Data S1).

**FIGURE 1:**
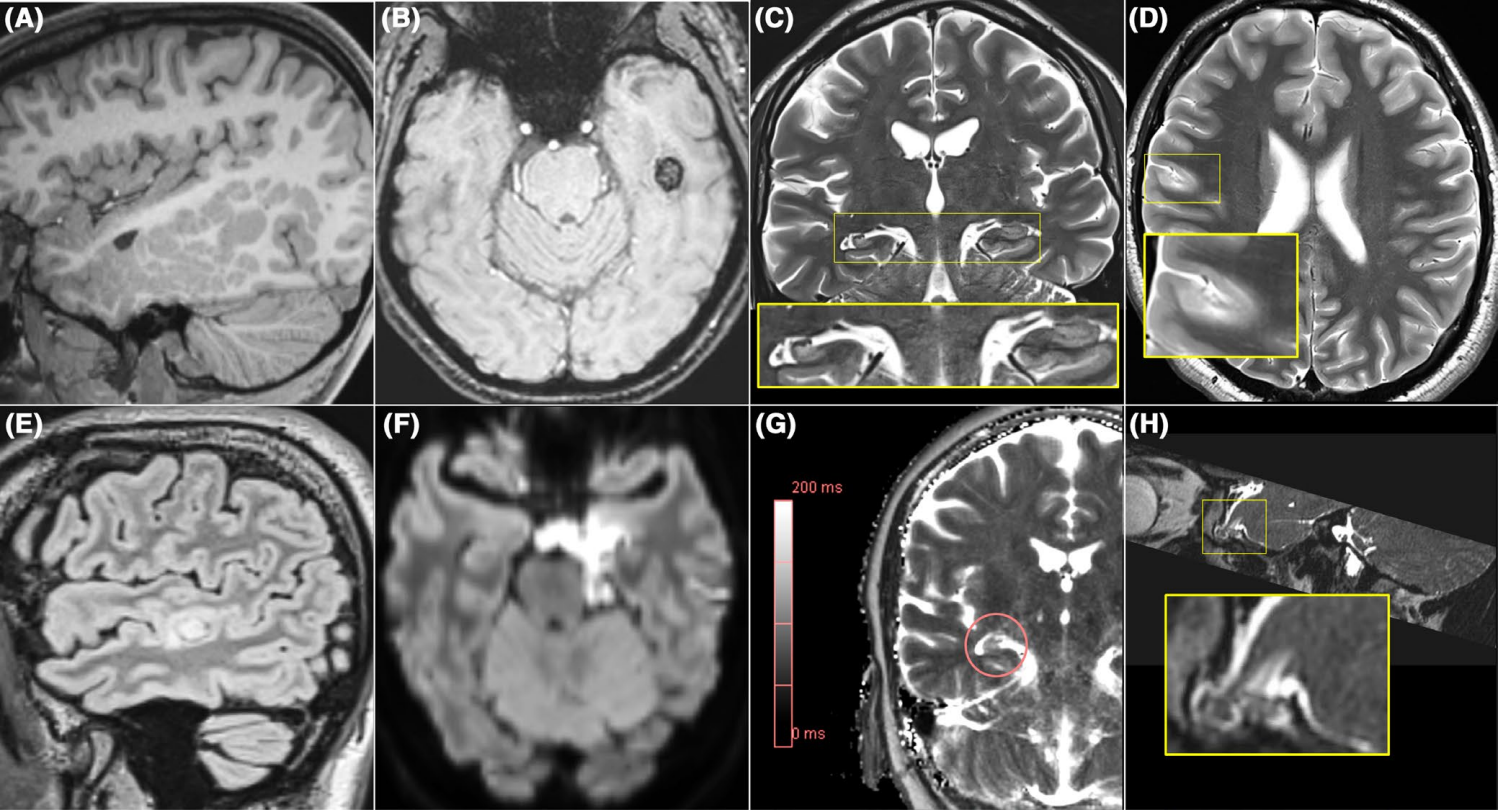
Structural magnetic resonance imaging sequences with examples of epileptogenic pathologies. (A) Three‐dimensional (3D) T1‐weighted (T1w) magnetization‐prepared rapid acquisition gradient echo: temporal lobe polymicrogyria and gray matter heterotopia. (B) 3D multiecho gradient echo–susceptibility‐weighted imaging: temporal cavernous hemangioma. (C) 2D coronal T2w turbo spin echo (TSE): right hippocampal sclerosis. (D) 2D axial T2w TSE: bottom‐of‐sulcus focal cortical dysplasia. (E) 3D T2w/fluid‐attenuated inversion recovery: dysembryoplastic neuroepithelial tumor/ganglioglioma at superior temporal sulcus. (F) 2D diffusion‐weighted imaging–trace: epidermoid cyst encasing brainstem and mesial temporal structures. (G) 2D GRAPPATINI T2 map: increased T2 values in right hippocampal sclerosis. (H) 3D T2w sampling perfection with application‐optimized contrast using different flip angle evolution “encephalocele field of view”: temporal pole encephalocele also showing increased T2 signal.

### 2.2 Participant recruitment

People with unprovoked seizures or epilepsy were referred to the study by their treating neurologist. Inclusion criteria were age 18–67 years, able to consent for themselves and complete surveys in English, available for follow‐up over 24 months, and no contraindication to having MRI. People with developmental and epileptic encephalopathy or a progressive medical or neurological condition were excluded. Recruitment was in three categories, based on diagnosis made by the referring neurologist:

1. First unprovoked seizure (FUS) within the past 6 months but no current diagnosis of epilepsy
2. New diagnosis of epilepsy (NDE) in the past 6 months, whether focal, generalized, or unknown
3. Drug‐resistant focal epilepsy (DRE), defined as having ongoing focal seizures despite adequate trials of at least two appropriate antiseizure medications.^14^

Study eligibility was determined based on the neurologist referral, review of medical records, and participant interview. People with longstanding well‐controlled epilepsy and people with medication‐resistant generalized epilepsy were not included.

Healthy controls were invited to participate, through word‐of‐mouth, social media advertisement, and personal invitation from relatives who were themselves participants with seizures or epilepsy (a “plus‐one” invitation). Healthy controls had no personal history of seizures, no other severe neurological diagnosis or brain disorder (excepting migraine and mood disorders), and met all other inclusion criteria above.

### 2.3 MRI data acquisition

MRI data were acquired prospectively on nine scanners at seven Australian research imaging sites. Scanners included 3‐T MAGNETOM Prisma (*n* = 5), Vida (*n* = 2), Skyra (*n* = 1), and Cima.X (*n* = 1) systems (Siemens Healthineers). Ultimately, all sites used a 64‐channel head–neck coil, although a 32‐channel head coil was used for the first 154 participants on one scanner only. Participants were trained in the functional MRI tasks prior to the session, during a videoconference‐based appointment for cognitive assessment.^15^ Pulse and respiratory belt monitoring were recorded throughout the scan. Participants were fitted with MRI‐compatible earphones (e.g., Optoacoustics) and provided with a pair of two‐button response boxes (Cedrus). Participants were asked to remain still, and their head position was stabilized with foam pads. Participants were encouraged to watch a video of their choice during structural imaging.

Acquisition parameters were prospectively harmonized across scanners. For diffusion imaging, two acquisition variants were established to accommodate the gradient performance of different scanners.

Of 1347 total scheduled participants, 17 (1.3%) attended but could not commence or complete the scan. Reasons included restrictive scanner bore or head coil dimensions (*n* = 8, .6%), claustrophobia or anxiety (*n* = 5, .4%), or excessive movement (*n* = 2, .1%). These participants were withdrawn from the study. A seizure occurred during the MRI session for 19 (1.4%) participants (one tonic–clonic, 10 focal impaired consciousness, five focal preserved consciousness, six mild seizures of unknown type), and scanning was completed at another session if required.

### 2.4 Quality control of MRI data

Acquisition parameters were developed and iteratively refined during the pilot phase at the first scanning site. Uniform parameter setup and scan quality was subsequently confirmed via test scans at each site initiation. For each participant, the radiographer reviewed image quality during the session, optionally repeated series if required, and completed a secure online survey form noting any unusual events and to upload task logfiles. Centralized data curation included an automated check of Digital Imaging and Communication in Medicine (DICOM) header image acquisition parameters against the expected protocol.^16^ Finally, neuroradiologist reporting included a comment on image quality whenever relevant to clinical interpretation.

### 2.5 Centralized MRI data processing and interpretation

Centralized postprocessing included generation of automated multiplanar reformats from volumetric 3D structural images. Trace and ADC images were generated from diffusion‐weighted imaging data. Susceptibility‐weighted imaging (SWI) was generated from the multiecho gradient‐recalled echo data. Then, the clinically relevant sequences were assembled in DICOM format (see Table 1).

Clinical reports were generated by a subspecialist epilepsy neuroradiologist (G.F., Y.P., M.S.), based on visual inspection, without routine use of quantitative measurements. Uncertain cases were discussed to reach a consensus diagnosis.

### 2.6 Classification of epileptogenic brain lesions

Findings on the neuroradiologist MRI report were manually classified according to a prespecified schema (Table S1), informed by the National Institutes of Health Epilepsy Imaging Common Data Elements,^17^ previous epilepsy MRI cohort studies,^18^ and other key descriptions of epileptogenic pathologies.^19^, ^20^, ^21^, ^22^ Findings were grouped as “epileptogenic” where there is a known strong association between the lesion type and epilepsy (e.g., focal cortical dysplasia). Lesion types where the association with epilepsy may be uncertain (e.g., temporal pole encephaloceles) and individual findings that could not be definitively resolved on imaging were classed as “potentially epileptogenic.” Post‐neurosurgery changes were defined as “potentially epileptogenic” if no additional lesion was visible. The remaining noted MRI findings, which are not usually considered to be causative of epilepsy, were grouped as “other findings.” Importantly, these groupings reflect the association between a *lesion type* and epilepsy at the population level, and do not assert that the lesion is a proven cause of seizures for an individual, as we did not have electroclinical or surgical confirmation for most cases. Conversely, for pathologies strongly associated with epilepsy, the clinical onus is often to demonstrate that the lesion is incidental, rather than to prove it is causative.

We obtained the radiologist report of any prior clinical brain MRI, performed before the AEP scan. These scans were performed in diverse settings, including private radiology providers, regional hospitals, and tertiary epilepsy centers. Prior clinical MRI findings were classified according to the same schema.

## 3 RESULTS

### 3.1 Participant demographics and clinical characteristics

In total, 1330 participants participated in the study and completed MRI between January 1, 2020 and July 1, 2025. Demographic and clinical details are shown in Table 2. Participants with seizures or epilepsy were on average younger than the controls (33 ± 13 SD vs. 39 ± 14 years, *t*‐test *p* < .001) and included fewer females (53% vs. 67%, chi‐squared *p* < .001). Self‐reported ethnicity included people with European (80.1%), Asian (11.3%), and Oceanian ancestry (5.2% including Australian Aboriginal and Torres Strait Islander people). The distributions of ethnicity were not significantly different between groups (bootstrap test of multiple marginal independence *p* = .088), although Asian ancestry was more common in the control group (Bonferroni‐corrected chi‐squared for multiple categories *p* = .027).

- FUS participants (*n* = 146) had only one seizure ever. For most it was a tonic–clonic seizure (91%), occurring a median of 2.4 months previously.
- NDE participants (*n* = 325) included focal (62%), generalized (15%), and unknown onset epilepsy (23%). Seizures had occurred for a median of 1.2 years before the study, with median frequency of 4 seizures per year. Some NDE participants (61/325) had a single seizure only, and epilepsy was diagnosed based on clinical investigation findings.
- DRE participants (*n* = 468) mostly experienced focal to bilateral tonic–clonic seizures (76%) and/or focal impaired consciousness seizures (77%). Median duration of epilepsy was 13.9 years, with a median of 53 seizures in the past year, despite treatment with a mean of 2.6 concurrent antiseizure medications. Prior neurosurgery had been performed in 58 (12.4%) of this group.

**TABLE 2:** Participant demographics and epilepsy characteristics.

Characteristic	FUS	NDE	DRE	Controls	*p*
*n*	146	325	468	391	—
Age, years, mean (range)	37.6 (18–67)	35.7 (18–68)	36.0 (18–67)	39.2 (18–68)	**<.001** ^a^
Sex, female, %	49%	53%	53%	67%	**<.001** ^b^
Years of education, mean (SD)	13.5 (2.6)	13.2 (2.5)	12.9 (2.3)	14.8 (2.3)	**<.001** ^a^
Ethnicity					
European (northwest Europe)	68%	69%	68%	68%	1.0^c^
European (southern and eastern Europe)	9%	11%	12%	12%	1.0
Asian	9%	11%	10%	19%	.**03**
Oceania (including indigenous Australian)	3%	6%	8%	5%	.31
Diagnosis at recruitment					
FUS only—no epilepsy diagnosis	100%	—	—		
Focal epilepsy	—	62%	100%		
Generalized epilepsy	—	15%	—		
Epilepsy with unknown onset	—	23%	—		
Seizure types					
Focal preserved consciousness	3%	25%	49%		
Focal impaired consciousness	7%	33%	77%		
Absence	—	3%	—		
Tonic–clonic	91%	73%	76%		
Years of seizures, median (range)	.2 (0–.7)	1.2 (.1–44)	13.9 (.7–60)		
Seizure count in past 12 months, median (Q1–Q3)	1 (1–1)	4 (1–21)	53 (14–203)		
Number of antiseizure medications, mean (range)	.1 (0–3)	1.0 (0–3)	2.6 (0–6)		

*Note*: Boldface indicates statistical significance.

Abbreviations: DRE, drug‐resistant focal epilepsy; FUS, first unprovoked seizure; NDE, new diagnosis of epilepsy.

^a^ Analysis of variance.

^b^ Chi‐squared test.

^c^ Chi‐squared (Bonferroni adjusted for multiple marginal independence).

### 3.2 MRI quality

Image artifacts due to head movement were seen more often in people with seizures than in controls (*χ* ^2^ = 50.1, *p* < .001). One or more sequences were rated by the neuroradiologist as “motion degraded” in 18.7% of patients versus 6.4% of healthy controls. Milder “motion affected” series were noted in an additional 15.2% of patients and 8.7% of healthy controls. Sequences impacted most often were the T2‐weighted turbo spin echo and T2 mapping.

Additional clinical imaging was recommended by the radiologist in 10.8% of patient scans versus 3.8% of healthy controls (*χ* ^2^ = 16.7, *p* < .001). Recommendations included postcontrast imaging, interval scanning to assess longitudinal change, or targeted pituitary/pineal imaging.

### 3.3 Incidence of epileptogenic structural brain lesions

Rate of epileptogenic lesions was highest in the DRE group at 39.3%, compared to 18.2% in NDE, 11.2% in FUS, and 2.0% in healthy controls (*p* < .001; Figure 2). Most had a single lesion (72.5% of 284 participants with epileptogenic lesions), with others having two or more epileptogenic findings. In those diagnosed with focal epilepsy, 23.0% of NDE‐focal (44/191) and 39.3% of DRE (all focal) had an epileptogenic lesion.

**FIGURE 2:**
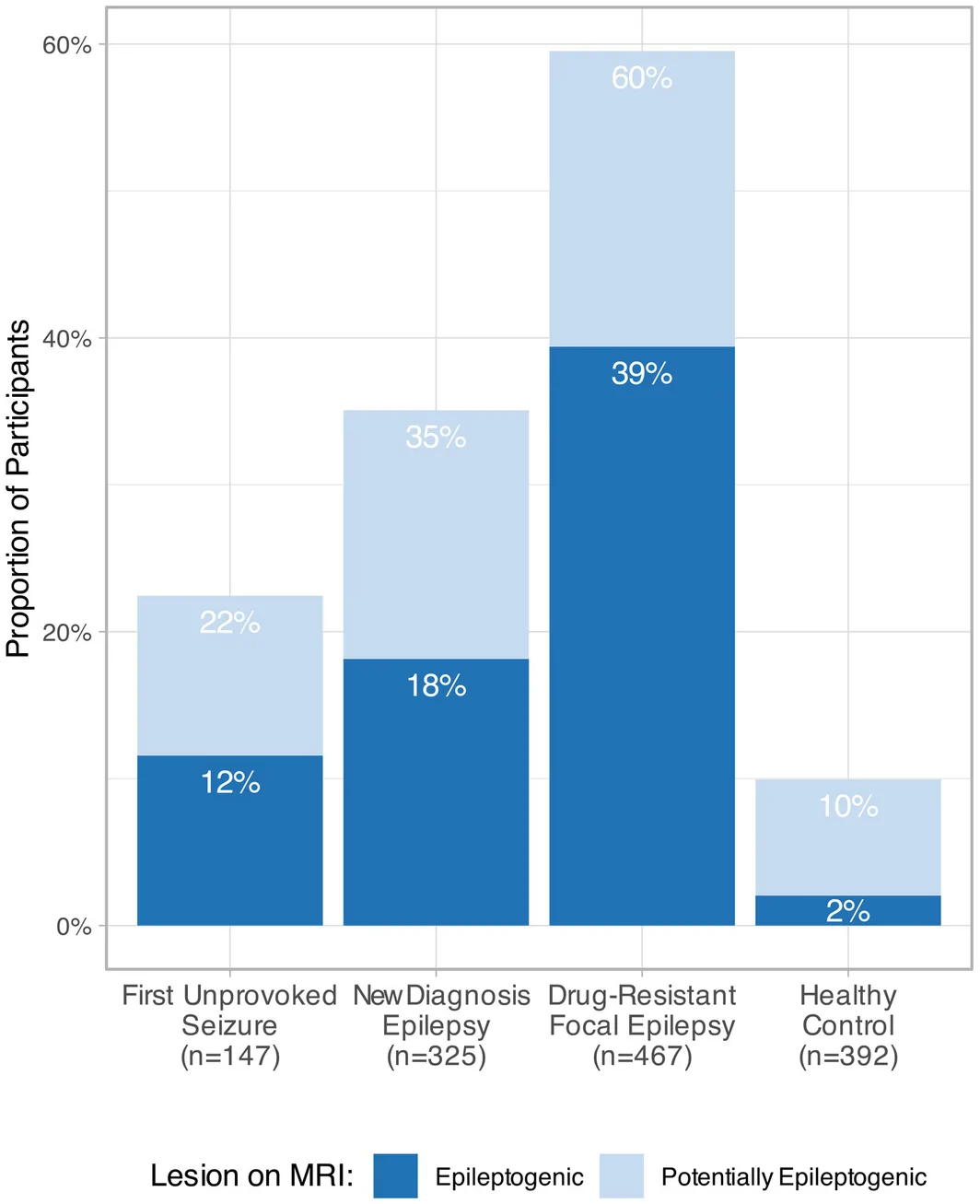
Participants with an epileptogenic lesion on magnetic resonance imaging (MRI). Dark bars show the proportion in each group with an epileptogenic lesion. Light bars show the proportion with either an epileptogenic or a potentially epileptogenic lesion. See Figure 3 for lesion types in each category.

Considering epileptogenic and potentially epileptogenic lesions together, the proportion of affected participants was 59.4% of DRE, 35.1% of NDE, 22.6% of FUS, and 9.9% of healthy controls (*p* < .001). In the DRE group, there was no association between duration of epilepsy and lesional status.

The nature of epileptogenic MRI findings is shown in Figure 3 and Table S2. In DRE, hippocampal sclerosis (13.5%), cortical injury with focal atrophy, encephalomalacia and/or gliosis (>9%), focal cortical dysplasia (7.9%), low‐grade epilepsy‐associated tumors (4.9%), polymicrogyria (3.0%), and gliomas (1.9%) were most frequent. These pathologies were seen more often in DRE than NDE or FUS, highlighting the association of these lesion types with pharmacoresistance. Gray matter heterotopia (4.7% in DRE vs. 3.7% in NDE and 1.0% in healthy controls) and cavernoma (3.4% in DRE vs. 3.4% in NDE and 1.3% in healthy controls) were also important causes of epilepsy but were not associated with pharmacoresistance. Chronic cortical infarct or hemorrhage (1.7% in DRE, 1.5% in NDE, and .3% in healthy controls) was an uncommon cause of epilepsy in our cohort, reflecting study referral patterns and the enrollment criterion of age less than 68 years.

**FIGURE 3:**
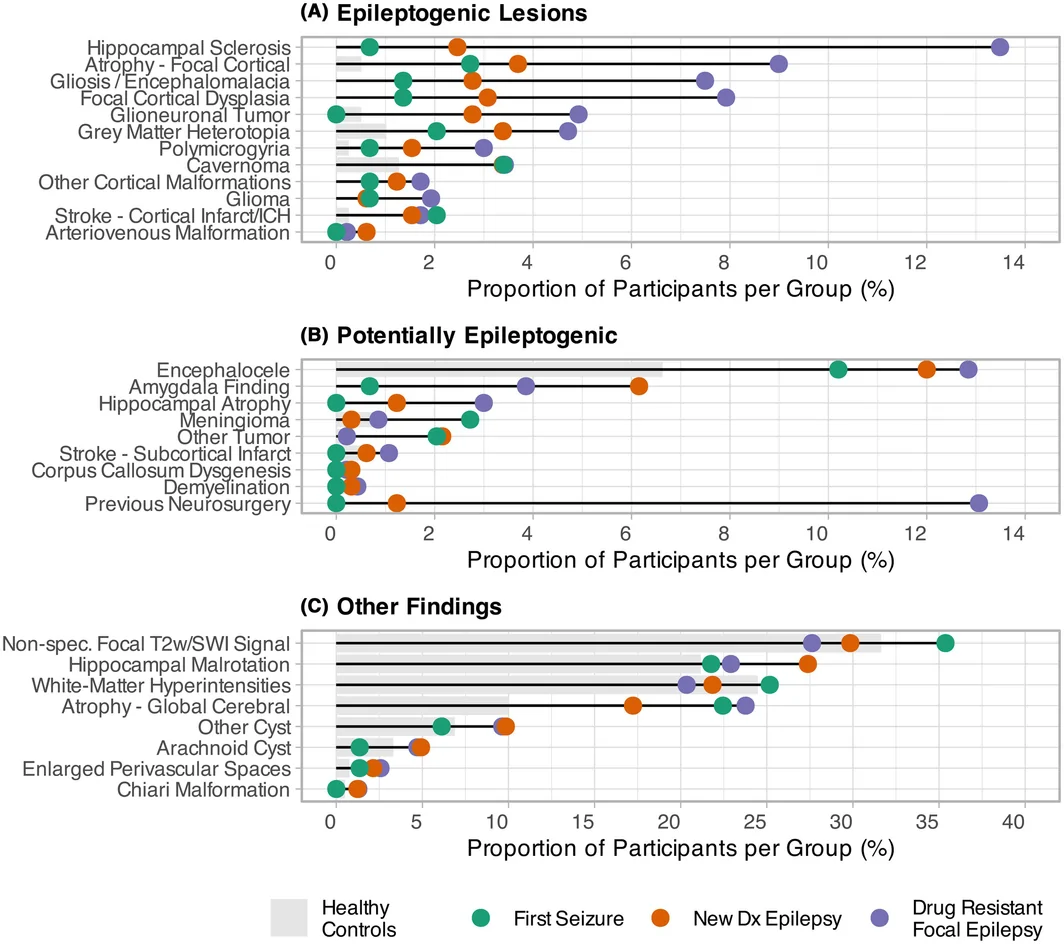
Structural magnetic resonance imaging (MRI) findings in the Australian Epilepsy Project. MRI findings are listed on the left, with the proportion occurring in each recruitment group shown by colored circles. Proportion of findings in healthy controls is shown as a gray bar. (A) The most common epileptogenic lesions were hippocampal sclerosis, acquired cortical injury, malformations of cortical development, and low‐grade tumors. (B) Potentially epileptogenic lesions included temporal pole encephaloceles and enlargement/signal change of the amygdala. Previous neurosurgery for epilepsy was common in the drug‐resistant focal epilepsy cohort. (C) Other findings such as nonspecific focal signal changes of uncertain etiology, hippocampal malrotation, and white matter T2‐weighted (T2w)/fluid‐attenuated inversion recovery hyperintensities were seen commonly in both people with seizures and healthy controls. Dx, diagnosis; ICH, intracerebral hemorrhage; SWI, susceptibility‐weighted imaging.

Temporal pole encephaloceles were detected in 12.1% of people with seizures versus 6.6% of healthy controls. Other potentially epileptogenic findings (Figure 3B and Table S3) included amygdala enlargement or signal change, hippocampal atrophy, and noncerebral tumors (e.g., meningioma, pineal or pituitary tumors). Additional individual‐level electroclinical correlation would be required to determine whether these MRI findings were the cause of epilepsy.

Other MRI findings (Figure 3C, Table S4) mostly occurred at similar rates to healthy controls. An exception was global cerebral atrophy, which was more common in all seizure groups (FUS 22.4%, NDE 17.2%, DRE 23.8%) versus healthy controls (9.9%, *χ* ^2^ = 24.2, *p* < .001).

### 3.4 Detection of epileptogenic lesions compared to prior standard clinical care

A prior clinical MRI radiologist report was obtained for 682 participants with seizures (399 DRE, 215 NDE, 68 FUS). Median time between the prior clinical MRI and the AEP MRI was 10.8 months (interquartile range = 3.4–37.6 months). This comparison evaluates the additional yield of performing the AEP sequentially and in addition to the existing standard clinical workup. There was high concordance (73.8% of cases) between prior clinical and AEP MRI for the finding of an epileptogenic or potentially epileptogenic lesion (Figure 4A).

**FIGURE 4:**
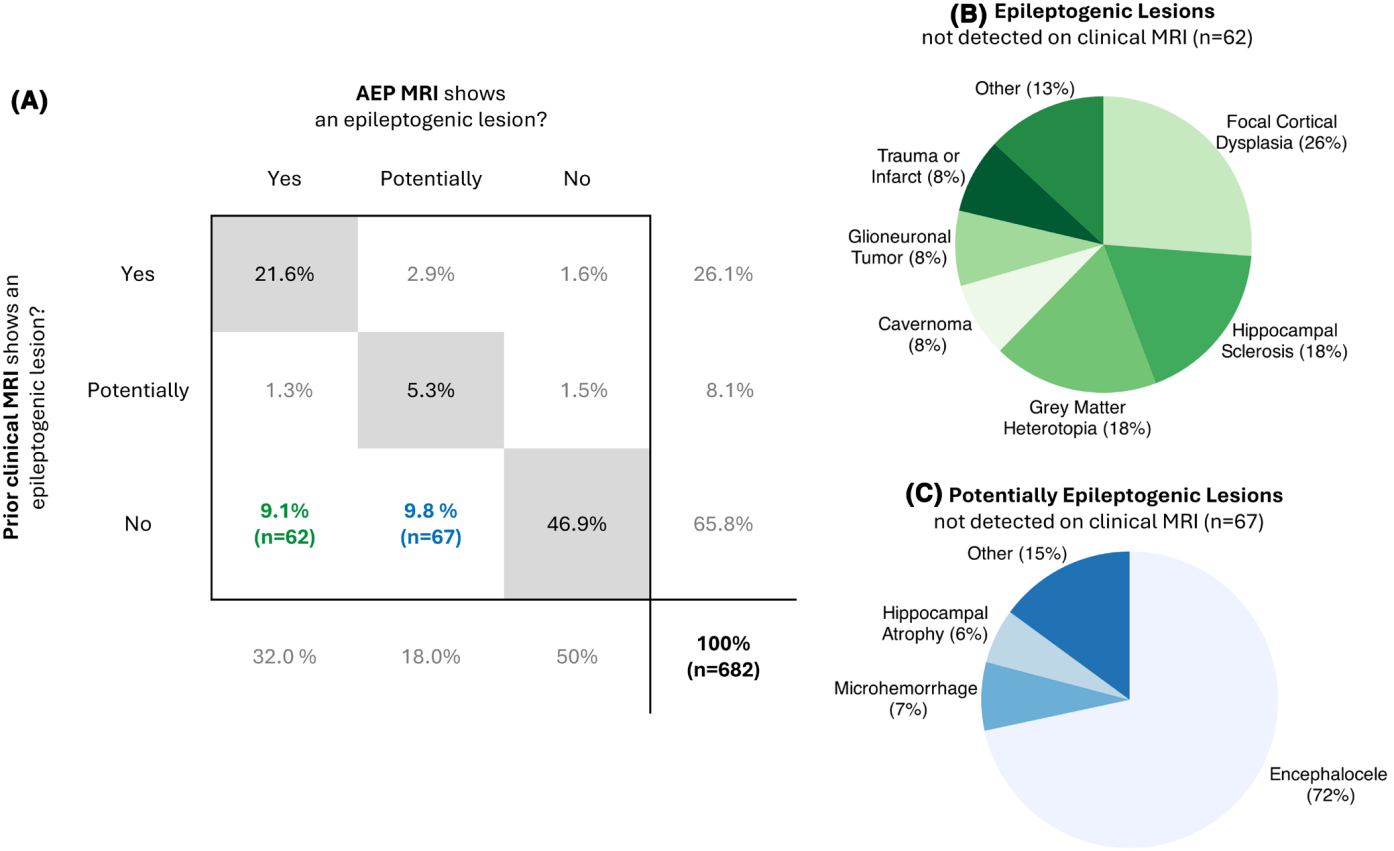
Comparison between Australian Epilepsy Project (AEP) magnetic resonance imaging (MRI) findings and prior clinical MRI findings. (A) Two‐way contingency showing proportion of people with epileptogenic findings on prior clinical MRI compared to the AEP MRI report. Of 682 people, 9.1% had a new epileptogenic finding (“No” ➔ “Yes,” highlighted in green), and 9.8% had a new potentially epileptogenic finding (“No” ➔ “Potentially,” highlighted in blue). (B, C) Lesion types in people with a new epileptogenic/potentially epileptogenic finding on the AEP MRI. Gray matter heterotopia also includes people with a new diagnosis of polymicrogyria.

The AEP protocol and reporting process detected a new epileptogenic lesion in 9.1% of participants, where their previous clinical MRI was negative. Only 1.6% participants had the converse pattern of an epileptogenic lesion seen on prior scan but not in AEP. This demonstrates significantly greater sensitivity of the AEP MRI approach for lesion detection (*p* < .001, McNemar–Bowker test).

Newly detected lesions (Figure 4B) included focal cortical dysplasia, hippocampal sclerosis, gray matter heterotopia/polymicrogyria, small tumors, and vascular lesions. The interval between the clinical and AEP MRI was not associated with the rate of new lesion detection after accounting for referral group (penalized logistic regression odds ratio [OR] = 1.03, *p* = .22). Sensitivity analysis in a restricted subgroup, excluding participants with any conceivably adult‐acquired lesion, still showed benefit of the AEP approach for detecting epileptogenic lesions (*n* = 295, 6.1% newly detected epileptogenic lesions, McNemar–Bowker *p* = .04).

The AEP MRI detected a new “potentially epileptogenic” finding in an additional 9.8% of participants, where their previous clinical MRI was negative, with the majority of these being temporal pole encephaloceles (Figure 4C).

## 4 DISCUSSION

In this study, we developed a standardized 3‐T MRI protocol for the AEP and evaluated its feasibility and diagnostic yield for epileptogenic brain lesions under expert neuroradiologist reporting. Among 939 participants with seizures or epilepsy, we identified epileptogenic lesions in 39% of people with DRE, 18% with NDE, and 12% with FUS, compared to only 2% of healthy controls. In the subset of participants with a prior clinical MRI report, a previously undetected epileptogenic lesion was identified in 9.1% of cases. This clinically and statistically significant increase demonstrates the value of a uniform, epilepsy‐directed protocol combined with centralized postprocessing, quality control, and expert interpretation. In addition to confirming the spectrum of lesion types known to cause epilepsy, this cohort also prospectively determines rates of epilepsy protocol findings in healthy controls, identifies which lesion types are enriched in pharmacoresistant disease, and highlights global cerebral atrophy as a potential biomarker in early epilepsy.

Our protocol design builds upon existing best‐practice guidelines for epilepsy imaging acquisition.^5^ The core sequences from the ILAE HARNESS‐MRI guidelines^13^ provide robust visualization of structural abnormalities across a broad spectrum of pathologies. We found adding an *axial* whole‐brain 2D T2‐weighted sequence was helpful for further characterizing lesions, especially when outside the limited field of view of the coronal T2‐weighted sequence. Two additional sequences boosted our ability to detect subtle lesions. The inclusion of SWI facilitated the sensitive diagnosis of small cavernomas and microhemorrhages that may be otherwise overlooked. Second, we developed a dedicated sequence to visualize temporal pole encephaloceles, using a 3D high‐resolution T2‐weighted SPACE.

In total, the sequences required for clinical radiologist reporting in this study take 32.5 min of MRI acquisition time. Although longer than some existing epilepsy protocols, this clinical component is optimized for subtle lesion detection and is suitable for first‐line epilepsy evaluation. The additional research sequences, T1/T2 mapping and functional MRI, require a further 30 min of acquisition using more specialized equipment and skills. The applicability of these sequences to populations not represented in our cohort, particularly those with more severe cognitive impairment or comorbidities affecting scanner tolerance, remains to be established.

Rates of epilepsy‐causing lesions in this study are broadly comparable to previous retrospective clinical studies, allowing for study‐specific differences in lesional definitions and cohort inclusion criteria. In the first seizure clinic setting, previous Australian studies found epileptogenic lesions in 14% of cases^23^ using 1.5‐T MRI, and more recently in 23% of cases.^18^ A Western Australian first‐seizure study^24^ found a higher rate of epileptogenic lesions at 29%, with the difference likely explained by an older cohort (up to age 91 years) and one third of lesions in that study being stroke. The international Human Epilepsy Project (HEP) study of newly diagnosed focal epilepsy,^25^ including centers from the United States, Europe and Australia, had similar findings to the AEP despite the geographic differences. HEP detected MRI findings likely causative of epilepsy in 19% of newly diagnosed participants, with an additional 17% of participants having a lesion with an unknown relationship to epilepsy. This compares to 23% with newly diagnosed focal epilepsy in the AEP, when we consider only the subset with confirmed focal seizure onset. However, HEP diagnosed barely any encephaloceles, and did not include quantitative T1/T2 imaging, so further analysis of these AEP sequences will be needed to evaluate their long‐term clinical relevance.

In patients with DRE, a higher rate of MRI lesions is expected than at diagnosis, due to the association between structural brain abnormalities and pharmacoresistance.^26^ This has also been demonstrated in a Toronto cohort,^27^ where people with DRE admitted for video‐electroencephalographic monitoring had high rates of both hippocampal sclerosis (17%) and encephalomalacia/gliosis (10%), comparable to our study. We also found focal cortical dysplasia and glioneuronal tumors more often than in newly diagnosed epilepsy, indicating increased risk of pharmacoresistance with these pathologies.

The detection of epileptogenic lesions in our FUS cohort may seem surprising, considering that a single seizure plus a definite epileptogenic lesion on MRI is sufficient for a diagnosis of epilepsy under ILAE criteria.^14^ The explanation is that some patients had no other clinical brain imaging before recruitment, whereas other individuals may have had clinical imaging where a subtle lesions was not yet detected. These scenarios demonstrate the importance of early accurate imaging in the assessment of an unselected first seizure group.

Global cerebral atrophy stands out among the “other MRI findings” as having a significantly higher incidence in people with seizures and epilepsy (21%) than in healthy controls (10%). Rates were similar between people with a first seizure and those with long‐standing drug‐resistant epilepsy. Increasing age was also significantly associated with global atrophy, with increasing risk (OR = 1.17) per decade of age in common across all groups. This indicates that global atrophy is more likely to reflect an underlying process associated with increased risk of seizure onset, rather than being solely a consequence of repeated seizures or antiseizure medications. Quantitative analyses of brain volume in first seizure cohorts are needed to understand this result. In contrast, white matter hyperintensities and enlarged perivascular spaces are just as common in patients as controls.

The diffusion, functional, and quantitative imaging in this protocol have extensive future research applications. Using dedicated research MRI facilities allowed us to employ research‐only sequences for compressed‐sensing T1‐mapping^28^ and accelerated T2‐mapping,^29^ and to acquire diffusion and functional MRI using multiecho multiband echo‐planar imaging via a collaborative‐to‐proprietary license (www.cmrr.umn.edu/multiband).^30^, ^31^ Although this approach has several advantages for research, the requirement for specialized agreements, equipment, and staff training could present a barrier to clinical adoption of these components.

Automated quantitative analysis may further improve detection of subtle epilepsy‐causing lesions, beyond the rates we have reported here. A recent meta‐analysis found a 39% gain in diagnostic yield after computational postprocessing.^32^ Approaches such as hippocampal quantification,^33^, ^34^ automated lesion detection as exemplified by the MELD project,^35^ and normative modeling of structural and functional MRI data^36^, ^37^, ^38^ each hold promise to improve individualized diagnosis.

Several aspects of the study need to be considered. First, lesion identification was based on expert visual review by one of four subspecialist neuroradiologists. Without histopathological, electroclinical, or surgical outcome confirmation in most cases, we could not prove that specific lesions were causative of epilepsy at the individual level. For reporting, clinical details about suspected seizure localization and previous scan findings were provided to the neuroradiologist when available, per usual clinical practice, which may assist with detecting relevant lesions. However, these data are not available for people without seizures, raising the possibility of differential reporting sensitivity in healthy controls. Next, although the MRI protocol was generally well tolerated by the 1330 participants who completed it, the cohort consisted of adult participants who were able to consent and complete cognitive assessments independently. People with significant neurodevelopmental disorders or other conditions that substantially impair cognition or tolerance of the scanner environment may require additional person‐centered adjustments,^39^ and our study has not established feasibility of the protocol in these groups.

Finally, comparison to prior clinical MRIs relied on available radiology reports without blinded rereview of source images, and consequently, interrater agreement between prior clinical and AEP reads could not be calculated. This design does not separate the added value of the acquisition protocol from the benefit of centralized subspecialist reporting. The added yield should therefore be read as their joint benefit, sequentially after standard‐of‐care imaging, rather than as evidence for any single component. Time between clinical and AEP scans introduces the possibility that lesions may have developed during this interval, although sensitivity analyses indicate this did not drive our overall result.

The AEP demonstrates that standardized, high‐resolution 3‐T MRI with harmonized acquisition and expert interpretation is feasible at national scale and significantly improves detection of structural brain abnormalities relevant to epilepsy. Compared to existing clinical services, this approach yields a higher rate of potentially epileptogenic findings, with detection of subtle and surgically relevant pathologies such as focal cortical dysplasia, hippocampal sclerosis, and temporal encephaloceles. Applying a harmonized advanced protocol at the early stages of epilepsy, including after a first unprovoked seizure, provides information that can reduce delays to specific effective treatment. Together, these results support the value of advanced imaging protocols in improving diagnostic accuracy, guiding treatment decisions, and informing prognosis for adults with epilepsy.

## AUTHOR CONTRIBUTIONS

**David Vaughan:** Conceptualization; methodology; formal analysis; project administration; software; visualization; writing—original draft; writing—review and editing. **David F. Abbott:** Conceptualization; methodology; data curation; project administration; software; writing—review and editing. **Amanda Anderson:** Data curation; writing—review and editing. **Greg Fitt:** Data curation; writing—review and editing. **Suyi Ooi:** Methodology; writing—review and editing. **Heath Pardoe:** Conceptualization; methodology; project administration; writing—review and editing. **Donna Parker:** Investigation; data curation. **Yuliya Perchyonok:** Data curation; writing—review and editing. **Eric Pierre:** Methodology; software; writing—review and editing. **Chris Kokkinos:** Data curation; methodology; writing—review and editing. **Robert E. Smith:** Methodology; software; writing—review and editing. **Michael Stewart:** Data curation; writing—review and editing. **Bahman Tahayori:** Methodology; software; writing—review and editing. **Chris Tailby:** Conceptualization; methodology; project administration; software; writing—review and editing. **Graeme D. Jackson:** Conceptualization; funding acquisition; methodology; project administration; supervision; writing—review and editing.

## FUNDING INFORMATION

The Australian Epilepsy Project received funding from the Australian Government under the Medical Research Future Fund, (Frontier Health and Medical Research initiative, grant numbers MRFF75908 and RFRHPSI000008) and the Victoria State Government (Victorian‐led Frontier Health and Medical Research Program).

## CONFLICT OF INTEREST STATEMENT

D.N.V. has received speaker honoraria to his institution from Siemens Healthineers. The remaining authors have no conflicts of interest. We confirm that we have read the Journal's position on issues involved in ethical publication and affirm that this report is consistent with those guidelines.

## ETHICAL APPROVAL

The Australian Epilepsy Project was approved by the Human Research Ethics Committee of Austin Hospital (HREC/68372/Austin‐2022) covering both patient and healthy control recruitment. All participants provided written informed consent prior to study enrollment.

## CLINICAL TRIAL REGISTRATION

The Australian Epilepsy Project is registered through the Australian New Zealand Clinical Trials Registry (ACTRN12623000008628).

## Supporting information


Data S1.
Table S1.
Table S2.
Table S3.
Table S4.

## Data Availability

The data that support the findings of this study are available on request from the Australian Epilepsy Project Data Access Committee (www.epilepsyproject.org.au/research/access‐to‐aep‐data).
