# Supplementary material for: Epileptogenic lesions in the Australian epilepsy project: A harmonized 3‐T magnetic resonance imaging protocol and its diagnostic yield
Source: Epilepsia. 2026 Jul 7;67(10):5398–411. doi: 10.1002/epi.70381 (PMC13675757; doi:10.1002/epi.70381)
Supplement: Supplementary file 1 [file EPI-67-5398-s001.pdf]

Epileptogenic Lesions in the Australian Epilepsy Project: A Harmonised 3T MRI Protocol and its Diagnostic Yield

Supplementary Information

Table of Contents

Supplementary Methods **1**
MRI Protocol Design – Clinical Sequences *1*
MRI Protocol Design - Research Sequences *2*
Centralized Post-processing of Image Data for Clinical Reporting *4*
Schema for Classification of MRI Findings from Radiologist Reports *5*

Supplementary Results **7**
Incidence of Epileptogenic Lesions and Other MRI Findings *7*

Supplementary References **9**

Australian Epilepsy Protocol MRI Sequence Parameters **11**
AEP MRI Protocol for Siemens 3T Prisma (XA60) *11*
AEP MRI Procotol for Siemens 3T Vida (XA60) *12*
AEP MRI Protocol for Siemens 3T Cima.X (XA61) *13*

Supplementary Methods

MRI Protocol Design – Clinical Sequences

The protocol adopts the ILAE HARNESS-MRI sequence recommendations[1] and includes whole-brain 3D T1-weighted MPRAGE, 3D T2-weighted FLAIR, and hippocampal-directed 2D T2-weighted sequences. Sequence parameters were based on existing protocols at our institution, parameters used at the Montreal Neurological Institute[1], and in population studies such as Human Connectome Project[2], UK Biobank[3], and ABCD[4]. We then tailored parameters to best suit the available hardware in our network. To optimize individual-participant image signal-to-noise against maximal spatial resolution, for whole-brain 3D structural sequences we chose an isotropic resolution of 0.9mm^3.

The 2D coronal-oblique T2w TSE 'Hippocampal' sequence is orientated perpendicular to the long axis of the hippocampus. It has a limited field-of-view that includes the entire hippocampus, extending from immediately posterior to the hippocampal fornixes, into the anterior temporal regions.

The T2w SPACE 'Encephalocele' sequence has a targeted field-of-view for examination of the antero-basal surface of the temporal lobe in the middle cranial fossa. This is achieved with an oblique axial slice orientation, at approximately +25 degrees to the AC-PC axis.

Administration of gadolinium contrast and individually tailored sequences were not performed in this study. When these were required, advice was provided to the treating neurologist to request an additional scan through their local clinical radiology service. Similarly, sedation or general anaesthesia was not available, and participants requiring these for MRI scanning were excluded.

MRI Protocol Design - Research Sequences

These sequences are aimed at obtaining individual-level quantitative imaging biomarkers useful for diagnosis, prediction of epilepsy outcomes, and clinical decision support. We focused on quantitative relaxometry, diffusion imaging and functional imaging.

Quantitative T1 and T2 mapping provides measures of brain tissue microstructure relevant to lesion detection, neurodevelopment and degeneration. We adapted the Lausanne Parametric Mapping protocol[5]. T1 mapping used a Siemens work-in-progress (WIP) research sequence for compressed sensing 3D MP2RAGE[6]. T2 mapping used the GRAPPATINI Siemens WIP research sequence for accelerated 2D multi-echo relaxometry assessment[7]. For T2 mapping we set the slice angle perpendicular to the hippocampal axis, to optimize estimation of hippocampal tissue[8]. These sequences were not available on Siemens Cima.X scanners during the initial study period.

Data for Quantitative Susceptibility Mapping were acquired using a 3D multi-echo gradient echo (MEGRE) approach. This sequence was also used to derive a multi-echo SWI image for clinical reporting, described below. Axial images were acquired perpendicular to B0, with voxel size 1.2mm isotropic. Five echoes were captured at TE1/ΔTE/TR=5.84/4.79/30ms. Acceleration included in-plane GRAPPA 3 and central elliptical filling of k-space only (‘elliptical scanning’). Magnitude and phase images were reconstructed online using the adaptive coil combine algorithm.

Diffusion weighted imaging employed a multi-shell, high-angular-resolution approach to enable quantitative analyses such as normative fixel-based analysis[9], tractography and structural connectomics. A total of 105 volumes are obtained, split evenly across two sequential acquisitions with opposing phase-encode directions[10], using the CMRR multiband EPI sequence (www.cmrr.umn.edu/multiband, MB factor = 4)[11,12]. Diffusion weightings were b=0 mm/s^2 (8 volumes), 300 mm/s^2 (13 volumes), 1000 mm/s^2 (24 volumes) and 3000 mm/s^2 (60 volumes)[13,14]. We defined two DWI protocols to allow for consistent diffusion weightings and acquisition times despite differing gradient performance capabilities: on Prisma and Cima.X scanners we obtained spatial resolution 1.8mm isotropic, with a shorter echo time (TE 83ms); on Skyra and Vida scanners we obtained spatial resolution 2.0mm isotropic using a longer echo time (TE 113ms). Multi-coil reconstruction used the SENSE1 algorithm[15]. Phase images were saved to facilitate complex data denoising[16].

Functional MRI was implemented for assessment of (i) language laterality using a pseudo-word rhyming task, (ii) working memory function using a spatial one-back task, and (iii) whole-brain

functional connectomics using a naturalistic movie-watching task. Training to perform these tasks is conducted before the day of the MRI scan, and participants are reminded of the instructions on the day.

fMRI Acquisition: Functional MRI acquisition used a Multi-Band Multi-Echo (MBME) echo-planar sequence (www.cmrr.umn.edu/multiband)[11,12], to optimize contrast-to-noise and facilitate advanced multi-echo denoising strategies[17]. Uniform acquisition parameters (e.g. TR, receiver bandwidth) were chosen to be compatible with intrinsic hardware frequency-band requirements across all scanner models. Key parameters were TR 900ms, three echoes at TE 13.4 / 32.96 / 52.52ms, multi-band factor 4, in-plane acceleration GRAPPA 2 and partial Fourier 7/8. Voxel size was 3mm isotropic, acquired as 44 interleaved slices with an anterior-to-posterior phase encode direction, with an axial oblique plane at +30 degrees to the AC-PC axis. LeakBlock (split-slice GRAPPA) kernel optimization was applied[11], and multi-coil reconstruction used the SENSE1 algorithm[15].

Single-band reference images ('SBref') were acquired with each fMRI run for use in subsequent registration and image-mask calculations.

For estimation and geometric correction of susceptibility distortion, we used a reversed phase encoding polarity ('blip-up/blip-down') approach[18]. A pair of spin-echo EPI volumes with opposite phase encoding (AP/PA) were acquired immediately after each fMRI run, for estimation of field inhomogeneity maps. These images had matched field-of-view and slice angle to the fMRI echo-planar acquisition, although with higher spatial resolution (2 mm isotropic voxels, matrix 108x120, 72 axial slices).

All fMRI task stimulus presentation and button-response recording was performed using Presentation software (NeuroBehavioural Systems, Albany, California).

Pseudoword rhyming task paradigm: This is a block-design task, with alternating 18 s blocks of active task versus baseline task. In the active task, participants are shown pairs of pseudowords (e.g. "blint" versus "glat") and must decide whether they rhyme. Participants perform the task without speaking aloud. They respond whether the words rhyme or not via button press, using index versus middle finger. The baseline task is a visual pattern matching task (e.g. "//\//" versus "\/\\\\") also with response via button press. Total task duration is 3:20 minutes.

Spatial N-back task paradigm: The paradigm is block design, moving pseudo-randomly through 5 blocks of fixation/rest (13.4 s), and 4 blocks each of 0-back (25.2 s), and 1-back (25.2 s) tasks, each preceded by a 1.0 s text cue. Participants are provided two buttons for each hand, corresponding to the 4 corners/quadrants of the screen. The N-back blocks consist of a 11 visual stimuli presented for a duration of 750ms each followed by a 1350ms blank period. The stimulus is a yellow dot shown in one of the four quadrants of the screen. A central fixation cross remains present throughout. During 0-back blocks, the participant is visually prompted ("*This dot*") to press the button corresponding to the location of the current dot. During 1-back blocks, the participant is visually prompted ("*Previous dot*") to press

the button corresponding to the dot from the previous stimulus. Visual feedback is provided (the fixation cross transiently turns green/red for correct/incorrect responses). The total task duration is 4:53, comprising 304 fMRI volumes of TR 900ms.

Movie-watching task paradigm: This 14-minute movie watching task was drawn from the Human Connectome Project Young Adult Movie task #2 (https://balsa.wustl.edu/project?project=HCP_YA). This includes three short clips from movies Inception (2010), The Social Network (2010) and Ocean's Eleven (2001), of approximately 4 minutes each, separated by 20 s with the word "REST" displayed. We omitted the "test-retest2" video clip that was also shown during the HCP study. We chose moving-watching rather than resting-state fMRI due to better maintenance of attention, reduced chance of sleep, and common dynamic activation of networks across individuals relevant to cognition and emotion[19,20].

Centralized Post-processing of Image Data for Clinical Reporting

Gradient distortion correction (GDC) was disabled during scanner-based reconstruction because it was not uniformly available across all sequences. Instead, GDC was applied during post-processing to ensure consistent correction across the dataset, excepting for sequence and scanner software versions where scanner-based GDC was mandatory and could not be disabled.

Multi-planar reformats (MPR) were generated for 3D T1w MPRAGE and 3D T2w FLAIR acquisitions, using a centralized automated processing pipeline. Images were re-sliced in (i) a sagittal plane, (ii) an oblique axial plane parallel to the hippocampal axis, (determined from the FreeSurfer segmentation of the hippocampi), and (iii) an oblique coronal plane perpendicular to the hippocampus. Voxel values were first interpolated in image space to 0.8 x 0.8 x 0.67mm in the appropriate plane, then averaged over 3 consecutive slices for a final output slice thickness of 2mm.

A multi-echo susceptibility weighted image was generated from the 3D multi-echo gradient echo data. First, a SWI image was calculated for each echo, using interpolation 2x in-plane via zero-padding, low pass filtering in k-space using a Hann window (with window size linearly increasing with TE[21,22]), and the classical approach to phase mask calculation[23]. The final image was obtained by averaging across all 5 per-echo SWI maps.

Trace maps and Apparent Diffusion Coefficient (ADC) maps were generated from the multi-shell diffusion weighted imaging data pre-processed using MRtrix3_connectome[24]. Trace maps were generated from b=1000 mm/s^2 data, as the mean signal across acquired volumes. ADC maps were calculated from the b=0, 300 and 1000 mm/s^2 shells using MRtrix3.[25]

Finally, all images for clinical reporting were re-converted into a single DICOM series and exported to the clinical PACS service for neuroradiologist review.

Schema for Classification of MRI Findings from Radiologist Reports

Table S1: Categories for classifying MRI Findings from the Radiologist Report

Category	Reported MRI Findings<br>*(for checkboxes, select all that apply)*
1. Was an epileptogenic abnormality identified?	Epileptogenic / Possibly Epileptogenic / No
2. Radiologist report says motion affected?	Not mentioned / Mild or Slightly affected / Motion degraded
3. Radiologist recommends repeat or further imaging?	Yes / No
4. Malformations of Cortical Development	☐ Focal Cortical Dysplasia – BOSD<br>☐ Focal Cortical Dysplasia – not BOSD<br>☐ Tuberous Sclerosis<br>☐ Grey Matter Heterotopia – Periventricular (PVNH)<br>☐ Grey Matter Heterotopia – Subcortical Nodular<br>☐ Grey Matter Heterotopia – Band Heterotopia<br>☐ Polymicrogyria<br>☐ Hypothalamic Hamartoma<br>☐ Meningocele<br>☐ Encephalocele<br>☐ Corpus Callosum Agenesis/Dysgenesis<br>☐ Chiari Malformation<br>☐ Other Cortical Malformation (e.g. schizencephaly, lissencephaly, hemimegalencephaly)
5. Hippocampus & Amygdala	☐ Hippocampal Sclerosis<br>☐ Hippocampal Atrophy (without sclerosis)<br>☐ Hippocampal Dysplasia<br>☐ Incomplete Hippocampal Inversion / Malrotation (HIMAL)<br>☐ Other Hippocampal finding
	☐ Amygdala Atrophy<br>☐ Amygdala Enlargement<br>☐ Amygdala Increased T2 signal<br>☐ Other Amygdala finding
6. Vascular & Tumours	☐ Arteriovenous Malformation<br>☐ Developmental Venous Anomaly<br>☐ Cavernoma<br>☐ Aneurysm<br>☐ Other Vascular Malformation
	☐ Glioneuronal Tumour (DNET, ganglioglioma)<br>☐ Glioma<br>☐ Meningioma<br>☐ Cerebral Metastasis<br>☐ Other Tumor (e.g. pituitary, pineal)<br>☐ Non-specific Focal Mass

7. Injury & Inflammation	☐ Atrophy – Global Cerebral<br>☐ Atrophy – Focal<br>☐ Atrophy – Cerebellar<br>☐ Stroke – Infarction<br>☐ Stroke – Hemorrhage<br>☐ Perinatal Hypoxic Ischemic Injury<br>☐ Demyelination<br>☐ Post-encephalitic (infectious/autoimmune)<br>☐ Vasculitis<br>☐ Post infection lesion<br>☐ Traumatic gliosis/encephalomalacia<br>☐ Other gliosis/encephalomalacia<br>☐ Cytotoxic lesion of corpus callosum (splenium)
8. Miscellaneous MRI findings	☐ Surgery – Anterior Temporal Lobectomy<br>☐ Surgery – Other cortical resection<br>☐ Surgery – Other intracranial surgery<br>☐ Post-radiation changes
	☐ Enlarged perivascular spaces (Virchow–Robin)<br>☐ Arachnoid cyst<br>☐ Other cyst (e.g. Rathke's cleft, Choroid fissure)
	☐ White-matter hyperintensities<br>☐ Cerebral microhemorrhages (including amyloid angiopathy)<br>☐ Hydrocephalus / ventriculomegaly<br>☐ Non-specific focal T2/FLAIR/SWI signal

Supplementary Results

Incidence of Epileptogenic Lesions and Other MRI Findings

Table S2: Epileptogenic Lesions in the AEP

	FUS (N=147)	NDE (N=325)	DRE (N=467)	CTRL (N=392)	TOTAL (N=1331)
Hippocampal Sclerosis	1 (0.7%)	8 (2.5%)	63 (13.5%)	0 (0.0%)	72 (5.4%)
Atrophy - Focal	4 (2.7%)	12 (3.7%)	42 (9.0%)	2 (0.5%)	60 (4.5%)
Gliosis / Encephalomalacia	2 (1.4%)	9 (2.8%)	35 (7.5%)	0 (0.0%)	46 (3.5%)
Focal Cortical Dysplasia	2 (1.4%)	10 (3.1%)	37 (7.9%)	0 (0.0%)	49 (3.7%)
Glioneuronal Tumour	0 (0.0%)	9 (2.8%)	23 (4.9%)	2 (0.5%)	34 (2.6%)
Grey Matter Heterotopia	3 (2.0%)	11 (3.4%)	22 (4.7%)	4 (1.0%)	40 (3.0%)
Polymicrogyria	1 (0.7%)	5 (1.5%)	14 (3.0%)	1 (0.3%)	21 (1.6%)
Cavernoma	5 (3.4%)	11 (3.4%)	16 (3.4%)	5 (1.3%)	37 (2.8%)
Other Cortical Malformations	1 (0.7%)	4 (1.2%)	8 (1.7%)	0 (0.0%)	13 (1.0%)
Glioma	1 (0.7%)	2 (0.6%)	9 (1.9%)	0 (0.0%)	12 (0.9%)
Stroke – Cortical Infarct or ICH	3 (2.0%)	5 (1.5%)	8 (1.7%)	1 (0.3%)	17 (1.3%)
Arteriovenous Malformation	0 (0.0%)	2 (0.6%)	1 (0.2%)	0 (0.0%)	3 (0.2%)

Table S3: Potentially Epileptogenic Lesions in the AEP

	FUS (N=147)	NDE (N=325)	DRE (N=467)	CTRL (N=392)	TOTAL (N=1331)
Encephalocele	15 (10.2%)	39 (12.0%)	60 (12.8%)	26 (6.6%)	140 (10.5%)
Amygdala Finding	1 (0.7%)	20 (6.2%)	18 (3.9%)	0 (0.0%)	39 (2.9%)
Hippocampal Atrophy	0 (0.0%)	4 (1.2%)	14 (3.0%)	0 (0.0%)	18 (1.4%)
Meningioma	4 (2.7%)	1 (0.3%)	4 (0.9%)	3 (0.8%)	12 (0.9%)
Other Tumour	3 (2.0%)	7 (2.2%)	1 (0.2%)	1 (0.3%)	12 (0.9%)
Stroke - Subcortical Infarct	0 (0.0%)	2 (0.6%)	5 (1.1%)	2 (0.5%)	9 (0.7%)
Corpus Callosum Agenesis/Dysgenesis	0 (0.0%)	1 (0.3%)	1 (0.2%)	1 (0.3%)	3 (0.2%)
Demyelination	0 (0.0%)	1 (0.3%)	2 (0.4%)	1 (0.3%)	4 (0.3%)
Previous Neurosurgery	0 (0.0%)	4 (1.2%)	61 (13.1%)	0 (0.0%)	65 (4.9%)

Table S4: Other MRI Findings in the AEP

	FUS (N=147)	NDE (N=325)	DRE (N=467)	CTRL (N=392)	TOTAL (N=1331)
Non-specific Focal T2/FLAIR or SWI signal	52 (35.4%)	97 (29.8%)	129 (27.6%)	124 (31.6%)	402 (30.2%)
Incomplete Hippocampal Inversion/Malrotation	32 (21.8%)	89 (27.4%)	107 (22.9%)	83 (21.2%)	311 (23.4%)
White Matter Hyperintensities	37 (25.2%)	71 (21.8%)	95 (20.3%)	96 (24.5%)	299 (22.5%)
Atrophy - Global Cerebral	33 (22.4%)	56 (17.2%)	111 (23.8%)	39 (9.9%)	239 (18.0%)
Other Cyst	9 (6.1%)	32 (9.8%)	45 (9.6%)	27 (6.9%)	113 (8.5%)
Arachnoid Cyst	2 (1.4%)	16 (4.9%)	22 (4.7%)	13 (3.3%)	53 (4.0%)
Enlarged Perivascular Spaces	2 (1.4%)	7 (2.2%)	12 (2.6%)	3 (0.8%)	24 (1.8%)
Chiari Malformation	0 (0.0%)	4 (1.2%)	6 (1.3%)	2 (0.5%)	12 (0.9%

'Other MRI findings' mostly had similar prevalence in healthy controls to people with seizures (Table S4). There was no difference between groups for isolated Non-specific Foci of T2/FLAIR or SWI signal abnormalities (29.4% in people with seizures versus 31.7% in controls, chi-square test p=.44), multiple white matter T2 hyperintensities (21.5% in people with seizures versus 24.6% in healthy controls, p=0.25), incomplete hippocampal inversion / hippocampal malrotation (24.3% in people with seizures versus 21.2% in healthy controls, p=.26), pituitary and pineal cysts (9.2% in people with seizures versus 6.9% in controls, p=.22) and arachnoid cysts (4.2% in people with seizures versus 3.3% in controls, p=.58).

Global cerebral atrophy was reported in 21.4% of people with seizures but only 10% controls (p<.001). Within people with seizures, rates of global cerebral atrophy were similar (FUS 22.6%, NDE 17.2%, DRE 23.9%, chi-squared test, p=.07 uncorrected). A logistic regression model for global cerebral atrophy, having independent variables of group and age, showed that each of the seizure groups was at significantly increased odds of global cerebral atrophy compared to controls (FUS: OR 3.0, 95% CI 1.8-5.2; NDE OR 2.3, 95% CI 1.5-3.7; DRE: OR 3.7, 95% CI 2.5-5.6) while increasing age was also a significant risk for atrophy in common to all groups (OR 1.69 per decade, 95% CI 1.51-1.89).

Supplementary References

1. Bernasconi A, Cendes F, Theodore WH, et al. Recommendations for the use of structural magnetic resonance imaging in the care of patients with epilepsy: A consensus report from the International League Against Epilepsy Neuroimaging Task Force. Epilepsia. 2019;60:1054–1068.
2. Glasser MF, Smith SM, Marcus DS, et al. The Human Connectome Project's neuroimaging approach. Nat Neurosci. Nature Publishing Group; 2016;19:1175–1187.
3. Littlejohns TJ, Holliday J, Gibson LM, et al. The UK Biobank imaging enhancement of 100,000 participants: rationale, data collection, management and future directions. Nat Commun. 2020;11:2624.
4. Casey BJ, Cannonier T, Conley MI, et al. The Adolescent Brain Cognitive Development (ABCD) study: Imaging acquisition across 21 sites. Dev Cogn Neurosci. 2018;32:43–54.
5. Piredda GF, Hilbert T, Granziera C, et al. Quantitative brain relaxation atlases for personalized detection and characterization of brain pathology. Magn Reson Med. 2020;83:337–351.
6. Mussard E, Hilbert T, Forman C, Meuli R, Thiran J-P, Kober T. Accelerated MP2RAGE imaging using Cartesian phyllotaxis readout and compressed sensing reconstruction. Magn Reson Med. 2020;84:1881–1894.
7. Hilbert T, Sumpf TJ, Weiland E, et al. Accelerated T2 mapping combining parallel MRI and model-based reconstruction: GRAPPATINI. J Magn Reson Imaging. 2018;48:359–368.
8. Jackson GD, Connelly A, Duncan JS, Grünewald RA, Gadian DG. Detection of hippocampal pathology in intractable partial epilepsy: increased sensitivity with quantitative magnetic resonance T2 relaxometry. Neurology. 1993;43:1793–1799.
9. Mito R, Pedersen M, Pardoe H, et al. Exploring individual fixel-based white matter abnormalities in epilepsy. Brain Commun. 2024;6:fcad352.
10. Hutter J, Tournier JD, Price AN, et al. Time-efficient and flexible design of optimized multishell HARDI diffusion. Magn Reson Med. 2018;79:1276–1292.
11. Cauley SF, Polimeni JR, Bhat H, Wald LL, Setsompop K. Interslice leakage artifact reduction technique for simultaneous multislice acquisitions. Magn Reson Med. 2014;72:93–102.
12. Setsompop K, Cohen-Adad J, Gagoski BA, et al. Improving diffusion MRI using simultaneous multi-slice echo planar imaging. NeuroImage. 2012;63:569–580.
13. Tournier J-D, Christiaens D, Hutter J, et al. A data-driven approach to optimising the encoding for multi-shell diffusion MRI with application to neonatal imaging. NMR Biomed. 2020;33:e4348.
14. Tournier J-D, Calamante F, Connelly A. Determination of the appropriate b value and number of gradient directions for high-angular-resolution diffusion-weighted imaging. NMR Biomed. 2013;26:1775–1786.
15. Sotiropoulos SN, Moeller S, Jbabdi S, et al. Effects of image reconstruction on fiber orientation mapping from multichannel diffusion MRI: Reducing the noise floor using SENSE. Magn Reson Med. 2013;70:1682–1689.
16. Manzano Patron JP, Moeller S, Andersson JLR, Ugurbil K, Yacoub E, Sotiropoulos SN. Denoising diffusion MRI: Considerations and implications for analysis. Imaging Neurosci. 2024;2:imag–2–00060.
17. Tahayori B, Smith RE, Vaughan DN, et al. Robust-tedana: An automated denoising pipeline for multi-echo fMRI data [online]. bioRxiv; 2025. p. 2025.06.16.660050.
18. Andersson JLR, Skare S, Ashburner J. How to correct susceptibility distortions in spin-echo echo-planar images: application to diffusion tensor imaging. NeuroImage. 2003;20:870–888.

19. Finn ES, Bandettini PA. Movie-watching outperforms rest for functional connectivity-based prediction of behavior. NeuroImage. 2021;235:117963.
20. Meer JN van der, Breakspear M, Chang LJ, Sonkusare S, Cocchi L. Movie viewing elicits rich and reliable brain state dynamics. Nat Commun. Nature Publishing Group; 2020;11:5004.
21. Haacke EM, Xu Y, Cheng Y-CN, Reichenbach JR. Susceptibility weighted imaging (SWI). Magn Reson Med. 2004;52:612–618.
22. Denk C, Rauscher A. Susceptibility weighted imaging with multiple echoes. J Magn Reson Imaging. 2010;31:185–191.
23. Wang Y, Yu Y, Li D, et al. Artery and vein separation using susceptibility-dependent phase in contrast-enhanced MRA. J Magn Reson Imaging. 2000;12:661–670.
24. Smith R, Connelly A. MRtrix3_connectome: A BIDS Application for quantitative structural connectome construction. Organ Hum Brain Mapp. Rome, Italy; 2019.
25. Tournier J-D, Smith R, Raffelt D, et al. *MRtrix3*: A fast, flexible and open software framework for medical image processing and visualisation. NeuroImage. 2019;202:116137.

Australian Epilepsy Protocol MRI Sequence Parameters

AEP MRI Diffusion Gradient Table File

See attached file: *AEP_20240716.dvs*

AEP MRI Protocol for Siemens 3T Prisma (XA60)

Table of contents

\\USER
2025_MBC_PROTOCOLS_XA60
SB.2020.353 AEP study - 64 Channel
SB.2020.353 - AEP MRI ACQUISITION PROTOCOL v22- 64 channel
Localiser-
T2w_TSE_Axial
MEGRE_Ax_RL-
T1w_Sag_AP-
T2wFLAIR_Sag_AP-
T2w_CorOblHipp_RL-
T2map_CorOblHipp_RL-
T2w_AxOblTempPole_RL-
DWI_Ax_AP_52Dir-
DWI_Ax_AP_51Dir_InvROPE-
T1map_MP2RAGE_Sag_AP- as imported *
Localiser-OptionalREPEAT-
T1w_Sag_AP-OptionalREPEAT-
T2wFLAIR_Sag_AP-OptionalREPEAT-
T2w_CorOblHipp_RL-OptionalREPEAT-
fMRI_AxObl_AP_Pseudoword-
FMap_AxObl_AP-
FMap_AxObl_AP_InvROPE-
fMRI_AxObl_AP_OneBack-
FMap_AxObl_AP-
FMap_AxObl_AP_InvROPE-
fMRI_AxObl_AP_Movie-
FMap_AxObl_AP-
FMap_AxObl_AP_InvROPE-

\\USER\2025_MBC_PROTOCOLS_XA60\SB.2020.353 AEP study - 64 Channel\SB.2020.353 - AEP MRI ACQUISITION PROTOCOL v22- 64 channel\Localiser-

TA: 37 sec Coil Selection: Auto Voxel Size: 0.5×0.5×7.0 mm³ Acc:: None Rel. SNR: 1.00

Properties

Start measurement without further preparation	Off
Wait for User to Start	Off
Start measurements	Single Measurement
Prio Recon	Off
Auto Open Inline Display	Off
Auto Close Inline Display	Off
Load Images to MR View&GO	On
Auto Store Images	On
Disable auto transfer to PACS	Off
Load Images to Stamp Segments	On
Load Images to Graphic Segments	Off
Graphic segment	Default
Inline Movie	Off

Routine

Slice Group	1
Slices	3
Distance Factor	100 %
Position	Isocenter
Orientation	Sagittal
Phase Encoding Dir.	A >> P
Slice Group	2
Slices	3
Distance Factor	100 %
Position	Isocenter
Orientation	Sagittal
Phase Encoding Dir.	A >> P
Slice Group	3
Slices	3
Distance Factor	100 %
Position	Isocenter
Orientation	Sagittal
Phase Encoding Dir.	A >> P
Phase Oversampling	0 %
FOV Read	250 mm
FOV Phase	100.0 %
Slice Thickness	7.0 mm
TR	8.6 ms
TE	4.00 ms
Averages	2
Concatenations	9
AutoAlign	---

Contrast - Common

TR	8.6 ms
TE	4.00 ms
TD	0.00 ms
MTC	Off

Contrast - Common

Magn. Preparation	None
Flip Angle	20 deg
Fat-Water Contrast	Standard
Dark Blood	Off
Contrasts	1
SWI	Off
Reconstruction	Magnitude

Contrast - Dynamic

Dynamic Mode	Standard
Measurements	1
Multiple Series	Each Measurement

Resolution - Common

FOV Read	250 mm
FOV Phase	100.0 %
Slice Thickness	7.0 mm
Base Resolution	256
Phase Resolution	90 %
Interpolation	On

Resolution - Acceleration

Acceleration Mode	None
Deep Resolve	Off
Phase Partial Fourier	Off
Asymmetric Echo	Allowed

Resolution - Filter

Raw Filter	Off
Elliptical Filter	On
Distortion Correction	2D
Normalize	Prescan
Image Filter	Off

Geometry - Common

Slice Group	1
Slices	3
Distance Factor	100 %
Position	Isocenter
Orientation	Sagittal
Phase Encoding Dir.	A >> P
Slice Group	2
Slices	3
Distance Factor	100 %
Position	Isocenter
Orientation	Sagittal
Phase Encoding Dir.	A >> P
Slice Group	3
Slices	3

Geometry - Common

Distance Factor	100 %
Position	Isocenter
Orientation	Sagittal
Phase Encoding Dir.	A >> P
Phase Oversampling	0 %
FOV Read	250 mm
FOV Phase	100.0 %
Slice Thickness	7.0 mm
TR	8.6 ms
Multi-Slice Mode	Sequential
Series	Interleaved
Concatenations	9

Geometry - AutoAlign

Slice Group	1
Position	Isocenter
Orientation	Sagittal
Phase Encoding Dir.	A >> P
Slice Group	2
Position	Isocenter
Orientation	Sagittal
Phase Encoding Dir.	A >> P
Slice Group	3
Position	Isocenter
Orientation	Sagittal
Phase Encoding Dir.	A >> P
AutoAlign	---
Initial Position	Isocenter
L	0.0 mm
P	0.0 mm
H	0.0 mm
Initial Orientation	Sagittal
Initial Rotation	0.00 deg

Geometry - Saturation

Saturation Mode	Standard
Special Saturation	None

Geometry - Tim Planning Suite

Set-n-Go Protocol	Off
Table Position	0 mm
Table Position	H
Inline Composing	Off

System - Miscellaneous

Coil Selection	ACS All but spine
Radial Sorting	Off
MSMA	S - C - T
Sagittal	R >> L
Coronal	P >> A
Transversal	F >> H
Coil Combination	Adaptive Combine
Matrix Optimization	Off
Coil Focus	Flat

System - Adjustments

Adjustment Strategy	Standard
B0 Shim	Tune up
B1 Shim	TrueForm
Adjustment Tolerance	Auto
Adjust with Body Coil	Off
Confirm Frequency	Never
Assume Silicone	Off

System - Adjust Volume

Position	Isocenter
Orientation	Transversal
Rotation	0.00 deg
A >> P	263 mm
R >> L	350 mm
F >> H	350 mm
Reset	Off

System - pTx

B1 Shim	TrueForm
Excitation	Slice-sel.
LR Balancing	Off

System - Tx/Rx

Frequency 1H	123.237845 MHz
? Ref. Amplitude 1H	0.000 V
Reset	Off
Image Scaling	1.000

Physio - Signal

1st Signal/Mode	None
TR	8.6 ms
Segments	1
Concatenations	9

Physio - Cardiac

Fat-Water Contrast	Standard
Magn. Preparation	None
Dark Blood	Off
FOV Read	250 mm
FOV Phase	100.0 %
Phase Resolution	90 %

Physio - PACE

Resp. Control	Off
Concatenations	9

Inline - Liver

Liver Registration	Off
Save Original Images	On

Inline - Subtraction

Subtract	Off
Measurements	1
StdDev	Off

Inline - Subtraction

Save Original Images	On

Inline - MIP

MIP Sag	Off
MIP Cor	Off
MIP Tra	Off
MIP Time	Off
Radial MIP	Off
Save Original Images	On
MPR Sag	Off
MPR Cor	Off
MPR Tra	Off

Inline - Soft Tissue

Wash-in	Off
Wash-out	Off
TTP	Off
PEI	Off
MIP Time	Off
Measurements	1

Inline - Composing

Inline Composing	Off

Inline - MapIt

MapIt	None
Flip Angle	20 deg
Measurements	1
Contrasts	1
TE	4.00 ms
TR	8.6 ms
Save Original Images	On

Sequence - Part 1

Sequence Name	fl
Dimension	2D
Excitation	Slice-sel.
RF Pulse Type	Normal
Gradient Mode	Normal
Flow Compensation	None
Bandwidth	320 Hz/Px
Asymmetric Echo	Allowed
Segments	1

Sequence - Part 2

Introduction	On
RF Spoiling	On
Acoustic noise reduction	Off

Sequence - Assistant

SAR Assistant	Off
Allowed Delay	0 s

\\USER\2025_MBC_PROTOCOLS_XA60\SB.2020.353 AEP study - 64 Channel\SB.2020.353 - AEP MRI ACQUISITION PROTOCOL v22- 64 channel\T2w_TSE_Axial

TA: 3:38 min Coil Selection: Auto Voxel Size: 0.2×0.2×3.0 mm³ Acc:: None Rel. SNR: 1.00

Properties

Start measurement without further preparation	On
Wait for User to Start	Off
Start measurements	Single Measurement
Prio Recon	Off
Auto Open Inline Display	Off
Auto Close Inline Display	Off
Load Images to MR View&GO	On
Auto Store Images	On
Disable auto transfer to PACS	Off
Load Images to Stamp Segments	Off
Load Images to Graphic Segments	Off
Graphic segment	Default
Inline Movie	Off

Routine

Slice Group	1
Slices	46
Distance Factor	0 %
Position	Isocenter
Orientation	T > C-16.0
Phase Encoding Dir.	R >> L
Phase Oversampling	5 %
FOV Read	208 mm
FOV Phase	84.4 %
Slice Thickness	3.0 mm
TR	4500.0 ms
TE	83.00 ms
Averages	1
Concatenations	2
AutoAlign	---

Contrast - Common

TR	4500.0 ms
TE	83.00 ms
TD	0.00 ms
MTC	Off
Magn. Preparation	None
Flip Angle Mode	Constant
Flip Angle	120 deg
Fat-Water Contrast	Standard
Dark Blood	Off
Contrasts	1
Wrap-up Magn.	None
Reconstruction	Magnitude

Contrast - Dynamic

Dynamic Mode	Standard
Measurements	1

Contrast - Dynamic

Multiple Series	Each Measurement

Resolution - Common

FOV Read	208 mm
FOV Phase	84.4 %
Slice Thickness	3.0 mm
Base Resolution	448
Phase Resolution	100 %
Interpolation	On

Resolution - Acceleration

Acceleration Mode	None
Deep Resolve	Off
Phase Partial Fourier	Off

Resolution - Filter

Raw Filter	Off
Elliptical Filter	Off
Distortion Correction	2D
Normalize	Prescan
Image Filter	On

Geometry - Common

Slice Group	1
Slices	46
Distance Factor	0 %
Position	Isocenter
Orientation	T > C-16.0
Phase Encoding Dir.	R >> L
Phase Oversampling	5 %
FOV Read	208 mm
FOV Phase	84.4 %
Slice Thickness	3.0 mm
TR	4500.0 ms
Multi-Slice Mode	Interleaved
Series	Interleaved
Concatenations	2

Geometry - AutoAlign

Slice Group	1
Position	Isocenter
Orientation	T > C-16.0
Phase Encoding Dir.	R >> L
AutoAlign	---
Initial Position	Isocenter
L	0.0 mm
A	0.0 mm
F	0.0 mm
Initial Orientation	T > C

Geometry - AutoAlign

T > C	-16.00
> S	0.00
Initial Rotation	90.00 deg

Geometry - Navigator

Geometry - Saturation

Special Saturation	None

Geometry - Tim Planning Suite

Set-n-Go Protocol	Off
Table Position	0 mm
Table Position	H
Inline Composing	Off

System - Miscellaneous

Coil Selection	ACS All but spine
Radial Sorting	Off
MSMA	S - C - T
Sagittal	R >> L
Coronal	A >> P
Transversal	F >> H
Coil Combination	Adaptive Combine
Matrix Optimization	Off
Coil Focus	Flat

System - Adjustments

Adjustment Strategy	Standard
B0 Shim	Standard
B1 Shim	TrueForm
Adjustment Tolerance	Auto
Adjust with Body Coil	Off
Confirm Frequency	Never
Assume Silicone	Off

System - Adjust Volume

Position	Isocenter
Orientation	T > C-16.0
Rotation	90.00 deg
R >> L	176 mm
A >> P	208 mm
F >> H	138 mm
Reset	Off

System - pTx

B1 Shim	TrueForm
LR Balancing	Off

System - Tx/Rx

Frequency 1H	123.237845 MHz
? Ref. Amplitude 1H	0.000 V
Reset	Off
Image Scaling	1.000

Physio - Signal

1st Signal/Mode	None
TR	4500.0 ms
Concatenations	2

Physio - Cardiac

Fat-Water Contrast	Standard
Magn. Preparation	None
Dark Blood	Off
FOV Read	208 mm
FOV Phase	84.4 %
Phase Resolution	100 %
Motion Correction	None

Physio - PACE

Resp. Control	Off
Concatenations	2

Inline - Subtraction

Subtract	Off
Measurements	1
StdDev	Off
Motion Correction	None
Save Original Images	On

Inline - MIP

MIP Sag	Off
MIP Cor	Off
MIP Tra	Off
MIP Time	Off
Radial MIP	Off
Save Original Images	On
MPR Sag	Off
MPR Cor	Off
MPR Tra	Off

Inline - Composing

Inline Composing	Off

Sequence - Part 1

Sequence Name	tse_rr
Dimension	2D
RF Pulse Type	Fast
Gradient Mode	Fast
Flow Compensation	Read
Bandwidth	180 Hz/Px
Echo Spacing	10.4 ms
Free Echo Spacing	Off
Define	Turbo Factor
Turbo Factor	18
Echo Trains per Slice	23

Sequence - Part 2

Introduction	On
Phase Correction	Automatic

Sequence - Part 2

Compensate T2 Decay	Off
Fast Mode	Off
WARP	Off
Red. EC Sensitivity	Off
Acoustic noise reduction	Off
Reduce Motion Sens.	On
Motion Correction	None

Sequence - Assistant

SAR Assistant	Off
Allowed Delay	60 s

\\USER\2025_MBC_PROTOCOLS_XA60\SB.2020.353 AEP study - 64 Channel\SB.2020.353 - AEP MRI ACQUISITION PROTOCOL v22- 64 channel\MEGRE_Ax_RL-

TA: 3:39 min Coil Selection: Auto Voxel Size: 1.2×1.2×1.2 mm³ Acc:: 3 Rel. SNR: 1.00

Properties

Start measurement without further preparation	On
Wait for User to Start	On
Start measurements	Single Measurement
Prio Recon	Off
Auto Open Inline Display	Off
Auto Close Inline Display	Off
Load Images to MR View&GO	On
Auto Store Images	On
Disable auto transfer to PACS	Off
Load Images to Stamp Segments	Off
Load Images to Graphic Segments	Off
Graphic segment	Default
Inline Movie	Off

Routine

Slab Group	1
Slabs	1
Distance Factor	20 %
Position	Isocenter
Orientation	Transversal
Phase Encoding Dir.	R >> L
Slices per Slab	120
Phase Oversampling	10 %
Slice Oversampling	0.0 %
FOV Read	230 mm
FOV Phase	81.3 %
Slice Thickness	1.20 mm
TR	30.0 ms
TE 1	5.84 ms
TE 2	10.63 ms
TE 3	15.42 ms
TE 4	20.21 ms
TE 5	25.00 ms
Averages	1
Concatenations	1
AutoAlign	---

Contrast - Common

TR	30.0 ms
TE 1	5.84 ms
TE 2	10.63 ms
TE 3	15.42 ms
TE 4	20.21 ms
TE 5	25.00 ms
MTC	Off
Magn. Preparation	None
Flip Angle	15 deg
Fat-Water Contrast	Standard

Contrast - Common

Dark Blood	Off
Contrasts	5
SWI	Off
Reconstruction	Magn./Phase

Contrast - Dynamic

Dynamic Mode	Standard
Measurements	1
Multiple Series	Each Measurement

Resolution - Common

FOV Read	230 mm
FOV Phase	81.3 %
Slice Thickness	1.20 mm
Base Resolution	192
Phase Resolution	100 %
Slice Resolution	100 %
Interpolation	Off

Resolution - Acceleration

Acceleration Mode	GRAPPA
Reference Scans	Integrated
Acceleration Factor PE	3
Reference Lines PE	24
Acceleration Factor 3D	1
Deep Resolve	Off
Phase Partial Fourier	Off
Slice Partial Fourier	Off
Asymmetric Echo	Off
Elliptical Scanning	On

Resolution - Filter

Raw Filter	Off
Elliptical Filter	Off
Distortion Correction	2D
Normalize	Prescan
Image Filter	Off

Geometry - Common

Slab Group	1
Slabs	1
Distance Factor	20 %
Position	Isocenter
Orientation	Transversal
Phase Encoding Dir.	R >> L
Slices per Slab	120
Phase Oversampling	10 %
Slice Oversampling	0.0 %
FOV Read	230 mm

Geometry - Common

FOV Phase	81.3 %
Slice Thickness	1.20 mm
TR	30.0 ms
Multi-Slice Mode	Interleaved
Series	Interleaved
Concatenations	1

Geometry - AutoAlign

Slab Group	1
Position	Isocenter
Orientation	Transversal
Phase Encoding Dir.	R >> L
AutoAlign	---
Initial Position	Isocenter
L	0.0 mm
P	0.0 mm
H	0.0 mm
Initial Orientation	Transversal
Initial Rotation	90.00 deg

Geometry - Saturation

Saturation Mode	Standard
Special Saturation	None

Geometry - Tim Planning Suite

Set-n-Go Protocol	Off
Table Position	0 mm
Table Position	H
Inline Composing	Off

System - Miscellaneous

Coil Selection	ACS All but spine
Radial Sorting	Off
MSMA	S - C - T
Sagittal	R >> L
Coronal	A >> P
Transversal	F >> H
Coil Combination	Adaptive Combine
Matrix Optimization	Off
Coil Focus	Flat

System - Adjustments

Adjustment Strategy	Standard
B0 Shim	Standard
B1 Shim	TrueForm
Adjustment Tolerance	Auto
Adjust with Body Coil	Off
Confirm Frequency	Never
Assume Silicone	Off

System - Adjust Volume

Position	Isocenter
Orientation	Transversal
Rotation	90.00 deg

System - Adjust Volume

R >> L	187 mm
A >> P	230 mm
F >> H	144 mm
Reset	Off

System - pTx

B1 Shim	TrueForm
Excitation	Slab-sel.
LR Balancing	Off

System - Tx/Rx

Frequency 1H	123.237845 MHz
? Ref. Amplitude 1H	0.000 V
Reset	Off
Image Scaling	1.000

Physio - Signal

1st Signal/Mode	None
TR	30.0 ms
Segments	1
Concatenations	1

Physio - Cardiac

Fat-Water Contrast	Standard
Magn. Preparation	None
Dark Blood	Off
FOV Read	230 mm
FOV Phase	81.3 %
Phase Resolution	100 %

Physio - PACE

Resp. Control	Off
Concatenations	1

Inline - Liver

Liver Registration	Off
Save Original Images	On

Inline - Subtraction

Subtract	Off
Measurements	1
StdDev	Off
Save Original Images	On

Inline - MIP

MIP Sag	Off
MIP Cor	Off
MIP Tra	Off
MIP Time	Off
Radial MIP	Off
Save Original Images	On
MPR Sag	Off
MPR Cor	Off
MPR Tra	Off

Inline - Soft Tissue

Wash-in	Off
Wash-out	Off
TTP	Off
PEI	Off
MIP Time	Off
Measurements	1

Inline - Composing

Inline Composing	Off

Inline - MapIt

MapIt	None
Flip Angle	15 deg
Measurements	1
Contrasts	5
TE 1	5.84 ms
TE 2	10.63 ms
TE 3	15.42 ms
TE 4	20.21 ms
TE 5	25.00 ms
TR	30.0 ms
Save Original Images	On

Sequence - Part 1

Sequence Name	fl_r
Dimension	3D
Excitation	Slab-sel.
RF Pulse Type	Fast
Readout Mode	Monopolar
Gradient Mode	Normal
Flow Compensation 1	On
Flow Compensation 2	None
Flow Compensation 3	None
Flow Compensation 4	None
Flow Compensation 5	None
Bandwidth 1	310 Hz/Px
Bandwidth 2	310 Hz/Px
Bandwidth 3	310 Hz/Px
Bandwidth 4	310 Hz/Px
Bandwidth 5	310 Hz/Px
Asymmetric Echo	Off
Segments	1

Sequence - Part 2

Introduction	Off
RF Spoiling	On
Acoustic noise reduction	Off

Sequence - Assistant

SAR Assistant	Off
Allowed Delay	0 s

\\USER\2025_MBC_PROTOCOLS_XA60\SB.2020.353 AEP study - 64 Channel\SB.2020.353 - AEP MRI ACQUISITION PROTOCOL v22- 64 channel\T1w_Sag_AP-

TA: 4:32 min Coil Selection: Auto Voxel Size: 0.9×0.9×0.9 mm³ Acc:: 2 Rel. SNR: 1.00

Properties

Start measurement without further preparation	On
Wait for User to Start	Off
Start measurements	Single Measurement
Prio Recon	Off
Auto Open Inline Display	Off
Auto Close Inline Display	Off
Load Images to MR View&GO	On
Auto Store Images	On
Disable auto transfer to PACS	Off
Load Images to Stamp Segments	Off
Load Images to Graphic Segments	Off
Graphic segment	Default
Inline Movie	Off

Routine

Slab Group	1
Slabs	1
Distance Factor	50 %
Position	Isocenter
Orientation	Sagittal
Phase Encoding Dir.	A >> P
Slices per Slab	192
Phase Oversampling	0 %
Slice Oversampling	25.0 %
FOV Read	230 mm
FOV Phase	100.0 %
Slice Thickness	0.90 mm
TR	1900.0 ms
TE	2.19 ms
Averages	1
Concatenations	1
AutoAlign	---

Contrast - Common

TR	1900.0 ms
TE	2.19 ms
Magn. Preparation	Non-sel. IR
TI	1000 ms
Flip Angle	8 deg
Fat-Water Contrast	Fast Water Excitation
Dark Blood	Off
Contrasts	1
Reconstruction	Magnitude

Contrast - Dynamic

Dynamic Mode	Standard
Measurements	1
Multiple Series	Each Measurement

Contrast - Dynamic

Reordering	Linear

Resolution - Common

FOV Read	230 mm
FOV Phase	100.0 %
Slice Thickness	0.90 mm
Base Resolution	256
Phase Resolution	100 %
Slice Resolution	100 %
Interpolation	Off

Resolution - Acceleration

Acceleration Mode	GRAPPA
Reference Scans	Integrated
Acceleration Factor PE	2
Reference Lines PE	32
Acceleration Factor 3D	1
Phase Partial Fourier	Off
Slice Partial Fourier	Off
Asymmetric Echo	Allowed
Elliptical Scanning	Off

Resolution - Filter

Raw Filter	Off
Elliptical Filter	Off
Distortion Correction	2D
Normalize	Prescan
Image Filter	Off

Geometry - Common

Slab Group	1
Slabs	1
Distance Factor	50 %
Position	Isocenter
Orientation	Sagittal
Phase Encoding Dir.	A >> P
Slices per Slab	192
Phase Oversampling	0 %
Slice Oversampling	25.0 %
FOV Read	230 mm
FOV Phase	100.0 %
Slice Thickness	0.90 mm
TR	1900.0 ms
Multi-Slice Mode	Single Shot
Series	Interleaved
Concatenations	1

Geometry - AutoAlign

Slab Group	1

Geometry - AutoAlign

Position	Isocenter
Orientation	Sagittal
Phase Encoding Dir.	A >> P
AutoAlign	---
Initial Position	Isocenter
L	0.0 mm
P	0.0 mm
H	0.0 mm
Initial Orientation	Sagittal
Initial Rotation	0.00 deg

Geometry - Navigator

Geometry - Tim Planning Suite

Set-n-Go Protocol	Off
Table Position	0 mm
Table Position	H
Inline Composing	Off

System - Miscellaneous

Coil Selection	Auto Coil Select
Radial Sorting	Off
MSMA	S - C - T
Sagittal	R >> L
Coronal	P >> A
Transversal	F >> H
Coil Combination	Adaptive Combine
Matrix Optimization	Off
Coil Focus	Flat

System - Adjustments

Adjustment Strategy	Standard
B0 Shim	Standard
B1 Shim	TrueForm
Adjustment Tolerance	Auto
Adjust with Body Coil	Off
Confirm Frequency	Never
Assume Silicone	Off

System - Adjust Volume

Position	Isocenter
Orientation	Sagittal
Rotation	0.00 deg
A >> P	230 mm
F >> H	230 mm
R >> L	173 mm
Reset	Off

System - pTx

B1 Shim	TrueForm
Excitation	Non-sel.

System - Tx/Rx

Frequency 1H	123.237845 MHz

System - Tx/Rx

? Ref. Amplitude 1H	0.000 V
Reset	Off
Image Scaling	1.000

Physio - Signal

1st Signal/Mode	None
TR	1900.0 ms
Concatenations	1

Physio - Cardiac

Fat-Water Contrast	Fast Water Excitation
Magn. Preparation	Non-sel. IR
TI	1000 ms
Dark Blood	Off
FOV Read	230 mm
FOV Phase	100.0 %
Phase Resolution	100 %

Physio - PACE

Resp. Control	Off
Concatenations	1

Inline - Subtraction

Subtract	Off
Measurements	1
StdDev	Off
Save Original Images	On

Inline - MIP

MIP Sag	Off
MIP Cor	Off
MIP Tra	Off
MIP Time	Off
Radial MIP	Off
Save Original Images	On
MPR Sag	Off
MPR Cor	Off
MPR Tra	Off

Inline - Composing

Inline Composing	Off

Inline - MapIt

MapIt	None
Flip Angle	8 deg
Measurements	1
Contrasts	1
TE	2.19 ms
TR	1900.0 ms
Save Original Images	On

Sequence - Part 1

Sequence Name	tfl
Dimension	3D

Sequence - Part 1

Excitation	Non-sel.
RF Pulse Type	Fast
Gradient Mode	Performance
Flow Compensation	None
Reordering	Linear
Bandwidth	220 Hz/Px
Echo Spacing	7.38 ms
Asymmetric Echo	Allowed
Turbo Factor	240

Sequence - Part 2

Introduction	Off
RF Spoiling	On
Incr. Gradient Spoiling	Off

Sequence - Assistant

SAR Assistant	Off

\\USER\2025_MBC_PROTOCOLS_XA60\SB.2020.353 AEP study - 64 Channel\SB.2020.353 - AEP MRI ACQUISITION PROTOCOL v22- 64 channel\T2wFLAIR_Sag_AP-

TA: 5:32 min Coil Selection: Auto Voxel Size: 0.9×0.9×0.9 mm³ Acc:: 2 Rel. SNR: 1.00

Properties

Start measurement without further preparation	On
Wait for User to Start	On
Start measurements	Single Measurement
Prio Recon	Off
Auto Open Inline Display	Off
Auto Close Inline Display	Off
Load Images to MR View&GO	On
Auto Store Images	On
Disable auto transfer to PACS	Off
Load Images to Stamp Segments	Off
Load Images to Graphic Segments	Off
Graphic segment	Default
Inline Movie	Off

Routine

Slab Group	1
Slabs	1
Position	Isocenter
Orientation	Sagittal
Phase Encoding Dir.	A >> P
Slices per Slab	192
Phase Oversampling	0 %
Slice Oversampling	0.0 %
FOV Read	230 mm
FOV Phase	100.0 %
Slice Thickness	0.90 mm
TR	5000.0 ms
TE	392.00 ms
Averages	1.0
Concatenations	1
AutoAlign	---

Contrast - Common

TR	5000.0 ms
TE	392.00 ms
MTC	Off
Magn. Preparation	Non-sel. T2 prep. IR
TI	1600 ms
T2 Prep. Duration	125 ms
Flip Angle Mode	T2 Var
Fat-Water Contrast	Standard
Dark Blood	Off
Blood Suppression	Off
Wrap-up Magn.	None
Reconstruction	Magnitude

Contrast - Dynamic

Dynamic Mode	Standard
Measurements	1
Multiple Series	Each Measurement
Reordering	Linear

Resolution - Common

FOV Read	230 mm
FOV Phase	100.0 %
Slice Thickness	0.90 mm
Base Resolution	256
Phase Resolution	100 %
Slice Resolution	100 %
Interpolation	Off

Resolution - Acceleration

Acceleration Mode	GRAPPA
Total Factor	2
Reference Scans	Integrated
Acceleration Factor PE	2
Reference Lines PE	24
Acceleration Factor 3D	1
Phase Partial Fourier	Allowed
Slice Partial Fourier	6/8
Elliptical Scanning	Off

Resolution - Filter

Raw Filter	Off
Elliptical Filter	Off
Distortion Correction	2D
Normalize	Prescan
Image Filter	On

Geometry - Common

Slab Group	1
Slabs	1
Position	Isocenter
Orientation	Sagittal
Phase Encoding Dir.	A >> P
Slices per Slab	192
Phase Oversampling	0 %
Slice Oversampling	0.0 %
FOV Read	230 mm
FOV Phase	100.0 %
Slice Thickness	0.90 mm
TR	5000.0 ms
Concatenations	1

Geometry - AutoAlign

Slab Group	1
Position	Isocenter

Geometry - AutoAlign

Orientation	Sagittal
Phase Encoding Dir.	A >> P
AutoAlign	---
Initial Position	Isocenter
L	0.0 mm
P	0.0 mm
H	0.0 mm
Initial Orientation	Sagittal
Initial Rotation	0.00 deg

Geometry - Navigator

Geometry - Saturation

Special Saturation	None

Geometry - Tim Planning Suite

Set-n-Go Protocol	Off
Table Position	0 mm
Table Position	H
Inline Composing	Off

System - Miscellaneous

Coil Selection	ACS All but spine
Radial Sorting	Off
MSMA	S - C - T
Sagittal	R >> L
Coronal	A >> P
Transversal	F >> H
Coil Combination	Adaptive Combine
Matrix Optimization	Off
Coil Focus	Flat

System - Adjustments

Adjustment Strategy	Standard
B0 Shim	Standard
B1 Shim	TrueForm
Adjustment Tolerance	Auto
Adjust with Body Coil	Off
Confirm Frequency	Never
Assume Silicone	Off

System - Adjust Volume

Position	Isocenter
Orientation	Sagittal
Rotation	0.00 deg
A >> P	230 mm
F >> H	230 mm
R >> L	173 mm
Reset	Off

System - pTx

B1 Shim	TrueForm
Excitation	Non-sel.

System - Tx/Rx

Frequency 1H	123.237845 MHz
? Ref. Amplitude 1H	0.000 V
Reset	Off
Image Scaling	1.000
Gain	High

Physio - Signal

1st Signal/Mode	None
Trigger Delay	0 ms
TR	5000.0 ms
Concatenations	1

Physio - Cardiac

Fat-Water Contrast	Standard
Magn. Preparation	Non-sel. T2 prep. IR
TI	1600 ms
T2 Prep. Duration	125 ms
Dark Blood	Off
FOV Read	230 mm
FOV Phase	100.0 %
Phase Resolution	100 %

Physio - PACE

Resp. Control	Off
Concatenations	1

Inline - Subtraction

Subtract	Off
Measurements	1
StdDev	Off
Save Original Images	On

Inline - MIP

MIP Sag	Off
MIP Cor	Off
MIP Tra	Off
MIP Time	Off
Radial MIP	Off
Save Original Images	On
MPR Sag	Off
MPR Cor	Off
MPR Tra	Off

Inline - Composing

Inline Composing	Off

Sequence - Part 1

Sequence Name	spcir
Dimension	3D
Excitation	Non-sel.
RF Pulse Type	Normal
Gradient Mode	Fast
Flow Compensation	None
Reordering	Linear

Sequence - Part 1

Bandwidth	781 Hz/Px
Echo Spacing	3.60 ms
Turbo Factor	310
Echo Train Duration	947 ms

Sequence - Part 2

Introduction	On

Sequence - Assistant

SAR Assistant	Off
Allowed Delay	0 s

\\USER\2025_MBC_PROTOCOLS_XA60\SB.2020.353 AEP study - 64 Channel\SB.2020.353 - AEP MRI ACQUISITION PROTOCOL v22- 64 channel\T2w_CorOblHipp_RL-

TA: 4:53 min Coil Selection: Auto Voxel Size: 0.4×0.4×2.0 mm³ Acc:: None Rel. SNR: 1.00

Properties

Start measurement without further preparation	On
Wait for User to Start	On
Start measurements	Single Measurement
Prio Recon	Off
Auto Open Inline Display	Off
Auto Close Inline Display	Off
Load Images to MR View&GO	On
Auto Store Images	On
Disable auto transfer to PACS	Off
Load Images to Stamp Segments	On
Load Images to Graphic Segments	Off
Graphic segment	Default
Inline Movie	Off

Routine

Slice Group	1
Slices	36
Distance Factor	0 %
Position	R0.1 P5.6 F15.1 mm
Orientation	C > T-22.6 > S-2.8
Phase Encoding Dir.	R >> L
Phase Oversampling	54 %
FOV Read	168 mm
FOV Phase	100.0 %
Slice Thickness	2.0 mm
TR	8100.0 ms
TE	83.00 ms
Averages	1
Concatenations	1
AutoAlign	---

Contrast - Common

TR	8100.0 ms
TE	83.00 ms
MTC	Off
Magn. Preparation	None
Flip Angle Mode	Hyperecho
Flip Angle	119 deg
Fat-Water Contrast	Standard
Dark Blood	Off
Contrasts	1
Wrap-up Magn.	Restore
Reconstruction	Magnitude

Contrast - Dynamic

Dynamic Mode	Standard
Measurements	1
Multiple Series	Each Measurement

Resolution - Common

FOV Read	168 mm
FOV Phase	100.0 %
Slice Thickness	2.0 mm
Base Resolution	384
Phase Resolution	100 %
Interpolation	Off

Resolution - Acceleration

Acceleration Mode	None
Deep Resolve	Off
Phase Partial Fourier	Off

Resolution - Filter

Raw Filter	Off
Elliptical Filter	Off
Distortion Correction	2D
Normalize	Prescan
Image Filter	On

Geometry - Common

Slice Group	1
Slices	36
Distance Factor	0 %
Position	R0.1 P5.6 F15.1 mm
Orientation	C > T-22.6 > S-2.8
Phase Encoding Dir.	R >> L
Phase Oversampling	54 %
FOV Read	168 mm
FOV Phase	100.0 %
Slice Thickness	2.0 mm
TR	8100.0 ms
Multi-Slice Mode	Interleaved
Series	Interleaved
Concatenations	1

Geometry - AutoAlign

Slice Group	1
Position	R0.1 P5.6 F15.1 mm
Orientation	C > T-22.6 > S-2.8
Phase Encoding Dir.	R >> L
AutoAlign	---
Initial Position	R0.1 P5.6 F15.1
R	0.1 mm
P	5.6 mm
F	15.1 mm
Initial Orientation	C > T
C > T	-22.60
> S	-2.80
Initial Rotation	0.00 deg

Geometry - Navigator

Geometry - Saturation

Saturation Region	1
Thickness	75.00 mm
Position	L0.2 A44.6 F130.3 mm
Orientation	T > C-2.4 > S-0.1
Shape	Standard
Special Saturation	None

Geometry - Tim Planning Suite

Set-n-Go Protocol	Off
Table Position	0 mm
Table Position	H
Inline Composing	Off

System - Miscellaneous

Coil Selection	ACS All but spine
Radial Sorting	Off
MSMA	S - C - T
Sagittal	L >> R
Coronal	A >> P
Transversal	F >> H
Coil Combination	Adaptive Combine
Matrix Optimization	Off
Coil Focus	Flat

System - Adjustments

Adjustment Strategy	Standard
B0 Shim	Standard
B1 Shim	TrueForm
Adjustment Tolerance	Auto
Adjust with Body Coil	Off
Confirm Frequency	Never
Assume Silicone	Off

System - Adjust Volume

Position	R0.1 P5.6 F15.1 mm
Orientation	C > T-22.6 > S-2.8
Rotation	0.00 deg
R >> L	168 mm
F >> H	168 mm
A >> P	72 mm
Reset	Off

System - pTx

B1 Shim	TrueForm
LR Balancing	Off

System - Tx/Rx

Frequency 1H	123.237845 MHz
? Ref. Amplitude 1H	0.000 V
Reset	Off
Image Scaling	1.000

Physio - Signal

1st Signal/Mode	None
TR	8100.0 ms
Concatenations	1

Physio - Cardiac

Fat-Water Contrast	Standard
Magn. Preparation	None
Dark Blood	Off
FOV Read	168 mm
FOV Phase	100.0 %
Phase Resolution	100 %
Motion Correction	None

Physio - PACE

Resp. Control	Off
Concatenations	1

Inline - Subtraction

Subtract	Off
Measurements	1
StdDev	Off
Motion Correction	None
Save Original Images	On

Inline - MIP

MIP Sag	Off
MIP Cor	Off
MIP Tra	Off
MIP Time	Off
Radial MIP	Off
Save Original Images	On
MPR Sag	Off
MPR Cor	Off
MPR Tra	Off

Inline - Composing

Inline Composing	Off

Sequence - Part 1

Sequence Name	tseR_rr
Dimension	2D
RF Pulse Type	Fast
Gradient Mode	Fast
Flow Compensation	Read
Bandwidth	181 Hz/Px
Echo Spacing	11.8 ms
Free Echo Spacing	Off
Define	Turbo Factor
Turbo Factor	17
Echo Trains per Slice	35

Sequence - Part 2

Introduction	On
Phase Correction	Automatic

Sequence - Part 2

Compensate T2 Decay	Off
Fast Mode	Off
WARP	Off
Red. EC Sensitivity	Off
Acoustic noise reduction	Off
Reduce Motion Sens.	On
Motion Correction	None

Sequence - Assistant

SAR Assistant	Off
Allowed Delay	60 s

\\USER\2025_MBC_PROTOCOLS_XA60\SB.2020.353 AEP study - 64 Channel\SB.2020.353 - AEP MRI ACQUISITION PROTOCOL v22- 64 channel\T2map_CorOblHipp_RL-

TA: 3:17 min Coil Selection: Auto Voxel Size: 0.9×0.9×3.6 mm³ Acc:: 2 Rel. SNR: 1.00

Properties

Start measurement without further preparation	On
Wait for User to Start	Off
Start measurements	Single Measurement
Prio Recon	Off
Auto Open Inline Display	Off
Auto Close Inline Display	Off
Load Images to MR View&GO	On
Auto Store Images	On
Disable auto transfer to PACS	Off
Load Images to Stamp Segments	Off
Load Images to Graphic Segments	Off
Graphic segment	Default
Inline Movie	Off

Routine

Slice Group	1
Slices	44
Distance Factor	10 %
Position	L0.2 P8.0 F10.3 mm
Orientation	C > T-22.6 > S-2.8
Phase Encoding Dir.	R >> L
Phase Oversampling	0 %
FOV Read	230 mm
FOV Phase	93.8 %
Slice Thickness	3.6 mm
TR	3910.0 ms
TE 1	10.70 ms
TE 2	21.40 ms
TE 3	32.10 ms
TE 4	42.80 ms
TE 5	53.50 ms
TE 6	64.20 ms
TE 7	74.90 ms
TE 8	85.60 ms
TE 9	96.30 ms
TE 10	107.00 ms
TE 11	117.70 ms
TE 12	128.40 ms
TE 13	139.10 ms
TE 14	149.80 ms
TE 15	160.50 ms
TE 16	171.20 ms
Averages	1
Concatenations	2
AutoAlign	---

Contrast - Common

TR	3910.0 ms
TE 1	10.70 ms
TE 2	21.40 ms
TE 3	32.10 ms
TE 4	42.80 ms
TE 5	53.50 ms
TE 6	64.20 ms
TE 7	74.90 ms
TE 8	85.60 ms
TE 9	96.30 ms
TE 10	107.00 ms
TE 11	117.70 ms
TE 12	128.40 ms
TE 13	139.10 ms
TE 14	149.80 ms
TE 15	160.50 ms
TE 16	171.20 ms
TD	0.00 ms
MTC	Off
Magn. Preparation	None
Flip Angle	180 deg
Fat-Water Contrast	Standard
Dark Blood	Off
Contrasts	16
Reconstruction	Magnitude

Contrast - Dynamic

Dynamic Mode	Standard
Measurements	1
Multiple Series	Each Measurement

Resolution - Common

FOV Read	230 mm
FOV Phase	93.8 %
Slice Thickness	3.6 mm
Base Resolution	256
Phase Resolution	100 %

Resolution - Acceleration

Acceleration Mode	GRAPPA
Reference Scans	Integrated
Acceleration Factor PE	2

Resolution - Filter

Raw Filter	Off
Elliptical Filter	Off
Distortion Correction	2D
Normalize	Prescan
Image Filter	On

Geometry - Common

Slice Group	1
Slices	44
Distance Factor	10 %
Position	L0.2 P8.0 F10.3 mm
Orientation	C > T-22.6 > S-2.8
Phase Encoding Dir.	R >> L
Phase Oversampling	0 %
FOV Read	230 mm
FOV Phase	93.8 %
Slice Thickness	3.6 mm
TR	3910.0 ms
Multi-Slice Mode	Interleaved
Series	Interleaved
Concatenations	2

Geometry - AutoAlign

Slice Group	1
Position	L0.2 P8.0 F10.3 mm
Orientation	C > T-22.6 > S-2.8
Phase Encoding Dir.	R >> L
AutoAlign	---
Initial Position	L0.2 P8.0 F10.3
L	0.2 mm
P	8.0 mm
F	10.3 mm
Initial Orientation	C > T
C > T	-22.60
> S	-2.80
Initial Rotation	0.00 deg

Geometry - Navigator

Geometry - Saturation

Special Saturation	None

Geometry - Tim Planning Suite

Set-n-Go Protocol	Off
Table Position	0 mm
Table Position	H
Inline Composing	Off

System - Miscellaneous

Coil Selection	Auto Coil Select
Radial Sorting	Off
MSMA	S - C - T
Sagittal	L >> R
Coronal	A >> P
Transversal	F >> H
Matrix Optimization	Performance
Coil Focus	Flat

System - Adjustments

Adjustment Strategy	Standard
B0 Shim	Standard

System - Adjustments

B1 Shim	TrueForm
Adjustment Tolerance	Auto
Adjust with Body Coil	Off
Confirm Frequency	Never
Assume Silicone	Off

System - Adjust Volume

Position	L0.2 P8.0 F10.3 mm
Orientation	C > T-22.6 > S-2.8
Rotation	0.00 deg
R >> L	216 mm
F >> H	230 mm
A >> P	174 mm
Reset	Off

System - pTx

B1 Shim	TrueForm

System - Tx/Rx

Frequency 1H	123.237845 MHz
? Ref. Amplitude 1H	0.000 V
Reset	Off
Image Scaling	1.000

Physio - Signal

1st Signal/Mode	None
TR	3910.0 ms
Concatenations	2

Physio - Cardiac

Fat-Water Contrast	Standard
Magn. Preparation	None
Dark Blood	Off
FOV Read	230 mm
FOV Phase	93.8 %
Phase Resolution	100 %

Physio - PACE

Resp. Control	Off
Concatenations	2

Inline - Liver

Liver Registration	Off
Save Original Images	On

Inline - Subtraction

Subtract	Off
Measurements	1
StdDev	Off
Save Original Images	On

Inline - MIP

MIP Sag	Off
MIP Cor	Off

Inline - MIP

MIP Tra	Off
MIP Time	Off
Radial MIP	Off
Save Original Images	On
MPR Sag	Off
MPR Cor	Off
MPR Tra	Off

Inline - Composing

Inline Composing	Off

Inline - MapIt

MapIt	None
Flip Angle	180 deg
Measurements	1
Contrasts	16
TE 1	10.70 ms
TE 2	21.40 ms
TE 3	32.10 ms
TE 4	42.80 ms
TE 5	53.50 ms
TE 6	64.20 ms
TE 7	74.90 ms
TE 8	85.60 ms
TE 9	96.30 ms
TE 10	107.00 ms
TE 11	117.70 ms
TE 12	128.40 ms
TE 13	139.10 ms
TE 14	149.80 ms
TE 15	160.50 ms
TE 16	171.20 ms
TR	3910.0 ms
Save Original Images	On

Sequence - Part 1

Sequence Name	WIP_se
RF Pulse Type	Low SAR
Gradient Mode	Fast
Bandwidth	219 Hz/Px

Sequence - Part 2

Introduction	On

Sequence - Special

Undersampling Factor	5
R2 Images	On
M0 Images	On
Interpolate	On
Filter Background	On
Regularization:	5.00
Virtual Channels	4
Simulated TE 0	83 ms
Simulated TE 1	0 ms

Sequence - Special

Simulated TE 2	0 ms
Simulated TE 3	0 ms
Simulated TE 4	0 ms

Sequence - Assistant

SAR Assistant	Off
Allowed Delay	0 s

\\USER\2025_MBC_PROTOCOLS_XA60\SB.2020.353 AEP study - 64 Channel\SB.2020.353 - AEP MRI ACQUISITION PROTOCOL v22- 64 channel\T2w_AxOblTempPole_RL-

TA: 5:38 min Coil Selection: Auto Voxel Size: 0.3×0.3×0.5 mm³ Acc:: 2 Rel. SNR: 1.00

Properties

Start measurement without further preparation	On
Wait for User to Start	Off
Start measurements	Single Measurement
Prio Recon	Off
Auto Open Inline Display	Off
Auto Close Inline Display	Off
Load Images to MR View&GO	On
Auto Store Images	On
Disable auto transfer to PACS	Off
Load Images to Stamp Segments	Off
Load Images to Graphic Segments	Off
Graphic segment	Default
Inline Movie	Off

Routine

Slab Group	1
Slabs	1
Position	Isocenter
Orientation	T > C-20.0
Phase Encoding Dir.	R >> L
Slices per Slab	72
Phase Oversampling	20 %
Slice Oversampling	22.2 %
FOV Read	160 mm
FOV Phase	100.0 %
Slice Thickness	0.50 mm
TR	1400.0 ms
TE	156.00 ms
Averages	2.0
Concatenations	1
AutoAlign	---

Contrast - Common

TR	1400.0 ms
TE	156.00 ms
MTC	Off
Magn. Preparation	None
Flip Angle Mode	Constant
Flip Angle	120 deg
Fat-Water Contrast	Standard
Dark Blood	Off
Blood Suppression	Off
Wrap-up Magn.	Restore
Reconstruction	Magnitude

Contrast - Dynamic

Dynamic Mode	Standard
Measurements	1

Contrast - Dynamic

Multiple Series	Each Measurement
Reordering	Linear

Resolution - Common

FOV Read	160 mm
FOV Phase	100.0 %
Slice Thickness	0.50 mm
Base Resolution	320
Phase Resolution	100 %
Slice Resolution	84 %
Interpolation	On

Resolution - Acceleration

Acceleration Mode	GRAPPA
Total Factor	2
Reference Scans	Integrated
Acceleration Factor PE	2
Reference Lines PE	24
Acceleration Factor 3D	1
Phase Partial Fourier	Allowed
Slice Partial Fourier	6/8
Elliptical Scanning	Off

Resolution - Filter

Raw Filter	On
Elliptical Filter	Off
Distortion Correction	2D
Normalize	Prescan
Image Filter	Off

Geometry - Common

Slab Group	1
Slabs	1
Position	Isocenter
Orientation	T > C-20.0
Phase Encoding Dir.	R >> L
Slices per Slab	72
Phase Oversampling	20 %
Slice Oversampling	22.2 %
FOV Read	160 mm
FOV Phase	100.0 %
Slice Thickness	0.50 mm
TR	1400.0 ms
Concatenations	1

Geometry - AutoAlign

Slab Group	1
Position	Isocenter
Orientation	T > C-20.0

Geometry - AutoAlign

Phase Encoding Dir.	R >> L
AutoAlign	---
Initial Position	Isocenter
L	0.0 mm
P	0.0 mm
H	0.0 mm
Initial Orientation	T > C
T > C	-20.00
> S	0.00
Initial Rotation	90.00 deg

Geometry - Navigator

Geometry - Saturation

Special Saturation	None

Geometry - Tim Planning Suite

Set-n-Go Protocol	Off
Table Position	0 mm
Table Position	H
Inline Composing	Off

System - Miscellaneous

Coil Selection	ACS All but spine
Radial Sorting	Off
MSMA	S - C - T
Sagittal	R >> L
Coronal	A >> P
Transversal	F >> H
Coil Combination	Adaptive Combine
Matrix Optimization	Off
Coil Focus	Flat

System - Adjustments

Adjustment Strategy	Standard
B0 Shim	Standard
B1 Shim	TrueForm
Adjustment Tolerance	Auto
Adjust with Body Coil	Off
Confirm Frequency	Never
Assume Silicone	Off

System - Adjust Volume

Position	Isocenter
Orientation	T > C-20.0
Rotation	90.00 deg
R >> L	160 mm
A >> P	160 mm
F >> H	36 mm
Reset	Off

System - pTx

B1 Shim	TrueForm
Excitation	Slab-sel.

System - Tx/Rx

Frequency 1H	123.237845 MHz
? Ref. Amplitude 1H	0.000 V
Reset	Off
Image Scaling	1.000
Gain	High

Physio - Signal

1st Signal/Mode	None
Trigger Delay	0 ms
TR	1400.0 ms
Concatenations	1

Physio - Cardiac

Fat-Water Contrast	Standard
Magn. Preparation	None
Dark Blood	Off
FOV Read	160 mm
FOV Phase	100.0 %
Phase Resolution	100 %

Physio - PACE

Resp. Control	Off
Concatenations	1

Inline - Subtraction

Subtract	Off
Measurements	1
StdDev	Off
Save Original Images	On

Inline - MIP

MIP Sag	Off
MIP Cor	Off
MIP Tra	Off
MIP Time	Off
Radial MIP	Off
Save Original Images	On
MPR Sag	Off
MPR Cor	Off
MPR Tra	Off

Inline - Composing

Inline Composing	Off

Sequence - Part 1

Sequence Name	spcR
Dimension	3D
Excitation	Slab-sel.
RF Pulse Type	Normal
Gradient Mode	Normal
Flow Compensation	None
Reordering	Linear
Bandwidth	289 Hz/Px
Echo Spacing	6.88 ms

Sequence - Part 1

Turbo Factor	96
Echo Train Duration	482 ms

Sequence - Part 2

Introduction	On

Sequence - Assistant

SAR Assistant	Off
Allowed Delay	0 s

\\USER\2025_MBC_PROTOCOLS_XA60\SB.2020.353 AEP study - 64 Channel\SB.2020.353 - AEP MRI ACQUISITION PROTOCOL v22- 64 channel\DWI_Ax_AP_52Dir-

TA: 2:58 min Coil Selection: Auto Voxel Size: 1.8×1.8×1.8 mm^3 Acc:: None Rel. SNR: 1.00

Properties

Start measurement without further preparation	On
Wait for User to Start	On
Start measurements	Single Measurement
Prio Recon	Off
Auto Open Inline Display	Off
Auto Close Inline Display	Off
Load Images to MR View&GO	On
Auto Store Images	On
Disable auto transfer to PACS	Off
Load Images to Stamp Segments	Off
Load Images to Graphic Segments	Off
Graphic segment	Default
Inline Movie	Off

Routine

Slice Group	1
Slices	84
Distance Factor	0 %
Position	Isocenter
Orientation	Transversal
Phase Encoding Dir.	A >> P
Phase Oversampling	0 %
FOV Read	230 mm
FOV Phase	100.0 %
Slice Thickness	1.8 mm
TR	3065.0 ms
TE	83.00 ms
Multi-band accel. factor	4
AutoAlign	---

Contrast - Common

TR	3065.0 ms
TE	83.00 ms
MTC	Off
Magn. Preparation	None
Flip Angle	90 deg
Refocus flip angle	180 deg
Fat-Water Contrast	Standard
Grad. rev. fat suppr.	Enabled
Reconstruction	Magn./Phase

Contrast - Dynamic

Dynamic Mode	Standard
Multiple Series	Off
Delay in TR	0.00 ms

Resolution - Common

FOV Read	230 mm
FOV Phase	100.0 %
Slice Thickness	1.8 mm
Base Resolution	128
Phase Resolution	100 %
Interpolation	Off

Resolution - Acceleration

Acceleration Mode	None
Phase Partial Fourier	6/8

Resolution - Filter

Raw Filter	Off
Elliptical Filter	Off
Distortion Correction	Off
Static Field Correction	Off
Normalize	Prescan
Noise Masking	Off

Geometry - Common

Slice Group	1
Slices	84
Distance Factor	0 %
Position	Isocenter
Orientation	Transversal
Phase Encoding Dir.	A >> P
Phase Oversampling	0 %
FOV Read	230 mm
FOV Phase	100.0 %
Slice Thickness	1.8 mm
TR	3065.0 ms
Multi-Slice Mode	Interleaved
Series	Interleaved
Multi-band accel. factor	4

Geometry - AutoAlign

Slice Group	1
Position	Isocenter
Orientation	Transversal
Phase Encoding Dir.	A >> P
AutoAlign	---
Initial Position	Isocenter
L	0.0 mm
P	0.0 mm
H	0.0 mm
Initial Orientation	Transversal
Initial Rotation	0.00 deg

Geometry - Navigator

Geometry - Saturation

Special Saturation	None

Geometry - Tim Planning Suite

Set-n-Go Protocol	Off
Table Position	0 mm
Table Position	H
Inline Composing	Off

System - Miscellaneous

Coil Selection	Auto Coil Select
Radial Sorting	Off
MSMA	S - C - T
Sagittal	R >> L
Coronal	A >> P
Transversal	F >> H
Coil Combination	Sum of Squares
Matrix Optimization	Off
Coil Focus	Flat

System - Adjustments

Adjustment Strategy	Standard
B0 Shim	Standard
B1 Shim	TrueForm
Adjustment Tolerance	Auto
Adjust with Body Coil	Off
Confirm Frequency	Never
Assume Silicone	Off

System - Adjust Volume

Position	Isocenter
Orientation	Transversal
Rotation	0.00 deg
A >> P	230 mm
R >> L	230 mm
F >> H	152 mm
Reset	Off

System - pTx

B1 Shim	TrueForm
Excitation	Standard

System - Tx/Rx

Frequency 1H	123.237845 MHz
? Ref. Amplitude 1H	0.000 V
Reset	Off
Image Scaling	1.000

Physio - Signal

1st Signal/Mode	None
TR	3065.0 ms
Multi-band accel. factor	4

Physio - PACE

Resp. Control	Off

Physio - PACE

Multi-band accel. factor	4

Diff

Diffusion Mode	Free
Diff. Directions	52
Diffusion Scheme	Monopolar
Diff. Weightings	2
b-value 1	0 s/mm²
b-value 2	3000 s/mm²
Averages 1	1
Averages 2	1
Dynamic Field Correction	Off
Invert Gray Scale	Off
Diff. Weighted Images	On
Trace Weighted Images	Off
Tensor	Off
FA Maps	Off
ADC Maps	Off
Exponential ADC Maps	Off
Noise Masking	Off
Calculated Image	Off

Sequence - Part 1

Sequence Name	epse
Dimension	2D
Excitation	Standard
Gradient Mode	Performance
Bandwidth	2056 Hz/Px
Echo Spacing	0.59 ms
Free Echo Spacing	Off
EPI Factor	128

Sequence - Part 2

Introduction	Off
RF Spoiling	Off
Phase Correction	Internal

Sequence - Special

Excite pulse duration	5120 us
Refocus pulse duration	10240 us
Min. prep scans	0
Min. prep scans SB	0
Delay before PC scans	0 us
Single-band images	On
MB LeakBlock kernel	On
MB dual kernel	Off
MB RF phase scramble	Off
Opt. MB RF pulse BW	Off
Time-shifted MB RF	Off
SENSE1 coil combine	On
Invert RO/PE polarity	Off
Force Maxwell corr.	Off
PF omits higher k-space	Off
Force GPA balance	Off

Sequence - Special

Disable B1 control loop	Off
Disable freq. update	Off
Suppress 16-bit DICOM	Off
Force equal slice timing	Off
Online multi-band recon.	Online
FFT scale factor	1.00
Physio recording	DICOM

Sequence - Assistant

SAR Assistant	Off

\\USER\2025_MBC_PROTOCOLS_XA60\SB.2020.353 AEP study - 64 Channel\SB.2020.353 - AEP MRI ACQUISITION PROTOCOL v22- 64 channel\DWI_Ax_AP_51Dir_InvROPE-

TA: 2:55 min Coil Selection: Auto Voxel Size: 1.8×1.8×1.8 mm^3 Acc:: None Rel. SNR: 1.00

Properties

Start measurement without further preparation	On
Wait for User to Start	Off
Start measurements	Single Measurement
Prio Recon	Off
Auto Open Inline Display	Off
Auto Close Inline Display	Off
Load Images to MR View&GO	On
Auto Store Images	On
Disable auto transfer to PACS	Off
Load Images to Stamp Segments	Off
Load Images to Graphic Segments	Off
Graphic segment	Default
Inline Movie	Off

Routine

Slice Group	1
Slices	84
Distance Factor	0 %
Position	Isocenter
Orientation	Transversal
Phase Encoding Dir.	A >> P
Phase Oversampling	0 %
FOV Read	230 mm
FOV Phase	100.0 %
Slice Thickness	1.8 mm
TR	3065.0 ms
TE	83.00 ms
Multi-band accel. factor	4
AutoAlign	---

Contrast - Common

TR	3065.0 ms
TE	83.00 ms
MTC	Off
Magn. Preparation	None
Flip Angle	90 deg
Refocus flip angle	180 deg
Fat-Water Contrast	Standard
Grad. rev. fat suppr.	Enabled
Reconstruction	Magn./Phase

Contrast - Dynamic

Dynamic Mode	Standard
Multiple Series	Off
Delay in TR	0.00 ms

Resolution - Common

FOV Read	230 mm
FOV Phase	100.0 %
Slice Thickness	1.8 mm
Base Resolution	128
Phase Resolution	100 %
Interpolation	Off

Resolution - Acceleration

Acceleration Mode	None
Phase Partial Fourier	6/8

Resolution - Filter

Raw Filter	Off
Elliptical Filter	Off
Distortion Correction	Off
Static Field Correction	Off
Normalize	Prescan
Noise Masking	Off

Geometry - Common

Slice Group	1
Slices	84
Distance Factor	0 %
Position	Isocenter
Orientation	Transversal
Phase Encoding Dir.	A >> P
Phase Oversampling	0 %
FOV Read	230 mm
FOV Phase	100.0 %
Slice Thickness	1.8 mm
TR	3065.0 ms
Multi-Slice Mode	Interleaved
Series	Interleaved
Multi-band accel. factor	4

Geometry - AutoAlign

Slice Group	1
Position	Isocenter
Orientation	Transversal
Phase Encoding Dir.	A >> P
AutoAlign	---
Initial Position	Isocenter
L	0.0 mm
P	0.0 mm
H	0.0 mm
Initial Orientation	Transversal
Initial Rotation	0.00 deg

Geometry - Navigator

Geometry - Saturation

Special Saturation	None

Geometry - Tim Planning Suite

Set-n-Go Protocol	Off
Table Position	0 mm
Table Position	H
Inline Composing	Off

System - Miscellaneous

Coil Selection	Auto Coil Select
Radial Sorting	Off
MSMA	S - C - T
Sagittal	R >> L
Coronal	A >> P
Transversal	F >> H
Coil Combination	Sum of Squares
Matrix Optimization	Off
Coil Focus	Flat

System - Adjustments

Adjustment Strategy	Standard
B0 Shim	Standard
B1 Shim	TrueForm
Adjustment Tolerance	Auto
Adjust with Body Coil	Off
Confirm Frequency	Never
Assume Silicone	Off

System - Adjust Volume

Position	Isocenter
Orientation	Transversal
Rotation	0.00 deg
A >> P	230 mm
R >> L	230 mm
F >> H	152 mm
Reset	Off

System - pTx

B1 Shim	TrueForm
Excitation	Standard

System - Tx/Rx

Frequency 1H	123.237845 MHz
? Ref. Amplitude 1H	0.000 V
Reset	Off
Image Scaling	1.000

Physio - Signal

1st Signal/Mode	None
TR	3065.0 ms
Multi-band accel. factor	4

Physio - PACE

Resp. Control	Off

Physio - PACE

Multi-band accel. factor	4

Diff

Diffusion Mode	Free
Diff. Directions	51
Diffusion Scheme	Monopolar
Diff. Weightings	2
b-value 1	0 s/mm²
b-value 2	3000 s/mm²
Averages 1	1
Averages 2	1
Dynamic Field Correction	Off
Invert Gray Scale	Off
Diff. Weighted Images	On
Trace Weighted Images	Off
Tensor	Off
FA Maps	Off
ADC Maps	Off
Exponential ADC Maps	Off
Noise Masking	Off
Calculated Image	Off

Sequence - Part 1

Sequence Name	epse
Dimension	2D
Excitation	Standard
Gradient Mode	Performance
Bandwidth	2056 Hz/Px
Echo Spacing	0.59 ms
Free Echo Spacing	Off
EPI Factor	128

Sequence - Part 2

Introduction	Off
RF Spoiling	Off
Phase Correction	Internal

Sequence - Special

Excite pulse duration	5120 us
Refocus pulse duration	10240 us
Min. prep scans	0
Min. prep scans SB	0
Delay before PC scans	0 us
Single-band images	On
MB LeakBlock kernel	On
MB dual kernel	Off
MB RF phase scramble	Off
Opt. MB RF pulse BW	Off
Time-shifted MB RF	Off
SENSE1 coil combine	On
Invert RO/PE polarity	On
Force Maxwell corr.	Off
PF omits higher k-space	Off
Force GPA balance	Off

Sequence - Special

Disable B1 control loop	Off
Disable freq. update	Off
Suppress 16-bit DICOM	Off
Force equal slice timing	Off
Online multi-band recon.	Online
FFT scale factor	1.00
Physio recording	DICOM

Sequence - Assistant

SAR Assistant	Off

\\USER\2025_MBC_PROTOCOLS_XA60\SB.2020.353 AEP study - 64 Channel\SB.2020.353 - AEP MRI ACQUISITION PROTOCOL v22- 64 channel\T1map_MP2RAGE_Sag_AP- as imported *

TA: 3:42 min Coil Selection: Auto Voxel Size: 1.0×1.0×1.0 mm³ Acc:: None Rel. SNR: 1.00

Properties

Start measurement without further preparation	On
Wait for User to Start	Off
Start measurements	Single Measurement
Prio Recon	Off
Auto Open Inline Display	Off
Auto Close Inline Display	Off
Load Images to MR View&GO	On
Auto Store Images	On
Disable auto transfer to PACS	Off
Load Images to Stamp Segments	Off
Load Images to Graphic Segments	Off
Graphic segment	Default
Inline Movie	Off

Routine

Slab Group	1
Slabs	1
Distance Factor	50 %
Position	Isocenter
Orientation	Sagittal
Phase Encoding Dir.	A >> P
Slices per Slab	208
Phase Oversampling	0 %
Slice Oversampling	0.0 %
FOV Read	256 mm
FOV Phase	93.8 %
Slice Thickness	1.00 mm
TR	5000.0 ms
TE	2.88 ms
Averages	1
Concatenations	1
AutoAlign	---

Contrast - Common

TR	5000.0 ms
TE	2.88 ms
Magn. Preparation	Non-sel. IR
TI 1	700 ms
TI 2	2500 ms
Flip Angle 1	4 deg
Flip Angle 2	5 deg
Fat-Water Contrast	Standard
Dark Blood	Off
Contrasts	1
Reconstruction	Magnitude

Contrast - Dynamic

Dynamic Mode	Standard

Contrast - Dynamic

Measurements	1
Multiple Series	Each Measurement
Reordering	Linear

Resolution - Common

FOV Read	256 mm
FOV Phase	93.8 %
Slice Thickness	1.00 mm
Base Resolution	256
Phase Resolution	100 %
Slice Resolution	100 %
Interpolation	Off

Resolution - Acceleration

Acceleration Mode	None
Reference Scans	GRE/Separate
Reference Lines PE	32
Phase Partial Fourier	Off
Slice Partial Fourier	Off
Asymmetric Echo	Off
Elliptical Scanning	Off

Resolution - Filter

Raw Filter	Off
Elliptical Filter	Off
Distortion Correction	2D
Normalize	Prescan
Image Filter	Off

Geometry - Common

Slab Group	1
Slabs	1
Distance Factor	50 %
Position	Isocenter
Orientation	Sagittal
Phase Encoding Dir.	A >> P
Slices per Slab	208
Phase Oversampling	0 %
Slice Oversampling	0.0 %
FOV Read	256 mm
FOV Phase	93.8 %
Slice Thickness	1.00 mm
TR	5000.0 ms
Multi-Slice Mode	Single Shot
Series	Interleaved
Concatenations	1

Geometry - AutoAlign

Slab Group	1

Geometry - AutoAlign

Position	Isocenter
Orientation	Sagittal
Phase Encoding Dir.	A >> P
AutoAlign	---
Initial Position	Isocenter
L	0.0 mm
P	0.0 mm
H	0.0 mm
Initial Orientation	Sagittal
Initial Rotation	0.00 deg

Geometry - Navigator

Geometry - Tim Planning Suite

Set-n-Go Protocol	Off
Table Position	0 mm
Table Position	H
Inline Composing	Off

System - Miscellaneous

Coil Selection	Auto Coil Select
Radial Sorting	Off
MSMA	S - C - T
Sagittal	R >> L
Coronal	A >> P
Transversal	F >> H
Coil Combination	Adaptive Combine
Matrix Optimization	Performance
Coil Focus	Flat

System - Adjustments

Adjustment Strategy	Standard
B0 Shim	Standard
B1 Shim	TrueForm
Adjustment Tolerance	Auto
Adjust with Body Coil	Off
Confirm Frequency	Never
Assume Silicone	Off

System - Adjust Volume

! Position	Isocenter
! Orientation	Sagittal
! Rotation	0.00 deg
! A >> P	240 mm
! F >> H	208 mm
! R >> L	256 mm
Reset	Off

System - pTx

B1 Shim	TrueForm
Excitation	Non-sel.

System - Tx/Rx

Frequency 1H	123.237845 MHz

System - Tx/Rx

? Ref. Amplitude 1H	0.000 V
Reset	Off
Image Scaling	1.000

Physio - Signal

1st Signal/Mode	None
TR	5000.0 ms
Concatenations	1

Physio - Cardiac

Fat-Water Contrast	Standard
Magn. Preparation	Non-sel. IR
TI 1	700 ms
TI 2	2500 ms
Dark Blood	Off
FOV Read	256 mm
FOV Phase	93.8 %
Phase Resolution	100 %

Physio - PACE

Resp. Control	Off
Concatenations	1

Inline - Subtraction

Subtract	Off
Measurements	1
StdDev	Off
Save Original Images	On

Inline - MIP

MIP Sag	Off
MIP Cor	Off
MIP Tra	Off
MIP Time	Off
Radial MIP	Off
Save Original Images	On
MPR Sag	Off
MPR Cor	Off
MPR Tra	Off

Inline - Composing

Inline Composing	Off

Inline - MapIt

MapIt	T1 Map
Flip Angle 1	4 deg
Flip Angle 2	5 deg
Measurements	1
Contrasts	1
TE	2.88 ms
TR	5000.0 ms
Save Original Images	On

Sequence - Part 1

Sequence Name	WIP_tfl
Dimension	3D
Excitation	Non-sel.
RF Pulse Type	Fast
Gradient Mode	Fast
Flow Compensation	None
Reordering	Linear
Bandwidth	240 Hz/Px
Echo Spacing	6.82 ms
Asymmetric Echo	Off
Turbo Factor	190

Sequence - Part 2

Introduction	Off
RF Spoiling	On
Incr. Gradient Spoiling	Off

Sequence - Special

Sparse Undersampling	On
Sampling Pattern	Cartesian Phyllotaxis
Undersampling Factor	4.6 x
Density	0.50
Jitter Radius	1.2
Diff. Mask per INV	Off
Regularisation INV1	0.00100
Regularisation INV2	0.00040
No. Iterations	20
CSM RO Resolution	0
DL Recon	Default
Synthetic TI1	0 ms
Synthetic TI2	0 ms
Uniform Img.	On
Denoised UNI	On
FLAWS-min	Off
FLAWS-hc	Off
FLAWS-hco	Off
Div. Image	Off
UNIDenoised Weight	1.0

Sequence - Assistant

SAR Assistant	Off

\\USER\2025_MBC_PROTOCOLS_XA60\SB.2020.353 AEP study - 64 Channel\SB.2020.353 - AEP MRI ACQUISITION PROTOCOL v22- 64 channel\fMRI_AxObl_AP_Pseudoword-

TA: 3:20 min Coil Selection: Auto Voxel Size: 3.0×3.0×3.0 mm³ Acc:: 2 Rel. SNR: 1.00

Properties

Start measurement without further preparation	On
Wait for User to Start	On
Start measurements	Single Measurement
Prio Recon	Off
Auto Open Inline Display	Off
Auto Close Inline Display	Off
Load Images to MR View&GO	On
Auto Store Images	On
Disable auto transfer to PACS	Off
Load Images to Stamp Segments	Off
Load Images to Graphic Segments	Off
Graphic segment	Default
Inline Movie	Off

Routine

Slice Group	1
Slices	44
Distance Factor	0 %
Position	Isocenter
Orientation	T > C-30.0
Phase Encoding Dir.	A >> P
Phase Oversampling	0 %
FOV Read	216 mm
FOV Phase	111.1 %
Slice Thickness	3.0 mm
TR	900.0 ms
TE 1	13.40 ms
TE 2	32.96 ms
TE 3	52.52 ms
Averages	1
Multi-band accel. factor	4
AutoAlign	---

Contrast - Common

TR	900.0 ms
TE 1	13.40 ms
TE 2	32.96 ms
TE 3	52.52 ms
MTC	Off
Magn. Preparation	None
Flip Angle	30 deg
Fat-Water Contrast	Fat Saturation
Contrasts	3
Reconstruction	Magn./Phase

Contrast - Dynamic

Dynamic Mode	Standard
Measurements	202

Contrast - Dynamic

Delay in TR	0.00 ms

Resolution - Common

FOV Read	216 mm
FOV Phase	111.1 %
Slice Thickness	3.0 mm
Base Resolution	72
Phase Resolution	100 %
Interpolation	Off

Resolution - Acceleration

Acceleration Mode	GRAPPA
Reference scan mode	Single-shot
Acceleration Factor PE	2
Reference Lines PE	24
Phase Partial Fourier	7/8

Resolution - Filter

Raw Filter	Off
Elliptical Filter	Off
Hamming	Off
Distortion Correction	Off
Static Field Correction	Off
Normalize	Prescan

Geometry - Common

Slice Group	1
Slices	44
Distance Factor	0 %
Position	Isocenter
Orientation	T > C-30.0
Phase Encoding Dir.	A >> P
Phase Oversampling	0 %
FOV Read	216 mm
FOV Phase	111.1 %
Slice Thickness	3.0 mm
TR	900.0 ms
Multi-Slice Mode	Interleaved
Series	Interleaved
Multi-band accel. factor	4

Geometry - AutoAlign

Slice Group	1
Position	Isocenter
Orientation	T > C-30.0
Phase Encoding Dir.	A >> P
AutoAlign	---
Initial Position	Isocenter
L	0.0 mm

Geometry - AutoAlign

P	0.0 mm
H	0.0 mm
Initial Orientation	T > C
T > C	-30.00
> S	0.00
Initial Rotation	0.00 deg

Geometry - Saturation

Special Saturation	None

Geometry - Tim Planning Suite

Set-n-Go Protocol	Off
Table Position	0 mm
Table Position	H
Inline Composing	Off

System - Miscellaneous

Coil Selection	Auto Coil Select
Radial Sorting	Off
MSMA	S - C - T
Sagittal	R >> L
Coronal	A >> P
Transversal	F >> H
Coil Combination	Sum of Squares
Matrix Optimization	Off
Coil Focus	Flat

System - Adjustments

Adjustment Strategy	Standard
B0 Shim	Standard
B1 Shim	TrueForm
Adjustment Tolerance	Auto
Adjust with Body Coil	Off
Confirm Frequency	Never
Assume Silicone	Off

System - Adjust Volume

Position	Isocenter
Orientation	T > C-30.0
Rotation	90.00 deg
R >> L	216 mm
A >> P	240 mm
F >> H	132 mm
Reset	Off

System - pTx

B1 Shim	TrueForm
Excitation	Standard

System - Tx/Rx

Frequency 1H	123.237845 MHz
? Ref. Amplitude 1H	0.000 V
Reset	Off
Image Scaling	1.000

Physio - Signal

1st Signal/Mode	None
TR	900.0 ms
Log Signals	Off
Multi-band accel. factor	4

BOLD

GLM Statistics	Off
Ignore Meas. at Start	0
Ignore After Transition	0
Model Transition States	Off
Temp. Highpass Filter	Off
Threshold	4.00
Paradigm Size	201
Meas[1]	Baseline
Meas[2]	Baseline
Meas[3]	Baseline
Meas[4]	Baseline
Meas[5]	Baseline
Meas[6]	Baseline
Meas[7]	Baseline
Meas[8]	Baseline
Meas[9]	Baseline
Meas[10]	Baseline
Meas[11]	Active
Meas[12]	Active
Meas[13]	Active
Meas[14]	Active
Meas[15]	Active
Meas[16]	Active
Meas[17]	Active
Meas[18]	Active
Meas[19]	Active
Meas[20]	Active
Meas[21]	Active
Meas[22]	Active
Meas[23]	Active
Meas[24]	Active
Meas[25]	Active
Meas[26]	Active
Meas[27]	Active
Meas[28]	Active
Meas[29]	Active
Meas[30]	Active
Meas[31]	Baseline
Meas[32]	Baseline
Meas[33]	Baseline
Meas[34]	Baseline
Meas[35]	Baseline
Meas[36]	Baseline
Meas[37]	Baseline
Meas[38]	Baseline
Meas[39]	Baseline
Meas[40]	Baseline
Meas[41]	Baseline

BOLD

Meas[42]	Baseline
Meas[43]	Baseline
Meas[44]	Baseline
Meas[45]	Baseline
Meas[46]	Baseline
Meas[47]	Baseline
Meas[48]	Baseline
Meas[49]	Baseline
Meas[50]	Baseline
Meas[51]	Active
Meas[52]	Active
Meas[53]	Active
Meas[54]	Active
Meas[55]	Active
Meas[56]	Active
Meas[57]	Active
Meas[58]	Active
Meas[59]	Active
Meas[60]	Active
Meas[61]	Active
Meas[62]	Active
Meas[63]	Active
Meas[64]	Active
Meas[65]	Active
Meas[66]	Active
Meas[67]	Active
Meas[68]	Active
Meas[69]	Active
Meas[70]	Active
Meas[71]	Baseline
Meas[72]	Baseline
Meas[73]	Baseline
Meas[74]	Baseline
Meas[75]	Baseline
Meas[76]	Baseline
Meas[77]	Baseline
Meas[78]	Baseline
Meas[79]	Baseline
Meas[80]	Baseline
Meas[81]	Baseline
Meas[82]	Baseline
Meas[83]	Baseline
Meas[84]	Baseline
Meas[85]	Baseline
Meas[86]	Baseline
Meas[87]	Baseline
Meas[88]	Baseline
Meas[89]	Baseline
Meas[90]	Baseline
Meas[91]	Active
Meas[92]	Active
Meas[93]	Active
Meas[94]	Active
Meas[95]	Active

BOLD

Meas[96]	Active
Meas[97]	Active
Meas[98]	Active
Meas[99]	Active
Meas[100]	Active
Meas[101]	Active
Meas[102]	Active
Meas[103]	Active
Meas[104]	Active
Meas[105]	Active
Meas[106]	Active
Meas[107]	Active
Meas[108]	Active
Meas[109]	Active
Meas[110]	Active
Meas[111]	Baseline
Meas[112]	Baseline
Meas[113]	Baseline
Meas[114]	Baseline
Meas[115]	Baseline
Meas[116]	Baseline
Meas[117]	Baseline
Meas[118]	Baseline
Meas[119]	Baseline
Meas[120]	Baseline
Meas[121]	Baseline
Meas[122]	Baseline
Meas[123]	Baseline
Meas[124]	Baseline
Meas[125]	Baseline
Meas[126]	Baseline
Meas[127]	Baseline
Meas[128]	Baseline
Meas[129]	Baseline
Meas[130]	Baseline
Meas[131]	Active
Meas[132]	Active
Meas[133]	Active
Meas[134]	Active
Meas[135]	Active
Meas[136]	Active
Meas[137]	Active
Meas[138]	Active
Meas[139]	Active
Meas[140]	Active
Meas[141]	Active
Meas[142]	Active
Meas[143]	Active
Meas[144]	Active
Meas[145]	Active
Meas[146]	Active
Meas[147]	Active
Meas[148]	Active
Meas[149]	Active

BOLD

Meas[150]	Active
Meas[151]	Baseline
Meas[152]	Baseline
Meas[153]	Baseline
Meas[154]	Baseline
Meas[155]	Baseline
Meas[156]	Baseline
Meas[157]	Baseline
Meas[158]	Baseline
Meas[159]	Baseline
Meas[160]	Baseline
Meas[161]	Baseline
Meas[162]	Baseline
Meas[163]	Baseline
Meas[164]	Baseline
Meas[165]	Baseline
Meas[166]	Baseline
Meas[167]	Baseline
Meas[168]	Baseline
Meas[169]	Baseline
Meas[170]	Baseline
Meas[171]	Active
Meas[172]	Active
Meas[173]	Active
Meas[174]	Active
Meas[175]	Active
Meas[176]	Active
Meas[177]	Active
Meas[178]	Active
Meas[179]	Active
Meas[180]	Active
Meas[181]	Active
Meas[182]	Active
Meas[183]	Active
Meas[184]	Active
Meas[185]	Active
Meas[186]	Active
Meas[187]	Active
Meas[188]	Active
Meas[189]	Active
Meas[190]	Active
Meas[191]	Baseline
Meas[192]	Baseline
Meas[193]	Baseline
Meas[194]	Baseline
Meas[195]	Baseline
Meas[196]	Baseline
Meas[197]	Baseline
Meas[198]	Baseline
Meas[199]	Baseline
Meas[200]	Baseline
Meas[201]	Baseline
Motion Correction	Off
Spatial Filter	Off

BOLD

Measurements	202
Delay in TR	0.00 ms

Sequence - Part 1

Sequence Name	epfid
Dimension	2D
Excitation	Standard
Gradient Mode	Performance
Flow Compensation	None
Bandwidth	2314 Hz/Px
Echo Spacing	0.54 ms
Free Echo Spacing	Off
EPI Factor	80

Sequence - Part 2

Introduction	Off
RF Spoiling	Off

Sequence - Special

Excite pulse duration	5120 us
Min. prep scans	0
Min. prep scans SB	0
Inter-TE delay	0 us
Delay before PC scans	0 us
Single-band images	On
MB LeakBlock kernel	On
MB dual kernel	Off
MB RF phase scramble	Off
Opt. MB RF pulse BW	Off
SENSE1 coil combine	On
Invert RO/PE polarity	Off
Force Maxwell corr.	Off
PF omits higher k-space	Off
Disable B1 control loop	Off
Disable freq. update	Off
Suppress 16-bit DICOM	Off
Force equal slice timing	Off
Online multi-band recon.	Online
FFT scale factor	1.00
Fat saturation FA	110.00 deg
Fat sat. offset	0.00 Hz
Sinc exc. pulse BWTP	5.20
Physio recording	DICOM
Triggering scheme	Standard

Sequence - Assistant

SAR Assistant	Off

\\USER\2025_MBC_PROTOCOLS_XA60\SB.2020.353 AEP study - 64 Channel\SB.2020.353 - AEP MRI ACQUISITION PROTOCOL v22- 64 channel\FMap_AxObl_AP-

TA: 10 sec Coil Selection: Auto Voxel Size: 2.0×2.0×2.0 mm³ Acc:: None Rel. SNR: 1.00

Properties

Start measurement without further preparation	On
Wait for User to Start	Off
Start measurements	Single Measurement
Prio Recon	Off
Auto Open Inline Display	Off
Auto Close Inline Display	Off
Load Images to MR View&GO	On
Auto Store Images	On
Disable auto transfer to PACS	Off
Load Images to Stamp Segments	Off
Load Images to Graphic Segments	Off
Graphic segment	Default
Inline Movie	Off

Routine

Slice Group	1
Slices	72
Distance Factor	0 %
Position	Isocenter
Orientation	T > C-30.0
Phase Encoding Dir.	A >> P
Phase Oversampling	0 %
FOV Read	216 mm
FOV Phase	111.1 %
Slice Thickness	2.0 mm
TR	10100.0 ms
TE	84.00 ms
Averages	1
Multi-band accel. factor	1
AutoAlign	---

Contrast - Common

TR	10100.0 ms
TE	84.00 ms
MTC	Off
Magn. Preparation	None
Flip Angle	90 deg
Refocus flip angle	180 deg
Fat-Water Contrast	Fat Saturation
Grad. rev. fat suppr.	Disabled
Contrasts	1
Reconstruction	Magnitude

Contrast - Dynamic

Dynamic Mode	Standard
Measurements	1
Delay in TR	0.00 ms

Resolution - Common

FOV Read	216 mm
FOV Phase	111.1 %
Slice Thickness	2.0 mm
Base Resolution	108
Phase Resolution	100 %
Interpolation	Off

Resolution - Acceleration

Acceleration Mode	None
Phase Partial Fourier	Off

Resolution - Filter

Raw Filter	Off
Elliptical Filter	Off
Hamming	Off
Distortion Correction	Off
Static Field Correction	Off
Normalize	Prescan

Geometry - Common

Slice Group	1
Slices	72
Distance Factor	0 %
Position	Isocenter
Orientation	T > C-30.0
Phase Encoding Dir.	A >> P
Phase Oversampling	0 %
FOV Read	216 mm
FOV Phase	111.1 %
Slice Thickness	2.0 mm
TR	10100.0 ms
Multi-Slice Mode	Interleaved
Series	Interleaved
Multi-band accel. factor	1

Geometry - AutoAlign

Slice Group	1
Position	Isocenter
Orientation	T > C-30.0
Phase Encoding Dir.	A >> P
AutoAlign	---
Initial Position	Isocenter
L	0.0 mm
P	0.0 mm
H	0.0 mm
Initial Orientation	T > C
T > C	-30.00
> S	0.00
Initial Rotation	0.00 deg

Geometry - Saturation

Special Saturation	None

Geometry - Tim Planning Suite

Set-n-Go Protocol	Off
Table Position	0 mm
Table Position	H
Inline Composing	Off

System - Miscellaneous

Coil Selection	Auto Coil Select
Radial Sorting	Off
MSMA	S - C - T
Sagittal	R >> L
Coronal	A >> P
Transversal	F >> H
Coil Combination	Sum of Squares
Matrix Optimization	Off
Coil Focus	Flat

System - Adjustments

Adjustment Strategy	Standard
B0 Shim	Standard
B1 Shim	TrueForm
Adjustment Tolerance	Auto
Adjust with Body Coil	Off
Confirm Frequency	Never
Assume Silicone	Off

System - Adjust Volume

Position	Isocenter
Orientation	T > C-30.0
Rotation	90.00 deg
R >> L	216 mm
A >> P	240 mm
F >> H	144 mm
Reset	Off

System - pTx

B1 Shim	TrueForm
Excitation	Standard

System - Tx/Rx

Frequency 1H	123.237845 MHz
? Ref. Amplitude 1H	0.000 V
Reset	Off
Image Scaling	1.000

Physio - Signal

1st Signal/Mode	None
TR	10100.0 ms
Log Signals	Off
Multi-band accel. factor	1

BOLD

GLM Statistics	Off
Ignore Meas. at Start	0
Ignore After Transition	0
Model Transition States	On
Temp. Highpass Filter	On
Threshold	4.00
Paradigm Size	4
Meas[1]	Baseline
Meas[2]	Baseline
Meas[3]	Baseline
Meas[4]	Active
Motion Correction	Off
Spatial Filter	Off
Measurements	1
Delay in TR	0.00 ms

Sequence - Part 1

Sequence Name	epse
Dimension	2D
Excitation	Standard
RF Pulse Type	Normal
Gradient Mode	Performance
Bandwidth	2104 Hz/Px
Echo Spacing	0.61 ms
Free Echo Spacing	On
EPI Factor	120

Sequence - Part 2

Introduction	Off

Sequence - Special

Min. prep scans	0
Delay before PC scans	0 us
SENSE1 coil combine	On
Invert RO/PE polarity	Off
Force GPA balance	Off
Disable B1 control loop	Off
Disable freq. update	Off
Suppress 16-bit DICOM	Off
Force equal slice timing	Off
FFT scale factor	1.00
Fat saturation FA	110.00 deg
Fat sat. offset	0.00 Hz
Physio recording	DICOM
Triggering scheme	Standard

Sequence - Assistant

SAR Assistant	Off

\\USER\2025_MBC_PROTOCOLS_XA60\SB.2020.353 AEP study - 64 Channel\SB.2020.353 - AEP MRI ACQUISITION PROTOCOL v22- 64 channel\FMap_AxObl_AP_InvROPE-

TA: 10 sec Coil Selection: Auto Voxel Size: 2.0×2.0×2.0 mm³ Acc:: None Rel. SNR: 1.00

Properties

Start measurement without further preparation	On
Wait for User to Start	Off
Start measurements	Single Measurement
Prio Recon	Off
Auto Open Inline Display	Off
Auto Close Inline Display	Off
Load Images to MR View&GO	On
Auto Store Images	On
Disable auto transfer to PACS	Off
Load Images to Stamp Segments	Off
Load Images to Graphic Segments	Off
Graphic segment	Default
Inline Movie	Off

Routine

Slice Group	1
Slices	72
Distance Factor	0 %
Position	Isocenter
Orientation	T > C-30.0
Phase Encoding Dir.	A >> P
Phase Oversampling	0 %
FOV Read	216 mm
FOV Phase	111.1 %
Slice Thickness	2.0 mm
TR	10100.0 ms
TE	84.00 ms
Averages	1
Multi-band accel. factor	1
AutoAlign	---

Contrast - Common

TR	10100.0 ms
TE	84.00 ms
MTC	Off
Magn. Preparation	None
Flip Angle	90 deg
Refocus flip angle	180 deg
Fat-Water Contrast	Fat Saturation
Grad. rev. fat suppr.	Disabled
Contrasts	1
Reconstruction	Magnitude

Contrast - Dynamic

Dynamic Mode	Standard
Measurements	1
Delay in TR	0.00 ms

Resolution - Common

FOV Read	216 mm
FOV Phase	111.1 %
Slice Thickness	2.0 mm
Base Resolution	108
Phase Resolution	100 %
Interpolation	Off

Resolution - Acceleration

Acceleration Mode	None
Phase Partial Fourier	Off

Resolution - Filter

Raw Filter	Off
Elliptical Filter	Off
Hamming	Off
Distortion Correction	Off
Static Field Correction	Off
Normalize	Prescan

Geometry - Common

Slice Group	1
Slices	72
Distance Factor	0 %
Position	Isocenter
Orientation	T > C-30.0
Phase Encoding Dir.	A >> P
Phase Oversampling	0 %
FOV Read	216 mm
FOV Phase	111.1 %
Slice Thickness	2.0 mm
TR	10100.0 ms
Multi-Slice Mode	Interleaved
Series	Interleaved
Multi-band accel. factor	1

Geometry - AutoAlign

Slice Group	1
Position	Isocenter
Orientation	T > C-30.0
Phase Encoding Dir.	A >> P
AutoAlign	---
Initial Position	Isocenter
L	0.0 mm
P	0.0 mm
H	0.0 mm
Initial Orientation	T > C
T > C	-30.00
> S	0.00
Initial Rotation	0.00 deg

Geometry - Saturation

Special Saturation	None

Geometry - Tim Planning Suite

Set-n-Go Protocol	Off
Table Position	0 mm
Table Position	H
Inline Composing	Off

System - Miscellaneous

Coil Selection	ACS All but spine
Radial Sorting	Off
MSMA	S - C - T
Sagittal	R >> L
Coronal	A >> P
Transversal	F >> H
Coil Combination	Sum of Squares
Matrix Optimization	Off
Coil Focus	Flat

System - Adjustments

Adjustment Strategy	Standard
B0 Shim	Standard
B1 Shim	TrueForm
Adjustment Tolerance	Auto
Adjust with Body Coil	Off
Confirm Frequency	Never
Assume Silicone	Off

System - Adjust Volume

Position	Isocenter
Orientation	T > C-30.0
Rotation	90.00 deg
R >> L	216 mm
A >> P	240 mm
F >> H	144 mm
Reset	Off

System - pTx

B1 Shim	TrueForm
Excitation	Standard

System - Tx/Rx

Frequency 1H	123.237845 MHz
? Ref. Amplitude 1H	0.000 V
Reset	Off
Image Scaling	1.000

Physio - Signal

1st Signal/Mode	None
TR	10100.0 ms
Log Signals	Off
Multi-band accel. factor	1

BOLD

GLM Statistics	Off
Ignore Meas. at Start	0
Ignore After Transition	0
Model Transition States	On
Temp. Highpass Filter	On
Threshold	4.00
Paradigm Size	4
Meas[1]	Baseline
Meas[2]	Baseline
Meas[3]	Baseline
Meas[4]	Active
Motion Correction	Off
Spatial Filter	Off
Measurements	1
Delay in TR	0.00 ms

Sequence - Part 1

Sequence Name	epse
Dimension	2D
Excitation	Standard
RF Pulse Type	Normal
Gradient Mode	Performance
Bandwidth	2104 Hz/Px
Echo Spacing	0.61 ms
Free Echo Spacing	On
EPI Factor	120

Sequence - Part 2

Introduction	Off

Sequence - Special

Min. prep scans	0
Delay before PC scans	0 us
SENSE1 coil combine	On
Invert RO/PE polarity	On
Force GPA balance	Off
Disable B1 control loop	Off
Disable freq. update	Off
Suppress 16-bit DICOM	Off
Force equal slice timing	Off
FFT scale factor	1.00
Fat saturation FA	110.00 deg
Fat sat. offset	0.00 Hz
Physio recording	DICOM
Triggering scheme	Standard

Sequence - Assistant

SAR Assistant	Off

\\USER\2025_MBC_PROTOCOLS_XA60\SB.2020.353 AEP study - 64 Channel\SB.2020.353 - AEP MRI ACQUISITION PROTOCOL v22- 64 channel\fMRI_AxObl_AP_OneBack-

TA: 4:53 min Coil Selection: Auto Voxel Size: 3.0×3.0×3.0 mm³ Acc:: 2 Rel. SNR: 1.00

Properties

Start measurement without further preparation	On
Wait for User to Start	On
Start measurements	Single Measurement
Prio Recon	Off
Auto Open Inline Display	Off
Auto Close Inline Display	Off
Load Images to MR View&GO	On
Auto Store Images	On
Disable auto transfer to PACS	Off
Load Images to Stamp Segments	Off
Load Images to Graphic Segments	Off
Graphic segment	Default
Inline Movie	Off

Routine

Slice Group	1
Slices	44
Distance Factor	0 %
Position	Isocenter
Orientation	T > C-30.0
Phase Encoding Dir.	A >> P
Phase Oversampling	0 %
FOV Read	216 mm
FOV Phase	111.1 %
Slice Thickness	3.0 mm
TR	900.0 ms
TE 1	13.40 ms
TE 2	32.96 ms
TE 3	52.52 ms
Averages	1
Multi-band accel. factor	4
AutoAlign	---

Contrast - Common

TR	900.0 ms
TE 1	13.40 ms
TE 2	32.96 ms
TE 3	52.52 ms
MTC	Off
Magn. Preparation	None
Flip Angle	30 deg
Fat-Water Contrast	Fat Saturation
Contrasts	3
Reconstruction	Magn./Phase

Contrast - Dynamic

Dynamic Mode	Standard
Measurements	305

Contrast - Dynamic

Delay in TR	0.00 ms

Resolution - Common

FOV Read	216 mm
FOV Phase	111.1 %
Slice Thickness	3.0 mm
Base Resolution	72
Phase Resolution	100 %
Interpolation	Off

Resolution - Acceleration

Acceleration Mode	GRAPPA
Reference scan mode	Single-shot
Acceleration Factor PE	2
Reference Lines PE	24
Phase Partial Fourier	7/8

Resolution - Filter

Raw Filter	Off
Elliptical Filter	Off
Hamming	Off
Distortion Correction	Off
Static Field Correction	Off
Normalize	Prescan

Geometry - Common

Slice Group	1
Slices	44
Distance Factor	0 %
Position	Isocenter
Orientation	T > C-30.0
Phase Encoding Dir.	A >> P
Phase Oversampling	0 %
FOV Read	216 mm
FOV Phase	111.1 %
Slice Thickness	3.0 mm
TR	900.0 ms
Multi-Slice Mode	Interleaved
Series	Interleaved
Multi-band accel. factor	4

Geometry - AutoAlign

Slice Group	1
Position	Isocenter
Orientation	T > C-30.0
Phase Encoding Dir.	A >> P
AutoAlign	---
Initial Position	Isocenter
L	0.0 mm

Geometry - AutoAlign

P	0.0 mm
H	0.0 mm
Initial Orientation	T > C
T > C	-30.00
> S	0.00
Initial Rotation	0.00 deg

Geometry - Saturation

Special Saturation	None

Geometry - Tim Planning Suite

Set-n-Go Protocol	Off
Table Position	0 mm
Table Position	H
Inline Composing	Off

System - Miscellaneous

Coil Selection	Auto Coil Select
Radial Sorting	Off
MSMA	S - C - T
Sagittal	R >> L
Coronal	A >> P
Transversal	F >> H
Coil Combination	Sum of Squares
Matrix Optimization	Off
Coil Focus	Flat

System - Adjustments

Adjustment Strategy	Standard
B0 Shim	Standard
B1 Shim	TrueForm
Adjustment Tolerance	Auto
Adjust with Body Coil	Off
Confirm Frequency	Never
Assume Silicone	Off

System - Adjust Volume

Position	Isocenter
Orientation	T > C-30.0
Rotation	90.00 deg
R >> L	216 mm
A >> P	240 mm
F >> H	132 mm
Reset	Off

System - pTx

B1 Shim	TrueForm
Excitation	Standard

System - Tx/Rx

Frequency 1H	123.237845 MHz
? Ref. Amplitude 1H	0.000 V
Reset	Off
Image Scaling	1.000

Physio - Signal

1st Signal/Mode	None
TR	900.0 ms
Log Signals	Off
Multi-band accel. factor	4

BOLD

GLM Statistics	Off
Ignore Meas. at Start	0
Ignore After Transition	0
Model Transition States	Off
Temp. Highpass Filter	Off
Threshold	4.00
Paradigm Size	305
Meas[1]	Baseline
Meas[2]	Baseline
Meas[3]	Baseline
Meas[4]	Baseline
Meas[5]	Baseline
Meas[6]	Baseline
Meas[7]	Baseline
Meas[8]	Baseline
Meas[9]	Baseline
Meas[10]	Baseline
Meas[11]	Baseline
Meas[12]	Baseline
Meas[13]	Baseline
Meas[14]	Baseline
Meas[15]	Baseline
Meas[16]	Baseline
Meas[17]	Baseline
Meas[18]	Active
Meas[19]	Active
Meas[20]	Active
Meas[21]	Active
Meas[22]	Active
Meas[23]	Active
Meas[24]	Active
Meas[25]	Active
Meas[26]	Active
Meas[27]	Active
Meas[28]	Active
Meas[29]	Active
Meas[30]	Active
Meas[31]	Active
Meas[32]	Active
Meas[33]	Active
Meas[34]	Active
Meas[35]	Active
Meas[36]	Active
Meas[37]	Active
Meas[38]	Active
Meas[39]	Active
Meas[40]	Active
Meas[41]	Active

BOLD

Meas[42]	Active
Meas[43]	Active
Meas[44]	Active
Meas[45]	Active
Meas[46]	Active
Meas[47]	Active
Meas[48]	Active
Meas[49]	Active
Meas[50]	Active
Meas[51]	Active
Meas[52]	Active
Meas[53]	Active
Meas[54]	Active
Meas[55]	Active
Meas[56]	Active
Meas[57]	Active
Meas[58]	Active
Meas[59]	Active
Meas[60]	Active
Meas[61]	Active
Meas[62]	Active
Meas[63]	Active
Meas[64]	Active
Meas[65]	Active
Meas[66]	Active
Meas[67]	Active
Meas[68]	Active
Meas[69]	Active
Meas[70]	Active
Meas[71]	Active
Meas[72]	Active
Meas[73]	Baseline
Meas[74]	Baseline
Meas[75]	Baseline
Meas[76]	Baseline
Meas[77]	Baseline
Meas[78]	Baseline
Meas[79]	Baseline
Meas[80]	Baseline
Meas[81]	Baseline
Meas[82]	Baseline
Meas[83]	Baseline
Meas[84]	Baseline
Meas[85]	Baseline
Meas[86]	Baseline
Meas[87]	Baseline
Meas[88]	Baseline
Meas[89]	Baseline
Meas[90]	Active
Meas[91]	Active
Meas[92]	Active
Meas[93]	Active
Meas[94]	Active
Meas[95]	Active

BOLD

Meas[96]	Active
Meas[97]	Active
Meas[98]	Active
Meas[99]	Active
Meas[100]	Active
Meas[101]	Active
Meas[102]	Active
Meas[103]	Active
Meas[104]	Active
Meas[105]	Active
Meas[106]	Active
Meas[107]	Active
Meas[108]	Active
Meas[109]	Active
Meas[110]	Active
Meas[111]	Active
Meas[112]	Active
Meas[113]	Active
Meas[114]	Active
Meas[115]	Active
Meas[116]	Active
Meas[117]	Active
Meas[118]	Active
Meas[119]	Active
Meas[120]	Active
Meas[121]	Active
Meas[122]	Active
Meas[123]	Active
Meas[124]	Active
Meas[125]	Active
Meas[126]	Active
Meas[127]	Active
Meas[128]	Active
Meas[129]	Active
Meas[130]	Active
Meas[131]	Active
Meas[132]	Active
Meas[133]	Active
Meas[134]	Active
Meas[135]	Active
Meas[136]	Active
Meas[137]	Active
Meas[138]	Active
Meas[139]	Active
Meas[140]	Active
Meas[141]	Active
Meas[142]	Active
Meas[143]	Active
Meas[144]	Active
Meas[145]	Baseline
Meas[146]	Baseline
Meas[147]	Baseline
Meas[148]	Baseline
Meas[149]	Baseline

BOLD

Meas[150]	Baseline
Meas[151]	Baseline
Meas[152]	Baseline
Meas[153]	Baseline
Meas[154]	Baseline
Meas[155]	Baseline
Meas[156]	Baseline
Meas[157]	Baseline
Meas[158]	Baseline
Meas[159]	Baseline
Meas[160]	Baseline
Meas[161]	Baseline
Meas[162]	Active
Meas[163]	Active
Meas[164]	Active
Meas[165]	Active
Meas[166]	Active
Meas[167]	Active
Meas[168]	Active
Meas[169]	Active
Meas[170]	Active
Meas[171]	Active
Meas[172]	Active
Meas[173]	Active
Meas[174]	Active
Meas[175]	Active
Meas[176]	Active
Meas[177]	Active
Meas[178]	Active
Meas[179]	Active
Meas[180]	Active
Meas[181]	Active
Meas[182]	Active
Meas[183]	Active
Meas[184]	Active
Meas[185]	Active
Meas[186]	Active
Meas[187]	Active
Meas[188]	Active
Meas[189]	Active
Meas[190]	Active
Meas[191]	Active
Meas[192]	Active
Meas[193]	Active
Meas[194]	Active
Meas[195]	Active
Meas[196]	Active
Meas[197]	Active
Meas[198]	Active
Meas[199]	Active
Meas[200]	Active
Meas[201]	Active
Meas[202]	Active
Meas[203]	Active

BOLD

Meas[204]	Active
Meas[205]	Active
Meas[206]	Active
Meas[207]	Active
Meas[208]	Active
Meas[209]	Active
Meas[210]	Active
Meas[211]	Active
Meas[212]	Active
Meas[213]	Active
Meas[214]	Active
Meas[215]	Active
Meas[216]	Active
Meas[217]	Baseline
Meas[218]	Baseline
Meas[219]	Baseline
Meas[220]	Baseline
Meas[221]	Baseline
Meas[222]	Baseline
Meas[223]	Baseline
Meas[224]	Baseline
Meas[225]	Baseline
Meas[226]	Baseline
Meas[227]	Baseline
Meas[228]	Baseline
Meas[229]	Baseline
Meas[230]	Baseline
Meas[231]	Baseline
Meas[232]	Baseline
Meas[233]	Baseline
Meas[234]	Active
Meas[235]	Active
Meas[236]	Active
Meas[237]	Active
Meas[238]	Active
Meas[239]	Active
Meas[240]	Active
Meas[241]	Active
Meas[242]	Active
Meas[243]	Active
Meas[244]	Active
Meas[245]	Active
Meas[246]	Active
Meas[247]	Active
Meas[248]	Active
Meas[249]	Active
Meas[250]	Active
Meas[251]	Active
Meas[252]	Active
Meas[253]	Active
Meas[254]	Active
Meas[255]	Active
Meas[256]	Active
Meas[257]	Active

BOLD

Meas[258]	Active
Meas[259]	Active
Meas[260]	Active
Meas[261]	Active
Meas[262]	Active
Meas[263]	Active
Meas[264]	Active
Meas[265]	Active
Meas[266]	Active
Meas[267]	Active
Meas[268]	Active
Meas[269]	Active
Meas[270]	Active
Meas[271]	Active
Meas[272]	Active
Meas[273]	Active
Meas[274]	Active
Meas[275]	Active
Meas[276]	Active
Meas[277]	Active
Meas[278]	Active
Meas[279]	Active
Meas[280]	Active
Meas[281]	Active
Meas[282]	Active
Meas[283]	Active
Meas[284]	Active
Meas[285]	Active
Meas[286]	Active
Meas[287]	Active
Meas[288]	Active
Meas[289]	Baseline
Meas[290]	Baseline
Meas[291]	Baseline
Meas[292]	Baseline
Meas[293]	Baseline
Meas[294]	Baseline
Meas[295]	Baseline
Meas[296]	Baseline
Meas[297]	Baseline
Meas[298]	Baseline
Meas[299]	Baseline
Meas[300]	Baseline
Meas[301]	Baseline
Meas[302]	Baseline
Meas[303]	Baseline
Meas[304]	Baseline
Meas[305]	Baseline
Motion Correction	Off
Spatial Filter	Off
Measurements	305
Delay in TR	0.00 ms

Sequence - Part 1

Sequence Name	epfid
Dimension	2D
Excitation	Standard
Gradient Mode	Performance
Flow Compensation	None
Bandwidth	2314 Hz/Px
Echo Spacing	0.54 ms
Free Echo Spacing	Off
EPI Factor	80

Sequence - Part 2

Introduction	Off
RF Spoiling	Off

Sequence - Special

Excite pulse duration	5120 us
Min. prep scans	0
Min. prep scans SB	0
Inter-TE delay	0 us
Delay before PC scans	0 us
Single-band images	On
MB LeakBlock kernel	On
MB dual kernel	Off
MB RF phase scramble	Off
Opt. MB RF pulse BW	Off
SENSE1 coil combine	On
Invert RO/PE polarity	Off
Force Maxwell corr.	Off
PF omits higher k-space	Off
Disable B1 control loop	Off
Disable freq. update	Off
Suppress 16-bit DICOM	Off
Force equal slice timing	Off
Online multi-band recon.	Online
FFT scale factor	1.00
Fat saturation FA	110.00 deg
Fat sat. offset	0.00 Hz
Sinc exc. pulse BWTP	5.20
Physio recording	DICOM
Triggering scheme	Standard

Sequence - Assistant

SAR Assistant	Off

\\USER\2025_MBC_PROTOCOLS_XA60\SB.2020.353 AEP study - 64 Channel\SB.2020.353 - AEP MRI ACQUISITION PROTOCOL v22- 64 channel\FMap_AxObl_AP-

TA: 10 sec Coil Selection: Auto Voxel Size: 2.0×2.0×2.0 mm³ Acc:: None Rel. SNR: 1.00

Properties

Start measurement without further preparation	On
Wait for User to Start	Off
Start measurements	Single Measurement
Prio Recon	Off
Auto Open Inline Display	Off
Auto Close Inline Display	Off
Load Images to MR View&GO	On
Auto Store Images	On
Disable auto transfer to PACS	Off
Load Images to Stamp Segments	Off
Load Images to Graphic Segments	Off
Graphic segment	Default
Inline Movie	Off

Routine

Slice Group	1
Slices	72
Distance Factor	0 %
Position	Isocenter
Orientation	T > C-30.0
Phase Encoding Dir.	A >> P
Phase Oversampling	0 %
FOV Read	216 mm
FOV Phase	111.1 %
Slice Thickness	2.0 mm
TR	10100.0 ms
TE	84.00 ms
Averages	1
Multi-band accel. factor	1
AutoAlign	---

Contrast - Common

TR	10100.0 ms
TE	84.00 ms
MTC	Off
Magn. Preparation	None
Flip Angle	90 deg
Refocus flip angle	180 deg
Fat-Water Contrast	Fat Saturation
Grad. rev. fat suppr.	Disabled
Contrasts	1
Reconstruction	Magnitude

Contrast - Dynamic

Dynamic Mode	Standard
Measurements	1
Delay in TR	0.00 ms

Resolution - Common

FOV Read	216 mm
FOV Phase	111.1 %
Slice Thickness	2.0 mm
Base Resolution	108
Phase Resolution	100 %
Interpolation	Off

Resolution - Acceleration

Acceleration Mode	None
Phase Partial Fourier	Off

Resolution - Filter

Raw Filter	Off
Elliptical Filter	Off
Hamming	Off
Distortion Correction	Off
Static Field Correction	Off
Normalize	Prescan

Geometry - Common

Slice Group	1
Slices	72
Distance Factor	0 %
Position	Isocenter
Orientation	T > C-30.0
Phase Encoding Dir.	A >> P
Phase Oversampling	0 %
FOV Read	216 mm
FOV Phase	111.1 %
Slice Thickness	2.0 mm
TR	10100.0 ms
Multi-Slice Mode	Interleaved
Series	Interleaved
Multi-band accel. factor	1

Geometry - AutoAlign

Slice Group	1
Position	Isocenter
Orientation	T > C-30.0
Phase Encoding Dir.	A >> P
AutoAlign	---
Initial Position	Isocenter
L	0.0 mm
P	0.0 mm
H	0.0 mm
Initial Orientation	T > C
T > C	-30.00
> S	0.00
Initial Rotation	0.00 deg

Geometry - Saturation

Special Saturation	None

Geometry - Tim Planning Suite

Set-n-Go Protocol	Off
Table Position	0 mm
Table Position	H
Inline Composing	Off

System - Miscellaneous

Coil Selection	Auto Coil Select
Radial Sorting	Off
MSMA	S - C - T
Sagittal	R >> L
Coronal	A >> P
Transversal	F >> H
Coil Combination	Sum of Squares
Matrix Optimization	Off
Coil Focus	Flat

System - Adjustments

Adjustment Strategy	Standard
B0 Shim	Standard
B1 Shim	TrueForm
Adjustment Tolerance	Auto
Adjust with Body Coil	Off
Confirm Frequency	Never
Assume Silicone	Off

System - Adjust Volume

Position	Isocenter
Orientation	T > C-30.0
Rotation	90.00 deg
R >> L	216 mm
A >> P	240 mm
F >> H	144 mm
Reset	Off

System - pTx

B1 Shim	TrueForm
Excitation	Standard

System - Tx/Rx

Frequency 1H	123.237845 MHz
? Ref. Amplitude 1H	0.000 V
Reset	Off
Image Scaling	1.000

Physio - Signal

1st Signal/Mode	None
TR	10100.0 ms
Log Signals	Off
Multi-band accel. factor	1

BOLD

GLM Statistics	Off
Ignore Meas. at Start	0
Ignore After Transition	0
Model Transition States	On
Temp. Highpass Filter	On
Threshold	4.00
Paradigm Size	4
Meas[1]	Baseline
Meas[2]	Baseline
Meas[3]	Baseline
Meas[4]	Active
Motion Correction	Off
Spatial Filter	Off
Measurements	1
Delay in TR	0.00 ms

Sequence - Part 1

Sequence Name	epse
Dimension	2D
Excitation	Standard
RF Pulse Type	Normal
Gradient Mode	Performance
Bandwidth	2104 Hz/Px
Echo Spacing	0.61 ms
Free Echo Spacing	On
EPI Factor	120

Sequence - Part 2

Introduction	Off

Sequence - Special

Min. prep scans	0
Delay before PC scans	0 us
SENSE1 coil combine	On
Invert RO/PE polarity	Off
Force GPA balance	Off
Disable B1 control loop	Off
Disable freq. update	Off
Suppress 16-bit DICOM	Off
Force equal slice timing	Off
FFT scale factor	1.00
Fat saturation FA	110.00 deg
Fat sat. offset	0.00 Hz
Physio recording	DICOM
Triggering scheme	Standard

Sequence - Assistant

SAR Assistant	Off

\\USER\2025_MBC_PROTOCOLS_XA60\SB.2020.353 AEP study - 64 Channel\SB.2020.353 - AEP MRI ACQUISITION PROTOCOL v22- 64 channel\FMap_AxObl_AP_InvROPE-

TA: 10 sec Coil Selection: Auto Voxel Size: 2.0×2.0×2.0 mm³ Acc:: None Rel. SNR: 1.00

Properties

Start measurement without further preparation	On
Wait for User to Start	Off
Start measurements	Single Measurement
Prio Recon	Off
Auto Open Inline Display	Off
Auto Close Inline Display	Off
Load Images to MR View&GO	On
Auto Store Images	On
Disable auto transfer to PACS	Off
Load Images to Stamp Segments	Off
Load Images to Graphic Segments	Off
Graphic segment	Default
Inline Movie	Off

Routine

Slice Group	1
Slices	72
Distance Factor	0 %
Position	Isocenter
Orientation	T > C-30.0
Phase Encoding Dir.	A >> P
Phase Oversampling	0 %
FOV Read	216 mm
FOV Phase	111.1 %
Slice Thickness	2.0 mm
TR	10100.0 ms
TE	84.00 ms
Averages	1
Multi-band accel. factor	1
AutoAlign	---

Contrast - Common

TR	10100.0 ms
TE	84.00 ms
MTC	Off
Magn. Preparation	None
Flip Angle	90 deg
Refocus flip angle	180 deg
Fat-Water Contrast	Fat Saturation
Grad. rev. fat suppr.	Disabled
Contrasts	1
Reconstruction	Magnitude

Contrast - Dynamic

Dynamic Mode	Standard
Measurements	1
Delay in TR	0.00 ms

Resolution - Common

FOV Read	216 mm
FOV Phase	111.1 %
Slice Thickness	2.0 mm
Base Resolution	108
Phase Resolution	100 %
Interpolation	Off

Resolution - Acceleration

Acceleration Mode	None
Phase Partial Fourier	Off

Resolution - Filter

Raw Filter	Off
Elliptical Filter	Off
Hamming	Off
Distortion Correction	Off
Static Field Correction	Off
Normalize	Prescan

Geometry - Common

Slice Group	1
Slices	72
Distance Factor	0 %
Position	Isocenter
Orientation	T > C-30.0
Phase Encoding Dir.	A >> P
Phase Oversampling	0 %
FOV Read	216 mm
FOV Phase	111.1 %
Slice Thickness	2.0 mm
TR	10100.0 ms
Multi-Slice Mode	Interleaved
Series	Interleaved
Multi-band accel. factor	1

Geometry - AutoAlign

Slice Group	1
Position	Isocenter
Orientation	T > C-30.0
Phase Encoding Dir.	A >> P
AutoAlign	---
Initial Position	Isocenter
L	0.0 mm
P	0.0 mm
H	0.0 mm
Initial Orientation	T > C
T > C	-30.00
> S	0.00
Initial Rotation	0.00 deg

Geometry - Saturation

Special Saturation	None

Geometry - Tim Planning Suite

Set-n-Go Protocol	Off
Table Position	0 mm
Table Position	H
Inline Composing	Off

System - Miscellaneous

Coil Selection	ACS All but spine
Radial Sorting	Off
MSMA	S - C - T
Sagittal	R >> L
Coronal	A >> P
Transversal	F >> H
Coil Combination	Sum of Squares
Matrix Optimization	Off
Coil Focus	Flat

System - Adjustments

Adjustment Strategy	Standard
B0 Shim	Standard
B1 Shim	TrueForm
Adjustment Tolerance	Auto
Adjust with Body Coil	Off
Confirm Frequency	Never
Assume Silicone	Off

System - Adjust Volume

Position	Isocenter
Orientation	T > C-30.0
Rotation	90.00 deg
R >> L	216 mm
A >> P	240 mm
F >> H	144 mm
Reset	Off

System - pTx

B1 Shim	TrueForm
Excitation	Standard

System - Tx/Rx

Frequency 1H	123.237845 MHz
? Ref. Amplitude 1H	0.000 V
Reset	Off
Image Scaling	1.000

Physio - Signal

1st Signal/Mode	None
TR	10100.0 ms
Log Signals	Off
Multi-band accel. factor	1

BOLD

GLM Statistics	Off
Ignore Meas. at Start	0
Ignore After Transition	0
Model Transition States	On
Temp. Highpass Filter	On
Threshold	4.00
Paradigm Size	4
Meas[1]	Baseline
Meas[2]	Baseline
Meas[3]	Baseline
Meas[4]	Active
Motion Correction	Off
Spatial Filter	Off
Measurements	1
Delay in TR	0.00 ms

Sequence - Part 1

Sequence Name	epse
Dimension	2D
Excitation	Standard
RF Pulse Type	Normal
Gradient Mode	Performance
Bandwidth	2104 Hz/Px
Echo Spacing	0.61 ms
Free Echo Spacing	On
EPI Factor	120

Sequence - Part 2

Introduction	Off

Sequence - Special

Min. prep scans	0
Delay before PC scans	0 us
SENSE1 coil combine	On
Invert RO/PE polarity	On
Force GPA balance	Off
Disable B1 control loop	Off
Disable freq. update	Off
Suppress 16-bit DICOM	Off
Force equal slice timing	Off
FFT scale factor	1.00
Fat saturation FA	110.00 deg
Fat sat. offset	0.00 Hz
Physio recording	DICOM
Triggering scheme	Standard

Sequence - Assistant

SAR Assistant	Off

\\USER\2025_MBC_PROTOCOLS_XA60\SB.2020.353 AEP study - 64 Channel\SB.2020.353 - AEP MRI ACQUISITION PROTOCOL v22- 64 channel\fMRI_AxObl_AP_Movie-

TA: 13:54 min Coil Selection: Auto Voxel Size: 3.0×3.0×3.0 mm³ Acc:: 2 Rel. SNR: 1.00

Properties

Start measurement without further preparation	On
Wait for User to Start	On
Start measurements	Single Measurement
Prio Recon	Off
Auto Open Inline Display	Off
Auto Close Inline Display	Off
Load Images to MR View&GO	On
Auto Store Images	On
Disable auto transfer to PACS	Off
Load Images to Stamp Segments	Off
Load Images to Graphic Segments	Off
Graphic segment	Default
Inline Movie	Off

Routine

Slice Group	1
Slices	44
Distance Factor	0 %
Position	Isocenter
Orientation	T > C-30.0
Phase Encoding Dir.	A >> P
Phase Oversampling	0 %
FOV Read	216 mm
FOV Phase	111.1 %
Slice Thickness	3.0 mm
TR	900.0 ms
TE 1	13.40 ms
TE 2	32.96 ms
TE 3	52.52 ms
Averages	1
Multi-band accel. factor	4
AutoAlign	---

Contrast - Common

TR	900.0 ms
TE 1	13.40 ms
TE 2	32.96 ms
TE 3	52.52 ms
MTC	Off
Magn. Preparation	None
Flip Angle	30 deg
Fat-Water Contrast	Fat Saturation
Contrasts	3
Reconstruction	Magn./Phase

Contrast - Dynamic

Dynamic Mode	Standard
Measurements	907

Contrast - Dynamic

Delay in TR	0.00 ms

Resolution - Common

FOV Read	216 mm
FOV Phase	111.1 %
Slice Thickness	3.0 mm
Base Resolution	72
Phase Resolution	100 %
Interpolation	Off

Resolution - Acceleration

Acceleration Mode	GRAPPA
Reference scan mode	Single-shot
Acceleration Factor PE	2
Reference Lines PE	24
Phase Partial Fourier	7/8

Resolution - Filter

Raw Filter	Off
Elliptical Filter	Off
Hamming	Off
Distortion Correction	Off
Static Field Correction	Off
Normalize	Prescan

Geometry - Common

Slice Group	1
Slices	44
Distance Factor	0 %
Position	Isocenter
Orientation	T > C-30.0
Phase Encoding Dir.	A >> P
Phase Oversampling	0 %
FOV Read	216 mm
FOV Phase	111.1 %
Slice Thickness	3.0 mm
TR	900.0 ms
Multi-Slice Mode	Interleaved
Series	Interleaved
Multi-band accel. factor	4

Geometry - AutoAlign

Slice Group	1
Position	Isocenter
Orientation	T > C-30.0
Phase Encoding Dir.	A >> P
AutoAlign	---
Initial Position	Isocenter
L	0.0 mm

Geometry - AutoAlign

P	0.0 mm
H	0.0 mm
Initial Orientation	T > C
T > C	-30.00
> S	0.00
Initial Rotation	0.00 deg

Geometry - Saturation

Special Saturation	None

Geometry - Tim Planning Suite

Set-n-Go Protocol	Off
Table Position	0 mm
Table Position	H
Inline Composing	Off

System - Miscellaneous

Coil Selection	Auto Coil Select
Radial Sorting	Off
MSMA	S - C - T
Sagittal	R >> L
Coronal	A >> P
Transversal	F >> H
Coil Combination	Sum of Squares
Matrix Optimization	Off
Coil Focus	Flat

System - Adjustments

Adjustment Strategy	Standard
B0 Shim	Standard
B1 Shim	TrueForm
Adjustment Tolerance	Auto
Adjust with Body Coil	Off
Confirm Frequency	Never
Assume Silicone	Off

System - Adjust Volume

Position	Isocenter
Orientation	T > C-30.0
Rotation	90.00 deg
R >> L	216 mm
A >> P	240 mm
F >> H	132 mm
Reset	Off

System - pTx

B1 Shim	TrueForm
Excitation	Standard

System - Tx/Rx

Frequency 1H	123.237845 MHz
? Ref. Amplitude 1H	0.000 V
Reset	Off
Image Scaling	1.000

Physio - Signal

1st Signal/Mode	None
TR	900.0 ms
Log Signals	Off
Multi-band accel. factor	4

BOLD

GLM Statistics	Off
Ignore Meas. at Start	0
Ignore After Transition	0
Model Transition States	Off
Temp. Highpass Filter	Off
Threshold	4.00
Paradigm Size	80
Meas[1]	Baseline
Meas[2]	Baseline
Meas[3]	Baseline
Meas[4]	Baseline
Meas[5]	Baseline
Meas[6]	Baseline
Meas[7]	Baseline
Meas[8]	Baseline
Meas[9]	Baseline
Meas[10]	Baseline
Meas[11]	Baseline
Meas[12]	Baseline
Meas[13]	Baseline
Meas[14]	Baseline
Meas[15]	Baseline
Meas[16]	Baseline
Meas[17]	Baseline
Meas[18]	Baseline
Meas[19]	Baseline
Meas[20]	Baseline
Meas[21]	Baseline
Meas[22]	Baseline
Meas[23]	Baseline
Meas[24]	Baseline
Meas[25]	Baseline
Meas[26]	Baseline
Meas[27]	Baseline
Meas[28]	Baseline
Meas[29]	Baseline
Meas[30]	Baseline
Meas[31]	Baseline
Meas[32]	Baseline
Meas[33]	Baseline
Meas[34]	Baseline
Meas[35]	Baseline
Meas[36]	Baseline
Meas[37]	Baseline
Meas[38]	Baseline
Meas[39]	Baseline
Meas[40]	Baseline
Meas[41]	Active

BOLD

Meas[42]	Active
Meas[43]	Active
Meas[44]	Active
Meas[45]	Active
Meas[46]	Active
Meas[47]	Active
Meas[48]	Active
Meas[49]	Active
Meas[50]	Active
Meas[51]	Active
Meas[52]	Active
Meas[53]	Active
Meas[54]	Active
Meas[55]	Active
Meas[56]	Active
Meas[57]	Active
Meas[58]	Active
Meas[59]	Active
Meas[60]	Active
Meas[61]	Active
Meas[62]	Active
Meas[63]	Active
Meas[64]	Active
Meas[65]	Active
Meas[66]	Active
Meas[67]	Active
Meas[68]	Active
Meas[69]	Active
Meas[70]	Active
Meas[71]	Active
Meas[72]	Active
Meas[73]	Active
Meas[74]	Active
Meas[75]	Active
Meas[76]	Active
Meas[77]	Active
Meas[78]	Active
Meas[79]	Active
Meas[80]	Active
Motion Correction	Off
Spatial Filter	Off
Measurements	907
Delay in TR	0.00 ms

Sequence - Part 1

Sequence Name	epfid
Dimension	2D
Excitation	Standard
Gradient Mode	Performance
Flow Compensation	None
Bandwidth	2314 Hz/Px
Echo Spacing	0.54 ms
Free Echo Spacing	Off
EPI Factor	80

Sequence - Part 2

Introduction	Off
RF Spoiling	Off

Sequence - Special

Excite pulse duration	5120 us
Min. prep scans	0
Min. prep scans SB	0
Inter-TE delay	0 us
Delay before PC scans	0 us
Single-band images	On
MB LeakBlock kernel	On
MB dual kernel	Off
MB RF phase scramble	Off
Opt. MB RF pulse BW	Off
SENSE1 coil combine	On
Invert RO/PE polarity	Off
Force Maxwell corr.	Off
PF omits higher k-space	Off
Disable B1 control loop	Off
Disable freq. update	Off
Suppress 16-bit DICOM	Off
Force equal slice timing	Off
Online multi-band recon.	Online
FFT scale factor	1.00
Fat saturation FA	110.00 deg
Fat sat. offset	0.00 Hz
Sinc exc. pulse BWTP	5.20
Physio recording	DICOM
Triggering scheme	Standard

Sequence - Assistant

SAR Assistant	Off

\\USER\2025_MBC_PROTOCOLS_XA60\SB.2020.353 AEP study - 64 Channel\SB.2020.353 - AEP MRI ACQUISITION PROTOCOL v22- 64 channel\FMap_AxObl_AP-

TA: 10 sec Coil Selection: Auto Voxel Size: 2.0×2.0×2.0 mm^3 Acc:: None Rel. SNR: 1.00

Properties

Start measurement without further preparation	On
Wait for User to Start	Off
Start measurements	Single Measurement
Prio Recon	Off
Auto Open Inline Display	Off
Auto Close Inline Display	Off
Load Images to MR View&GO	On
Auto Store Images	On
Disable auto transfer to PACS	Off
Load Images to Stamp Segments	Off
Load Images to Graphic Segments	Off
Graphic segment	Default
Inline Movie	Off

Routine

Slice Group	1
Slices	72
Distance Factor	0 %
Position	Isocenter
Orientation	T > C-30.0
Phase Encoding Dir.	A >> P
Phase Oversampling	0 %
FOV Read	216 mm
FOV Phase	111.1 %
Slice Thickness	2.0 mm
TR	10100.0 ms
TE	84.00 ms
Averages	1
Multi-band accel. factor	1
AutoAlign	---

Contrast - Common

TR	10100.0 ms
TE	84.00 ms
MTC	Off
Magn. Preparation	None
Flip Angle	90 deg
Refocus flip angle	180 deg
Fat-Water Contrast	Fat Saturation
Grad. rev. fat suppr.	Disabled
Contrasts	1
Reconstruction	Magnitude

Contrast - Dynamic

Dynamic Mode	Standard
Measurements	1
Delay in TR	0.00 ms

Resolution - Common

FOV Read	216 mm
FOV Phase	111.1 %
Slice Thickness	2.0 mm
Base Resolution	108
Phase Resolution	100 %
Interpolation	Off

Resolution - Acceleration

Acceleration Mode	None
Phase Partial Fourier	Off

Resolution - Filter

Raw Filter	Off
Elliptical Filter	Off
Hamming	Off
Distortion Correction	Off
Static Field Correction	Off
Normalize	Prescan

Geometry - Common

Slice Group	1
Slices	72
Distance Factor	0 %
Position	Isocenter
Orientation	T > C-30.0
Phase Encoding Dir.	A >> P
Phase Oversampling	0 %
FOV Read	216 mm
FOV Phase	111.1 %
Slice Thickness	2.0 mm
TR	10100.0 ms
Multi-Slice Mode	Interleaved
Series	Interleaved
Multi-band accel. factor	1

Geometry - AutoAlign

Slice Group	1
Position	Isocenter
Orientation	T > C-30.0
Phase Encoding Dir.	A >> P
AutoAlign	---
Initial Position	Isocenter
L	0.0 mm
P	0.0 mm
H	0.0 mm
Initial Orientation	T > C
T > C	-30.00
> S	0.00
Initial Rotation	0.00 deg

Geometry - Saturation

Special Saturation	None

Geometry - Tim Planning Suite

Set-n-Go Protocol	Off
Table Position	0 mm
Table Position	H
Inline Composing	Off

System - Miscellaneous

Coil Selection	Auto Coil Select
Radial Sorting	Off
MSMA	S - C - T
Sagittal	R >> L
Coronal	A >> P
Transversal	F >> H
Coil Combination	Sum of Squares
Matrix Optimization	Off
Coil Focus	Flat

System - Adjustments

Adjustment Strategy	Standard
B0 Shim	Standard
B1 Shim	TrueForm
Adjustment Tolerance	Auto
Adjust with Body Coil	Off
Confirm Frequency	Never
Assume Silicone	Off

System - Adjust Volume

Position	Isocenter
Orientation	T > C-30.0
Rotation	90.00 deg
R >> L	216 mm
A >> P	240 mm
F >> H	144 mm
Reset	Off

System - pTx

B1 Shim	TrueForm
Excitation	Standard

System - Tx/Rx

Frequency 1H	123.237845 MHz
? Ref. Amplitude 1H	0.000 V
Reset	Off
Image Scaling	1.000

Physio - Signal

1st Signal/Mode	None
TR	10100.0 ms
Log Signals	Off
Multi-band accel. factor	1

BOLD

GLM Statistics	Off
Ignore Meas. at Start	0
Ignore After Transition	0
Model Transition States	On
Temp. Highpass Filter	On
Threshold	4.00
Paradigm Size	4
Meas[1]	Baseline
Meas[2]	Baseline
Meas[3]	Baseline
Meas[4]	Active
Motion Correction	Off
Spatial Filter	Off
Measurements	1
Delay in TR	0.00 ms

Sequence - Part 1

Sequence Name	epse
Dimension	2D
Excitation	Standard
RF Pulse Type	Normal
Gradient Mode	Performance
Bandwidth	2104 Hz/Px
Echo Spacing	0.61 ms
Free Echo Spacing	On
EPI Factor	120

Sequence - Part 2

Introduction	Off

Sequence - Special

Min. prep scans	0
Delay before PC scans	0 us
SENSE1 coil combine	On
Invert RO/PE polarity	Off
Force GPA balance	Off
Disable B1 control loop	Off
Disable freq. update	Off
Suppress 16-bit DICOM	Off
Force equal slice timing	Off
FFT scale factor	1.00
Fat saturation FA	110.00 deg
Fat sat. offset	0.00 Hz
Physio recording	DICOM
Triggering scheme	Standard

Sequence - Assistant

SAR Assistant	Off

\\USER\2025_MBC_PROTOCOLS_XA60\SB.2020.353 AEP study - 64 Channel\SB.2020.353 - AEP MRI ACQUISITION PROTOCOL v22- 64 channel\FMap_AxObl_AP_InvROPE-

TA: 10 sec Coil Selection: Auto Voxel Size: 2.0×2.0×2.0 mm³ Acc:: None Rel. SNR: 1.00

Properties

Start measurement without further preparation	On
Wait for User to Start	Off
Start measurements	Single Measurement
Prio Recon	Off
Auto Open Inline Display	Off
Auto Close Inline Display	Off
Load Images to MR View&GO	On
Auto Store Images	On
Disable auto transfer to PACS	Off
Load Images to Stamp Segments	Off
Load Images to Graphic Segments	Off
Graphic segment	Default
Inline Movie	Off

Routine

Slice Group	1
Slices	72
Distance Factor	0 %
Position	Isocenter
Orientation	T > C-30.0
Phase Encoding Dir.	A >> P
Phase Oversampling	0 %
FOV Read	216 mm
FOV Phase	111.1 %
Slice Thickness	2.0 mm
TR	10100.0 ms
TE	84.00 ms
Averages	1
Multi-band accel. factor	1
AutoAlign	---

Contrast - Common

TR	10100.0 ms
TE	84.00 ms
MTC	Off
Magn. Preparation	None
Flip Angle	90 deg
Refocus flip angle	180 deg
Fat-Water Contrast	Fat Saturation
Grad. rev. fat suppr.	Disabled
Contrasts	1
Reconstruction	Magnitude

Contrast - Dynamic

Dynamic Mode	Standard
Measurements	1
Delay in TR	0.00 ms

Resolution - Common

FOV Read	216 mm
FOV Phase	111.1 %
Slice Thickness	2.0 mm
Base Resolution	108
Phase Resolution	100 %
Interpolation	Off

Resolution - Acceleration

Acceleration Mode	None
Phase Partial Fourier	Off

Resolution - Filter

Raw Filter	Off
Elliptical Filter	Off
Hamming	Off
Distortion Correction	Off
Static Field Correction	Off
Normalize	Prescan

Geometry - Common

Slice Group	1
Slices	72
Distance Factor	0 %
Position	Isocenter
Orientation	T > C-30.0
Phase Encoding Dir.	A >> P
Phase Oversampling	0 %
FOV Read	216 mm
FOV Phase	111.1 %
Slice Thickness	2.0 mm
TR	10100.0 ms
Multi-Slice Mode	Interleaved
Series	Interleaved
Multi-band accel. factor	1

Geometry - AutoAlign

Slice Group	1
Position	Isocenter
Orientation	T > C-30.0
Phase Encoding Dir.	A >> P
AutoAlign	---
Initial Position	Isocenter
L	0.0 mm
P	0.0 mm
H	0.0 mm
Initial Orientation	T > C
T > C	-30.00
> S	0.00
Initial Rotation	0.00 deg

Geometry - Saturation

Special Saturation	None

Geometry - Tim Planning Suite

Set-n-Go Protocol	Off
Table Position	0 mm
Table Position	H
Inline Composing	Off

System - Miscellaneous

Coil Selection	ACS All but spine
Radial Sorting	Off
MSMA	S - C - T
Sagittal	R >> L
Coronal	A >> P
Transversal	F >> H
Coil Combination	Sum of Squares
Matrix Optimization	Off
Coil Focus	Flat

System - Adjustments

Adjustment Strategy	Standard
B0 Shim	Standard
B1 Shim	TrueForm
Adjustment Tolerance	Auto
Adjust with Body Coil	Off
Confirm Frequency	Never
Assume Silicone	Off

System - Adjust Volume

Position	Isocenter
Orientation	T > C-30.0
Rotation	90.00 deg
R >> L	216 mm
A >> P	240 mm
F >> H	144 mm
Reset	Off

System - pTx

B1 Shim	TrueForm
Excitation	Standard

System - Tx/Rx

Frequency 1H	123.237845 MHz
? Ref. Amplitude 1H	0.000 V
Reset	Off
Image Scaling	1.000

Physio - Signal

1st Signal/Mode	None
TR	10100.0 ms
Log Signals	Off
Multi-band accel. factor	1

BOLD

GLM Statistics	Off
Ignore Meas. at Start	0
Ignore After Transition	0
Model Transition States	On
Temp. Highpass Filter	On
Threshold	4.00
Paradigm Size	4
Meas[1]	Baseline
Meas[2]	Baseline
Meas[3]	Baseline
Meas[4]	Active
Motion Correction	Off
Spatial Filter	Off
Measurements	1
Delay in TR	0.00 ms

Sequence - Part 1

Sequence Name	epse
Dimension	2D
Excitation	Standard
RF Pulse Type	Normal
Gradient Mode	Performance
Bandwidth	2104 Hz/Px
Echo Spacing	0.61 ms
Free Echo Spacing	On
EPI Factor	120

Sequence - Part 2

Introduction	Off

Sequence - Special

Min. prep scans	0
Delay before PC scans	0 us
SENSE1 coil combine	On
Invert RO/PE polarity	On
Force GPA balance	Off
Disable B1 control loop	Off
Disable freq. update	Off
Suppress 16-bit DICOM	Off
Force equal slice timing	Off
FFT scale factor	1.00
Fat saturation FA	110.00 deg
Fat sat. offset	0.00 Hz
Physio recording	DICOM
Triggering scheme	Standard

Sequence - Assistant

SAR Assistant	Off

AEP MRI Protocol for Siemens 3T Vida (XA60)

Table of contents

\\USER

2023_MBC_ VIDA PROTOCOLS_XA50

SB.2020.353 AEP study - 64 Channel

SB.2020.353 - AEP MRI ACQUISITION PROTOCOL v23_Vida- 64 channel

Localizer- *
T2w_TSE_Axial_RL- *
MEGRE_Ax_RL- *
T1w_Sag_AP- *
T2wFLAIR_Sag_AP- *
T2w_CorOblHipp_RL- *
T2map_CorOblHipp_RL- *
T2w_AxOblTempPole_RL- *
DWI_Ax_AP_52Dir_AP- *
DWI_Ax_AP_52Dir_APinvROPE- *
T1map_MP2RAGE_Sag_AP- *
Localizer-OptionalREPEAT- *
T1w_Sag_AP- OptionalREPEAT- *
T2wFLAIR_Sag_AP-OptionalREPEAT- *
T2w_CorOblHipp_RL-OptionalREPEAT- *
fMRI_AxObl_AP_Pseudoword- *
FMap_AxObl_AP- *
FMap_AxObl_AP-InvROPE- *
fMRI_AxObl_AP_OneBack- *
FMap_AxObl_AP- *
FMap_AxObl_AP-InvROPE- *
fMRI_AxObl_AP_Movie- *
FMap_AxObl_AP- *
FMap_AxObl_AP-InvROPE- *

\\USER\2023_MBC_ VIDA PROTOCOLS_XA50\SB.2020.353 AEP study - 64 Channel\SB.2020.353 - AEP MRI ACQUISITION PROTOCOL v23_Vida- 64 channel\Localizer- *

TA: 12 sec Coil Selection: Auto Voxel Size: 0.5×0.5×7.0 mm³ Acc:: None Rel. SNR: 1.00

Properties

Start measurement without further preparation	Off
Wait for User to Start	Off
Start measurements	Single Measurement
Prio Recon	On
Auto Open Inline Display	Off
Auto Close Inline Display	Off
Load Images to MR View&GO	On
Auto Store Images	On
Disable auto transfer to PACS	Off
Load Images to Stamp Segments	On
Load Images to Graphic Segments	On
Graphic segment	All Segments
Inline Movie	Off

Routine

Slice Group	1
Slices	1
Distance Factor	20 %
Position	Isocenter
Orientation	Sagittal
Phase Encoding Dir.	A >> P
Slice Group	2
Slices	1
Distance Factor	20 %
Position	Isocenter
Orientation	Sagittal
Phase Encoding Dir.	A >> P
Slice Group	3
Slices	1
Distance Factor	20 %
Position	Isocenter
Orientation	Sagittal
Phase Encoding Dir.	A >> P
Phase Oversampling	0 %
FOV Read	250 mm
FOV Phase	100.0 %
Slice Thickness	7.0 mm
TR	7.5 ms
TE	3.69 ms
Averages	2
Concatenations	3
AutoAlign	---

Contrast - Common

TR	7.5 ms
TE	3.69 ms
TD	0.00 ms
MTC	Off

Contrast - Common

Magn. Preparation	None
Flip Angle	20 deg
Fat-Water Contrast	Standard
Dark Blood	Off
Contrasts	1
SWI	Off
Reconstruction	Magnitude

Contrast - Dynamic

Dynamic Mode	Standard
Measurements	1
Multiple Series	Each Measurement

Resolution - Common

FOV Read	250 mm
FOV Phase	100.0 %
Slice Thickness	7.0 mm
Base Resolution	256
Phase Resolution	91 %
Interpolation	On

Resolution - Acceleration

Acceleration Mode	None
Deep Resolve	Off
Phase Partial Fourier	Off
Asymmetric Echo	Allowed

Resolution - Filter

Raw Filter	Off
Elliptical Filter	On
POCS	Off
Distortion Correction	2D
Normalize	Prescan
Image Filter	Off

Geometry - Common

Slice Group	1
Slices	1
Distance Factor	20 %
Position	Isocenter
Orientation	Sagittal
Phase Encoding Dir.	A >> P
Slice Group	2
Slices	1
Distance Factor	20 %
Position	Isocenter
Orientation	Sagittal
Phase Encoding Dir.	A >> P
Slice Group	3

Geometry - Common

Slices	1
Distance Factor	20 %
Position	Isocenter
Orientation	Sagittal
Phase Encoding Dir.	A >> P
Phase Oversampling	0 %
FOV Read	250 mm
FOV Phase	100.0 %
Slice Thickness	7.0 mm
TR	7.5 ms
Multi-Slice Mode	Sequential
Series	Interleaved
Concatenations	3

Geometry - AutoAlign

Slice Group	1
Position	Isocenter
Orientation	Sagittal
Phase Encoding Dir.	A >> P
Slice Group	2
Position	Isocenter
Orientation	Sagittal
Phase Encoding Dir.	A >> P
Slice Group	3
Position	Isocenter
Orientation	Sagittal
Phase Encoding Dir.	A >> P
AutoAlign	---
Initial Position	Isocenter
L	0.0 mm
P	0.0 mm
H	0.0 mm
Initial Orientation	Coronal
Initial Rotation	0.00 deg

Geometry - Saturation

Saturation Mode	Standard
Special Saturation	None

Geometry - Tim Planning Suite

Set-n-Go Protocol	Off
Table Position	0 mm
Table Position	H
Inline Composing	Off

System - Miscellaneous

Coil Selection	Auto Coil Select
Radial Sorting	Off
MSMA	S - C - T
Sagittal	R >> L
Coronal	A >> P
Transversal	F >> H
Coil Combination	Adaptive Combine
Matrix Optimization	Off

System - Miscellaneous

Coil Focus	Flat

System - Adjustments

Adjustment Strategy	Standard
B0 Shim	Tune up
B1 Shim	TrueForm
CoilShim	Off
Adjustment Tolerance	Auto
Adjust with Body Coil	Off
Confirm Frequency	Never
Assume Silicone	Off

System - Adjust Volume

Position	Isocenter
Orientation	Transversal
Rotation	0.00 deg
A >> P	263 mm
R >> L	350 mm
F >> H	350 mm
Reset	Off

System - pTx

B1 Shim	TrueForm
Excitation	Slice-sel.
LR Balancing	Off

System - Tx/Rx

Frequency 1H	123.213354 MHz
? Ref. Amplitude 1H	0.000 V
Reset	Off
Image Scaling	1.000

Physio - Signal

1st Signal/Mode	None
TR	7.5 ms
Segments	1
Concatenations	3

Physio - Cardiac

Tagging	None
Fat-Water Contrast	Standard
Magn. Preparation	None
Dark Blood	Off
FOV Read	250 mm
FOV Phase	100.0 %
Phase Resolution	91 %

Physio - PACE

Resp. Control	Off
Concatenations	3

Inline - Liver

Liver Registration	Off
Save Original Images	On

Inline - Subtraction

Subtract	Off
Measurements	1
StdDev	Off
Save Original Images	On

Inline - MIP

MIP Sag	Off
MIP Cor	Off
MIP Tra	Off
MIP Time	Off
Radial MIP	Off
Save Original Images	On
MPR Sag	Off
MPR Cor	Off
MPR Tra	Off

Inline - Soft Tissue

Wash-in	Off
Wash-out	Off
TTP	Off
PEI	Off
MIP Time	Off
Measurements	1

Inline - Composing

Inline Composing	Off

Inline - MapIt

MapIt	None
Flip Angle	20 deg
Measurements	1
Contrasts	1
TE	3.69 ms
TR	7.5 ms
Save Original Images	On

Sequence - Part 1

Sequence Name	fl
Dimension	2D
Excitation	Slice-sel.
RF Pulse Type	Fast
Gradient Mode	Fast
Flow Compensation	None
Bandwidth	340 Hz/Px
Asymmetric Echo	Allowed
Segments	1

Sequence - Part 2

Introduction	On
RF Spoiling	On
Acoustic noise reduction	Off

Sequence - Assistant

SAR Assistant	Off

Sequence - Assistant

Allowed Delay	0 s

\\USER\2023_MBC_ VIDA PROTOCOLS_XA50\SB.2020.353 AEP study - 64 Channel\SB.2020.353 - AEP MRI ACQUISITION PROTOCOL v23_Vida- 64 channel\T2w_TSE_Axial_RL- *

TA: 3:28 min Coil Selection: Auto Voxel Size: 0.2×0.2×3.0 mm³ Acc:: None Rel. SNR: 1.00

Properties

Start measurement without further preparation	On
Wait for User to Start	Off
Start measurements	Single Measurement
Prio Recon	Off
Auto Open Inline Display	Off
Auto Close Inline Display	Off
Load Images to MR View&GO	On
Auto Store Images	On
Disable auto transfer to PACS	Off
Load Images to Stamp Segments	On
Load Images to Graphic Segments	Off
Graphic segment	Default
Inline Movie	Off

Routine

Slice Group	1
Slices	44
Distance Factor	0 %
Position	Isocenter
Orientation	Transversal
Phase Encoding Dir.	R >> L
Phase Oversampling	5 %
FOV Read	208 mm
FOV Phase	84.4 %
Slice Thickness	3.0 mm
TR	4300.0 ms
TE	83.00 ms
Averages	1
Concatenations	2
AutoAlign	---

Contrast - Common

TR	4300.0 ms
TE	83.00 ms
TD	0.00 ms
MTC	Off
Magn. Preparation	None
Flip Angle Mode	Constant
Flip Angle	120 deg
Fat-Water Contrast	Standard
Dark Blood	Off
Contrasts	1
Wrap-up Magn.	None
Reconstruction	Magnitude

Contrast - Dynamic

Dynamic Mode	Standard
Measurements	1

Contrast - Dynamic

Multiple Series	Each Measurement

Resolution - Common

FOV Read	208 mm
FOV Phase	84.4 %
Slice Thickness	3.0 mm
Base Resolution	448
Phase Resolution	100 %
Interpolation	On

Resolution - Acceleration

Acceleration Mode	None
Deep Resolve	Off
Phase Partial Fourier	Off

Resolution - Filter

Raw Filter	Off
Elliptical Filter	Off
Distortion Correction	2D
Normalize	Prescan
Image Filter	On

Geometry - Common

Slice Group	1
Slices	44
Distance Factor	0 %
Position	Isocenter
Orientation	Transversal
Phase Encoding Dir.	R >> L
Phase Oversampling	5 %
FOV Read	208 mm
FOV Phase	84.4 %
Slice Thickness	3.0 mm
TR	4300.0 ms
Multi-Slice Mode	Interleaved
Series	Interleaved
Concatenations	2

Geometry - AutoAlign

Slice Group	1
Position	Isocenter
Orientation	Transversal
Phase Encoding Dir.	R >> L
AutoAlign	---
Initial Position	Isocenter
L	0.0 mm
P	0.0 mm
H	0.0 mm
Initial Orientation	Transversal

Geometry - AutoAlign

Initial Rotation	90.00 deg

Geometry - Navigator

Geometry - Saturation

Special Saturation	None

Geometry - Tim Planning Suite

Set-n-Go Protocol	Off
Table Position	0 mm
Table Position	H
Inline Composing	Off

System - Miscellaneous

Coil Selection	Auto Coil Select
Radial Sorting	Off
MSMA	S - C - T
Sagittal	R >> L
Coronal	A >> P
Transversal	F >> H
Coil Combination	Adaptive Combine
Matrix Optimization	Off
Coil Focus	Flat

System - Adjustments

Adjustment Strategy	Standard
B0 Shim	Standard
B1 Shim	TrueForm
CoilShim	Off
Adjustment Tolerance	Auto
Adjust with Body Coil	Off
Confirm Frequency	Never
Assume Silicone	Off

System - Adjust Volume

Position	Isocenter
Orientation	Transversal
Rotation	90.00 deg
R >> L	176 mm
A >> P	208 mm
F >> H	132 mm
Reset	Off

System - pTx

B1 Shim	TrueForm
LR Balancing	Off

System - Tx/Rx

Frequency 1H	123.213354 MHz
? Ref. Amplitude 1H	0.000 V
Reset	Off
Image Scaling	1.000

Physio - Signal

1st Signal/Mode	None
TR	4300.0 ms
Concatenations	2

Physio - Cardiac

Fat-Water Contrast	Standard
Magn. Preparation	None
Dark Blood	Off
FOV Read	208 mm
FOV Phase	84.4 %
Phase Resolution	100 %
Motion Correction	None

Physio - PACE

Resp. Control	Off
Concatenations	2

Inline - Subtraction

Subtract	Off
Measurements	1
StdDev	Off
Motion Correction	None
Save Original Images	On

Inline - MIP

MIP Sag	Off
MIP Cor	Off
MIP Tra	Off
MIP Time	Off
Radial MIP	Off
Save Original Images	On
MPR Sag	Off
MPR Cor	Off
MPR Tra	Off

Inline - Composing

Inline Composing	Off

Sequence - Part 1

Sequence Name	tse_rr
Dimension	2D
RF Pulse Type	Fast
Gradient Mode	Fast
Flow Compensation	Read
Bandwidth	180 Hz/Px
Echo Spacing	10.4 ms
Free Echo Spacing	Off
Define	Turbo Factor
Turbo Factor	18
Echo Trains per Slice	23

Sequence - Part 2

Introduction	On
Phase Correction	Automatic

Sequence - Part 2

Compensate T2 Decay	Off
Fast Mode	Off
WARP	Off
Red. EC Sensitivity	Off
Acoustic noise reduction	Off
Reduce Motion Sens.	On
Motion Correction	None

Sequence - Assistant

SAR Assistant	TR
Max. TR	6500.0 ms
Allowed Delay	60 s

\\USER\2023_MBC_ VIDA PROTOCOLS_XA50\SB.2020.353 AEP study - 64 Channel\SB.2020.353 - AEP MRI ACQUISITION PROTOCOL v23_Vida- 64 channel\MEGRE_Ax_RL- *

TA: 3:39 min Coil Selection: Auto Voxel Size: 1.2×1.2×1.2 mm³ Acc:: 3 Rel. SNR: 1.00

Properties

Start measurement without further preparation	On
Wait for User to Start	On
Start measurements	Single Measurement
Prio Recon	Off
Auto Open Inline Display	Off
Auto Close Inline Display	Off
Load Images to MR View&GO	On
Auto Store Images	On
Disable auto transfer to PACS	Off
Load Images to Stamp Segments	Off
Load Images to Graphic Segments	Off
Graphic segment	Default
Inline Movie	Off

Routine

Slab Group	1
Slabs	1
Distance Factor	20 %
Position	Isocenter
Orientation	Transversal
Phase Encoding Dir.	R >> L
Slices per Slab	120
Phase Oversampling	10 %
Slice Oversampling	0.0 %
FOV Read	230 mm
FOV Phase	81.3 %
Slice Thickness	1.20 mm
TR	30.0 ms
TE 1	5.84 ms
TE 2	10.63 ms
TE 3	15.42 ms
TE 4	20.21 ms
TE 5	25.00 ms
Averages	1
Concatenations	1
AutoAlign	---

Contrast - Common

TR	30.0 ms
TE 1	5.84 ms
TE 2	10.63 ms
TE 3	15.42 ms
TE 4	20.21 ms
TE 5	25.00 ms
MTC	Off
Magn. Preparation	None
Flip Angle	15 deg
Fat-Water Contrast	Standard

Contrast - Common

Dark Blood	Off
Contrasts	5
SWI	Off
Reconstruction	Magn./Phase

Contrast - Dynamic

Dynamic Mode	Standard
Measurements	1
Multiple Series	Each Measurement

Resolution - Common

FOV Read	230 mm
FOV Phase	81.3 %
Slice Thickness	1.20 mm
Base Resolution	192
Phase Resolution	100 %
Slice Resolution	100 %
Interpolation	Off

Resolution - Acceleration

Acceleration Mode	GRAPPA
Reference Scans	Integrated
Acceleration Factor PE	3
Reference Lines PE	24
Acceleration Factor 3D	1
Deep Resolve	Off
Phase Partial Fourier	Off
Slice Partial Fourier	Off
Asymmetric Echo	Off
Elliptical Scanning	On

Resolution - Filter

Raw Filter	Off
Elliptical Filter	Off
POCS	Off
Distortion Correction	2D
Normalize	Prescan
Image Filter	Off

Geometry - Common

Slab Group	1
Slabs	1
Distance Factor	20 %
Position	Isocenter
Orientation	Transversal
Phase Encoding Dir.	R >> L
Slices per Slab	120
Phase Oversampling	10 %
Slice Oversampling	0.0 %

Geometry - Common

FOV Read	230 mm
FOV Phase	81.3 %
Slice Thickness	1.20 mm
TR	30.0 ms
Multi-Slice Mode	Interleaved
Series	Interleaved
Concatenations	1

Geometry - AutoAlign

Slab Group	1
Position	Isocenter
Orientation	Transversal
Phase Encoding Dir.	R >> L
AutoAlign	---
Initial Position	Isocenter
L	0.0 mm
P	0.0 mm
H	0.0 mm
Initial Orientation	Transversal
Initial Rotation	90.00 deg

Geometry - Saturation

Saturation Mode	Standard
Special Saturation	None

Geometry - Tim Planning Suite

Set-n-Go Protocol	Off
Table Position	0 mm
Table Position	H
Inline Composing	Off

System - Miscellaneous

Coil Selection	ACS All but spine
Radial Sorting	Off
MSMA	S - C - T
Sagittal	R >> L
Coronal	A >> P
Transversal	F >> H
Coil Combination	Adaptive Combine
Matrix Optimization	Off
Coil Focus	Flat

System - Adjustments

Adjustment Strategy	Standard
B0 Shim	Standard
B1 Shim	TrueForm
CoilShim	Off
Adjustment Tolerance	Auto
Adjust with Body Coil	Off
Confirm Frequency	Never
Assume Silicone	Off

System - Adjust Volume

Position	Isocenter

System - Adjust Volume

Orientation	Transversal
Rotation	90.00 deg
R >> L	187 mm
A >> P	230 mm
F >> H	144 mm
Reset	Off

System - pTx

B1 Shim	TrueForm
Excitation	Slab-sel.
LR Balancing	Off

System - Tx/Rx

Frequency 1H	123.213354 MHz
? Ref. Amplitude 1H	0.000 V
Reset	Off
Image Scaling	1.000

Physio - Signal

1st Signal/Mode	None
TR	30.0 ms
Segments	1
Concatenations	1

Physio - Cardiac

Tagging	None
Fat-Water Contrast	Standard
Magn. Preparation	None
Dark Blood	Off
FOV Read	230 mm
FOV Phase	81.3 %
Phase Resolution	100 %

Physio - PACE

Resp. Control	Off
Concatenations	1

Inline - Liver

Liver Registration	Off
Save Original Images	On

Inline - Subtraction

Subtract	Off
Measurements	1
StdDev	Off
Save Original Images	On

Inline - MIP

MIP Sag	Off
MIP Cor	Off
MIP Tra	Off
MIP Time	Off
Radial MIP	Off
Save Original Images	On

Inline - MIP

MPR Sag	Off
MPR Cor	Off
MPR Tra	Off

Inline - Soft Tissue

Wash-in	Off
Wash-out	Off
TTP	Off
PEI	Off
MIP Time	Off
Measurements	1

Inline - Composing

Inline Composing	Off

Inline - MapIt

MapIt	None
Flip Angle	15 deg
Measurements	1
Contrasts	5
TE 1	5.84 ms
TE 2	10.63 ms
TE 3	15.42 ms
TE 4	20.21 ms
TE 5	25.00 ms
TR	30.0 ms
Save Original Images	On

Sequence - Part 1

Sequence Name	fl_r
Dimension	3D
Excitation	Slab-sel.
RF Pulse Type	Fast
Readout Mode	Monopolar
Gradient Mode	Normal
Flow Compensation 1	On
Flow Compensation 2	None
Flow Compensation 3	None
Flow Compensation 4	None
Flow Compensation 5	None
Bandwidth 1	310 Hz/Px
Bandwidth 2	310 Hz/Px
Bandwidth 3	310 Hz/Px
Bandwidth 4	310 Hz/Px
Bandwidth 5	310 Hz/Px
Asymmetric Echo	Off
Segments	1

Sequence - Part 2

Introduction	Off
RF Spoiling	On
Acoustic noise reduction	Off

Sequence - Assistant

SAR Assistant	Off
Allowed Delay	0 s

\\USER\2023_MBC_ VIDA PROTOCOLS_XA50\SB.2020.353 AEP study - 64 Channel\SB.2020.353 - AEP MRI ACQUISITION PROTOCOL v23_Vida- 64 channel\T1w_Sag_AP- *

TA: 4:33 min Coil Selection: Auto Voxel Size: 0.9×0.9×0.9 mm³ Acc:: 2 Rel. SNR: 1.00

Properties

Start measurement without further preparation	On
Wait for User to Start	Off
Start measurements	Single Measurement
Prio Recon	Off
Auto Open Inline Display	Off
Auto Close Inline Display	Off
Load Images to MR View&GO	On
Auto Store Images	On
Disable auto transfer to PACS	Off
Load Images to Stamp Segments	Off
Load Images to Graphic Segments	Off
Graphic segment	Default
Inline Movie	Off

Routine

Slab Group	1
Slabs	1
Distance Factor	50 %
Position	Isocenter
Orientation	Sagittal
Phase Encoding Dir.	A >> P
Slices per Slab	192
Phase Oversampling	0 %
Slice Oversampling	25.0 %
FOV Read	230 mm
FOV Phase	100.0 %
Slice Thickness	0.90 mm
TR	1910.0 ms
TE	2.19 ms
Averages	1
Concatenations	1
AutoAlign	---

Contrast - Common

TR	1910.0 ms
TE	2.19 ms
Magn. Preparation	Non-sel. IR
TI	1000 ms
Flip Angle	8 deg
Fat-Water Contrast	Fast Water Excitation
Dark Blood	Off
Contrasts	1
Reconstruction	Magnitude

Contrast - Dynamic

Dynamic Mode	Standard
Measurements	1
Multiple Series	Each Measurement

Contrast - Dynamic

Reordering	Linear

Resolution - Common

FOV Read	230 mm
FOV Phase	100.0 %
Slice Thickness	0.90 mm
Base Resolution	256
Phase Resolution	100 %
Slice Resolution	100 %
Interpolation	Off

Resolution - Acceleration

Acceleration Mode	GRAPPA
Reference Scans	Integrated
Acceleration Factor PE	2
Reference Lines PE	32
Acceleration Factor 3D	1
Phase Partial Fourier	Off
Slice Partial Fourier	Off
Asymmetric Echo	Allowed
Elliptical Scanning	Off

Resolution - Filter

Raw Filter	Off
Elliptical Filter	Off
Distortion Correction	2D
Normalize	Prescan
Image Filter	Off

Geometry - Common

Slab Group	1
Slabs	1
Distance Factor	50 %
Position	Isocenter
Orientation	Sagittal
Phase Encoding Dir.	A >> P
Slices per Slab	192
Phase Oversampling	0 %
Slice Oversampling	25.0 %
FOV Read	230 mm
FOV Phase	100.0 %
Slice Thickness	0.90 mm
TR	1910.0 ms
Multi-Slice Mode	Single Shot
Series	Interleaved
Concatenations	1

Geometry - AutoAlign

Slab Group	1

Geometry - AutoAlign

Position	Isocenter
Orientation	Sagittal
Phase Encoding Dir.	A >> P
AutoAlign	---
Initial Position	Isocenter
L	0.0 mm
P	0.0 mm
H	0.0 mm
Initial Orientation	Sagittal
Initial Rotation	0.00 deg

Geometry - Navigator

Geometry - Tim Planning Suite

Set-n-Go Protocol	Off
Table Position	0 mm
Table Position	H
Inline Composing	Off

System - Miscellaneous

Coil Selection	Auto Coil Select
Radial Sorting	Off
MSMA	S - C - T
Sagittal	R >> L
Coronal	P >> A
Transversal	F >> H
Coil Combination	Adaptive Combine
Matrix Optimization	Off
Coil Focus	Flat

System - Adjustments

Adjustment Strategy	Standard
B0 Shim	Standard
B1 Shim	TrueForm
CoilShim	Off
Adjustment Tolerance	Auto
Adjust with Body Coil	Off
Confirm Frequency	Never
Assume Silicone	Off

System - Adjust Volume

Position	Isocenter
Orientation	Sagittal
Rotation	0.00 deg
A >> P	230 mm
F >> H	230 mm
R >> L	173 mm
Reset	Off

System - pTx

B1 Shim	TrueForm
Excitation	Non-sel.

System - Tx/Rx

Frequency 1H	123.213354 MHz
? Ref. Amplitude 1H	0.000 V
Reset	Off
Image Scaling	1.000

Physio - Signal

1st Signal/Mode	None
TR	1910.0 ms
Concatenations	1

Physio - Cardiac

Fat-Water Contrast	Fast Water Excitation
Magn. Preparation	Non-sel. IR
TI	1000 ms
Dark Blood	Off
FOV Read	230 mm
FOV Phase	100.0 %
Phase Resolution	100 %

Physio - PACE

Resp. Control	Off
Concatenations	1

Inline - Subtraction

Subtract	Off
Measurements	1
StdDev	Off
Save Original Images	On

Inline - MIP

MIP Sag	Off
MIP Cor	Off
MIP Tra	Off
MIP Time	Off
Radial MIP	Off
Save Original Images	On
MPR Sag	Off
MPR Cor	Off
MPR Tra	Off

Inline - Composing

Inline Composing	Off

Inline - MapIt

MapIt	None
Flip Angle	8 deg
Measurements	1
Contrasts	1
TE	2.19 ms
TR	1910.0 ms
Save Original Images	On

Sequence - Part 1

Sequence Name	tfl

Sequence - Part 1

Dimension	3D
Excitation	Non-sel.
RF Pulse Type	Fast
Gradient Mode	Performance
Flow Compensation	None
Reordering	Linear
Bandwidth	220 Hz/Px
Echo Spacing	7.58 ms
Asymmetric Echo	Allowed
Turbo Factor	240

Sequence - Part 2

Introduction	Off
RF Spoiling	On
Incr. Gradient Spoiling	Off

Sequence - Assistant

SAR Assistant	Off

\\USER\2023_MBC_ VIDA PROTOCOLS_XA50\SB.2020.353 AEP study - 64 Channel\SB.2020.353 - AEP MRI ACQUISITION PROTOCOL v23_Vida- 64 channel\T2wFLAIR_Sag_AP- *

TA: 5:32 min Coil Selection: Auto Voxel Size: 0.9×0.9×0.9 mm^3 Acc:: 2 Rel. SNR: 1.00

Properties

Start measurement without further preparation	On
Wait for User to Start	On
Start measurements	Single Measurement
Prio Recon	Off
Auto Open Inline Display	Off
Auto Close Inline Display	Off
Load Images to MR View&GO	On
Auto Store Images	On
Disable auto transfer to PACS	Off
Load Images to Stamp Segments	Off
Load Images to Graphic Segments	Off
Graphic segment	Default
Inline Movie	Off

Routine

Slab Group	1
Slabs	1
Position	Isocenter
Orientation	Sagittal
Phase Encoding Dir.	A >> P
Slices per Slab	192
Phase Oversampling	0 %
Slice Oversampling	0.0 %
FOV Read	230 mm
FOV Phase	100.0 %
Slice Thickness	0.90 mm
TR	5000.0 ms
TE	392.00 ms
Averages	1.0
Concatenations	1
AutoAlign	---

Contrast - Common

TR	5000.0 ms
TE	392.00 ms
MTC	Off
Magn. Preparation	Non-sel. T2-IR
TI 1	1800 ms
Flip Angle Mode	T2 Var
Fat-Water Contrast	Standard
Dark Blood	Off
Blood Suppression	Off
Wrap-up Magn.	None
Reconstruction	Magnitude

Contrast - Dynamic

Dynamic Mode	Standard
Measurements	1

Contrast - Dynamic

Multiple Series	Each Measurement
Reordering	Linear

Resolution - Common

FOV Read	230 mm
FOV Phase	100.0 %
Slice Thickness	0.90 mm
Base Resolution	256
Phase Resolution	100 %
Slice Resolution	100 %
Interpolation	Off

Resolution - Acceleration

Acceleration Mode	GRAPPA
Total Factor	2
Reference Scans	Integrated
Acceleration Factor PE	2
Reference Lines PE	24
Acceleration Factor 3D	1
Phase Partial Fourier	Allowed
Slice Partial Fourier	6/8
Elliptical Scanning	Off

Resolution - Filter

Raw Filter	Off
Elliptical Filter	Off
Distortion Correction	2D
Normalize	Prescan
Image Filter	On

Geometry - Common

Slab Group	1
Slabs	1
Position	Isocenter
Orientation	Sagittal
Phase Encoding Dir.	A >> P
Slices per Slab	192
Phase Oversampling	0 %
Slice Oversampling	0.0 %
FOV Read	230 mm
FOV Phase	100.0 %
Slice Thickness	0.90 mm
TR	5000.0 ms
Concatenations	1

Geometry - AutoAlign

Slab Group	1
Position	Isocenter
Orientation	Sagittal

Geometry - AutoAlign

Phase Encoding Dir.	A >> P
AutoAlign	---
Initial Position	Isocenter
L	0.0 mm
P	0.0 mm
H	0.0 mm
Initial Orientation	Sagittal
Initial Rotation	0.00 deg

Geometry - Navigator

Geometry - Saturation

Special Saturation	None

Geometry - Tim Planning Suite

Set-n-Go Protocol	Off
Table Position	0 mm
Table Position	H
Inline Composing	Off

System - Miscellaneous

Coil Selection	ACS All but spine
Radial Sorting	Off
MSMA	S - C - T
Sagittal	R >> L
Coronal	A >> P
Transversal	F >> H
Coil Combination	Adaptive Combine
Matrix Optimization	Off
Coil Focus	Flat

System - Adjustments

Adjustment Strategy	Standard
B0 Shim	Standard
B1 Shim	TrueForm
CoilShim	Off
Adjustment Tolerance	Auto
Adjust with Body Coil	Off
Confirm Frequency	Never
Assume Silicone	Off

System - Adjust Volume

Position	Isocenter
Orientation	Sagittal
Rotation	0.00 deg
A >> P	230 mm
F >> H	230 mm
R >> L	173 mm
Reset	Off

System - pTx

B1 Shim	TrueForm
Excitation	Non-sel.

System - Tx/Rx

Frequency 1H	123.213354 MHz
? Ref. Amplitude 1H	0.000 V
Reset	Off
Image Scaling	1.000
Gain	High

Physio - Signal

1st Signal/Mode	None
Trigger Delay	0 ms
TR	5000.0 ms
Concatenations	1

Physio - Cardiac

Fat-Water Contrast	Standard
Magn. Preparation	Non-sel. T2-IR
TI 1	1800 ms
Dark Blood	Off
FOV Read	230 mm
FOV Phase	100.0 %
Phase Resolution	100 %

Physio - PACE

Resp. Control	Off
Concatenations	1

Inline - Subtraction

Subtract	Off
Measurements	1
StdDev	Off
Save Original Images	On

Inline - MIP

MIP Sag	Off
MIP Cor	Off
MIP Tra	Off
MIP Time	Off
Radial MIP	Off
Save Original Images	On
MPR Sag	Off
MPR Cor	Off
MPR Tra	Off

Inline - Composing

Inline Composing	Off

Sequence - Part 1

Sequence Name	spcir
Dimension	3D
Excitation	Non-sel.
RF Pulse Type	Normal
Gradient Mode	Fast
Flow Compensation	None
Reordering	Linear
Bandwidth	781 Hz/Px

Sequence - Part 1

Echo Spacing	3.60 ms
Turbo Factor	310
Echo Train Duration	947 ms

Sequence - Part 2

Introduction	On

Sequence - Assistant

SAR Assistant	Off
Allowed Delay	0 s

\\USER\2023_MBC_ VIDA PROTOCOLS_XA50\SB.2020.353 AEP study - 64 Channel\SB.2020.353 - AEP MRI ACQUISITION PROTOCOL v23_Vida- 64 channel\T2w_CorOblHipp_RL- *

TA: 4:53 min Coil Selection: Auto Voxel Size: 0.4×0.4×2.0 mm³ Acc:: None Rel. SNR: 1.00

Properties

Start measurement without further preparation	On
Wait for User to Start	On
Start measurements	Single Measurement
Prio Recon	Off
Auto Open Inline Display	Off
Auto Close Inline Display	Off
Load Images to MR View&GO	On
Auto Store Images	On
Disable auto transfer to PACS	Off
Load Images to Stamp Segments	On
Load Images to Graphic Segments	Off
Graphic segment	Default
Inline Movie	Off

Routine

Slice Group	1
Slices	36
Distance Factor	0 %
Position	Isocenter
Orientation	C > T-25.0
Phase Encoding Dir.	R >> L
Phase Oversampling	54 %
FOV Read	168 mm
FOV Phase	100.0 %
Slice Thickness	2.0 mm
TR	8100.0 ms
TE	83.00 ms
Averages	1
Concatenations	1
AutoAlign	---

Contrast - Common

TR	8100.0 ms
TE	83.00 ms
MTC	Off
Magn. Preparation	None
Flip Angle Mode	Hyperecho
Flip Angle	119 deg
Fat-Water Contrast	Standard
Dark Blood	Off
Contrasts	1
Wrap-up Magn.	Restore
Reconstruction	Magnitude

Contrast - Dynamic

Dynamic Mode	Standard
Measurements	1
Multiple Series	Each Measurement

Resolution - Common

FOV Read	168 mm
FOV Phase	100.0 %
Slice Thickness	2.0 mm
Base Resolution	384
Phase Resolution	100 %
Interpolation	Off

Resolution - Acceleration

Acceleration Mode	None
Deep Resolve	Off
Phase Partial Fourier	Off

Resolution - Filter

Raw Filter	Off
Elliptical Filter	Off
Distortion Correction	2D
Normalize	Prescan
Image Filter	On

Geometry - Common

Slice Group	1
Slices	36
Distance Factor	0 %
Position	Isocenter
Orientation	C > T-25.0
Phase Encoding Dir.	R >> L
Phase Oversampling	54 %
FOV Read	168 mm
FOV Phase	100.0 %
Slice Thickness	2.0 mm
TR	8100.0 ms
Multi-Slice Mode	Interleaved
Series	Interleaved
Concatenations	1

Geometry - AutoAlign

Slice Group	1
Position	Isocenter
Orientation	C > T-25.0
Phase Encoding Dir.	R >> L
AutoAlign	---
Initial Position	Isocenter
L	0.0 mm
P	0.0 mm
H	0.0 mm
Initial Orientation	C > T
C > T	-25.00
> S	0.00
Initial Rotation	0.00 deg

Geometry - Navigator

Geometry - Saturation

Saturation Region	1
Thickness	75.00 mm
Position	L0.0 P4.8 F105.8 mm
Orientation	Transversal
Shape	Standard
Special Saturation	None

Geometry - Tim Planning Suite

Set-n-Go Protocol	Off
Table Position	0 mm
Table Position	H
Inline Composing	Off

System - Miscellaneous

Coil Selection	ACS All but spine
Radial Sorting	Off
MSMA	S - C - T
Sagittal	L >> R
Coronal	A >> P
Transversal	F >> H
Coil Combination	Adaptive Combine
Matrix Optimization	Off
Coil Focus	Flat

System - Adjustments

Adjustment Strategy	Standard
B0 Shim	Standard
B1 Shim	TrueForm
CoilShim	Off
Adjustment Tolerance	Auto
Adjust with Body Coil	Off
Confirm Frequency	Never
Assume Silicone	Off

System - Adjust Volume

Position	Isocenter
Orientation	C > T-25.0
Rotation	0.00 deg
R >> L	168 mm
F >> H	168 mm
A >> P	72 mm
Reset	Off

System - pTx

B1 Shim	TrueForm
LR Balancing	Off

System - Tx/Rx

Frequency 1H	123.213354 MHz
? Ref. Amplitude 1H	0.000 V
Reset	Off
Image Scaling	1.000

Physio - Signal

1st Signal/Mode	None
TR	8100.0 ms
Concatenations	1

Physio - Cardiac

Fat-Water Contrast	Standard
Magn. Preparation	None
Dark Blood	Off
FOV Read	168 mm
FOV Phase	100.0 %
Phase Resolution	100 %
Motion Correction	None

Physio - PACE

Resp. Control	Off
Concatenations	1

Inline - Subtraction

Subtract	Off
Measurements	1
StdDev	Off
Motion Correction	None
Save Original Images	On

Inline - MIP

MIP Sag	Off
MIP Cor	Off
MIP Tra	Off
MIP Time	Off
Radial MIP	Off
Save Original Images	On
MPR Sag	Off
MPR Cor	Off
MPR Tra	Off

Inline - Composing

Inline Composing	Off

Sequence - Part 1

Sequence Name	tseR_rr
Dimension	2D
RF Pulse Type	Fast
Gradient Mode	Fast
Flow Compensation	Read
Bandwidth	181 Hz/Px
Echo Spacing	11.8 ms
Free Echo Spacing	Off
Define	Turbo Factor
Turbo Factor	17
Echo Trains per Slice	35

Sequence - Part 2

Introduction	On
Phase Correction	Automatic

Sequence - Part 2

Compensate T2 Decay	Off
Fast Mode	Off
WARP	Off
Red. EC Sensitivity	Off
Acoustic noise reduction	Off
Reduce Motion Sens.	On
Motion Correction	None

Sequence - Assistant

SAR Assistant	Off
Allowed Delay	60 s

\\USER\2023_MBC_ VIDA PROTOCOLS_XA50\SB.2020.353 AEP study - 64 Channel\SB.2020.353 - AEP MRI ACQUISITION PROTOCOL v23_Vida- 64 channel\T2map_CorOblHipp_RL- *

TA: 3:17 min Coil Selection: Auto Voxel Size: 0.9×0.9×3.6 mm³ Acc:: 2 Rel. SNR: 1.00

Properties

Start measurement without further preparation	On
Wait for User to Start	Off
Start measurements	Single Measurement
Prio Recon	Off
Auto Open Inline Display	Off
Auto Close Inline Display	Off
Load Images to MR View&GO	On
Auto Store Images	On
Disable auto transfer to PACS	Off
Load Images to Stamp Segments	Off
Load Images to Graphic Segments	Off
Graphic segment	Default
Inline Movie	Off

Routine

Slice Group	1
Slices	44
Distance Factor	10 %
Position	Isocenter
Orientation	Coronal
Phase Encoding Dir.	R >> L
Phase Oversampling	0 %
FOV Read	230 mm
FOV Phase	93.8 %
Slice Thickness	3.6 mm
TR	3910.0 ms
TE 1	10.70 ms
TE 2	21.40 ms
TE 3	32.10 ms
TE 4	42.80 ms
TE 5	53.50 ms
TE 6	64.20 ms
TE 7	74.90 ms
TE 8	85.60 ms
TE 9	96.30 ms
TE 10	107.00 ms
TE 11	117.70 ms
TE 12	128.40 ms
TE 13	139.10 ms
TE 14	149.80 ms
TE 15	160.50 ms
TE 16	171.20 ms
Averages	1
Concatenations	2
AutoAlign	---

Contrast - Common

TR	3910.0 ms
TE 1	10.70 ms
TE 2	21.40 ms
TE 3	32.10 ms
TE 4	42.80 ms
TE 5	53.50 ms
TE 6	64.20 ms
TE 7	74.90 ms
TE 8	85.60 ms
TE 9	96.30 ms
TE 10	107.00 ms
TE 11	117.70 ms
TE 12	128.40 ms
TE 13	139.10 ms
TE 14	149.80 ms
TE 15	160.50 ms
TE 16	171.20 ms
TD	0.00 ms
MTC	Off
Magn. Preparation	None
Flip Angle	180 deg
Fat-Water Contrast	Standard
Dark Blood	Off
Contrasts	16
Reconstruction	Magnitude

Contrast - Dynamic

Dynamic Mode	Standard
Measurements	1
Multiple Series	Each Measurement

Resolution - Common

FOV Read	230 mm
FOV Phase	93.8 %
Slice Thickness	3.6 mm
Base Resolution	256
Phase Resolution	100 %

Resolution - Acceleration

Acceleration Mode	GRAPPA
Reference Scans	Integrated
Acceleration Factor PE	2

Resolution - Filter

Raw Filter	Off
Elliptical Filter	Off
Distortion Correction	2D
Normalize	Prescan
Image Filter	On

Geometry - Common

Slice Group	1
Slices	44
Distance Factor	10 %
Position	Isocenter
Orientation	Coronal
Phase Encoding Dir.	R >> L
Phase Oversampling	0 %
FOV Read	230 mm
FOV Phase	93.8 %
Slice Thickness	3.6 mm
TR	3910.0 ms
Multi-Slice Mode	Interleaved
Series	Interleaved
Concatenations	2

Geometry - AutoAlign

Slice Group	1
Position	Isocenter
Orientation	Coronal
Phase Encoding Dir.	R >> L
AutoAlign	---
Initial Position	Isocenter
L	0.0 mm
P	0.0 mm
H	0.0 mm
Initial Orientation	Coronal
Initial Rotation	0.00 deg

Geometry - Navigator

Geometry - Saturation

Special Saturation	None

Geometry - Tim Planning Suite

Set-n-Go Protocol	Off
Table Position	0 mm
Table Position	H
Inline Composing	Off

System - Miscellaneous

Coil Selection	Auto Coil Select
Radial Sorting	Off
MSMA	S - C - T
Sagittal	R >> L
Coronal	A >> P
Transversal	F >> H
Matrix Optimization	Performance
Coil Focus	Flat

System - Adjustments

Adjustment Strategy	Standard
B0 Shim	Standard
B1 Shim	TrueForm
CoilShim	Off

System - Adjustments

Adjustment Tolerance	Auto
Adjust with Body Coil	Off
Confirm Frequency	Never
Assume Silicone	Off

System - Adjust Volume

Position	Isocenter
Orientation	Coronal
Rotation	0.00 deg
R >> L	216 mm
F >> H	230 mm
A >> P	174 mm
Reset	Off

System - pTx

B1 Shim	TrueForm

System - Tx/Rx

Frequency 1H	123.213354 MHz
? Ref. Amplitude 1H	0.000 V
Reset	Off
Image Scaling	1.000

Physio - Signal

1st Signal/Mode	None
TR	3910.0 ms
Concatenations	2

Physio - Cardiac

Fat-Water Contrast	Standard
Magn. Preparation	None
Dark Blood	Off
FOV Read	230 mm
FOV Phase	93.8 %
Phase Resolution	100 %

Physio - PACE

Resp. Control	Off
Concatenations	2

Inline - Liver

Liver Registration	Off
Save Original Images	On

Inline - Subtraction

Subtract	Off
Measurements	1
StdDev	Off
Save Original Images	On

Inline - MIP

MIP Sag	Off
MIP Cor	Off
MIP Tra	Off

Inline - MIP

MIP Time	Off
Radial MIP	Off
Save Original Images	On
MPR Sag	Off
MPR Cor	Off
MPR Tra	Off

Inline - Composing

Inline Composing	Off

Inline - MapIt

MapIt	None
Flip Angle	180 deg
Measurements	1
Contrasts	16
TE 1	10.70 ms
TE 2	21.40 ms
TE 3	32.10 ms
TE 4	42.80 ms
TE 5	53.50 ms
TE 6	64.20 ms
TE 7	74.90 ms
TE 8	85.60 ms
TE 9	96.30 ms
TE 10	107.00 ms
TE 11	117.70 ms
TE 12	128.40 ms
TE 13	139.10 ms
TE 14	149.80 ms
TE 15	160.50 ms
TE 16	171.20 ms
TR	3910.0 ms
Save Original Images	On

Sequence - Part 1

Sequence Name	WIP_se
RF Pulse Type	Low SAR
Gradient Mode	Fast
Bandwidth	219 Hz/Px

Sequence - Part 2

Introduction	On

Sequence - Special

Undersampling Factor	5
R2 Images	On
M0 Images	On
Interpolate	On
Filter Background	On
Regularization:	5.00
Virtual Channels	4
Simulated TE 0	83 ms
Simulated TE 1	0 ms
Simulated TE 2	0 ms

Sequence - Special

Simulated TE 3	0 ms
Simulated TE 4	0 ms

Sequence - Assistant

SAR Assistant	Off
Allowed Delay	0 s

\\USER\2023_MBC_ VIDA PROTOCOLS_XA50\SB.2020.353 AEP study - 64 Channel\SB.2020.353 - AEP MRI ACQUISITION PROTOCOL v23_Vida- 64 channel\T2w_AxOblTempPole_RL- *

TA: 5:38 min Coil Selection: Auto Voxel Size: 0.3×0.3×0.5 mm³ Acc:: 2 Rel. SNR: 1.00

Properties

Start measurement without further preparation	On
Wait for User to Start	Off
Start measurements	Single Measurement
Prio Recon	Off
Auto Open Inline Display	Off
Auto Close Inline Display	Off
Load Images to MR View&GO	On
Auto Store Images	On
Disable auto transfer to PACS	Off
Load Images to Stamp Segments	Off
Load Images to Graphic Segments	Off
Graphic segment	Default
Inline Movie	Off

Routine

Slab Group	1
Slabs	1
Position	Isocenter
Orientation	T > C-20.0
Phase Encoding Dir.	R >> L
Slices per Slab	72
Phase Oversampling	20 %
Slice Oversampling	22.2 %
FOV Read	160 mm
FOV Phase	100.0 %
Slice Thickness	0.50 mm
TR	1400.0 ms
TE	156.00 ms
Averages	2.0
Concatenations	1
AutoAlign	---

Contrast - Common

TR	1400.0 ms
TE	156.00 ms
MTC	Off
Magn. Preparation	None
Flip Angle Mode	Constant
Flip Angle	120 deg
Fat-Water Contrast	Standard
Dark Blood	Off
Blood Suppression	Off
Wrap-up Magn.	Restore
Reconstruction	Magnitude

Contrast - Dynamic

Dynamic Mode	Standard
Measurements	1

Contrast - Dynamic

Multiple Series	Each Measurement
Reordering	Linear

Resolution - Common

FOV Read	160 mm
FOV Phase	100.0 %
Slice Thickness	0.50 mm
Base Resolution	320
Phase Resolution	100 %
Slice Resolution	84 %
Interpolation	On

Resolution - Acceleration

Acceleration Mode	GRAPPA
Total Factor	2
Reference Scans	Integrated
Acceleration Factor PE	2
Reference Lines PE	24
Acceleration Factor 3D	1
Phase Partial Fourier	Allowed
Slice Partial Fourier	6/8
Elliptical Scanning	Off

Resolution - Filter

Raw Filter	On
Elliptical Filter	Off
Distortion Correction	2D
Normalize	Prescan
Image Filter	Off

Geometry - Common

Slab Group	1
Slabs	1
Position	Isocenter
Orientation	T > C-20.0
Phase Encoding Dir.	R >> L
Slices per Slab	72
Phase Oversampling	20 %
Slice Oversampling	22.2 %
FOV Read	160 mm
FOV Phase	100.0 %
Slice Thickness	0.50 mm
TR	1400.0 ms
Concatenations	1

Geometry - AutoAlign

Slab Group	1
Position	Isocenter
Orientation	T > C-20.0

Geometry - AutoAlign

Phase Encoding Dir.	R >> L
AutoAlign	---
Initial Position	Isocenter
L	0.0 mm
P	0.0 mm
H	0.0 mm
Initial Orientation	T > C
T > C	-20.00
> S	0.00
Initial Rotation	90.00 deg

Geometry - Navigator

Geometry - Saturation

Special Saturation	None

Geometry - Tim Planning Suite

Set-n-Go Protocol	Off
Table Position	0 mm
Table Position	H
Inline Composing	Off

System - Miscellaneous

Coil Selection	ACS All but spine
Radial Sorting	Off
MSMA	S - C - T
Sagittal	R >> L
Coronal	A >> P
Transversal	F >> H
Coil Combination	Adaptive Combine
Matrix Optimization	Off
Coil Focus	Flat

System - Adjustments

Adjustment Strategy	Standard
B0 Shim	Standard
B1 Shim	TrueForm
CoilShim	Off
Adjustment Tolerance	Auto
Adjust with Body Coil	Off
Confirm Frequency	Never
Assume Silicone	Off

System - Adjust Volume

Position	Isocenter
Orientation	T > C-20.0
Rotation	90.00 deg
R >> L	160 mm
A >> P	160 mm
F >> H	36 mm
Reset	Off

System - pTx

B1 Shim	TrueForm

System - pTx

Excitation	Slab-sel.

System - Tx/Rx

Frequency 1H	123.213354 MHz
? Ref. Amplitude 1H	0.000 V
Reset	Off
Image Scaling	1.000
Gain	High

Physio - Signal

1st Signal/Mode	None
Trigger Delay	0 ms
TR	1400.0 ms
Concatenations	1

Physio - Cardiac

Fat-Water Contrast	Standard
Magn. Preparation	None
Dark Blood	Off
FOV Read	160 mm
FOV Phase	100.0 %
Phase Resolution	100 %

Physio - PACE

Resp. Control	Off
Concatenations	1

Inline - Subtraction

Subtract	Off
Measurements	1
StdDev	Off
Save Original Images	On

Inline - MIP

MIP Sag	Off
MIP Cor	Off
MIP Tra	Off
MIP Time	Off
Radial MIP	Off
Save Original Images	On
MPR Sag	Off
MPR Cor	Off
MPR Tra	Off

Inline - Composing

Inline Composing	Off

Sequence - Part 1

Sequence Name	spcR
Dimension	3D
Excitation	Slab-sel.
RF Pulse Type	Normal
Gradient Mode	Normal
Flow Compensation	None

Sequence - Part 1

Reordering	Linear
Bandwidth	289 Hz/Px
Echo Spacing	6.88 ms
Turbo Factor	96
Echo Train Duration	482 ms

Sequence - Part 2

Introduction	On

Sequence - Assistant

SAR Assistant	Off
Allowed Delay	0 s

\\USER\2023_MBC_ VIDA PROTOCOLS_XA50\SB.2020.353 AEP study - 64 Channel\SB.2020.353 - AEP MRI ACQUISITION PROTOCOL v23_Vida- 64 channel\DWI_Ax_AP_52Dir_AP- *

TA: 3:14 min Coil Selection: Auto Voxel Size: 2.0×2.0×2.0 mm³ Acc:: None Rel. SNR: 1.00

Properties

Start measurement without further preparation	On
Wait for User to Start	Off
Start measurements	Single Measurement
Prio Recon	Off
Auto Open Inline Display	Off
Auto Close Inline Display	Off
Load Images to MR View&GO	On
Auto Store Images	On
Disable auto transfer to PACS	Off
Load Images to Stamp Segments	Off
Load Images to Graphic Segments	Off
Graphic segment	Default
Inline Movie	Off

Routine

Slice Group	1
Slices	76
Distance Factor	0 %
Position	Isocenter
Orientation	Transversal
Phase Encoding Dir.	A >> P
Phase Oversampling	0 %
FOV Read	232 mm
FOV Phase	100.0 %
Slice Thickness	2.0 mm
TR	3345.0 ms
TE	113.00 ms
Multi-band accel. factor	4
AutoAlign	---

Contrast - Common

TR	3345.0 ms
TE	113.00 ms
MTC	Off
Magn. Preparation	None
Flip Angle	90 deg
Refocus flip angle	180 deg
Fat-Water Contrast	Standard
Grad. rev. fat suppr.	Enabled
Reconstruction	Magnitude

Contrast - Dynamic

Dynamic Mode	Standard
Multiple Series	Off
Delay in TR	0.00 ms

Resolution - Common

FOV Read	232 mm
FOV Phase	100.0 %
Slice Thickness	2.0 mm
Base Resolution	116
Phase Resolution	100 %
Interpolation	Off

Resolution - Acceleration

Acceleration Mode	None
Phase Partial Fourier	6/8

Resolution - Filter

Raw Filter	Off
Elliptical Filter	Off
Distortion Correction	Off
Static Field Correction	Off
Normalize	Prescan
Noise Masking	Off

Geometry - Common

Slice Group	1
Slices	76
Distance Factor	0 %
Position	Isocenter
Orientation	Transversal
Phase Encoding Dir.	A >> P
Phase Oversampling	0 %
FOV Read	232 mm
FOV Phase	100.0 %
Slice Thickness	2.0 mm
TR	3345.0 ms
Multi-Slice Mode	Interleaved
Series	Interleaved
Multi-band accel. factor	4

Geometry - AutoAlign

Slice Group	1
Position	Isocenter
Orientation	Transversal
Phase Encoding Dir.	A >> P
AutoAlign	---
Initial Position	Isocenter
L	0.0 mm
P	0.0 mm
H	0.0 mm
Initial Orientation	Transversal
Initial Rotation	0.00 deg

Geometry - Navigator

Geometry - Saturation

Special Saturation	None

Geometry - Tim Planning Suite

Set-n-Go Protocol	Off
Table Position	0 mm
Table Position	H
Inline Composing	Off

System - Miscellaneous

Coil Selection	Auto Coil Select
Radial Sorting	Off
MSMA	S - C - T
Sagittal	R >> L
Coronal	A >> P
Transversal	F >> H
Coil Combination	Sum of Squares
Matrix Optimization	Off
Coil Focus	Flat

System - Adjustments

Adjustment Strategy	Standard
B0 Shim	Standard
B1 Shim	TrueForm
CoilShim	Off
Adjustment Tolerance	Auto
Adjust with Body Coil	Off
Confirm Frequency	Never
Assume Silicone	Off

System - Adjust Volume

Position	Isocenter
Orientation	Transversal
Rotation	0.00 deg
A >> P	232 mm
R >> L	232 mm
F >> H	152 mm
Reset	Off

System - pTx

B1 Shim	TrueForm
Excitation	Standard

System - Tx/Rx

Frequency 1H	123.213354 MHz
? Ref. Amplitude 1H	0.000 V
Reset	Off
Image Scaling	1.000

Physio - Signal

1st Signal/Mode	None
TR	3345.0 ms
Multi-band accel. factor	4

Physio - PACE

Resp. Control	Off
Multi-band accel. factor	4

Diff

Diffusion Mode	Free
Diff. Directions	52
Diffusion Scheme	Monopolar
Diff. Weightings	2
b-value 1	0 s/mm²
b-value 2	3000 s/mm²
Averages 1	1
Averages 2	1
Dynamic Field Correction	Off
Invert Gray Scale	Off
Diff. Weighted Images	On
Trace Weighted Images	Off
Tensor	Off
FA Maps	Off
ADC Maps	On
Exponential ADC Maps	Off
ADC Noise Threshold	40
Noise Masking	Off
Calculated Image	Off

Sequence - Part 1

Sequence Name	epse
Dimension	2D
Excitation	Standard
Gradient Mode	Performance
Bandwidth	1724 Hz/Px
Echo Spacing	0.68 ms
Free Echo Spacing	Off
EPI Factor	116

Sequence - Part 2

Introduction	Off
RF Spoiling	Off
Phase Correction	Internal

Sequence - Special

Excite pulse duration	5120 us
Refocus pulse duration	10240 us
Min. prep scans	0
Min. prep scans SB	0
Delay before PC scans	0 us
Single-band images	On
MB LeakBlock kernel	On
MB dual kernel	Off
MB RF phase scramble	On
Opt. MB RF pulse BW	Off
Time-shifted MB RF	Off
SENSE1 coil combine	On
Invert RO/PE polarity	Off
PF omits higher k-space	Off

Sequence - Special

Force GPA balance	On
Disable B1 control loop	Off
Disable freq. update	Off
Suppress 16-bit DICOM	Off
Force equal slice timing	Off
Online multi-band recon.	Online
FFT scale factor	1.00
Physio recording	DICOM

Sequence - Assistant

SAR Assistant	Off

\\USER\2023_MBC_ VIDA PROTOCOLS_XA50\SB.2020.353 AEP study - 64 Channel\SB.2020.353 - AEP MRI ACQUISITION PROTOCOL v23_Vida- 64 channel\DWI_Ax_AP_52Dir_APinvROPE- *

TA: 3:11 min Coil Selection: Auto Voxel Size: 2.0×2.0×2.0 mm³ Acc:: None Rel. SNR: 1.00

Properties

Start measurement without further preparation	On
Wait for User to Start	Off
Start measurements	Single Measurement
Prio Recon	Off
Auto Open Inline Display	Off
Auto Close Inline Display	Off
Load Images to MR View&GO	On
Auto Store Images	On
Disable auto transfer to PACS	Off
Load Images to Stamp Segments	Off
Load Images to Graphic Segments	Off
Graphic segment	Default
Inline Movie	Off

Routine

Slice Group	1
Slices	76
Distance Factor	0 %
Position	Isocenter
Orientation	Transversal
Phase Encoding Dir.	A >> P
Phase Oversampling	0 %
FOV Read	232 mm
FOV Phase	100.0 %
Slice Thickness	2.0 mm
TR	3345.0 ms
TE	113.00 ms
Multi-band accel. factor	4
AutoAlign	---

Contrast - Common

TR	3345.0 ms
TE	113.00 ms
MTC	Off
Magn. Preparation	None
Flip Angle	90 deg
Refocus flip angle	180 deg
Fat-Water Contrast	Standard
Grad. rev. fat suppr.	Enabled
Reconstruction	Magnitude

Contrast - Dynamic

Dynamic Mode	Standard
Multiple Series	Off
Delay in TR	0.00 ms

Resolution - Common

FOV Read	232 mm

Resolution - Common

FOV Phase	100.0 %
Slice Thickness	2.0 mm
Base Resolution	116
Phase Resolution	100 %
Interpolation	Off

Resolution - Acceleration

Acceleration Mode	None
Phase Partial Fourier	6/8

Resolution - Filter

Raw Filter	Off
Elliptical Filter	Off
Distortion Correction	Off
Static Field Correction	Off
Normalize	Prescan
Noise Masking	Off

Geometry - Common

Slice Group	1
Slices	76
Distance Factor	0 %
Position	Isocenter
Orientation	Transversal
Phase Encoding Dir.	A >> P
Phase Oversampling	0 %
FOV Read	232 mm
FOV Phase	100.0 %
Slice Thickness	2.0 mm
TR	3345.0 ms
Multi-Slice Mode	Interleaved
Series	Interleaved
Multi-band accel. factor	4

Geometry - AutoAlign

Slice Group	1
Position	Isocenter
Orientation	Transversal
Phase Encoding Dir.	A >> P
AutoAlign	---
Initial Position	Isocenter
L	0.0 mm
P	0.0 mm
H	0.0 mm
Initial Orientation	Transversal
Initial Rotation	0.00 deg

Geometry - Navigator

Geometry - Saturation

Special Saturation	None

Geometry - Tim Planning Suite

Set-n-Go Protocol	Off
Table Position	0 mm
Table Position	H
Inline Composing	Off

System - Miscellaneous

Coil Selection	Auto Coil Select
Radial Sorting	Off
MSMA	S - C - T
Sagittal	R >> L
Coronal	A >> P
Transversal	F >> H
Coil Combination	Sum of Squares
Matrix Optimization	Off
Coil Focus	Flat

System - Adjustments

Adjustment Strategy	Standard
B0 Shim	Standard
B1 Shim	TrueForm
CoilShim	Off
Adjustment Tolerance	Auto
Adjust with Body Coil	Off
Confirm Frequency	Never
Assume Silicone	Off

System - Adjust Volume

Position	Isocenter
Orientation	Transversal
Rotation	0.00 deg
A >> P	232 mm
R >> L	232 mm
F >> H	152 mm
Reset	Off

System - pTx

B1 Shim	TrueForm
Excitation	Standard

System - Tx/Rx

Frequency 1H	123.213354 MHz
? Ref. Amplitude 1H	0.000 V
Reset	Off
Image Scaling	1.000

Physio - Signal

1st Signal/Mode	None
TR	3345.0 ms
Multi-band accel. factor	4

Physio - PACE

Resp. Control	Off
Multi-band accel. factor	4

Diff

Diffusion Mode	Free
Diff. Directions	51
Diffusion Scheme	Monopolar
Diff. Weightings	2
b-value 1	0 s/mm²
b-value 2	3000 s/mm²
Averages 1	1
Averages 2	1
Dynamic Field Correction	Off
Invert Gray Scale	Off
Diff. Weighted Images	On
Trace Weighted Images	Off
Tensor	Off
FA Maps	Off
ADC Maps	On
Exponential ADC Maps	Off
ADC Noise Threshold	40
Noise Masking	Off
Calculated Image	Off

Sequence - Part 1

Sequence Name	epse
Dimension	2D
Excitation	Standard
Gradient Mode	Performance
Bandwidth	1724 Hz/Px
Echo Spacing	0.68 ms
Free Echo Spacing	Off
EPI Factor	116

Sequence - Part 2

Introduction	Off
RF Spoiling	Off
Phase Correction	Internal

Sequence - Special

Excite pulse duration	5120 us
Refocus pulse duration	10240 us
Min. prep scans	0
Min. prep scans SB	0
Delay before PC scans	0 us
Single-band images	On
MB LeakBlock kernel	On
MB dual kernel	Off
MB RF phase scramble	On
Opt. MB RF pulse BW	Off
Time-shifted MB RF	Off
SENSE1 coil combine	On
Invert RO/PE polarity	On
PF omits higher k-space	Off

Sequence - Special

Force GPA balance	On
Disable B1 control loop	Off
Disable freq. update	Off
Suppress 16-bit DICOM	Off
Force equal slice timing	Off
Online multi-band recon.	Online
FFT scale factor	1.00
Physio recording	DICOM

Sequence - Assistant

SAR Assistant	Off

\\USER\2023_MBC_ VIDA PROTOCOLS_XA50\SB.2020.353 AEP study - 64 Channel\SB.2020.353 - AEP MRI ACQUISITION PROTOCOL v23_Vida- 64 channel\T1map_MP2RAGE_Sag_AP- *

TA: 3:42 min Coil Selection: Auto Voxel Size: 1.0×1.0×1.0 mm³ Acc:: None Rel. SNR: 1.00

Properties

Start measurement without further preparation	On
Wait for User to Start	Off
Start measurements	Single Measurement
Prio Recon	Off
Auto Open Inline Display	Off
Auto Close Inline Display	Off
Load Images to MR View&GO	On
Auto Store Images	On
Disable auto transfer to PACS	Off
Load Images to Stamp Segments	Off
Load Images to Graphic Segments	Off
Graphic segment	Default
Inline Movie	Off

Routine

Slab Group	1
Slabs	1
Distance Factor	50 %
Position	Isocenter
Orientation	Sagittal
Phase Encoding Dir.	A >> P
Slices per Slab	208
Phase Oversampling	0 %
Slice Oversampling	0.0 %
FOV Read	256 mm
FOV Phase	93.8 %
Slice Thickness	1.00 mm
TR	5000.0 ms
TE	2.88 ms
Averages	1
Concatenations	1
AutoAlign	---

Contrast - Common

TR	5000.0 ms
TE	2.88 ms
Magn. Preparation	Non-sel. IR
TI 1	700 ms
TI 2	2500 ms
Flip Angle 1	4 deg
Flip Angle 2	5 deg
Fat-Water Contrast	Standard
Dark Blood	Off
Contrasts	1
Reconstruction	Magnitude

Contrast - Dynamic

Dynamic Mode	Standard

Contrast - Dynamic

Measurements	1
Multiple Series	Each Measurement
Reordering	Linear

Resolution - Common

FOV Read	256 mm
FOV Phase	93.8 %
Slice Thickness	1.00 mm
Base Resolution	256
Phase Resolution	100 %
Slice Resolution	100 %
Interpolation	Off

Resolution - Acceleration

Acceleration Mode	None
Reference Scans	GRE/Separate
Reference Lines PE	32
Phase Partial Fourier	Off
Slice Partial Fourier	Off
Asymmetric Echo	Off
Elliptical Scanning	Off

Resolution - Filter

Raw Filter	Off
Elliptical Filter	Off
Distortion Correction	3D
Normalize	Prescan
Image Filter	Off

Geometry - Common

Slab Group	1
Slabs	1
Distance Factor	50 %
Position	Isocenter
Orientation	Sagittal
Phase Encoding Dir.	A >> P
Slices per Slab	208
Phase Oversampling	0 %
Slice Oversampling	0.0 %
FOV Read	256 mm
FOV Phase	93.8 %
Slice Thickness	1.00 mm
TR	5000.0 ms
Multi-Slice Mode	Single Shot
Series	Interleaved
Concatenations	1

Geometry - AutoAlign

Slab Group	1

Geometry - AutoAlign

Position	Isocenter
Orientation	Sagittal
Phase Encoding Dir.	A >> P
AutoAlign	---
Initial Position	Isocenter
L	0.0 mm
P	0.0 mm
H	0.0 mm
Initial Orientation	Sagittal
Initial Rotation	0.00 deg

Geometry - Navigator

Geometry - Tim Planning Suite

Set-n-Go Protocol	Off
Table Position	0 mm
Table Position	H
Inline Composing	Off

System - Miscellaneous

Coil Selection	Auto Coil Select
Radial Sorting	Off
MSMA	S - C - T
Sagittal	R >> L
Coronal	A >> P
Transversal	F >> H
Coil Combination	Adaptive Combine
Matrix Optimization	Performance
Coil Focus	Flat

System - Adjustments

Adjustment Strategy	Standard
B0 Shim	Standard
B1 Shim	TrueForm
CoilShim	Off
Adjustment Tolerance	Auto
Adjust with Body Coil	Off
Confirm Frequency	Never
Assume Silicone	Off

System - Adjust Volume

Position	Isocenter
Orientation	Sagittal
Rotation	0.00 deg
A >> P	240 mm
F >> H	256 mm
R >> L	208 mm
Reset	Off

System - pTx

B1 Shim	TrueForm
Excitation	Non-sel.

System - Tx/Rx

Frequency 1H	123.213354 MHz
? Ref. Amplitude 1H	0.000 V
Reset	Off
Image Scaling	1.000

Physio - Signal

1st Signal/Mode	None
TR	5000.0 ms
Concatenations	1

Physio - Cardiac

Fat-Water Contrast	Standard
Magn. Preparation	Non-sel. IR
TI 1	700 ms
TI 2	2500 ms
Dark Blood	Off
FOV Read	256 mm
FOV Phase	93.8 %
Phase Resolution	100 %

Physio - PACE

Resp. Control	Off
Concatenations	1

Inline - Subtraction

Subtract	Off
Measurements	1
StdDev	Off
Save Original Images	On

Inline - MIP

MIP Sag	Off
MIP Cor	Off
MIP Tra	Off
MIP Time	Off
Radial MIP	Off
Save Original Images	On
MPR Sag	Off
MPR Cor	Off
MPR Tra	Off

Inline - Composing

Inline Composing	Off

Inline - MapIt

MapIt	T1 Map
Flip Angle 1	4 deg
Flip Angle 2	5 deg
Measurements	1
Contrasts	1
TE	2.88 ms
TR	5000.0 ms
Save Original Images	On

Sequence - Part 1

Sequence Name	WIP_tfl
Dimension	3D
Excitation	Non-sel.
RF Pulse Type	Fast
Gradient Mode	Fast
Flow Compensation	None
Reordering	Linear
Bandwidth	240 Hz/Px
Echo Spacing	6.82 ms
Asymmetric Echo	Off
Turbo Factor	190

Sequence - Part 2

Introduction	Off
RF Spoiling	On
Incr. Gradient Spoiling	Off

Sequence - Special

Sparse Undersampling	On
Sampling Pattern	Cartesian Phyllotaxis
Undersampling Factor	4.6 x
Density	0.50
Jitter Radius	1.2
Diff. Mask per INV	Off
Regularisation INV1	0.00100
Regularisation INV2	0.00040
No. Iterations	20
CSM RO Resolution	0
DL Recon	Default
Synthetic TI1	410 ms
Synthetic TI2	1100 ms
Uniform Img.	On
Denoised UNI	On
FLAWS-min	Off
FLAWS-hc	Off
FLAWS-hco	Off
Div. Image	Off
UNIDenoised Weight	1.0

Sequence - Assistant

SAR Assistant	Off

\\USER\2023_MBC_ VIDA PROTOCOLS_XA50\SB.2020.353 AEP study - 64 Channel\SB.2020.353 - AEP MRI ACQUISITION PROTOCOL v23_Vida- 64 channel\fMRI_AxObl_AP_Pseudoword- *

TA: 3:20 min Coil Selection: Auto Voxel Size: 3.0×3.0×3.0 mm³ Acc:: 2 Rel. SNR: 1.00

Properties

Start measurement without further preparation	On
Wait for User to Start	Off
Start measurements	Single Measurement
Prio Recon	Off
Auto Open Inline Display	Off
Auto Close Inline Display	Off
Load Images to MR View&GO	On
Auto Store Images	On
Disable auto transfer to PACS	Off
Load Images to Stamp Segments	Off
Load Images to Graphic Segments	Off
Graphic segment	Default
Inline Movie	Off

Routine

Slice Group	1
Slices	44
Distance Factor	0 %
Position	Isocenter
Orientation	T > C-30.0
Phase Encoding Dir.	A >> P
Phase Oversampling	0 %
FOV Read	216 mm
FOV Phase	111.1 %
Slice Thickness	3.0 mm
TR	900.0 ms
TE 1	13.40 ms
TE 2	32.96 ms
TE 3	52.52 ms
Averages	1
Multi-band accel. factor	4
AutoAlign	---

Contrast - Common

TR	900.0 ms
TE 1	13.40 ms
TE 2	32.96 ms
TE 3	52.52 ms
MTC	Off
Magn. Preparation	None
Flip Angle	30 deg
Fat-Water Contrast	Fat Saturation
Contrasts	3
Reconstruction	Magn./Phase

Contrast - Dynamic

Dynamic Mode	Standard
Measurements	202

Contrast - Dynamic

Delay in TR	0.00 ms

Resolution - Common

FOV Read	216 mm
FOV Phase	111.1 %
Slice Thickness	3.0 mm
Base Resolution	72
Phase Resolution	100 %
Interpolation	Off

Resolution - Acceleration

Acceleration Mode	GRAPPA
Reference scan mode	Single-shot
Acceleration Factor PE	2
Reference Lines PE	24
Phase Partial Fourier	7/8

Resolution - Filter

Raw Filter	Off
Elliptical Filter	Off
Hamming	Off
Distortion Correction	Off
Static Field Correction	Off
Normalize	Prescan

Geometry - Common

Slice Group	1
Slices	44
Distance Factor	0 %
Position	Isocenter
Orientation	T > C-30.0
Phase Encoding Dir.	A >> P
Phase Oversampling	0 %
FOV Read	216 mm
FOV Phase	111.1 %
Slice Thickness	3.0 mm
TR	900.0 ms
Multi-Slice Mode	Interleaved
Series	Interleaved
Multi-band accel. factor	4

Geometry - AutoAlign

Slice Group	1
Position	Isocenter
Orientation	T > C-30.0
Phase Encoding Dir.	A >> P
AutoAlign	---
Initial Position	Isocenter
L	0.0 mm

Geometry - AutoAlign

P	0.0 mm
H	0.0 mm
Initial Orientation	T > C
T > C	-30.00
> S	0.00
Initial Rotation	0.00 deg

Geometry - Saturation

Special Saturation	None

Geometry - Tim Planning Suite

Set-n-Go Protocol	Off
Table Position	0 mm
Table Position	H
Inline Composing	Off

System - Miscellaneous

Coil Selection	Auto Coil Select
Radial Sorting	Off
MSMA	S - C - T
Sagittal	R >> L
Coronal	A >> P
Transversal	F >> H
Coil Combination	Sum of Squares
Matrix Optimization	Off
Coil Focus	Flat

System - Adjustments

Adjustment Strategy	Standard
B0 Shim	Standard
B1 Shim	TrueForm
CoilShim	Off
Adjustment Tolerance	Auto
Adjust with Body Coil	Off
Confirm Frequency	Never
Assume Silicone	Off

System - Adjust Volume

Position	Isocenter
Orientation	T > C-30.0
Rotation	90.00 deg
R >> L	216 mm
A >> P	240 mm
F >> H	132 mm
Reset	Off

System - pTx

B1 Shim	TrueForm
Excitation	Standard

System - Tx/Rx

Frequency 1H	123.213354 MHz
? Ref. Amplitude 1H	0.000 V
Reset	Off

System - Tx/Rx

Image Scaling	1.000

Physio - Signal

1st Signal/Mode	None
TR	900.0 ms
Log Signals	Off
Multi-band accel. factor	4

BOLD

GLM Statistics	Off
Ignore Meas. at Start	0
Ignore After Transition	0
Model Transition States	On
Temp. Highpass Filter	On
Threshold	4.00
Paradigm Size	201
Meas[1]	Baseline
Meas[2]	Baseline
Meas[3]	Baseline
Meas[4]	Baseline
Meas[5]	Baseline
Meas[6]	Baseline
Meas[7]	Baseline
Meas[8]	Baseline
Meas[9]	Baseline
Meas[10]	Baseline
Meas[11]	Active
Meas[12]	Active
Meas[13]	Active
Meas[14]	Active
Meas[15]	Active
Meas[16]	Active
Meas[17]	Active
Meas[18]	Active
Meas[19]	Active
Meas[20]	Active
Meas[21]	Active
Meas[22]	Active
Meas[23]	Active
Meas[24]	Active
Meas[25]	Active
Meas[26]	Active
Meas[27]	Active
Meas[28]	Active
Meas[29]	Active
Meas[30]	Active
Meas[31]	Baseline
Meas[32]	Baseline
Meas[33]	Baseline
Meas[34]	Baseline
Meas[35]	Baseline
Meas[36]	Baseline
Meas[37]	Baseline
Meas[38]	Baseline

BOLD

Meas[39]	Baseline
Meas[40]	Baseline
Meas[41]	Baseline
Meas[42]	Baseline
Meas[43]	Baseline
Meas[44]	Baseline
Meas[45]	Baseline
Meas[46]	Baseline
Meas[47]	Baseline
Meas[48]	Baseline
Meas[49]	Baseline
Meas[50]	Baseline
Meas[51]	Active
Meas[52]	Active
Meas[53]	Active
Meas[54]	Active
Meas[55]	Active
Meas[56]	Active
Meas[57]	Active
Meas[58]	Active
Meas[59]	Active
Meas[60]	Active
Meas[61]	Active
Meas[62]	Active
Meas[63]	Active
Meas[64]	Active
Meas[65]	Active
Meas[66]	Active
Meas[67]	Active
Meas[68]	Active
Meas[69]	Active
Meas[70]	Active
Meas[71]	Baseline
Meas[72]	Baseline
Meas[73]	Baseline
Meas[74]	Baseline
Meas[75]	Baseline
Meas[76]	Baseline
Meas[77]	Baseline
Meas[78]	Baseline
Meas[79]	Baseline
Meas[80]	Baseline
Meas[81]	Baseline
Meas[82]	Baseline
Meas[83]	Baseline
Meas[84]	Baseline
Meas[85]	Baseline
Meas[86]	Baseline
Meas[87]	Baseline
Meas[88]	Baseline
Meas[89]	Baseline
Meas[90]	Baseline
Meas[91]	Active
Meas[92]	Active

BOLD

Meas[93]	Active
Meas[94]	Active
Meas[95]	Active
Meas[96]	Active
Meas[97]	Active
Meas[98]	Active
Meas[99]	Active
Meas[100]	Active
Meas[101]	Active
Meas[102]	Active
Meas[103]	Active
Meas[104]	Active
Meas[105]	Active
Meas[106]	Active
Meas[107]	Active
Meas[108]	Active
Meas[109]	Active
Meas[110]	Active
Meas[111]	Baseline
Meas[112]	Baseline
Meas[113]	Baseline
Meas[114]	Baseline
Meas[115]	Baseline
Meas[116]	Baseline
Meas[117]	Baseline
Meas[118]	Baseline
Meas[119]	Baseline
Meas[120]	Baseline
Meas[121]	Baseline
Meas[122]	Baseline
Meas[123]	Baseline
Meas[124]	Baseline
Meas[125]	Baseline
Meas[126]	Baseline
Meas[127]	Baseline
Meas[128]	Baseline
Meas[129]	Baseline
Meas[130]	Baseline
Meas[131]	Active
Meas[132]	Active
Meas[133]	Active
Meas[134]	Active
Meas[135]	Active
Meas[136]	Active
Meas[137]	Active
Meas[138]	Active
Meas[139]	Active
Meas[140]	Active
Meas[141]	Active
Meas[142]	Active
Meas[143]	Active
Meas[144]	Active
Meas[145]	Active
Meas[146]	Active

BOLD

Meas[147]	Active
Meas[148]	Active
Meas[149]	Active
Meas[150]	Active
Meas[151]	Baseline
Meas[152]	Baseline
Meas[153]	Baseline
Meas[154]	Baseline
Meas[155]	Baseline
Meas[156]	Baseline
Meas[157]	Baseline
Meas[158]	Baseline
Meas[159]	Baseline
Meas[160]	Baseline
Meas[161]	Baseline
Meas[162]	Baseline
Meas[163]	Baseline
Meas[164]	Baseline
Meas[165]	Baseline
Meas[166]	Baseline
Meas[167]	Baseline
Meas[168]	Baseline
Meas[169]	Baseline
Meas[170]	Baseline
Meas[171]	Active
Meas[172]	Active
Meas[173]	Active
Meas[174]	Active
Meas[175]	Active
Meas[176]	Active
Meas[177]	Active
Meas[178]	Active
Meas[179]	Active
Meas[180]	Active
Meas[181]	Active
Meas[182]	Active
Meas[183]	Active
Meas[184]	Active
Meas[185]	Active
Meas[186]	Active
Meas[187]	Active
Meas[188]	Active
Meas[189]	Active
Meas[190]	Active
Meas[191]	Baseline
Meas[192]	Baseline
Meas[193]	Baseline
Meas[194]	Baseline
Meas[195]	Baseline
Meas[196]	Baseline
Meas[197]	Baseline
Meas[198]	Baseline
Meas[199]	Baseline
Meas[200]	Baseline

BOLD

Meas[201]	Baseline
Motion Correction	Off
Spatial Filter	Off
Measurements	202
Delay in TR	0.00 ms

Sequence - Part 1

Sequence Name	epfid
Dimension	2D
Excitation	Standard
Gradient Mode	Performance
Flow Compensation	None
Bandwidth	2314 Hz/Px
Echo Spacing	0.54 ms
Free Echo Spacing	Off
EPI Factor	80

Sequence - Part 2

Introduction	Off
RF Spoiling	Off

Sequence - Special

Excite pulse duration	5120 us
Min. prep scans	0
Min. prep scans SB	0
Inter-TE delay	0 us
Delay before PC scans	0 us
Single-band images	On
MB LeakBlock kernel	On
MB dual kernel	Off
MB RF phase scramble	Off
Opt. MB RF pulse BW	Off
SENSE1 coil combine	On
Invert RO/PE polarity	Off
Force Maxwell corr.	Off
PF omits higher k-space	Off
Disable B1 control loop	Off
Disable freq. update	Off
Suppress 16-bit DICOM	Off
Force equal slice timing	Off
Online multi-band recon.	Online
FFT scale factor	1.00
Fat saturation FA	110.00 deg
Fat sat. offset	0.00 Hz
Sinc exc. pulse BWTP	5.20
Physio recording	DICOM
Triggering scheme	Standard

Sequence - Assistant

SAR Assistant	Off

\\USER\2023_MBC_ VIDA PROTOCOLS_XA50\SB.2020.353 AEP study - 64 Channel\SB.2020.353 - AEP MRI ACQUISITION PROTOCOL v23_Vida- 64 channel\FMap_AxObl_AP- *

TA: 10 sec Coil Selection: Auto Voxel Size: 2.0×2.0×2.0 mm³ Acc:: None Rel. SNR: 1.00

Properties

Start measurement without further preparation	On
Wait for User to Start	Off
Start measurements	Single Measurement
Prio Recon	Off
Auto Open Inline Display	Off
Auto Close Inline Display	Off
Load Images to MR View&GO	On
Auto Store Images	On
Disable auto transfer to PACS	Off
Load Images to Stamp Segments	Off
Load Images to Graphic Segments	Off
Graphic segment	Default
Inline Movie	Off

Routine

Slice Group	1
Slices	72
Distance Factor	0 %
Position	Isocenter
Orientation	T > C-30.0
Phase Encoding Dir.	A >> P
Phase Oversampling	0 %
FOV Read	216 mm
FOV Phase	111.1 %
Slice Thickness	2.0 mm
TR	10100.0 ms
TE	84.00 ms
Averages	1
Multi-band accel. factor	1
AutoAlign	---

Contrast - Common

TR	10100.0 ms
TE	84.00 ms
MTC	Off
Magn. Preparation	None
Flip Angle	90 deg
Refocus flip angle	180 deg
Fat-Water Contrast	Fat Saturation
Grad. rev. fat suppr.	Disabled
Contrasts	1
Reconstruction	Magnitude

Contrast - Dynamic

Dynamic Mode	Standard
Measurements	1
Delay in TR	0.00 ms

Resolution - Common

FOV Read	216 mm
FOV Phase	111.1 %
Slice Thickness	2.0 mm
Base Resolution	108
Phase Resolution	100 %
Interpolation	Off

Resolution - Acceleration

Acceleration Mode	None
Phase Partial Fourier	Off

Resolution - Filter

Raw Filter	Off
Elliptical Filter	Off
Hamming	Off
Distortion Correction	Off
Static Field Correction	Off
Normalize	Prescan

Geometry - Common

Slice Group	1
Slices	72
Distance Factor	0 %
Position	Isocenter
Orientation	T > C-30.0
Phase Encoding Dir.	A >> P
Phase Oversampling	0 %
FOV Read	216 mm
FOV Phase	111.1 %
Slice Thickness	2.0 mm
TR	10100.0 ms
Multi-Slice Mode	Interleaved
Series	Interleaved
Multi-band accel. factor	1

Geometry - AutoAlign

Slice Group	1
Position	Isocenter
Orientation	T > C-30.0
Phase Encoding Dir.	A >> P
AutoAlign	---
Initial Position	Isocenter
L	0.0 mm
P	0.0 mm
H	0.0 mm
Initial Orientation	T > C
T > C	-30.00
> S	0.00
Initial Rotation	0.00 deg

Geometry - Saturation

Special Saturation	None

Geometry - Tim Planning Suite

Set-n-Go Protocol	Off
Table Position	0 mm
Table Position	H
Inline Composing	Off

System - Miscellaneous

Coil Selection	Auto Coil Select
Radial Sorting	Off
MSMA	S - C - T
Sagittal	R >> L
Coronal	A >> P
Transversal	F >> H
Coil Combination	Sum of Squares
Matrix Optimization	Off
Coil Focus	Flat

System - Adjustments

Adjustment Strategy	Standard
B0 Shim	Standard
B1 Shim	TrueForm
CoilShim	Off
Adjustment Tolerance	Auto
Adjust with Body Coil	Off
Confirm Frequency	Never
Assume Silicone	Off

System - Adjust Volume

Position	Isocenter
Orientation	T > C-30.0
Rotation	90.00 deg
R >> L	216 mm
A >> P	240 mm
F >> H	144 mm
Reset	Off

System - pTx

B1 Shim	TrueForm
Excitation	Standard

System - Tx/Rx

Frequency 1H	123.213354 MHz
? Ref. Amplitude 1H	0.000 V
Reset	Off
Image Scaling	1.000

Physio - Signal

1st Signal/Mode	None
TR	10100.0 ms
Log Signals	Off
Multi-band accel. factor	1

BOLD

GLM Statistics	Off
Ignore Meas. at Start	0
Ignore After Transition	0
Model Transition States	On
Temp. Highpass Filter	On
Threshold	4.00
Paradigm Size	4
Meas[1]	Baseline
Meas[2]	Baseline
Meas[3]	Baseline
Meas[4]	Active
Motion Correction	Off
Spatial Filter	Off
Measurements	1
Delay in TR	0.00 ms

Sequence - Part 1

Sequence Name	epse
Dimension	2D
Excitation	Standard
RF Pulse Type	Normal
Gradient Mode	Performance
Bandwidth	2104 Hz/Px
Echo Spacing	0.61 ms
Free Echo Spacing	Off
EPI Factor	120

Sequence - Part 2

Introduction	Off

Sequence - Special

Min. prep scans	0
Delay before PC scans	0 us
SENSE1 coil combine	On
Invert RO/PE polarity	Off
Force GPA balance	On
Disable B1 control loop	Off
Disable freq. update	Off
Suppress 16-bit DICOM	Off
Force equal slice timing	Off
FFT scale factor	1.00
Fat saturation FA	110.00 deg
Fat sat. offset	0.00 Hz
Physio recording	DICOM
Triggering scheme	Standard

Sequence - Assistant

SAR Assistant	Off

\\USER\2023_MBC_ VIDA PROTOCOLS_XA50\SB.2020.353 AEP study - 64 Channel\SB.2020.353 - AEP MRI ACQUISITION PROTOCOL v23_Vida- 64 channel\FMap_AxObl_AP-InvROPE- *

TA: 10 sec Coil Selection: Auto Voxel Size: 2.0×2.0×2.0 mm³ Acc:: None Rel. SNR: 1.00

Properties

Start measurement without further preparation	On
Wait for User to Start	Off
Start measurements	Single Measurement
Prio Recon	Off
Auto Open Inline Display	Off
Auto Close Inline Display	Off
Load Images to MR View&GO	On
Auto Store Images	On
Disable auto transfer to PACS	Off
Load Images to Stamp Segments	Off
Load Images to Graphic Segments	Off
Graphic segment	Default
Inline Movie	Off

Routine

Slice Group	1
Slices	72
Distance Factor	0 %
Position	Isocenter
Orientation	T > C-30.0
Phase Encoding Dir.	A >> P
Phase Oversampling	0 %
FOV Read	216 mm
FOV Phase	111.1 %
Slice Thickness	2.0 mm
TR	10100.0 ms
TE	84.00 ms
Averages	1
Multi-band accel. factor	1
AutoAlign	---

Contrast - Common

TR	10100.0 ms
TE	84.00 ms
MTC	Off
Magn. Preparation	None
Flip Angle	90 deg
Refocus flip angle	180 deg
Fat-Water Contrast	Fat Saturation
Grad. rev. fat suppr.	Disabled
Contrasts	1
Reconstruction	Magnitude

Contrast - Dynamic

Dynamic Mode	Standard
Measurements	1
Delay in TR	0.00 ms

Resolution - Common

FOV Read	216 mm
FOV Phase	111.1 %
Slice Thickness	2.0 mm
Base Resolution	108
Phase Resolution	100 %
Interpolation	Off

Resolution - Acceleration

Acceleration Mode	None
Phase Partial Fourier	Off

Resolution - Filter

Raw Filter	Off
Elliptical Filter	Off
Hamming	Off
Distortion Correction	Off
Static Field Correction	Off
Normalize	Prescan

Geometry - Common

Slice Group	1
Slices	72
Distance Factor	0 %
Position	Isocenter
Orientation	T > C-30.0
Phase Encoding Dir.	A >> P
Phase Oversampling	0 %
FOV Read	216 mm
FOV Phase	111.1 %
Slice Thickness	2.0 mm
TR	10100.0 ms
Multi-Slice Mode	Interleaved
Series	Interleaved
Multi-band accel. factor	1

Geometry - AutoAlign

Slice Group	1
Position	Isocenter
Orientation	T > C-30.0
Phase Encoding Dir.	A >> P
AutoAlign	---
Initial Position	Isocenter
L	0.0 mm
P	0.0 mm
H	0.0 mm
Initial Orientation	T > C
T > C	-30.00
> S	0.00
Initial Rotation	0.00 deg

Geometry - Saturation

Special Saturation	None

Geometry - Tim Planning Suite

Set-n-Go Protocol	Off
Table Position	0 mm
Table Position	H
Inline Composing	Off

System - Miscellaneous

Coil Selection	Auto Coil Select
Radial Sorting	Off
MSMA	S - C - T
Sagittal	R >> L
Coronal	A >> P
Transversal	F >> H
Coil Combination	Sum of Squares
Matrix Optimization	Off
Coil Focus	Flat

System - Adjustments

Adjustment Strategy	Standard
B0 Shim	Standard
B1 Shim	TrueForm
CoilShim	Off
Adjustment Tolerance	Auto
Adjust with Body Coil	Off
Confirm Frequency	Never
Assume Silicone	Off

System - Adjust Volume

Position	Isocenter
Orientation	T > C-30.0
Rotation	90.00 deg
R >> L	216 mm
A >> P	240 mm
F >> H	144 mm
Reset	Off

System - pTx

B1 Shim	TrueForm
Excitation	Standard

System - Tx/Rx

Frequency 1H	123.213354 MHz
? Ref. Amplitude 1H	0.000 V
Reset	Off
Image Scaling	1.000

Physio - Signal

1st Signal/Mode	None
TR	10100.0 ms
Log Signals	Off
Multi-band accel. factor	1

BOLD

GLM Statistics	Off
Ignore Meas. at Start	0
Ignore After Transition	0
Model Transition States	On
Temp. Highpass Filter	On
Threshold	4.00
Paradigm Size	4
Meas[1]	Baseline
Meas[2]	Baseline
Meas[3]	Baseline
Meas[4]	Active
Motion Correction	Off
Spatial Filter	Off
Measurements	1
Delay in TR	0.00 ms

Sequence - Part 1

Sequence Name	epse
Dimension	2D
Excitation	Standard
RF Pulse Type	Normal
Gradient Mode	Performance
Bandwidth	2104 Hz/Px
Echo Spacing	0.61 ms
Free Echo Spacing	Off
EPI Factor	120

Sequence - Part 2

Introduction	Off

Sequence - Special

Min. prep scans	0
Delay before PC scans	0 us
SENSE1 coil combine	On
Invert RO/PE polarity	On
Force GPA balance	On
Disable B1 control loop	Off
Disable freq. update	Off
Suppress 16-bit DICOM	Off
Force equal slice timing	Off
FFT scale factor	1.00
Fat saturation FA	110.00 deg
Fat sat. offset	0.00 Hz
Physio recording	DICOM
Triggering scheme	Standard

Sequence - Assistant

SAR Assistant	Off

\\USER\2023_MBC_ VIDA PROTOCOLS_XA50\SB.2020.353 AEP study - 64 Channel\SB.2020.353 - AEP MRI ACQUISITION PROTOCOL v23_Vida- 64 channel\fMRI_AxObl_AP_OneBack- *

TA: 4:53 min Coil Selection: Auto Voxel Size: 3.0×3.0×3.0 mm^3 Acc:: 2 Rel. SNR: 1.00

Properties

Start measurement without further preparation	On
Wait for User to Start	Off
Start measurements	Single Measurement
Prio Recon	Off
Auto Open Inline Display	Off
Auto Close Inline Display	Off
Load Images to MR View&GO	On
Auto Store Images	On
Disable auto transfer to PACS	Off
Load Images to Stamp Segments	Off
Load Images to Graphic Segments	Off
Graphic segment	Default
Inline Movie	Off

Routine

Slice Group	1
Slices	44
Distance Factor	0 %
Position	Isocenter
Orientation	T > C-30.0
Phase Encoding Dir.	A >> P
Phase Oversampling	0 %
FOV Read	216 mm
FOV Phase	111.1 %
Slice Thickness	3.0 mm
TR	900.0 ms
TE 1	13.40 ms
TE 2	32.96 ms
TE 3	52.52 ms
Averages	1
Multi-band accel. factor	4
AutoAlign	---

Contrast - Common

TR	900.0 ms
TE 1	13.40 ms
TE 2	32.96 ms
TE 3	52.52 ms
MTC	Off
Magn. Preparation	None
Flip Angle	30 deg
Fat-Water Contrast	Fat Saturation
Contrasts	3
Reconstruction	Magn./Phase

Contrast - Dynamic

Dynamic Mode	Standard
Measurements	305

Contrast - Dynamic

Delay in TR	0.00 ms

Resolution - Common

FOV Read	216 mm
FOV Phase	111.1 %
Slice Thickness	3.0 mm
Base Resolution	72
Phase Resolution	100 %
Interpolation	Off

Resolution - Acceleration

Acceleration Mode	GRAPPA
Reference scan mode	Single-shot
Acceleration Factor PE	2
Reference Lines PE	24
Phase Partial Fourier	7/8

Resolution - Filter

Raw Filter	Off
Elliptical Filter	Off
Hamming	Off
Distortion Correction	Off
Static Field Correction	Off
Normalize	Prescan

Geometry - Common

Slice Group	1
Slices	44
Distance Factor	0 %
Position	Isocenter
Orientation	T > C-30.0
Phase Encoding Dir.	A >> P
Phase Oversampling	0 %
FOV Read	216 mm
FOV Phase	111.1 %
Slice Thickness	3.0 mm
TR	900.0 ms
Multi-Slice Mode	Interleaved
Series	Interleaved
Multi-band accel. factor	4

Geometry - AutoAlign

Slice Group	1
Position	Isocenter
Orientation	T > C-30.0
Phase Encoding Dir.	A >> P
AutoAlign	---
Initial Position	Isocenter
L	0.0 mm

Geometry - AutoAlign

P	0.0 mm
H	0.0 mm
Initial Orientation	T > C
T > C	-30.00
> S	0.00
Initial Rotation	0.00 deg

Geometry - Saturation

Special Saturation	None

Geometry - Tim Planning Suite

Set-n-Go Protocol	Off
Table Position	0 mm
Table Position	H
Inline Composing	Off

System - Miscellaneous

Coil Selection	Auto Coil Select
Radial Sorting	Off
MSMA	S - C - T
Sagittal	R >> L
Coronal	A >> P
Transversal	F >> H
Coil Combination	Sum of Squares
Matrix Optimization	Off
Coil Focus	Flat

System - Adjustments

Adjustment Strategy	Standard
B0 Shim	Standard
B1 Shim	TrueForm
CoilShim	Off
Adjustment Tolerance	Auto
Adjust with Body Coil	Off
Confirm Frequency	Never
Assume Silicone	Off

System - Adjust Volume

Position	Isocenter
Orientation	T > C-30.0
Rotation	90.00 deg
R >> L	216 mm
A >> P	240 mm
F >> H	132 mm
Reset	Off

System - pTx

B1 Shim	TrueForm
Excitation	Standard

System - Tx/Rx

Frequency 1H	123.213354 MHz
? Ref. Amplitude 1H	0.000 V
Reset	Off

System - Tx/Rx

Image Scaling	1.000

Physio - Signal

1st Signal/Mode	None
TR	900.0 ms
Log Signals	Off
Multi-band accel. factor	4

BOLD

GLM Statistics	Off
Ignore Meas. at Start	0
Ignore After Transition	0
Model Transition States	On
Temp. Highpass Filter	On
Threshold	4.00
Paradigm Size	305
Meas[1]	Baseline
Meas[2]	Baseline
Meas[3]	Baseline
Meas[4]	Baseline
Meas[5]	Baseline
Meas[6]	Baseline
Meas[7]	Baseline
Meas[8]	Baseline
Meas[9]	Baseline
Meas[10]	Baseline
Meas[11]	Baseline
Meas[12]	Baseline
Meas[13]	Baseline
Meas[14]	Baseline
Meas[15]	Baseline
Meas[16]	Baseline
Meas[17]	Baseline
Meas[18]	Active
Meas[19]	Active
Meas[20]	Active
Meas[21]	Active
Meas[22]	Active
Meas[23]	Active
Meas[24]	Active
Meas[25]	Active
Meas[26]	Active
Meas[27]	Active
Meas[28]	Active
Meas[29]	Active
Meas[30]	Active
Meas[31]	Active
Meas[32]	Active
Meas[33]	Active
Meas[34]	Active
Meas[35]	Active
Meas[36]	Active
Meas[37]	Active
Meas[38]	Active

BOLD

Meas[39]	Active
Meas[40]	Active
Meas[41]	Active
Meas[42]	Active
Meas[43]	Active
Meas[44]	Active
Meas[45]	Active
Meas[46]	Active
Meas[47]	Active
Meas[48]	Active
Meas[49]	Active
Meas[50]	Active
Meas[51]	Active
Meas[52]	Active
Meas[53]	Active
Meas[54]	Active
Meas[55]	Active
Meas[56]	Active
Meas[57]	Active
Meas[58]	Active
Meas[59]	Active
Meas[60]	Active
Meas[61]	Active
Meas[62]	Active
Meas[63]	Active
Meas[64]	Active
Meas[65]	Active
Meas[66]	Active
Meas[67]	Active
Meas[68]	Active
Meas[69]	Active
Meas[70]	Active
Meas[71]	Active
Meas[72]	Active
Meas[73]	Baseline
Meas[74]	Baseline
Meas[75]	Baseline
Meas[76]	Baseline
Meas[77]	Baseline
Meas[78]	Baseline
Meas[79]	Baseline
Meas[80]	Baseline
Meas[81]	Baseline
Meas[82]	Baseline
Meas[83]	Baseline
Meas[84]	Baseline
Meas[85]	Baseline
Meas[86]	Baseline
Meas[87]	Baseline
Meas[88]	Baseline
Meas[89]	Baseline
Meas[90]	Active
Meas[91]	Active
Meas[92]	Active

BOLD

Meas[93]	Active
Meas[94]	Active
Meas[95]	Active
Meas[96]	Active
Meas[97]	Active
Meas[98]	Active
Meas[99]	Active
Meas[100]	Active
Meas[101]	Active
Meas[102]	Active
Meas[103]	Active
Meas[104]	Active
Meas[105]	Active
Meas[106]	Active
Meas[107]	Active
Meas[108]	Active
Meas[109]	Active
Meas[110]	Active
Meas[111]	Active
Meas[112]	Active
Meas[113]	Active
Meas[114]	Active
Meas[115]	Active
Meas[116]	Active
Meas[117]	Active
Meas[118]	Active
Meas[119]	Active
Meas[120]	Active
Meas[121]	Active
Meas[122]	Active
Meas[123]	Active
Meas[124]	Active
Meas[125]	Active
Meas[126]	Active
Meas[127]	Active
Meas[128]	Active
Meas[129]	Active
Meas[130]	Active
Meas[131]	Active
Meas[132]	Active
Meas[133]	Active
Meas[134]	Active
Meas[135]	Active
Meas[136]	Active
Meas[137]	Active
Meas[138]	Active
Meas[139]	Active
Meas[140]	Active
Meas[141]	Active
Meas[142]	Active
Meas[143]	Active
Meas[144]	Active
Meas[145]	Baseline
Meas[146]	Baseline

BOLD

Meas[147]	Baseline
Meas[148]	Baseline
Meas[149]	Baseline
Meas[150]	Baseline
Meas[151]	Baseline
Meas[152]	Baseline
Meas[153]	Baseline
Meas[154]	Baseline
Meas[155]	Baseline
Meas[156]	Baseline
Meas[157]	Baseline
Meas[158]	Baseline
Meas[159]	Baseline
Meas[160]	Baseline
Meas[161]	Baseline
Meas[162]	Active
Meas[163]	Active
Meas[164]	Active
Meas[165]	Active
Meas[166]	Active
Meas[167]	Active
Meas[168]	Active
Meas[169]	Active
Meas[170]	Active
Meas[171]	Active
Meas[172]	Active
Meas[173]	Active
Meas[174]	Active
Meas[175]	Active
Meas[176]	Active
Meas[177]	Active
Meas[178]	Active
Meas[179]	Active
Meas[180]	Active
Meas[181]	Active
Meas[182]	Active
Meas[183]	Active
Meas[184]	Active
Meas[185]	Active
Meas[186]	Active
Meas[187]	Active
Meas[188]	Active
Meas[189]	Active
Meas[190]	Active
Meas[191]	Active
Meas[192]	Active
Meas[193]	Active
Meas[194]	Active
Meas[195]	Active
Meas[196]	Active
Meas[197]	Active
Meas[198]	Active
Meas[199]	Active
Meas[200]	Active

BOLD

Meas[201]	Active
Meas[202]	Active
Meas[203]	Active
Meas[204]	Active
Meas[205]	Active
Meas[206]	Active
Meas[207]	Active
Meas[208]	Active
Meas[209]	Active
Meas[210]	Active
Meas[211]	Active
Meas[212]	Active
Meas[213]	Active
Meas[214]	Active
Meas[215]	Active
Meas[216]	Active
Meas[217]	Baseline
Meas[218]	Baseline
Meas[219]	Baseline
Meas[220]	Baseline
Meas[221]	Baseline
Meas[222]	Baseline
Meas[223]	Baseline
Meas[224]	Baseline
Meas[225]	Baseline
Meas[226]	Baseline
Meas[227]	Baseline
Meas[228]	Baseline
Meas[229]	Baseline
Meas[230]	Baseline
Meas[231]	Baseline
Meas[232]	Baseline
Meas[233]	Baseline
Meas[234]	Active
Meas[235]	Active
Meas[236]	Active
Meas[237]	Active
Meas[238]	Active
Meas[239]	Active
Meas[240]	Active
Meas[241]	Active
Meas[242]	Active
Meas[243]	Active
Meas[244]	Active
Meas[245]	Active
Meas[246]	Active
Meas[247]	Active
Meas[248]	Active
Meas[249]	Active
Meas[250]	Active
Meas[251]	Active
Meas[252]	Active
Meas[253]	Active
Meas[254]	Active

BOLD

Meas[255]	Active
Meas[256]	Active
Meas[257]	Active
Meas[258]	Active
Meas[259]	Active
Meas[260]	Active
Meas[261]	Active
Meas[262]	Active
Meas[263]	Active
Meas[264]	Active
Meas[265]	Active
Meas[266]	Active
Meas[267]	Active
Meas[268]	Active
Meas[269]	Active
Meas[270]	Active
Meas[271]	Active
Meas[272]	Active
Meas[273]	Active
Meas[274]	Active
Meas[275]	Active
Meas[276]	Active
Meas[277]	Active
Meas[278]	Active
Meas[279]	Active
Meas[280]	Active
Meas[281]	Active
Meas[282]	Active
Meas[283]	Active
Meas[284]	Active
Meas[285]	Active
Meas[286]	Active
Meas[287]	Active
Meas[288]	Active
Meas[289]	Baseline
Meas[290]	Baseline
Meas[291]	Baseline
Meas[292]	Baseline
Meas[293]	Baseline
Meas[294]	Baseline
Meas[295]	Baseline
Meas[296]	Baseline
Meas[297]	Baseline
Meas[298]	Baseline
Meas[299]	Baseline
Meas[300]	Baseline
Meas[301]	Baseline
Meas[302]	Baseline
Meas[303]	Baseline
Meas[304]	Baseline
Meas[305]	Baseline
Motion Correction	Off
Spatial Filter	Off
Measurements	305

BOLD

Delay in TR	0.00 ms

Sequence - Part 1

Sequence Name	epfid
Dimension	2D
Excitation	Standard
Gradient Mode	Performance
Flow Compensation	None
Bandwidth	2314 Hz/Px
Echo Spacing	0.54 ms
Free Echo Spacing	Off
EPI Factor	80

Sequence - Part 2

Introduction	Off
RF Spoiling	Off

Sequence - Special

Excite pulse duration	5120 us
Min. prep scans	0
Min. prep scans SB	0
Inter-TE delay	0 us
Delay before PC scans	0 us
Single-band images	On
MB LeakBlock kernel	On
MB dual kernel	Off
MB RF phase scramble	Off
Opt. MB RF pulse BW	Off
SENSE1 coil combine	On
Invert RO/PE polarity	Off
Force Maxwell corr.	Off
PF omits higher k-space	Off
Disable B1 control loop	Off
Disable freq. update	Off
Suppress 16-bit DICOM	Off
Force equal slice timing	Off
Online multi-band recon.	Online
FFT scale factor	1.00
Fat saturation FA	110.00 deg
Fat sat. offset	0.00 Hz
Sinc exc. pulse BWTP	5.20
Physio recording	DICOM
Triggering scheme	Standard

Sequence - Assistant

SAR Assistant	Off

\\USER\2023_MBC_ VIDA PROTOCOLS_XA50\SB.2020.353 AEP study - 64 Channel\SB.2020.353 - AEP MRI ACQUISITION PROTOCOL v23_Vida- 64 channel\FMap_AxObl_AP- *

TA: 10 sec Coil Selection: Auto Voxel Size: 2.0×2.0×2.0 mm³ Acc:: None Rel. SNR: 1.00

Properties

Start measurement without further preparation	On
Wait for User to Start	Off
Start measurements	Single Measurement
Prio Recon	Off
Auto Open Inline Display	Off
Auto Close Inline Display	Off
Load Images to MR View&GO	On
Auto Store Images	On
Disable auto transfer to PACS	Off
Load Images to Stamp Segments	Off
Load Images to Graphic Segments	Off
Graphic segment	Default
Inline Movie	Off

Routine

Slice Group	1
Slices	72
Distance Factor	0 %
Position	Isocenter
Orientation	T > C-30.0
Phase Encoding Dir.	A >> P
Phase Oversampling	0 %
FOV Read	216 mm
FOV Phase	111.1 %
Slice Thickness	2.0 mm
TR	10100.0 ms
TE	84.00 ms
Averages	1
Multi-band accel. factor	1
AutoAlign	---

Contrast - Common

TR	10100.0 ms
TE	84.00 ms
MTC	Off
Magn. Preparation	None
Flip Angle	90 deg
Refocus flip angle	180 deg
Fat-Water Contrast	Fat Saturation
Grad. rev. fat suppr.	Disabled
Contrasts	1
Reconstruction	Magnitude

Contrast - Dynamic

Dynamic Mode	Standard
Measurements	1
Delay in TR	0.00 ms

Resolution - Common

FOV Read	216 mm
FOV Phase	111.1 %
Slice Thickness	2.0 mm
Base Resolution	108
Phase Resolution	100 %
Interpolation	Off

Resolution - Acceleration

Acceleration Mode	None
Phase Partial Fourier	Off

Resolution - Filter

Raw Filter	Off
Elliptical Filter	Off
Hamming	Off
Distortion Correction	Off
Static Field Correction	Off
Normalize	Prescan

Geometry - Common

Slice Group	1
Slices	72
Distance Factor	0 %
Position	Isocenter
Orientation	T > C-30.0
Phase Encoding Dir.	A >> P
Phase Oversampling	0 %
FOV Read	216 mm
FOV Phase	111.1 %
Slice Thickness	2.0 mm
TR	10100.0 ms
Multi-Slice Mode	Interleaved
Series	Interleaved
Multi-band accel. factor	1

Geometry - AutoAlign

Slice Group	1
Position	Isocenter
Orientation	T > C-30.0
Phase Encoding Dir.	A >> P
AutoAlign	---
Initial Position	Isocenter
L	0.0 mm
P	0.0 mm
H	0.0 mm
Initial Orientation	T > C
T > C	-30.00
> S	0.00
Initial Rotation	0.00 deg

Geometry - Saturation

Special Saturation	None

Geometry - Tim Planning Suite

Set-n-Go Protocol	Off
Table Position	0 mm
Table Position	H
Inline Composing	Off

System - Miscellaneous

Coil Selection	Auto Coil Select
Radial Sorting	Off
MSMA	S - C - T
Sagittal	R >> L
Coronal	A >> P
Transversal	F >> H
Coil Combination	Sum of Squares
Matrix Optimization	Off
Coil Focus	Flat

System - Adjustments

Adjustment Strategy	Standard
B0 Shim	Standard
B1 Shim	TrueForm
CoilShim	Off
Adjustment Tolerance	Auto
Adjust with Body Coil	Off
Confirm Frequency	Never
Assume Silicone	Off

System - Adjust Volume

Position	Isocenter
Orientation	T > C-30.0
Rotation	90.00 deg
R >> L	216 mm
A >> P	240 mm
F >> H	144 mm
Reset	Off

System - pTx

B1 Shim	TrueForm
Excitation	Standard

System - Tx/Rx

Frequency 1H	123.213354 MHz
? Ref. Amplitude 1H	0.000 V
Reset	Off
Image Scaling	1.000

Physio - Signal

1st Signal/Mode	None
TR	10100.0 ms
Log Signals	Off
Multi-band accel. factor	1

BOLD

GLM Statistics	Off
Ignore Meas. at Start	0
Ignore After Transition	0
Model Transition States	On
Temp. Highpass Filter	On
Threshold	4.00
Paradigm Size	4
Meas[1]	Baseline
Meas[2]	Baseline
Meas[3]	Baseline
Meas[4]	Active
Motion Correction	Off
Spatial Filter	Off
Measurements	1
Delay in TR	0.00 ms

Sequence - Part 1

Sequence Name	epse
Dimension	2D
Excitation	Standard
RF Pulse Type	Normal
Gradient Mode	Performance
Bandwidth	2104 Hz/Px
Echo Spacing	0.61 ms
Free Echo Spacing	Off
EPI Factor	120

Sequence - Part 2

Introduction	Off

Sequence - Special

Min. prep scans	0
Delay before PC scans	0 us
SENSE1 coil combine	On
Invert RO/PE polarity	Off
Force GPA balance	On
Disable B1 control loop	Off
Disable freq. update	Off
Suppress 16-bit DICOM	Off
Force equal slice timing	Off
FFT scale factor	1.00
Fat saturation FA	110.00 deg
Fat sat. offset	0.00 Hz
Physio recording	DICOM
Triggering scheme	Standard

Sequence - Assistant

SAR Assistant	Off

\\USER\2023_MBC_ VIDA PROTOCOLS_XA50\SB.2020.353 AEP study - 64 Channel\SB.2020.353 - AEP MRI ACQUISITION PROTOCOL v23_Vida- 64 channel\FMap_AxObl_AP-InvROPE- *

TA: 10 sec Coil Selection: Auto Voxel Size: 2.0×2.0×2.0 mm³ Acc:: None Rel. SNR: 1.00

Properties

Start measurement without further preparation	On
Wait for User to Start	Off
Start measurements	Single Measurement
Prio Recon	Off
Auto Open Inline Display	Off
Auto Close Inline Display	Off
Load Images to MR View&GO	On
Auto Store Images	On
Disable auto transfer to PACS	Off
Load Images to Stamp Segments	Off
Load Images to Graphic Segments	Off
Graphic segment	Default
Inline Movie	Off

Routine

Slice Group	1
Slices	72
Distance Factor	0 %
Position	Isocenter
Orientation	T > C-30.0
Phase Encoding Dir.	A >> P
Phase Oversampling	0 %
FOV Read	216 mm
FOV Phase	111.1 %
Slice Thickness	2.0 mm
TR	10100.0 ms
TE	84.00 ms
Averages	1
Multi-band accel. factor	1
AutoAlign	---

Contrast - Common

TR	10100.0 ms
TE	84.00 ms
MTC	Off
Magn. Preparation	None
Flip Angle	90 deg
Refocus flip angle	180 deg
Fat-Water Contrast	Fat Saturation
Grad. rev. fat suppr.	Disabled
Contrasts	1
Reconstruction	Magnitude

Contrast - Dynamic

Dynamic Mode	Standard
Measurements	1
Delay in TR	0.00 ms

Resolution - Common

FOV Read	216 mm
FOV Phase	111.1 %
Slice Thickness	2.0 mm
Base Resolution	108
Phase Resolution	100 %
Interpolation	Off

Resolution - Acceleration

Acceleration Mode	None
Phase Partial Fourier	Off

Resolution - Filter

Raw Filter	Off
Elliptical Filter	Off
Hamming	Off
Distortion Correction	Off
Static Field Correction	Off
Normalize	Prescan

Geometry - Common

Slice Group	1
Slices	72
Distance Factor	0 %
Position	Isocenter
Orientation	T > C-30.0
Phase Encoding Dir.	A >> P
Phase Oversampling	0 %
FOV Read	216 mm
FOV Phase	111.1 %
Slice Thickness	2.0 mm
TR	10100.0 ms
Multi-Slice Mode	Interleaved
Series	Interleaved
Multi-band accel. factor	1

Geometry - AutoAlign

Slice Group	1
Position	Isocenter
Orientation	T > C-30.0
Phase Encoding Dir.	A >> P
AutoAlign	---
Initial Position	Isocenter
L	0.0 mm
P	0.0 mm
H	0.0 mm
Initial Orientation	T > C
T > C	-30.00
> S	0.00
Initial Rotation	0.00 deg

Geometry - Saturation

Special Saturation	None

Geometry - Tim Planning Suite

Set-n-Go Protocol	Off
Table Position	0 mm
Table Position	H
Inline Composing	Off

System - Miscellaneous

Coil Selection	Auto Coil Select
Radial Sorting	Off
MSMA	S - C - T
Sagittal	R >> L
Coronal	A >> P
Transversal	F >> H
Coil Combination	Sum of Squares
Matrix Optimization	Off
Coil Focus	Flat

System - Adjustments

Adjustment Strategy	Standard
B0 Shim	Standard
B1 Shim	TrueForm
CoilShim	Off
Adjustment Tolerance	Auto
Adjust with Body Coil	Off
Confirm Frequency	Never
Assume Silicone	Off

System - Adjust Volume

Position	Isocenter
Orientation	T > C-30.0
Rotation	90.00 deg
R >> L	216 mm
A >> P	240 mm
F >> H	144 mm
Reset	Off

System - pTx

B1 Shim	TrueForm
Excitation	Standard

System - Tx/Rx

Frequency 1H	123.213354 MHz
? Ref. Amplitude 1H	0.000 V
Reset	Off
Image Scaling	1.000

Physio - Signal

1st Signal/Mode	None
TR	10100.0 ms
Log Signals	Off
Multi-band accel. factor	1

BOLD

GLM Statistics	Off
Ignore Meas. at Start	0
Ignore After Transition	0
Model Transition States	On
Temp. Highpass Filter	On
Threshold	4.00
Paradigm Size	4
Meas[1]	Baseline
Meas[2]	Baseline
Meas[3]	Baseline
Meas[4]	Active
Motion Correction	Off
Spatial Filter	Off
Measurements	1
Delay in TR	0.00 ms

Sequence - Part 1

Sequence Name	epse
Dimension	2D
Excitation	Standard
RF Pulse Type	Normal
Gradient Mode	Performance
Bandwidth	2104 Hz/Px
Echo Spacing	0.61 ms
Free Echo Spacing	Off
EPI Factor	120

Sequence - Part 2

Introduction	Off

Sequence - Special

Min. prep scans	0
Delay before PC scans	0 us
SENSE1 coil combine	On
Invert RO/PE polarity	On
Force GPA balance	On
Disable B1 control loop	Off
Disable freq. update	Off
Suppress 16-bit DICOM	Off
Force equal slice timing	Off
FFT scale factor	1.00
Fat saturation FA	110.00 deg
Fat sat. offset	0.00 Hz
Physio recording	DICOM
Triggering scheme	Standard

Sequence - Assistant

SAR Assistant	Off

\\USER\2023_MBC_ VIDA PROTOCOLS_XA50\SB.2020.353 AEP study - 64 Channel\SB.2020.353 - AEP MRI ACQUISITION PROTOCOL v23_Vida- 64 channel\fMRI_AxObl_AP_Movie- *

TA: 13:54 min Coil Selection: Auto Voxel Size: 3.0×3.0×3.0 mm³ Acc:: 2 Rel. SNR: 1.00

Properties

Start measurement without further preparation	On
Wait for User to Start	Off
Start measurements	Single Measurement
Prio Recon	Off
Auto Open Inline Display	Off
Auto Close Inline Display	Off
Load Images to MR View&GO	On
Auto Store Images	On
Disable auto transfer to PACS	Off
Load Images to Stamp Segments	Off
Load Images to Graphic Segments	Off
Graphic segment	Default
Inline Movie	Off

Routine

Slice Group	1
Slices	44
Distance Factor	0 %
Position	Isocenter
Orientation	T > C-30.0
Phase Encoding Dir.	A >> P
Phase Oversampling	0 %
FOV Read	216 mm
FOV Phase	111.1 %
Slice Thickness	3.0 mm
TR	900.0 ms
TE 1	13.40 ms
TE 2	32.96 ms
TE 3	52.52 ms
Averages	1
Multi-band accel. factor	4
AutoAlign	---

Contrast - Common

TR	900.0 ms
TE 1	13.40 ms
TE 2	32.96 ms
TE 3	52.52 ms
MTC	Off
Magn. Preparation	None
Flip Angle	30 deg
Fat-Water Contrast	Fat Saturation
Contrasts	3
Reconstruction	Magn./Phase

Contrast - Dynamic

Dynamic Mode	Standard
Measurements	907

Contrast - Dynamic

Delay in TR	0.00 ms

Resolution - Common

FOV Read	216 mm
FOV Phase	111.1 %
Slice Thickness	3.0 mm
Base Resolution	72
Phase Resolution	100 %
Interpolation	Off

Resolution - Acceleration

Acceleration Mode	GRAPPA
Reference scan mode	Single-shot
Acceleration Factor PE	2
Reference Lines PE	24
Phase Partial Fourier	7/8

Resolution - Filter

Raw Filter	Off
Elliptical Filter	Off
Hamming	Off
Distortion Correction	Off
Static Field Correction	Off
Normalize	Prescan

Geometry - Common

Slice Group	1
Slices	44
Distance Factor	0 %
Position	Isocenter
Orientation	T > C-30.0
Phase Encoding Dir.	A >> P
Phase Oversampling	0 %
FOV Read	216 mm
FOV Phase	111.1 %
Slice Thickness	3.0 mm
TR	900.0 ms
Multi-Slice Mode	Interleaved
Series	Interleaved
Multi-band accel. factor	4

Geometry - AutoAlign

Slice Group	1
Position	Isocenter
Orientation	T > C-30.0
Phase Encoding Dir.	A >> P
AutoAlign	---
Initial Position	Isocenter
L	0.0 mm

Geometry - AutoAlign

P	0.0 mm
H	0.0 mm
Initial Orientation	T > C
T > C	-30.00
> S	0.00
Initial Rotation	0.00 deg

Geometry - Saturation

Special Saturation	None

Geometry - Tim Planning Suite

Set-n-Go Protocol	Off
Table Position	0 mm
Table Position	H
Inline Composing	Off

System - Miscellaneous

Coil Selection	Auto Coil Select
Radial Sorting	Off
MSMA	S - C - T
Sagittal	R >> L
Coronal	A >> P
Transversal	F >> H
Coil Combination	Sum of Squares
Matrix Optimization	Off
Coil Focus	Flat

System - Adjustments

Adjustment Strategy	Standard
B0 Shim	Standard
B1 Shim	TrueForm
CoilShim	Off
Adjustment Tolerance	Auto
Adjust with Body Coil	Off
Confirm Frequency	Never
Assume Silicone	Off

System - Adjust Volume

Position	Isocenter
Orientation	T > C-30.0
Rotation	90.00 deg
R >> L	216 mm
A >> P	240 mm
F >> H	132 mm
Reset	Off

System - pTx

B1 Shim	TrueForm
Excitation	Standard

System - Tx/Rx

Frequency 1H	123.213354 MHz
? Ref. Amplitude 1H	0.000 V
Reset	Off

System - Tx/Rx

Image Scaling	1.000

Physio - Signal

1st Signal/Mode	None
TR	900.0 ms
Log Signals	Off
Multi-band accel. factor	4

BOLD

GLM Statistics	Off
Ignore Meas. at Start	0
Ignore After Transition	0
Model Transition States	On
Temp. Highpass Filter	On
Threshold	4.00
Paradigm Size	80
Meas[1]	Baseline
Meas[2]	Baseline
Meas[3]	Baseline
Meas[4]	Baseline
Meas[5]	Baseline
Meas[6]	Baseline
Meas[7]	Baseline
Meas[8]	Baseline
Meas[9]	Baseline
Meas[10]	Baseline
Meas[11]	Baseline
Meas[12]	Baseline
Meas[13]	Baseline
Meas[14]	Baseline
Meas[15]	Baseline
Meas[16]	Baseline
Meas[17]	Baseline
Meas[18]	Baseline
Meas[19]	Baseline
Meas[20]	Baseline
Meas[21]	Baseline
Meas[22]	Baseline
Meas[23]	Baseline
Meas[24]	Baseline
Meas[25]	Baseline
Meas[26]	Baseline
Meas[27]	Baseline
Meas[28]	Baseline
Meas[29]	Baseline
Meas[30]	Baseline
Meas[31]	Baseline
Meas[32]	Baseline
Meas[33]	Baseline
Meas[34]	Baseline
Meas[35]	Baseline
Meas[36]	Baseline
Meas[37]	Baseline
Meas[38]	Baseline

BOLD

Meas[39]	Baseline
Meas[40]	Baseline
Meas[41]	Active
Meas[42]	Active
Meas[43]	Active
Meas[44]	Active
Meas[45]	Active
Meas[46]	Active
Meas[47]	Active
Meas[48]	Active
Meas[49]	Active
Meas[50]	Active
Meas[51]	Active
Meas[52]	Active
Meas[53]	Active
Meas[54]	Active
Meas[55]	Active
Meas[56]	Active
Meas[57]	Active
Meas[58]	Active
Meas[59]	Active
Meas[60]	Active
Meas[61]	Active
Meas[62]	Active
Meas[63]	Active
Meas[64]	Active
Meas[65]	Active
Meas[66]	Active
Meas[67]	Active
Meas[68]	Active
Meas[69]	Active
Meas[70]	Active
Meas[71]	Active
Meas[72]	Active
Meas[73]	Active
Meas[74]	Active
Meas[75]	Active
Meas[76]	Active
Meas[77]	Active
Meas[78]	Active
Meas[79]	Active
Meas[80]	Active
Motion Correction	Off
Spatial Filter	Off
Measurements	907
Delay in TR	0.00 ms

Sequence - Part 1

Sequence Name	epfid
Dimension	2D
Excitation	Standard
Gradient Mode	Performance
Flow Compensation	None
Bandwidth	2314 Hz/Px

Sequence - Part 1

Echo Spacing	0.54 ms
Free Echo Spacing	Off
EPI Factor	80

Sequence - Part 2

Introduction	Off
RF Spoiling	Off

Sequence - Special

Excite pulse duration	5120 us
Min. prep scans	0
Min. prep scans SB	0
Inter-TE delay	0 us
Delay before PC scans	0 us
Single-band images	On
MB LeakBlock kernel	On
MB dual kernel	Off
MB RF phase scramble	Off
Opt. MB RF pulse BW	Off
SENSE1 coil combine	On
Invert RO/PE polarity	Off
Force Maxwell corr.	Off
PF omits higher k-space	Off
Disable B1 control loop	Off
Disable freq. update	Off
Suppress 16-bit DICOM	Off
Force equal slice timing	Off
Online multi-band recon.	Online
FFT scale factor	1.00
Fat saturation FA	110.00 deg
Fat sat. offset	0.00 Hz
Sinc exc. pulse BWTP	5.20
Physio recording	DICOM
Triggering scheme	Standard

Sequence - Assistant

SAR Assistant	Off

\\USER\2023_MBC_ VIDA PROTOCOLS_XA50\SB.2020.353 AEP study - 64 Channel\SB.2020.353 - AEP MRI ACQUISITION PROTOCOL v23_Vida- 64 channel\FMap_AxObl_AP- *

TA: 10 sec Coil Selection: Auto Voxel Size: 2.0×2.0×2.0 mm³ Acc:: None Rel. SNR: 1.00

Properties

Start measurement without further preparation	On
Wait for User to Start	Off
Start measurements	Single Measurement
Prio Recon	Off
Auto Open Inline Display	Off
Auto Close Inline Display	Off
Load Images to MR View&GO	On
Auto Store Images	On
Disable auto transfer to PACS	Off
Load Images to Stamp Segments	Off
Load Images to Graphic Segments	Off
Graphic segment	Default
Inline Movie	Off

Routine

Slice Group	1
Slices	72
Distance Factor	0 %
Position	Isocenter
Orientation	T > C-30.0
Phase Encoding Dir.	A >> P
Phase Oversampling	0 %
FOV Read	216 mm
FOV Phase	111.1 %
Slice Thickness	2.0 mm
TR	10100.0 ms
TE	84.00 ms
Averages	1
Multi-band accel. factor	1
AutoAlign	---

Contrast - Common

TR	10100.0 ms
TE	84.00 ms
MTC	Off
Magn. Preparation	None
Flip Angle	90 deg
Refocus flip angle	180 deg
Fat-Water Contrast	Fat Saturation
Grad. rev. fat suppr.	Disabled
Contrasts	1
Reconstruction	Magnitude

Contrast - Dynamic

Dynamic Mode	Standard
Measurements	1
Delay in TR	0.00 ms

Resolution - Common

FOV Read	216 mm
FOV Phase	111.1 %
Slice Thickness	2.0 mm
Base Resolution	108
Phase Resolution	100 %
Interpolation	Off

Resolution - Acceleration

Acceleration Mode	None
Phase Partial Fourier	Off

Resolution - Filter

Raw Filter	Off
Elliptical Filter	Off
Hamming	Off
Distortion Correction	Off
Static Field Correction	Off
Normalize	Prescan

Geometry - Common

Slice Group	1
Slices	72
Distance Factor	0 %
Position	Isocenter
Orientation	T > C-30.0
Phase Encoding Dir.	A >> P
Phase Oversampling	0 %
FOV Read	216 mm
FOV Phase	111.1 %
Slice Thickness	2.0 mm
TR	10100.0 ms
Multi-Slice Mode	Interleaved
Series	Interleaved
Multi-band accel. factor	1

Geometry - AutoAlign

Slice Group	1
Position	Isocenter
Orientation	T > C-30.0
Phase Encoding Dir.	A >> P
AutoAlign	---
Initial Position	Isocenter
L	0.0 mm
P	0.0 mm
H	0.0 mm
Initial Orientation	T > C
T > C	-30.00
> S	0.00
Initial Rotation	0.00 deg

Geometry - Saturation

Special Saturation	None

Geometry - Tim Planning Suite

Set-n-Go Protocol	Off
Table Position	0 mm
Table Position	H
Inline Composing	Off

System - Miscellaneous

Coil Selection	Auto Coil Select
Radial Sorting	Off
MSMA	S - C - T
Sagittal	R >> L
Coronal	A >> P
Transversal	F >> H
Coil Combination	Sum of Squares
Matrix Optimization	Off
Coil Focus	Flat

System - Adjustments

Adjustment Strategy	Standard
B0 Shim	Standard
B1 Shim	TrueForm
CoilShim	Off
Adjustment Tolerance	Auto
Adjust with Body Coil	Off
Confirm Frequency	Never
Assume Silicone	Off

System - Adjust Volume

Position	Isocenter
Orientation	T > C-30.0
Rotation	90.00 deg
R >> L	216 mm
A >> P	240 mm
F >> H	144 mm
Reset	Off

System - pTx

B1 Shim	TrueForm
Excitation	Standard

System - Tx/Rx

Frequency 1H	123.213354 MHz
? Ref. Amplitude 1H	0.000 V
Reset	Off
Image Scaling	1.000

Physio - Signal

1st Signal/Mode	None
TR	10100.0 ms
Log Signals	Off
Multi-band accel. factor	1

BOLD

GLM Statistics	Off
Ignore Meas. at Start	0
Ignore After Transition	0
Model Transition States	On
Temp. Highpass Filter	On
Threshold	4.00
Paradigm Size	4
Meas[1]	Baseline
Meas[2]	Baseline
Meas[3]	Baseline
Meas[4]	Active
Motion Correction	Off
Spatial Filter	Off
Measurements	1
Delay in TR	0.00 ms

Sequence - Part 1

Sequence Name	epse
Dimension	2D
Excitation	Standard
RF Pulse Type	Normal
Gradient Mode	Performance
Bandwidth	2104 Hz/Px
Echo Spacing	0.61 ms
Free Echo Spacing	Off
EPI Factor	120

Sequence - Part 2

Introduction	Off

Sequence - Special

Min. prep scans	0
Delay before PC scans	0 us
SENSE1 coil combine	On
Invert RO/PE polarity	Off
Force GPA balance	On
Disable B1 control loop	Off
Disable freq. update	Off
Suppress 16-bit DICOM	Off
Force equal slice timing	Off
FFT scale factor	1.00
Fat saturation FA	110.00 deg
Fat sat. offset	0.00 Hz
Physio recording	DICOM
Triggering scheme	Standard

Sequence - Assistant

SAR Assistant	Off

\\USER\2023_MBC_ VIDA PROTOCOLS_XA50\SB.2020.353 AEP study - 64 Channel\SB.2020.353 - AEP MRI ACQUISITION PROTOCOL v23_Vida- 64 channel\FMap_AxObl_AP-InvROPE- *

TA: 10 sec Coil Selection: Auto Voxel Size: 2.0×2.0×2.0 mm³ Acc:: None Rel. SNR: 1.00

Properties

Start measurement without further preparation	On
Wait for User to Start	Off
Start measurements	Single Measurement
Prio Recon	Off
Auto Open Inline Display	Off
Auto Close Inline Display	Off
Load Images to MR View&GO	On
Auto Store Images	On
Disable auto transfer to PACS	Off
Load Images to Stamp Segments	Off
Load Images to Graphic Segments	Off
Graphic segment	Default
Inline Movie	Off

Routine

Slice Group	1
Slices	72
Distance Factor	0 %
Position	Isocenter
Orientation	T > C-30.0
Phase Encoding Dir.	A >> P
Phase Oversampling	0 %
FOV Read	216 mm
FOV Phase	111.1 %
Slice Thickness	2.0 mm
TR	10100.0 ms
TE	84.00 ms
Averages	1
Multi-band accel. factor	1
AutoAlign	---

Contrast - Common

TR	10100.0 ms
TE	84.00 ms
MTC	Off
Magn. Preparation	None
Flip Angle	90 deg
Refocus flip angle	180 deg
Fat-Water Contrast	Fat Saturation
Grad. rev. fat suppr.	Disabled
Contrasts	1
Reconstruction	Magnitude

Contrast - Dynamic

Dynamic Mode	Standard
Measurements	1
Delay in TR	0.00 ms

Resolution - Common

FOV Read	216 mm
FOV Phase	111.1 %
Slice Thickness	2.0 mm
Base Resolution	108
Phase Resolution	100 %
Interpolation	Off

Resolution - Acceleration

Acceleration Mode	None
Phase Partial Fourier	Off

Resolution - Filter

Raw Filter	Off
Elliptical Filter	Off
Hamming	Off
Distortion Correction	Off
Static Field Correction	Off
Normalize	Prescan

Geometry - Common

Slice Group	1
Slices	72
Distance Factor	0 %
Position	Isocenter
Orientation	T > C-30.0
Phase Encoding Dir.	A >> P
Phase Oversampling	0 %
FOV Read	216 mm
FOV Phase	111.1 %
Slice Thickness	2.0 mm
TR	10100.0 ms
Multi-Slice Mode	Interleaved
Series	Interleaved
Multi-band accel. factor	1

Geometry - AutoAlign

Slice Group	1
Position	Isocenter
Orientation	T > C-30.0
Phase Encoding Dir.	A >> P
AutoAlign	---
Initial Position	Isocenter
L	0.0 mm
P	0.0 mm
H	0.0 mm
Initial Orientation	T > C
T > C	-30.00
> S	0.00
Initial Rotation	0.00 deg

Geometry - Saturation

Special Saturation	None

Geometry - Tim Planning Suite

Set-n-Go Protocol	Off
Table Position	0 mm
Table Position	H
Inline Composing	Off

System - Miscellaneous

Coil Selection	Auto Coil Select
Radial Sorting	Off
MSMA	S - C - T
Sagittal	R >> L
Coronal	A >> P
Transversal	F >> H
Coil Combination	Sum of Squares
Matrix Optimization	Off
Coil Focus	Flat

System - Adjustments

Adjustment Strategy	Standard
B0 Shim	Standard
B1 Shim	TrueForm
CoilShim	Off
Adjustment Tolerance	Auto
Adjust with Body Coil	Off
Confirm Frequency	Never
Assume Silicone	Off

System - Adjust Volume

Position	Isocenter
Orientation	T > C-30.0
Rotation	90.00 deg
R >> L	216 mm
A >> P	240 mm
F >> H	144 mm
Reset	Off

System - pTx

B1 Shim	TrueForm
Excitation	Standard

System - Tx/Rx

Frequency 1H	123.213354 MHz
? Ref. Amplitude 1H	0.000 V
Reset	Off
Image Scaling	1.000

Physio - Signal

1st Signal/Mode	None
TR	10100.0 ms
Log Signals	Off
Multi-band accel. factor	1

BOLD

GLM Statistics	Off
Ignore Meas. at Start	0
Ignore After Transition	0
Model Transition States	On
Temp. Highpass Filter	On
Threshold	4.00
Paradigm Size	4
Meas[1]	Baseline
Meas[2]	Baseline
Meas[3]	Baseline
Meas[4]	Active
Motion Correction	Off
Spatial Filter	Off
Measurements	1
Delay in TR	0.00 ms

Sequence - Part 1

Sequence Name	epse
Dimension	2D
Excitation	Standard
RF Pulse Type	Normal
Gradient Mode	Performance
Bandwidth	2104 Hz/Px
Echo Spacing	0.61 ms
Free Echo Spacing	Off
EPI Factor	120

Sequence - Part 2

Introduction	Off

Sequence - Special

Min. prep scans	0
Delay before PC scans	0 us
SENSE1 coil combine	On
Invert RO/PE polarity	On
Force GPA balance	On
Disable B1 control loop	Off
Disable freq. update	Off
Suppress 16-bit DICOM	Off
Force equal slice timing	Off
FFT scale factor	1.00
Fat saturation FA	110.00 deg
Fat sat. offset	0.00 Hz
Physio recording	DICOM
Triggering scheme	Standard

Sequence - Assistant

SAR Assistant	Off

AEP MRI Protocol for Siemens 3T Cima.X (XA61)

Table of contents

\\SAHMRI_Research

Head

AEP

AEP MRI ACQUISITION PROTOCOL Cima_v21

Localiser- *
T2w_TSE_Axial- *
MEGRE_Ax_RL- *
T1w_Sag_AP- *
T2wFLAIR_Sag_AP- *
T2w_CorOblHipp_RL- *
T2w_AxOblTempPole_RL- *
DWI_Ax_AP_52Dir- *
DWI_Ax_AP_51Dir_InvROPE- *
T1map_MP2RAGE_Sag_AP- *
Localiser-OptionalREPEAT- *
T1w_Sag_AP-OptionalREPEAT- *
T2wFLAIR_Sag_AP-OptionalREPEAT- *
T2w_CorOblHipp_RL-OptionalREPEAT *
fMRI_AxObl_AP_Pseudoword- *
FMap_AxObl_AP- *
FMap_AxObl_AP_InvROPE- *
fMRI_AxObl_AP_OneBack- *
FMap_AxObl_AP- *
FMap_AxObl_AP_InvROPE- *
fMRI_AxObl_AP_Movie- *
FMap_AxObl_AP- *
FMap_AxObl_AP_InvROPE- *

\\SAHMRI_Research\Head\AEP\AEP MRI ACQUISITION PROTOCOL Cima_v21\Localiser- *

TA: 57 sec Coil Selection: Auto Voxel Size: 0.5×0.5×7.0 mm³ Acc:: None Rel. SNR: 1.00

Properties

Start measurement without further preparation	Off
Wait for User to Start	Off
Start measurements	Single Measurement
Prio Recon	Off
Auto Open Inline Display	Off
Auto Close Inline Display	Off
Load Images to MR View&GO	On
Auto Store Images	On
Disable auto transfer to PACS	Off
Load Images to Stamp Segments	On
Load Images to Graphic Segments	Off
Graphic segment	Default
Inline Movie	Off

Routine

Slice Group	1
Slices	8
Distance Factor	100 %
Position	Isocenter
Orientation	Sagittal
Phase Encoding Dir.	A >> P
Slice Group	2
Slices	8
Distance Factor	100 %
Position	Isocenter
Orientation	Sagittal
Phase Encoding Dir.	A >> P
Slice Group	3
Slices	8
Distance Factor	100 %
Position	Isocenter
Orientation	Sagittal
Phase Encoding Dir.	A >> P
Phase Oversampling	0 %
FOV Read	250 mm
FOV Phase	100.0 %
Slice Thickness	7.0 mm
TR	8.6 ms
TE	4.00 ms
Averages	2
Concatenations	14
AutoAlign	---

Contrast - Common

TR	8.6 ms
TE	4.00 ms
TD	0.00 ms
MTC	Off
Magn. Preparation	None
Flip Angle	20 deg

Contrast - Common

Fat-Water Contrast	Standard
Dark Blood	Off
Contrasts	1
SWI	Off
Reconstruction	Magnitude

Contrast - Dynamic

Dynamic Mode	Standard
Measurements	1
Multiple Series	Each Measurement

Resolution - Common

FOV Read	250 mm
FOV Phase	100.0 %
Slice Thickness	7.0 mm
Base Resolution	256
Phase Resolution	90 %
Interpolation	On

Resolution - Acceleration

Acceleration Mode	None
Deep Resolve	Off
Phase Partial Fourier	Off
Asymmetric Echo	Allowed

Resolution - Filter

Raw Filter	Off
Elliptical Filter	On
Distortion Correction	2D
Normalize	Prescan
Image Filter	Off

Geometry - Common

Slice Group	1
Slices	8
Distance Factor	100 %
Position	Isocenter
Orientation	Sagittal
Phase Encoding Dir.	A >> P
Slice Group	2
Slices	8
Distance Factor	100 %
Position	Isocenter
Orientation	Sagittal
Phase Encoding Dir.	A >> P
Slice Group	3
Slices	8
Distance Factor	100 %
Position	Isocenter
Orientation	Sagittal

Geometry - Common

Phase Encoding Dir.	A >> P
Phase Oversampling	0 %
FOV Read	250 mm
FOV Phase	100.0 %
Slice Thickness	7.0 mm
TR	8.6 ms
Multi-Slice Mode	Sequential
Series	Interleaved
Concatenations	14

Geometry - AutoAlign

Slice Group	1
Position	Isocenter
Orientation	Sagittal
Phase Encoding Dir.	A >> P
Slice Group	2
Position	Isocenter
Orientation	Sagittal
Phase Encoding Dir.	A >> P
Slice Group	3
Position	Isocenter
Orientation	Sagittal
Phase Encoding Dir.	A >> P
AutoAlign	---
Initial Position	Isocenter
L	0.0 mm
P	0.0 mm
H	0.0 mm
Initial Orientation	Sagittal
Initial Rotation	0.00 deg

Geometry - Saturation

Saturation Mode	Standard
Special Saturation	None

Geometry - Tim Planning Suite

Set-n-Go Protocol	Off
Table Position	0 mm
Table Position	H
Inline Composing	Off

System - Miscellaneous

Coil Selection	ACS All but spine
Radial Sorting	Off
MSMA	S - C - T
Sagittal	R >> L
Coronal	P >> A
Transversal	F >> H
Coil Combination	Adaptive Combine
Matrix Optimization	Off
Coil Focus	Flat

System - Adjustments

Adjustment Strategy	Standard

System - Adjustments

B0 Shim	Tune up
B1 Shim	TrueForm
CoilShim	Off
Adjustment Tolerance	Auto
Adjust with Body Coil	Off
Confirm Frequency	Never
Assume Silicone	Off

System - Adjust Volume

Position	Isocenter
Orientation	Transversal
Rotation	0.00 deg
A >> P	263 mm
R >> L	350 mm
F >> H	350 mm
Reset	Off

System - pTx

B1 Shim	TrueForm
Excitation	Slice-sel.
LR Balancing	Off

System - Tx/Rx

Frequency 1H	123.256549 MHz
? Ref. Amplitude 1H	0.000 V
Reset	Off
Image Scaling	1.000

Physio - Signal

1st Signal/Mode	None
TR	8.6 ms
Segments	1
Concatenations	14

Physio - Cardiac

Tagging	None
Fat-Water Contrast	Standard
Magn. Preparation	None
Dark Blood	Off
FOV Read	250 mm
FOV Phase	100.0 %
Phase Resolution	90 %

Physio - PACE

Resp. Control	Off
Concatenations	14

Inline - Liver

Liver Registration	Off
Save Original Images	On

Inline - Subtraction

Subtract	Off
Measurements	1

Inline - Subtraction

StdDev	Off
Save Original Images	On

Inline - MIP

MIP Sag	Off
MIP Cor	Off
MIP Tra	Off
MIP Time	Off
Radial MIP	Off
Save Original Images	On
MPR Sag	Off
MPR Cor	Off
MPR Tra	Off

Inline - Soft Tissue

Wash-in	Off
Wash-out	Off
TTP	Off
PEI	Off
MIP Time	Off
Measurements	1

Inline - Composing

Inline Composing	Off

Inline - MapIt

MapIt	None
Flip Angle	20 deg
Measurements	1
Contrasts	1
TE	4.00 ms
TR	8.6 ms
Save Original Images	On

Inline - Open Recon

Algorithm	None

Sequence - Part 1

Sequence Name	fl
Dimension	2D
Excitation	Slice-sel.
RF Pulse Type	Normal
Gradient Mode	Normal
Flow Compensation	None
Bandwidth	320 Hz/Px
Asymmetric Echo	Allowed
Segments	1

Sequence - Part 2

Introduction	On
RF Spoiling	On
Acoustic noise reduction	Off

Sequence - Assistant

SAR Assistant	Off
Allowed Delay	0 s

\\SAHMRI_Research\Head\AEP\AEP MRI ACQUISITION PROTOCOL Cima_v21\T2w_TSE_Axial- *

TA: 3:28 min Coil Selection: Auto Voxel Size: 0.2×0.2×3.0 mm³ Acc:: None Rel. SNR: 1.00

Properties

Start measurement without further preparation	On
Wait for User to Start	On
Start measurements	Single Measurement
Prio Recon	Off
Auto Open Inline Display	Off
Auto Close Inline Display	Off
Load Images to MR View&GO	On
Auto Store Images	On
Disable auto transfer to PACS	Off
Load Images to Stamp Segments	On
Load Images to Graphic Segments	Off
Graphic segment	Default
Inline Movie	Off

Routine

Slice Group	1
Slices	44
Distance Factor	0 %
Position	R3.8 A10.0 F36.4 mm
Orientation	T > C-13.4
Phase Encoding Dir.	R >> L
Phase Oversampling	5 %
FOV Read	208 mm
FOV Phase	84.4 %
Slice Thickness	3.0 mm
TR	4300.0 ms
TE	83.00 ms
Averages	1
Concatenations	2
AutoAlign	---

Contrast - Common

TR	4300.0 ms
TE	83.00 ms
TD	0.00 ms
MTC	Off
Magn. Preparation	None
Flip Angle Mode	Constant
Flip Angle 1	120 deg
Flip Angle 2	90 deg
Fat-Water Contrast	Standard
Dark Blood	Off
Contrasts	1
Wrap-up Magn.	None
Reconstruction	Magnitude

Contrast - Dynamic

Dynamic Mode	Standard
Measurements	1
Multiple Series	Each Measurement

Resolution - Common

FOV Read	208 mm
FOV Phase	84.4 %
Slice Thickness	3.0 mm
Base Resolution	448
Phase Resolution	100 %
Interpolation	On

Resolution - Acceleration

Acceleration Mode	None
Deep Resolve	Off
Phase Partial Fourier	Off

Resolution - Filter

Raw Filter	Off
Elliptical Filter	Off
Distortion Correction	2D
Normalize	Prescan
Image Filter	On

Geometry - Common

Slice Group	1
Slices	44
Distance Factor	0 %
Position	R3.8 A10.0 F36.4 mm
Orientation	T > C-13.4
Phase Encoding Dir.	R >> L
Phase Oversampling	5 %
FOV Read	208 mm
FOV Phase	84.4 %
Slice Thickness	3.0 mm
TR	4300.0 ms
Multi-Slice Mode	Interleaved
Series	Interleaved
Concatenations	2

Geometry - AutoAlign

Slice Group	1
Position	R3.8 A10.0 F36.4 mm
Orientation	T > C-13.4
Phase Encoding Dir.	R >> L
AutoAlign	---
Initial Position	R3.8 A10.0 F36.4
R	3.8 mm
A	10.0 mm
F	36.4 mm
Initial Orientation	T > C
T > C	-13.40
> S	0.00
Initial Rotation	90.00 deg

Geometry - Navigator

Geometry - Saturation

Special Saturation	None

Geometry - Tim Planning Suite

Set-n-Go Protocol	Off
Table Position	0 mm
Table Position	H
Inline Composing	Off

System - Miscellaneous

Coil Selection	Auto Coil Select
Radial Sorting	Off
MSMA	S - C - T
Sagittal	L >> R
Coronal	A >> P
Transversal	F >> H
Coil Combination	Adaptive Combine
Matrix Optimization	Off
Coil Focus	Flat

System - Adjustments

Adjustment Strategy	Standard
B0 Shim	Standard
B1 Shim	TrueForm
CoilShim	Off
Adjustment Tolerance	Auto
Adjust with Body Coil	Off
Confirm Frequency	Never
Assume Silicone	Off

System - Adjust Volume

Position	R3.8 A10.0 F36.4 mm
Orientation	T > C-13.4
Rotation	90.00 deg
R >> L	176 mm
A >> P	208 mm
F >> H	132 mm
Reset	Off

System - pTx

B1 Shim	TrueForm
LR Balancing	Off

System - Tx/Rx

Frequency 1H	123.256549 MHz
? Ref. Amplitude 1H	0.000 V
Reset	Off
Image Scaling	1.000

Physio - Signal

1st Signal/Mode	None
TR	4300.0 ms
Concatenations	2

Physio - Cardiac

Fat-Water Contrast	Standard
Magn. Preparation	None
Dark Blood	Off
FOV Read	208 mm
FOV Phase	84.4 %
Phase Resolution	100 %
Motion Correction	None

Physio - PACE

Resp. Control	Off
Concatenations	2

Inline - Subtraction

Subtract	Off
Measurements	1
StdDev	Off
Motion Correction	None
Save Original Images	On

Inline - MIP

MIP Sag	Off
MIP Cor	Off
MIP Tra	Off
MIP Time	Off
Radial MIP	Off
Save Original Images	On
MPR Sag	Off
MPR Cor	Off
MPR Tra	Off

Inline - Composing

Inline Composing	Off

Inline - Open Recon

Algorithm	None

Sequence - Part 1

Sequence Name	tse_rr
Dimension	2D
RF Pulse Type	Fast
Gradient Mode	Fast
Flow Compensation	Read
Bandwidth	180 Hz/Px
Echo Spacing	10.4 ms
Free Echo Spacing	Off
Define	Turbo Factor
Turbo Factor	18
Echo Trains per Slice	23

Sequence - Part 2

Introduction	On
Phase Correction	Automatic
Compensate T2 Decay	Off
Fast Mode	Off

Sequence - Part 2

WARP	Off
Red. EC Sensitivity	Off
Acoustic noise reduction	Off
Reduce Motion Sens.	On
Motion Correction	None

Sequence - Assistant

SAR Assistant	Off
Allowed Delay	60 s

\\SAHMRI_Research\Head\AEP\AEP MRI ACQUISITION PROTOCOL Cima_v21\MEGRE_Ax_RL- *

TA: 3:39 min Coil Selection: Auto Voxel Size: 1.2×1.2×1.2 mm³ Acc:: 3 Rel. SNR: 1.00

Properties

Start measurement without further preparation	On
Wait for User to Start	On
Start measurements	Single Measurement
Prio Recon	Off
Auto Open Inline Display	Off
Auto Close Inline Display	Off
Load Images to MR View&GO	On
Auto Store Images	On
Disable auto transfer to PACS	Off
Load Images to Stamp Segments	Off
Load Images to Graphic Segments	Off
Graphic segment	Default
Inline Movie	Off

Routine

Slab Group	1
Slabs	1
Distance Factor	20 %
Position	Isocenter
Orientation	Transversal
Phase Encoding Dir.	R >> L
Slices per Slab	120
Phase Oversampling	10 %
Slice Oversampling	0.0 %
FOV Read	230 mm
FOV Phase	81.3 %
Slice Thickness	1.20 mm
TR	30.0 ms
TE 1	5.84 ms
TE 2	10.63 ms
TE 3	15.42 ms
TE 4	20.21 ms
TE 5	25.00 ms
Averages	1
Concatenations	1
AutoAlign	---

Contrast - Common

TR	30.0 ms
TE 1	5.84 ms
TE 2	10.63 ms
TE 3	15.42 ms
TE 4	20.21 ms
TE 5	25.00 ms
MTC	Off
Magn. Preparation	None
Flip Angle	15 deg
Fat-Water Contrast	Standard
Dark Blood	Off
Contrasts	5

Contrast - Common

SWI	Off
Reconstruction	Magn./Phase

Contrast - Dynamic

Dynamic Mode	Standard
Measurements	1
Multiple Series	Each Measurement

Resolution - Common

FOV Read	230 mm
FOV Phase	81.3 %
Slice Thickness	1.20 mm
Base Resolution	192
Phase Resolution	100 %
Slice Resolution	100 %
Interpolation	Off

Resolution - Acceleration

Acceleration Mode	GRAPPA
Reference Scans	Integrated
Acceleration Factor PE	3
Reference Lines PE	24
Acceleration Factor 3D	1
Deep Resolve	Off
Phase Partial Fourier	Off
Slice Partial Fourier	Off
Asymmetric Echo	Off
Elliptical Scanning	On

Resolution - Filter

Raw Filter	Off
Elliptical Filter	Off
Distortion Correction	2D
Normalize	Prescan
Image Filter	Off

Geometry - Common

Slab Group	1
Slabs	1
Distance Factor	20 %
Position	Isocenter
Orientation	Transversal
Phase Encoding Dir.	R >> L
Slices per Slab	120
Phase Oversampling	10 %
Slice Oversampling	0.0 %
FOV Read	230 mm
FOV Phase	81.3 %
Slice Thickness	1.20 mm
TR	30.0 ms

Geometry - Common

Multi-Slice Mode	Interleaved
Series	Interleaved
Concatenations	1

Geometry - AutoAlign

Slab Group	1
Position	Isocenter
Orientation	Transversal
Phase Encoding Dir.	R >> L
AutoAlign	---
Initial Position	Isocenter
L	0.0 mm
P	0.0 mm
H	0.0 mm
Initial Orientation	Transversal
Initial Rotation	90.00 deg

Geometry - Saturation

Saturation Mode	Standard
Special Saturation	None

Geometry - Tim Planning Suite

Set-n-Go Protocol	Off
Table Position	0 mm
Table Position	H
Inline Composing	Off

System - Miscellaneous

Coil Selection	ACS All but spine
Radial Sorting	Off
MSMA	S - C - T
Sagittal	R >> L
Coronal	A >> P
Transversal	F >> H
Coil Combination	Adaptive Combine
Matrix Optimization	Off
Coil Focus	Flat

System - Adjustments

Adjustment Strategy	Standard
B0 Shim	Standard
B1 Shim	TrueForm
CoilShim	Off
Adjustment Tolerance	Auto
Adjust with Body Coil	Off
Confirm Frequency	Never
Assume Silicone	Off

System - Adjust Volume

Position	Isocenter
Orientation	Transversal
Rotation	90.00 deg
R >> L	187 mm
A >> P	230 mm

System - Adjust Volume

F >> H	144 mm
Reset	Off

System - pTx

B1 Shim	TrueForm
Excitation	Slab-sel.
LR Balancing	Off

System - Tx/Rx

Frequency 1H	123.256549 MHz
? Ref. Amplitude 1H	0.000 V
Reset	Off
Image Scaling	1.000

Physio - Signal

1st Signal/Mode	None
TR	30.0 ms
Segments	1
Concatenations	1

Physio - Cardiac

Tagging	None
Fat-Water Contrast	Standard
Magn. Preparation	None
Dark Blood	Off
FOV Read	230 mm
FOV Phase	81.3 %
Phase Resolution	100 %

Physio - PACE

Resp. Control	Off
Concatenations	1

Inline - Liver

Liver Registration	Off
Save Original Images	On

Inline - Subtraction

Subtract	Off
Measurements	1
StdDev	Off
Save Original Images	On

Inline - MIP

MIP Sag	Off
MIP Cor	Off
MIP Tra	Off
MIP Time	Off
Radial MIP	Off
Save Original Images	On
MPR Sag	Off
MPR Cor	Off
MPR Tra	Off

Inline - Soft Tissue

Wash-in	Off
Wash-out	Off
TTP	Off
PEI	Off
MIP Time	Off
Measurements	1

Inline - Composing

Inline Composing	Off

Inline - MapIt

MapIt	None
Flip Angle	15 deg
Measurements	1
Contrasts	5
TE 1	5.84 ms
TE 2	10.63 ms
TE 3	15.42 ms
TE 4	20.21 ms
TE 5	25.00 ms
TR	30.0 ms
Save Original Images	On

Inline - Open Recon

Algorithm	None

Sequence - Part 1

Sequence Name	fl_r
Dimension	3D
Excitation	Slab-sel.
RF Pulse Type	Fast
Readout Mode	Monopolar
Gradient Mode	Normal
Flow Compensation 1	On
Flow Compensation 2	None
Flow Compensation 3	None
Flow Compensation 4	None
Flow Compensation 5	None
Bandwidth 1	310 Hz/Px
Bandwidth 2	310 Hz/Px
Bandwidth 3	310 Hz/Px
Bandwidth 4	310 Hz/Px
Bandwidth 5	310 Hz/Px
Asymmetric Echo	Off
Segments	1

Sequence - Part 2

Introduction	Off
RF Spoiling	On
Acoustic noise reduction	Off

Sequence - Assistant

SAR Assistant	Off
Allowed Delay	0 s

\\SAHMRI_Research\Head\AEP\AEP MRI ACQUISITION PROTOCOL Cima_v21\T1w_Sag_AP- *
TA: 4:32 min Coil Selection: Auto Voxel Size: 0.9×0.9×0.9 mm^3 Acc:: 2 Rel. SNR: 1.00

Properties

Start measurement without further preparation	On
Wait for User to Start	Off
Start measurements	Single Measurement
Prio Recon	Off
Auto Open Inline Display	Off
Auto Close Inline Display	Off
Load Images to MR View&GO	On
Auto Store Images	On
Disable auto transfer to PACS	Off
Load Images to Stamp Segments	Off
Load Images to Graphic Segments	Off
Graphic segment	Default
Inline Movie	Off

Routine

Slab Group	1
Slabs	1
Distance Factor	50 %
Position	Isocenter
Orientation	Sagittal
Phase Encoding Dir.	A >> P
Slices per Slab	192
Phase Oversampling	0 %
Slice Oversampling	25.0 %
FOV Read	230 mm
FOV Phase	100.0 %
Slice Thickness	0.90 mm
TR	1900.0 ms
TE	2.19 ms
Averages	1
Concatenations	1
AutoAlign	---

Contrast - Common

TR	1900.0 ms
TE	2.19 ms
Magn. Preparation	Non-sel. IR
TI	1000 ms
Flip Angle	8 deg
Fat-Water Contrast	Fast Water Excitation
Dark Blood	Off
Contrasts	1
Reconstruction	Magnitude

Contrast - Dynamic

Dynamic Mode	Standard
Measurements	1
Multiple Series	Each Measurement
Reordering	Linear

Resolution - Common

FOV Read	230 mm
FOV Phase	100.0 %
Slice Thickness	0.90 mm
Base Resolution	256
Phase Resolution	100 %
Slice Resolution	100 %
Interpolation	Off

Resolution - Acceleration

Acceleration Mode	GRAPPA
Reference Scans	Integrated
Acceleration Factor PE	2
Reference Lines PE	32
Acceleration Factor 3D	1
Phase Partial Fourier	Off
Slice Partial Fourier	Off
Asymmetric Echo	Allowed
Elliptical Scanning	Off

Resolution - Filter

Raw Filter	Off
Elliptical Filter	Off
Distortion Correction	2D
Normalize	Prescan
Image Filter	Off

Geometry - Common

Slab Group	1
Slabs	1
Distance Factor	50 %
Position	Isocenter
Orientation	Sagittal
Phase Encoding Dir.	A >> P
Slices per Slab	192
Phase Oversampling	0 %
Slice Oversampling	25.0 %
FOV Read	230 mm
FOV Phase	100.0 %
Slice Thickness	0.90 mm
TR	1900.0 ms
Multi-Slice Mode	Single Shot
Series	Interleaved
Concatenations	1

Geometry - AutoAlign

Slab Group	1
Position	Isocenter
Orientation	Sagittal
Phase Encoding Dir.	A >> P
AutoAlign	---

Geometry - AutoAlign

Initial Position	Isocenter
L	0.0 mm
P	0.0 mm
H	0.0 mm
Initial Orientation	Sagittal
Initial Rotation	0.00 deg

Geometry - Navigator

Geometry - Tim Planning Suite

Set-n-Go Protocol	Off
Table Position	0 mm
Table Position	H
Inline Composing	Off

System - Miscellaneous

Coil Selection	Auto Coil Select
Radial Sorting	Off
MSMA	S - C - T
Sagittal	R >> L
Coronal	P >> A
Transversal	F >> H
Coil Combination	Adaptive Combine
Matrix Optimization	Off
Coil Focus	Flat

System - Adjustments

Adjustment Strategy	Standard
B0 Shim	Standard
B1 Shim	TrueForm
CoilShim	Off
Adjustment Tolerance	Auto
Adjust with Body Coil	Off
Confirm Frequency	Never
Assume Silicone	Off

System - Adjust Volume

Position	Isocenter
Orientation	Sagittal
Rotation	0.00 deg
A >> P	230 mm
F >> H	230 mm
R >> L	173 mm
Reset	Off

System - pTx

B1 Shim	TrueForm
Excitation	Non-sel.

System - Tx/Rx

Frequency 1H	123.256549 MHz
? Ref. Amplitude 1H	0.000 V
Reset	Off
Image Scaling	1.000

Physio - Signal

1st Signal/Mode	None
TR	1900.0 ms
Concatenations	1

Physio - Cardiac

Fat-Water Contrast	Fast Water Excitation
Magn. Preparation	Non-sel. IR
TI	1000 ms
Dark Blood	Off
FOV Read	230 mm
FOV Phase	100.0 %
Phase Resolution	100 %
Motion Correction	None

Physio - PACE

Resp. Control	Off
Concatenations	1

Inline - Subtraction

Subtract	Off
Measurements	1
StdDev	Off
Save Original Images	On

Inline - MIP

MIP Sag	Off
MIP Cor	Off
MIP Tra	Off
MIP Time	Off
Radial MIP	Off
Save Original Images	On
MPR Sag	Off
MPR Cor	Off
MPR Tra	Off

Inline - Composing

Inline Composing	Off

Inline - MapIt

MapIt	None
Flip Angle	8 deg
Measurements	1
Contrasts	1
TE	2.19 ms
TR	1900.0 ms
Save Original Images	On

Inline - Open Recon

Algorithm	None

Sequence - Part 1

Sequence Name	tfl
Dimension	3D
Excitation	Non-sel.

Sequence - Part 1

RF Pulse Type	Fast
Gradient Mode	Performance
Flow Compensation	None
Reordering	Linear
Bandwidth	220 Hz/Px
Echo Spacing	7.38 ms
Asymmetric Echo	Allowed
Turbo Factor	240

Sequence - Part 2

Introduction	Off
RF Spoiling	On
Incr. Gradient Spoiling	Off
Motion Correction	None

Sequence - Assistant

SAR Assistant	Off

\\SAHMRI_Research\Head\AEP\AEP MRI ACQUISITION PROTOCOL Cima_v21\T2wFLAIR_Sag_AP- *
TA: 5:37 min Coil Selection: Auto Voxel Size: 0.9×0.9×0.9 mm³ Acc:: 4 Rel. SNR: 1.00

Properties

Start measurement without further preparation	On
Wait for User to Start	On
Start measurements	Single Measurement
Prio Recon	Off
Auto Open Inline Display	Off
Auto Close Inline Display	Off
Load Images to MR View&GO	On
Auto Store Images	On
Disable auto transfer to PACS	Off
Load Images to Stamp Segments	Off
Load Images to Graphic Segments	Off
Graphic segment	Default
Inline Movie	Off

Routine

Slab Group	1
Slabs	1
Position	Isocenter
Orientation	Sagittal
Phase Encoding Dir.	A >> P
Slices per Slab	192
Phase Oversampling	0 %
Slice Oversampling	0.0 %
FOV Read	230 mm
FOV Phase	100.0 %
Slice Thickness	0.90 mm
TR	5000.0 ms
TE	392.00 ms
Averages	2.0
Concatenations	1
AutoAlign	---

Contrast - Common

TR	5000.0 ms
TE	392.00 ms
MTC	Off
Magn. Preparation	Non-sel. T2 prep. IR
TI	1600 ms
T2 Prep. Duration	125 ms
Flip Angle Mode	T2 Var
Flip Angle 1	120 deg
Flip Angle 2	90 deg
Fat-Water Contrast	Standard
Dark Blood	Off
Blood Suppression	Off
Wrap-up Magn.	None
Reconstruction	Magnitude

Contrast - Dynamic

Dynamic Mode	Standard
Measurements	1
Multiple Series	Each Measurement
Reordering	Linear

Resolution - Common

FOV Read	230 mm
FOV Phase	100.0 %
Slice Thickness	0.90 mm
Base Resolution	256
Phase Resolution	100 %
Slice Resolution	100 %
Interpolation	Off

Resolution - Acceleration

Acceleration Mode	CAIPIRINHA
Total Factor	4
Reference Scans	Integrated
Acceleration Factor PE	2
Reference Lines PE	24
Acceleration Factor 3D	2
Reference Lines 3D	24
Reordering Shift 3D	1
Phase Partial Fourier	Allowed
Slice Partial Fourier	Off
Elliptical Scanning	On

Resolution - Filter

Raw Filter	Off
Elliptical Filter	Off
Distortion Correction	2D
Normalize	Prescan
Image Filter	On

Geometry - Common

Slab Group	1
Slabs	1
Position	Isocenter
Orientation	Sagittal
Phase Encoding Dir.	A >> P
Slices per Slab	192
Phase Oversampling	0 %
Slice Oversampling	0.0 %
FOV Read	230 mm
FOV Phase	100.0 %
Slice Thickness	0.90 mm
TR	5000.0 ms
Concatenations	1

Geometry - AutoAlign

Slab Group	1

Geometry - AutoAlign

Position	Isocenter
Orientation	Sagittal
Phase Encoding Dir.	A >> P
AutoAlign	---
Initial Position	Isocenter
L	0.0 mm
P	0.0 mm
H	0.0 mm
Initial Orientation	Sagittal
Initial Rotation	0.00 deg

Geometry - Navigator

Geometry - Saturation

Special Saturation	None

Geometry - Tim Planning Suite

Set-n-Go Protocol	Off
Table Position	0 mm
Table Position	H
Inline Composing	Off

System - Miscellaneous

Coil Selection	ACS All but spine
Radial Sorting	Off
MSMA	S - C - T
Sagittal	R >> L
Coronal	P >> A
Transversal	F >> H
Coil Combination	Adaptive Combine
Matrix Optimization	Off
Coil Focus	Flat

System - Adjustments

Adjustment Strategy	Standard
B0 Shim	Standard
B1 Shim	TrueForm
CoilShim	Off
Adjustment Tolerance	Auto
Adjust with Body Coil	Off
Confirm Frequency	Never
Assume Silicone	Off

System - Adjust Volume

Position	Isocenter
Orientation	Sagittal
Rotation	0.00 deg
A >> P	230 mm
F >> H	230 mm
R >> L	173 mm
Reset	Off

System - pTx

B1 Shim	TrueForm
Excitation	Non-sel.

System - Tx/Rx

Frequency 1H	123.256549 MHz
? Ref. Amplitude 1H	0.000 V
Reset	Off
Image Scaling	1.000
Gain	High

Physio - Signal

1st Signal/Mode	None
Trigger Delay	0 ms
TR	5000.0 ms
Concatenations	1

Physio - Cardiac

Fat-Water Contrast	Standard
Magn. Preparation	Non-sel. T2 prep. IR
TI	1600 ms
T2 Prep. Duration	125 ms
Dark Blood	Off
FOV Read	230 mm
FOV Phase	100.0 %
Phase Resolution	100 %

Physio - PACE

Resp. Control	Off
Concatenations	1

Inline - Subtraction

Subtract	Off
Measurements	1
StdDev	Off
Save Original Images	On

Inline - MIP

MIP Sag	Off
MIP Cor	Off
MIP Tra	Off
MIP Time	Off
Radial MIP	Off
Save Original Images	On
MPR Sag	Off
MPR Cor	Off
MPR Tra	Off

Inline - Composing

Inline Composing	Off

Inline - Open Recon

Algorithm	None

Sequence - Part 1

Sequence Name	spcir

Sequence - Part 1

Dimension	3D
Excitation	Non-sel.
RF Pulse Type	Normal
Gradient Mode	Fast
Flow Compensation	None
Reordering	Linear
Bandwidth	781 Hz/Px
Echo Spacing	3.60 ms
Turbo Factor	310
Echo Train Duration	947 ms

Sequence - Part 2

Introduction	On

Sequence - Assistant

SAR Assistant	Off
Allowed Delay	0 s

\\SAHMRI_Research\Head\AEP\AEP MRI ACQUISITION PROTOCOL Cima_v21\T2w_CorOblHipp_RL- *

TA: 4:53 min Coil Selection: Auto Voxel Size: 0.4×0.4×2.0 mm³ Acc:: None Rel. SNR: 1.00

Properties

Start measurement without further preparation	On
Wait for User to Start	On
Start measurements	Single Measurement
Prio Recon	Off
Auto Open Inline Display	Off
Auto Close Inline Display	Off
Load Images to MR View&GO	On
Auto Store Images	On
Disable auto transfer to PACS	Off
Load Images to Stamp Segments	On
Load Images to Graphic Segments	Off
Graphic segment	Default
Inline Movie	Off

Routine

Slice Group	1
Slices	36
Distance Factor	0 %
Position	Isocenter
Orientation	C > T-25.0
Phase Encoding Dir.	R >> L
Phase Oversampling	54 %
FOV Read	168 mm
FOV Phase	100.0 %
Slice Thickness	2.0 mm
TR	8100.0 ms
TE	83.00 ms
Averages	1
Concatenations	1
AutoAlign	---

Contrast - Common

TR	8100.0 ms
TE	83.00 ms
MTC	Off
Magn. Preparation	None
Flip Angle Mode	Hyperecho
Flip Angle 1	119 deg
Flip Angle 2	90 deg
Fat-Water Contrast	Standard
Dark Blood	Off
Contrasts	1
Wrap-up Magn.	Restore
Reconstruction	Magnitude

Contrast - Dynamic

Dynamic Mode	Standard
Measurements	1
Multiple Series	Each Measurement

Resolution - Common

FOV Read	168 mm
FOV Phase	100.0 %
Slice Thickness	2.0 mm
Base Resolution	384
Phase Resolution	100 %
Interpolation	Off

Resolution - Acceleration

Acceleration Mode	None
Deep Resolve	Off
Phase Partial Fourier	Off

Resolution - Filter

Raw Filter	Off
Elliptical Filter	Off
Distortion Correction	2D
Normalize	Prescan
Image Filter	On

Geometry - Common

Slice Group	1
Slices	36
Distance Factor	0 %
Position	Isocenter
Orientation	C > T-25.0
Phase Encoding Dir.	R >> L
Phase Oversampling	54 %
FOV Read	168 mm
FOV Phase	100.0 %
Slice Thickness	2.0 mm
TR	8100.0 ms
Multi-Slice Mode	Interleaved
Series	Interleaved
Concatenations	1

Geometry - AutoAlign

Slice Group	1
Position	Isocenter
Orientation	C > T-25.0
Phase Encoding Dir.	R >> L
AutoAlign	---
Initial Position	Isocenter
L	0.0 mm
P	0.0 mm
H	0.0 mm
Initial Orientation	C > T
C > T	-25.00
> S	0.00
Initial Rotation	0.00 deg

Geometry - Navigator

Geometry - Saturation

Saturation Region	1
Thickness	75.00 mm
Position	L0.0 P4.8 F105.8 mm
Orientation	Transversal
Shape	Standard
Special Saturation	None

Geometry - Tim Planning Suite

Set-n-Go Protocol	Off
Table Position	0 mm
Table Position	H
Inline Composing	Off

System - Miscellaneous

Coil Selection	Auto Coil Select
Radial Sorting	Off
MSMA	S - C - T
Sagittal	L >> R
Coronal	A >> P
Transversal	F >> H
Coil Combination	Adaptive Combine
Matrix Optimization	Off
Coil Focus	Flat

System - Adjustments

Adjustment Strategy	Standard
B0 Shim	Standard
B1 Shim	TrueForm
CoilShim	Off
Adjustment Tolerance	Auto
Adjust with Body Coil	Off
Confirm Frequency	Never
Assume Silicone	Off

System - Adjust Volume

Position	Isocenter
Orientation	C > T-25.0
Rotation	0.00 deg
R >> L	168 mm
F >> H	168 mm
A >> P	72 mm
Reset	Off

System - pTx

B1 Shim	TrueForm
LR Balancing	Off

System - Tx/Rx

Frequency 1H	123.256549 MHz
? Ref. Amplitude 1H	0.000 V
Reset	Off
Image Scaling	1.000

Physio - Signal

1st Signal/Mode	None
TR	8100.0 ms
Concatenations	1

Physio - Cardiac

Fat-Water Contrast	Standard
Magn. Preparation	None
Dark Blood	Off
FOV Read	168 mm
FOV Phase	100.0 %
Phase Resolution	100 %
Motion Correction	None

Physio - PACE

Resp. Control	Off
Concatenations	1

Inline - Subtraction

Subtract	Off
Measurements	1
StdDev	Off
Motion Correction	None
Save Original Images	On

Inline - MIP

MIP Sag	Off
MIP Cor	Off
MIP Tra	Off
MIP Time	Off
Radial MIP	Off
Save Original Images	On
MPR Sag	Off
MPR Cor	Off
MPR Tra	Off

Inline - Composing

Inline Composing	Off

Inline - Open Recon

Algorithm	None

Sequence - Part 1

Sequence Name	tseR_rr
Dimension	2D
RF Pulse Type	Fast
Gradient Mode	Fast
Flow Compensation	Read
Bandwidth	181 Hz/Px
Echo Spacing	11.8 ms
Free Echo Spacing	Off
Define	Turbo Factor
Turbo Factor	17
Echo Trains per Slice	35

Sequence - Part 2

Introduction	On
Phase Correction	Automatic
Compensate T2 Decay	Off
Fast Mode	Off
WARP	Off
Red. EC Sensitivity	Off
Acoustic noise reduction	Off
Reduce Motion Sens.	On
Motion Correction	None

Sequence - Assistant

SAR Assistant	Off
Allowed Delay	60 s

\\SAHMRI_Research\Head\AEP\AEP MRI ACQUISITION PROTOCOL Cima_v21\T2w_AxOblTempPole_RL- *
TA: 6:53 min Coil Selection: Auto Voxel Size: 0.3×0.3×0.5 mm³ Acc:: 4 Rel. SNR: 1.00

Properties

Start measurement without further preparation	On
Wait for User to Start	Off
Start measurements	Single Measurement
Prio Recon	Off
Auto Open Inline Display	Off
Auto Close Inline Display	Off
Load Images to MR View&GO	On
Auto Store Images	On
Disable auto transfer to PACS	Off
Load Images to Stamp Segments	Off
Load Images to Graphic Segments	Off
Graphic segment	Default
Inline Movie	Off

Routine

Slab Group	1
Slabs	1
Position	Isocenter
Orientation	T > C-20.0
Phase Encoding Dir.	R >> L
Slices per Slab	72
Phase Oversampling	60 %
Slice Oversampling	44.4 %
FOV Read	160 mm
FOV Phase	100.0 %
Slice Thickness	0.50 mm
TR	1400.0 ms
TE	156.00 ms
Averages	2.0
Concatenations	1
AutoAlign	---

Contrast - Common

TR	1400.0 ms
TE	156.00 ms
MTC	Off
Magn. Preparation	None
Flip Angle Mode	Constant
Flip Angle 1	120 deg
Flip Angle 2	90 deg
Fat-Water Contrast	Standard
Dark Blood	Off
Blood Suppression	Off
Wrap-up Magn.	Restore
Reconstruction	Magnitude

Contrast - Dynamic

Dynamic Mode	Standard
Measurements	1
Multiple Series	Each Measurement

Contrast - Dynamic

Reordering	Linear

Resolution - Common

FOV Read	160 mm
FOV Phase	100.0 %
Slice Thickness	0.50 mm
Base Resolution	320
Phase Resolution	100 %
Slice Resolution	100 %
Interpolation	On

Resolution - Acceleration

Acceleration Mode	CAIPIRINHA
Total Factor	4
Reference Scans	Integrated
Acceleration Factor PE	2
Reference Lines PE	32
Acceleration Factor 3D	2
Reference Lines 3D	32
Reordering Shift 3D	1
Phase Partial Fourier	Allowed
Slice Partial Fourier	Off
Elliptical Scanning	Off

Resolution - Filter

Raw Filter	On
Elliptical Filter	Off
Distortion Correction	2D
Normalize	Prescan
Image Filter	Off

Geometry - Common

Slab Group	1
Slabs	1
Position	Isocenter
Orientation	T > C-20.0
Phase Encoding Dir.	R >> L
Slices per Slab	72
Phase Oversampling	60 %
Slice Oversampling	44.4 %
FOV Read	160 mm
FOV Phase	100.0 %
Slice Thickness	0.50 mm
TR	1400.0 ms
Concatenations	1

Geometry - AutoAlign

Slab Group	1
Position	Isocenter
Orientation	T > C-20.0

Geometry - AutoAlign

Phase Encoding Dir.	R >> L
AutoAlign	---
Initial Position	Isocenter
L	0.0 mm
P	0.0 mm
H	0.0 mm
Initial Orientation	T > C
T > C	-20.00
> S	0.00
Initial Rotation	90.00 deg

Geometry - Navigator

Geometry - Saturation

Special Saturation	None

Geometry - Tim Planning Suite

Set-n-Go Protocol	Off
Table Position	0 mm
Table Position	H
Inline Composing	Off

System - Miscellaneous

Coil Selection	Auto Coil Select
Radial Sorting	Off
MSMA	S - C - T
Sagittal	R >> L
Coronal	A >> P
Transversal	F >> H
Coil Combination	Adaptive Combine
Matrix Optimization	Off
Coil Focus	Flat

System - Adjustments

Adjustment Strategy	Standard
B0 Shim	Standard
B1 Shim	TrueForm
CoilShim	Off
Adjustment Tolerance	Auto
Adjust with Body Coil	Off
Confirm Frequency	Never
Assume Silicone	Off

System - Adjust Volume

Position	Isocenter
Orientation	T > C-20.0
Rotation	90.00 deg
R >> L	160 mm
A >> P	160 mm
F >> H	36 mm
Reset	Off

System - pTx

B1 Shim	TrueForm

System - pTx

Excitation	Slab-sel.

System - Tx/Rx

Frequency 1H	123.256549 MHz
? Ref. Amplitude 1H	0.000 V
Reset	Off
Image Scaling	1.000
Gain	High

Physio - Signal

1st Signal/Mode	None
Trigger Delay	0 ms
TR	1400.0 ms
Concatenations	1

Physio - Cardiac

Fat-Water Contrast	Standard
Magn. Preparation	None
Dark Blood	Off
FOV Read	160 mm
FOV Phase	100.0 %
Phase Resolution	100 %

Physio - PACE

Resp. Control	Off
Concatenations	1

Inline - Subtraction

Subtract	Off
Measurements	1
StdDev	Off
Save Original Images	On

Inline - MIP

MIP Sag	Off
MIP Cor	Off
MIP Tra	Off
MIP Time	Off
Radial MIP	Off
Save Original Images	On
MPR Sag	Off
MPR Cor	Off
MPR Tra	Off

Inline - Composing

Inline Composing	Off

Inline - Open Recon

Algorithm	None

Sequence - Part 1

Sequence Name	spcR
Dimension	3D
Excitation	Slab-sel.

Sequence - Part 1

RF Pulse Type	Normal
Gradient Mode	Normal
Flow Compensation	None
Reordering	Linear
Bandwidth	289 Hz/Px
Echo Spacing	6.88 ms
Turbo Factor	96
Echo Train Duration	482 ms

Sequence - Part 2

Introduction	On

Sequence - Assistant

SAR Assistant	Off
Allowed Delay	0 s

\\SAHMRI_Research\Head\AEP\AEP MRI ACQUISITION PROTOCOL Cima_v21\DWI_Ax_AP_52Dir- *
TA: 2:58 min Coil Selection: Auto Voxel Size: 1.8×1.8×1.8 mm³ Acc:: None Rel. SNR: 1.00

Properties

Start measurement without further preparation	On
Wait for User to Start	Off
Start measurements	Single Measurement
Prio Recon	Off
Auto Open Inline Display	Off
Auto Close Inline Display	Off
Load Images to MR View&GO	On
Auto Store Images	On
Disable auto transfer to PACS	Off
Load Images to Stamp Segments	Off
Load Images to Graphic Segments	Off
Graphic segment	Default
Inline Movie	Off

Routine

Slice Group	1
Slices	84
Distance Factor	0 %
Position	R1.1 P6.9 F25.5 mm
Orientation	Transversal
Phase Encoding Dir.	A >> P
Phase Oversampling	0 %
FOV Read	230 mm
FOV Phase	100.0 %
Slice Thickness	1.8 mm
TR	3065.0 ms
TE	83.00 ms
Multi-band accel. factor	4
AutoAlign	---

Contrast - Common

TR	3065.0 ms
TE	83.00 ms
MTC	Off
Magn. Preparation	None
Flip Angle	90 deg
Refocus flip angle	180 deg
Fat-Water Contrast	Standard
Grad. rev. fat suppr.	Enabled
Reconstruction	Magn./Phase

Contrast - Dynamic

Dynamic Mode	Standard
Multiple Series	Off
Delay in TR	0.00 ms

Resolution - Common

FOV Read	230 mm
FOV Phase	100.0 %
Slice Thickness	1.8 mm
Base Resolution	128
Phase Resolution	100 %
Interpolation	Off

Resolution - Acceleration

Acceleration Mode	None
Phase Partial Fourier	6/8

Resolution - Filter

Raw Filter	Off
Elliptical Filter	Off
Distortion Correction	Off
Static Field Correction	Off
Normalize	Prescan
Noise Masking	Off

Geometry - Common

Slice Group	1
Slices	84
Distance Factor	0 %
Position	R1.1 P6.9 F25.5 mm
Orientation	Transversal
Phase Encoding Dir.	A >> P
Phase Oversampling	0 %
FOV Read	230 mm
FOV Phase	100.0 %
Slice Thickness	1.8 mm
TR	3065.0 ms
Multi-Slice Mode	Interleaved
Series	Interleaved
Multi-band accel. factor	4

Geometry - AutoAlign

Slice Group	1
Position	R1.1 P6.9 F25.5 mm
Orientation	Transversal
Phase Encoding Dir.	A >> P
AutoAlign	---
Initial Position	R1.1 P6.9 F25.5
R	1.1 mm
P	6.9 mm
F	25.5 mm
Initial Orientation	Transversal
Initial Rotation	0.00 deg

Geometry - Navigator

Geometry - Saturation

Special Saturation	None

Geometry - Tim Planning Suite

Set-n-Go Protocol	Off
Table Position	0 mm
Table Position	H
Inline Composing	Off

System - Miscellaneous

Coil Selection	Auto Coil Select
Radial Sorting	Off
MSMA	S - C - T
Sagittal	R >> L
Coronal	A >> P
Transversal	F >> H
Coil Combination	Sum of Squares
Matrix Optimization	Off
Coil Focus	Flat

System - Adjustments

Adjustment Strategy	Standard
B0 Shim	Standard
B1 Shim	TrueForm
CoilShim	Off
Adjustment Tolerance	Auto
Adjust with Body Coil	Off
Confirm Frequency	Never
Assume Silicone	Off

System - Adjust Volume

Position	R1.1 P6.9 F25.5 mm
Orientation	Transversal
Rotation	0.00 deg
A >> P	230 mm
R >> L	230 mm
F >> H	152 mm
Reset	Off

System - pTx

B1 Shim	TrueForm
Excitation	Standard

System - Tx/Rx

Frequency 1H	123.256549 MHz
? Ref. Amplitude 1H	0.000 V
Reset	Off
Image Scaling	1.000

Physio - Signal

1st Signal/Mode	None
TR	3065.0 ms
Multi-band accel. factor	4

Physio - PACE

Resp. Control	Off
Multi-band accel. factor	4

Diff

Diffusion Mode	Free
Diff. Directions	52
Diffusion Scheme	Monopolar
Diff. Weightings	2
b-value 1	0 s/mm²
b-value 2	3000 s/mm²
Averages 1	1
Averages 2	1
Dynamic Field Correction	Off
Invert Gray Scale	Off
Diff. Weighted Images	On
Trace Weighted Images	Off
Tensor	Off
FA Maps	Off
ADC Maps	Off
Exponential ADC Maps	Off
Noise Masking	Off
Calculated Image	Off

Inline - Open Recon

Algorithm	None

Sequence - Part 1

Sequence Name	epse
Dimension	2D
Excitation	Standard
Gradient Mode	Performance
Bandwidth	2056 Hz/Px
Echo Spacing	0.59 ms
Free Echo Spacing	Off
EPI Factor	128

Sequence - Part 2

Introduction	Off
RF Spoiling	Off
Phase Correction	Internal

Sequence - Special

Excite pulse duration	5120 us
Refocus pulse duration	10240 us
Min. prep scans	0
Min. prep scans SB	0
Delay before PC scans	0 us
Single-band images	On
MB LeakBlock kernel	On
MB dual kernel	Off
MB RF phase scramble	Off
Opt. MB RF pulse BW	Off
Time-shifted MB RF	Off
SENSE1 coil combine	On
Invert RO/PE polarity	Off
Force Maxwell corr.	Off
PF omits higher k-space	Off
Disable B1 control loop	Off

Sequence - Special

Disable freq. update	Off
Suppress 16-bit DICOM	Off
Force equal slice timing	Off
Online multi-band recon.	Online
FFT scale factor	1.00
Physio recording	DICOM

Sequence - Assistant

SAR Assistant	Off

\\SAHMRI_Research\Head\AEP\AEP MRI ACQUISITION PROTOCOL Cima_v21\DWI_Ax_AP_51Dir_InvROPE - *

TA: 2:55 min Coil Selection: Auto Voxel Size: 1.8×1.8×1.8 mm³ Acc:: None Rel. SNR: 1.00

Properties

Start measurement without further preparation	On
Wait for User to Start	Off
Start measurements	Single Measurement
Prio Recon	Off
Auto Open Inline Display	Off
Auto Close Inline Display	Off
Load Images to MR View&GO	On
Auto Store Images	On
Disable auto transfer to PACS	Off
Load Images to Stamp Segments	Off
Load Images to Graphic Segments	Off
Graphic segment	Default
Inline Movie	Off

Routine

Slice Group	1
Slices	84
Distance Factor	0 %
Position	R1.1 P6.9 F25.5 mm
Orientation	Transversal
Phase Encoding Dir.	A >> P
Phase Oversampling	0 %
FOV Read	230 mm
FOV Phase	100.0 %
Slice Thickness	1.8 mm
TR	3065.0 ms
TE	83.00 ms
Multi-band accel. factor	4
AutoAlign	---

Contrast - Common

TR	3065.0 ms
TE	83.00 ms
MTC	Off
Magn. Preparation	None
Flip Angle	90 deg
Refocus flip angle	180 deg
Fat-Water Contrast	Standard
Grad. rev. fat suppr.	Enabled
Reconstruction	Magn./Phase

Contrast - Dynamic

Dynamic Mode	Standard
Multiple Series	Off
Delay in TR	0.00 ms

Resolution - Common

FOV Read	230 mm

Resolution - Common

FOV Phase	100.0 %
Slice Thickness	1.8 mm
Base Resolution	128
Phase Resolution	100 %
Interpolation	Off

Resolution - Acceleration

Acceleration Mode	None
Phase Partial Fourier	6/8

Resolution - Filter

Raw Filter	Off
Elliptical Filter	Off
Distortion Correction	Off
Static Field Correction	Off
Normalize	Prescan
Noise Masking	Off

Geometry - Common

Slice Group	1
Slices	84
Distance Factor	0 %
Position	R1.1 P6.9 F25.5 mm
Orientation	Transversal
Phase Encoding Dir.	A >> P
Phase Oversampling	0 %
FOV Read	230 mm
FOV Phase	100.0 %
Slice Thickness	1.8 mm
TR	3065.0 ms
Multi-Slice Mode	Interleaved
Series	Interleaved
Multi-band accel. factor	4

Geometry - AutoAlign

Slice Group	1
Position	R1.1 P6.9 F25.5 mm
Orientation	Transversal
Phase Encoding Dir.	A >> P
AutoAlign	---
Initial Position	R1.1 P6.9 F25.5
R	1.1 mm
P	6.9 mm
F	25.5 mm
Initial Orientation	Transversal
Initial Rotation	0.00 deg

Geometry - Navigator

Geometry - Saturation

Special Saturation	None

Geometry - Tim Planning Suite

Set-n-Go Protocol	Off
Table Position	0 mm
Table Position	H
Inline Composing	Off

System - Miscellaneous

Coil Selection	Auto Coil Select
Radial Sorting	Off
MSMA	S - C - T
Sagittal	R >> L
Coronal	A >> P
Transversal	F >> H
Coil Combination	Sum of Squares
Matrix Optimization	Off
Coil Focus	Flat

System - Adjustments

Adjustment Strategy	Standard
B0 Shim	Standard
B1 Shim	TrueForm
CoilShim	Off
Adjustment Tolerance	Auto
Adjust with Body Coil	Off
Confirm Frequency	Never
Assume Silicone	Off

System - Adjust Volume

Position	R1.1 P6.9 F25.5 mm
Orientation	Transversal
Rotation	0.00 deg
A >> P	230 mm
R >> L	230 mm
F >> H	152 mm
Reset	Off

System - pTx

B1 Shim	TrueForm
Excitation	Standard

System - Tx/Rx

Frequency 1H	123.256549 MHz
? Ref. Amplitude 1H	0.000 V
Reset	Off
Image Scaling	1.000

Physio - Signal

1st Signal/Mode	None
TR	3065.0 ms
Multi-band accel. factor	4

Physio - PACE

Resp. Control	Off
Multi-band accel. factor	4

Diff

Diffusion Mode	Free
Diff. Directions	51
Diffusion Scheme	Monopolar
Diff. Weightings	2
b-value 1	0 s/mm²
b-value 2	3000 s/mm²
Averages 1	1
Averages 2	1
Dynamic Field Correction	Off
Invert Gray Scale	Off
Diff. Weighted Images	On
Trace Weighted Images	Off
Tensor	Off
FA Maps	Off
ADC Maps	Off
Exponential ADC Maps	Off
Noise Masking	Off
Calculated Image	Off

Inline - Open Recon

Algorithm	None

Sequence - Part 1

Sequence Name	epse
Dimension	2D
Excitation	Standard
Gradient Mode	Performance
Bandwidth	2056 Hz/Px
Echo Spacing	0.59 ms
Free Echo Spacing	Off
EPI Factor	128

Sequence - Part 2

Introduction	Off
RF Spoiling	Off
Phase Correction	Internal

Sequence - Special

Excite pulse duration	5120 us
Refocus pulse duration	10240 us
Min. prep scans	0
Min. prep scans SB	0
Delay before PC scans	0 us
Single-band images	On
MB LeakBlock kernel	On
MB dual kernel	Off
MB RF phase scramble	Off
Opt. MB RF pulse BW	Off
Time-shifted MB RF	Off
SENSE1 coil combine	On

Sequence - Special

Invert RO/PE polarity	On
Force Maxwell corr.	Off
PF omits higher k-space	Off
Disable B1 control loop	Off
Disable freq. update	Off
Suppress 16-bit DICOM	Off
Force equal slice timing	Off
Online multi-band recon.	Online
FFT scale factor	1.00
Physio recording	DICOM

Sequence - Assistant

SAR Assistant	Off

\\SAHMRI_Research\Head\AEP\AEP MRI ACQUISITION PROTOCOL Cima_v21\T1map_MP2RAGE_Sag_AP-*

TA: 7:55 min Coil Selection: Auto Voxel Size: 1.0×1.0×1.0 mm³ Acc:: 3 Rel. SNR: 1.00

Properties

Start measurement without further preparation	On
Wait for User to Start	Off
Start measurements	Single Measurement
Prio Recon	Off
Auto Open Inline Display	Off
Auto Close Inline Display	Off
Load Images to MR View&GO	On
Auto Store Images	On
Disable auto transfer to PACS	Off
Load Images to Stamp Segments	Off
Load Images to Graphic Segments	Off
Graphic segment	Default
Inline Movie	Off

Routine

Slab Group	1
Slabs	1
Distance Factor	50 %
Position	Isocenter
Orientation	Sagittal
Phase Encoding Dir.	A >> P
Slices per Slab	208
Phase Oversampling	0 %
Slice Oversampling	0.0 %
FOV Read	256 mm
FOV Phase	93.8 %
Slice Thickness	1.00 mm
TR	5000.0 ms
TE	2.83 ms
Averages	1
Concatenations	1
AutoAlign	---

Contrast - Common

TR	5000.0 ms
TE	2.83 ms
Magn. Preparation	Non-sel. IR
TI 1	700 ms
TI 2	2500 ms
Flip Angle 1	4 deg
Flip Angle 2	5 deg
Fat-Water Contrast	Standard
Dark Blood	Off
Contrasts	1
Reconstruction	Magnitude

Contrast - Dynamic

Dynamic Mode	Standard
Measurements	1
Multiple Series	Each Measurement
Reordering	Linear

Resolution - Common

FOV Read	256 mm
FOV Phase	93.8 %
Slice Thickness	1.00 mm
Base Resolution	256
Phase Resolution	100 %
Slice Resolution	100 %
Interpolation	Off

Resolution - Acceleration

Acceleration Mode	GRAPPA
Reference Scans	Integrated
Acceleration Factor PE	3
Reference Lines PE	24
Acceleration Factor 3D	1
Phase Partial Fourier	Off
Slice Partial Fourier	Off
Asymmetric Echo	Off
Elliptical Scanning	Off

Resolution - Filter

Raw Filter	Off
Elliptical Filter	Off
Distortion Correction	3D
Normalize	Prescan
Image Filter	Off

Geometry - Common

Slab Group	1
Slabs	1
Distance Factor	50 %
Position	Isocenter
Orientation	Sagittal
Phase Encoding Dir.	A >> P
Slices per Slab	208
Phase Oversampling	0 %
Slice Oversampling	0.0 %
FOV Read	256 mm
FOV Phase	93.8 %
Slice Thickness	1.00 mm
TR	5000.0 ms
Multi-Slice Mode	Single Shot
Series	Interleaved
Concatenations	1

Geometry - AutoAlign

Slab Group	1
Position	Isocenter
Orientation	Sagittal
Phase Encoding Dir.	A >> P
AutoAlign	---
Initial Position	Isocenter
L	0.0 mm
P	0.0 mm
H	0.0 mm
Initial Orientation	Sagittal
Initial Rotation	0.00 deg

Geometry - Navigator

Geometry - Tim Planning Suite

Set-n-Go Protocol	Off
Table Position	0 mm
Table Position	H
Inline Composing	Off

System - Miscellaneous

Coil Selection	Auto Coil Select
Radial Sorting	Off
MSMA	S - C - T
Sagittal	R >> L
Coronal	P >> A
Transversal	F >> H
Coil Combination	Adaptive Combine
Matrix Optimization	Off
Coil Focus	Flat

System - Adjustments

Adjustment Strategy	Standard
B0 Shim	Standard
B1 Shim	TrueForm
CoilShim	Off
Adjustment Tolerance	Auto
Adjust with Body Coil	Off
Confirm Frequency	Never
Assume Silicone	Off

System - Adjust Volume

Position	Isocenter
Orientation	Sagittal
Rotation	0.00 deg
A >> P	240 mm
F >> H	256 mm
R >> L	208 mm
Reset	Off

System - pTx

B1 Shim	TrueForm
Excitation	Non-sel.

System - Tx/Rx

Frequency 1H	123.256549 MHz
? Ref. Amplitude 1H	0.000 V
Reset	Off
Image Scaling	1.000

Physio - Signal

1st Signal/Mode	None
TR	5000.0 ms
Concatenations	1

Physio - Cardiac

Fat-Water Contrast	Standard
Magn. Preparation	Non-sel. IR
TI 1	700 ms
TI 2	2500 ms
Dark Blood	Off
FOV Read	256 mm
FOV Phase	93.8 %
Phase Resolution	100 %
Motion Correction	None

Physio - PACE

Resp. Control	Off
Concatenations	1

Inline - Subtraction

Subtract	Off
Measurements	1
StdDev	Off
Save Original Images	On

Inline - MIP

MIP Sag	Off
MIP Cor	Off
MIP Tra	Off
MIP Time	Off
Radial MIP	Off
Save Original Images	On
MPR Sag	Off
MPR Cor	Off
MPR Tra	Off

Inline - Composing

Inline Composing	Off

Inline - MapIt

MapIt	T1 Map
Flip Angle 1	4 deg
Flip Angle 2	5 deg
Measurements	1
Contrasts	1
TE	2.83 ms
TR	5000.0 ms
Save Original Images	On

Inline - Open Recon

Algorithm	None

Sequence - Part 1

Sequence Name	tfl
Dimension	3D
Excitation	Non-sel.
RF Pulse Type	Fast
Gradient Mode	Performance
Flow Compensation	None
Reordering	Linear
Bandwidth	250 Hz/Px
Echo Spacing	6.42 ms
Asymmetric Echo	Off
Turbo Factor	208

Sequence - Part 2

Introduction	Off
RF Spoiling	On
Incr. Gradient Spoiling	Off
Motion Correction	None

Sequence - Assistant

SAR Assistant	Off

\\SAHMRI_Research\Head\AEP\AEP MRI ACQUISITION PROTOCOL Cima_v21\fMRI_AxObl_AP_Pseudoword- *

TA: 3:20 min Coil Selection: Auto Voxel Size: 3.0×3.0×3.0 mm³ Acc:: 2 Rel. SNR: 1.00

Properties

Start measurement without further preparation	Off
Wait for User to Start	Off
Start measurements	Single Measurement
Prio Recon	Off
Auto Open Inline Display	Off
Auto Close Inline Display	Off
Load Images to MR View&GO	On
Auto Store Images	On
Disable auto transfer to PACS	Off
Load Images to Stamp Segments	Off
Load Images to Graphic Segments	Off
Graphic segment	Default
Inline Movie	Off

Routine

Slice Group	1
Slices	44
Distance Factor	0 %
Position	R1.1 P6.9 F25.5 mm
Orientation	Transversal
Phase Encoding Dir.	A >> P
Phase Oversampling	0 %
FOV Read	216 mm
FOV Phase	111.1 %
Slice Thickness	3.0 mm
TR	900.0 ms
TE 1	13.40 ms
TE 2	32.96 ms
TE 3	52.52 ms
Averages	1
Multi-band accel. factor	4
AutoAlign	---

Contrast - Common

TR	900.0 ms
TE 1	13.40 ms
TE 2	32.96 ms
TE 3	52.52 ms
MTC	Off
Magn. Preparation	None
Flip Angle	30 deg
Fat-Water Contrast	Fat Saturation
Contrasts	3
Reconstruction	Magn./Phase

Contrast - Dynamic

Dynamic Mode	Standard
Measurements	202

Contrast - Dynamic

Delay in TR	0.00 ms

Resolution - Common

FOV Read	216 mm
FOV Phase	111.1 %
Slice Thickness	3.0 mm
Base Resolution	72
Phase Resolution	100 %
Interpolation	Off

Resolution - Acceleration

Acceleration Mode	GRAPPA
Reference scan mode	Single-shot
Acceleration Factor PE	2
Reference Lines PE	24
Phase Partial Fourier	7/8

Resolution - Filter

Raw Filter	Off
Elliptical Filter	Off
Hamming	Off
Distortion Correction	Off
Static Field Correction	Off
Normalize	Prescan

Geometry - Common

Slice Group	1
Slices	44
Distance Factor	0 %
Position	R1.1 P6.9 F25.5 mm
Orientation	Transversal
Phase Encoding Dir.	A >> P
Phase Oversampling	0 %
FOV Read	216 mm
FOV Phase	111.1 %
Slice Thickness	3.0 mm
TR	900.0 ms
Multi-Slice Mode	Interleaved
Series	Interleaved
Multi-band accel. factor	4

Geometry - AutoAlign

Slice Group	1
Position	R1.1 P6.9 F25.5 mm
Orientation	Transversal
Phase Encoding Dir.	A >> P
AutoAlign	---
Initial Position	R1.1 P6.9 F25.5
R	1.1 mm

Geometry - AutoAlign

P	6.9 mm
F	25.5 mm
Initial Orientation	Transversal
Initial Rotation	0.00 deg

Geometry - Saturation

Special Saturation	None

Geometry - Tim Planning Suite

Set-n-Go Protocol	Off
Table Position	0 mm
Table Position	H
Inline Composing	Off

System - Miscellaneous

Coil Selection	Auto Coil Select
Radial Sorting	Off
MSMA	S - C - T
Sagittal	R >> L
Coronal	A >> P
Transversal	F >> H
Coil Combination	Sum of Squares
Matrix Optimization	Off
Coil Focus	Flat

System - Adjustments

Adjustment Strategy	Standard
B0 Shim	Standard
B1 Shim	TrueForm
CoilShim	Off
Adjustment Tolerance	Auto
Adjust with Body Coil	Off
Confirm Frequency	Never
Assume Silicone	Off

System - Adjust Volume

Position	R1.1 P6.9 F25.5 mm
Orientation	Transversal
Rotation	90.00 deg
R >> L	216 mm
A >> P	240 mm
F >> H	132 mm
Reset	Off

System - pTx

B1 Shim	TrueForm
Excitation	Standard

System - Tx/Rx

Frequency 1H	123.256549 MHz
? Ref. Amplitude 1H	0.000 V
Reset	Off
Image Scaling	1.000

Physio - Signal

1st Signal/Mode	None
TR	900.0 ms
Multi-band accel. factor	4

BOLD

GLM Statistics	Off
Ignore Meas. at Start	0
Ignore After Transition	0
Model Transition States	Off
Temp. Highpass Filter	Off
Threshold	4.00
Paradigm Size	201
Meas[1]	Baseline
Meas[2]	Baseline
Meas[3]	Baseline
Meas[4]	Baseline
Meas[5]	Baseline
Meas[6]	Baseline
Meas[7]	Baseline
Meas[8]	Baseline
Meas[9]	Baseline
Meas[10]	Baseline
Meas[11]	Active
Meas[12]	Active
Meas[13]	Active
Meas[14]	Active
Meas[15]	Active
Meas[16]	Active
Meas[17]	Active
Meas[18]	Active
Meas[19]	Active
Meas[20]	Active
Meas[21]	Active
Meas[22]	Active
Meas[23]	Active
Meas[24]	Active
Meas[25]	Active
Meas[26]	Active
Meas[27]	Active
Meas[28]	Active
Meas[29]	Active
Meas[30]	Active
Meas[31]	Baseline
Meas[32]	Baseline
Meas[33]	Baseline
Meas[34]	Baseline
Meas[35]	Baseline
Meas[36]	Baseline
Meas[37]	Baseline
Meas[38]	Baseline
Meas[39]	Baseline
Meas[40]	Baseline
Meas[41]	Baseline
Meas[42]	Baseline

BOLD

Meas[43]	Baseline
Meas[44]	Baseline
Meas[45]	Baseline
Meas[46]	Baseline
Meas[47]	Baseline
Meas[48]	Baseline
Meas[49]	Baseline
Meas[50]	Baseline
Meas[51]	Active
Meas[52]	Active
Meas[53]	Active
Meas[54]	Active
Meas[55]	Active
Meas[56]	Active
Meas[57]	Active
Meas[58]	Active
Meas[59]	Active
Meas[60]	Active
Meas[61]	Active
Meas[62]	Active
Meas[63]	Active
Meas[64]	Active
Meas[65]	Active
Meas[66]	Active
Meas[67]	Active
Meas[68]	Active
Meas[69]	Active
Meas[70]	Active
Meas[71]	Baseline
Meas[72]	Baseline
Meas[73]	Baseline
Meas[74]	Baseline
Meas[75]	Baseline
Meas[76]	Baseline
Meas[77]	Baseline
Meas[78]	Baseline
Meas[79]	Baseline
Meas[80]	Baseline
Meas[81]	Baseline
Meas[82]	Baseline
Meas[83]	Baseline
Meas[84]	Baseline
Meas[85]	Baseline
Meas[86]	Baseline
Meas[87]	Baseline
Meas[88]	Baseline
Meas[89]	Baseline
Meas[90]	Baseline
Meas[91]	Active
Meas[92]	Active
Meas[93]	Active
Meas[94]	Active
Meas[95]	Active
Meas[96]	Active

BOLD

Meas[97]	Active
Meas[98]	Active
Meas[99]	Active
Meas[100]	Active
Meas[101]	Active
Meas[102]	Active
Meas[103]	Active
Meas[104]	Active
Meas[105]	Active
Meas[106]	Active
Meas[107]	Active
Meas[108]	Active
Meas[109]	Active
Meas[110]	Active
Meas[111]	Baseline
Meas[112]	Baseline
Meas[113]	Baseline
Meas[114]	Baseline
Meas[115]	Baseline
Meas[116]	Baseline
Meas[117]	Baseline
Meas[118]	Baseline
Meas[119]	Baseline
Meas[120]	Baseline
Meas[121]	Baseline
Meas[122]	Baseline
Meas[123]	Baseline
Meas[124]	Baseline
Meas[125]	Baseline
Meas[126]	Baseline
Meas[127]	Baseline
Meas[128]	Baseline
Meas[129]	Baseline
Meas[130]	Baseline
Meas[131]	Active
Meas[132]	Active
Meas[133]	Active
Meas[134]	Active
Meas[135]	Active
Meas[136]	Active
Meas[137]	Active
Meas[138]	Active
Meas[139]	Active
Meas[140]	Active
Meas[141]	Active
Meas[142]	Active
Meas[143]	Active
Meas[144]	Active
Meas[145]	Active
Meas[146]	Active
Meas[147]	Active
Meas[148]	Active
Meas[149]	Active
Meas[150]	Active

BOLD

Meas[151]	Baseline
Meas[152]	Baseline
Meas[153]	Baseline
Meas[154]	Baseline
Meas[155]	Baseline
Meas[156]	Baseline
Meas[157]	Baseline
Meas[158]	Baseline
Meas[159]	Baseline
Meas[160]	Baseline
Meas[161]	Baseline
Meas[162]	Baseline
Meas[163]	Baseline
Meas[164]	Baseline
Meas[165]	Baseline
Meas[166]	Baseline
Meas[167]	Baseline
Meas[168]	Baseline
Meas[169]	Baseline
Meas[170]	Baseline
Meas[171]	Active
Meas[172]	Active
Meas[173]	Active
Meas[174]	Active
Meas[175]	Active
Meas[176]	Active
Meas[177]	Active
Meas[178]	Active
Meas[179]	Active
Meas[180]	Active
Meas[181]	Active
Meas[182]	Active
Meas[183]	Active
Meas[184]	Active
Meas[185]	Active
Meas[186]	Active
Meas[187]	Active
Meas[188]	Active
Meas[189]	Active
Meas[190]	Active
Meas[191]	Baseline
Meas[192]	Baseline
Meas[193]	Baseline
Meas[194]	Baseline
Meas[195]	Baseline
Meas[196]	Baseline
Meas[197]	Baseline
Meas[198]	Baseline
Meas[199]	Baseline
Meas[200]	Baseline
Meas[201]	Baseline
Motion Correction	Off
Spatial Filter	Off
Measurements	202

BOLD

Delay in TR	0.00 ms

Inline - Open Recon

Algorithm	None

Sequence - Part 1

Sequence Name	epfid
Dimension	2D
Excitation	Standard
Gradient Mode	Performance
Flow Compensation	None
Bandwidth	2314 Hz/Px
Echo Spacing	0.54 ms
Free Echo Spacing	Off
EPI Factor	80

Sequence - Part 2

Introduction	Off
RF Spoiling	Off

Sequence - Special

Excite pulse duration	5120 us
Min. prep scans	0
Min. prep scans SB	0
Inter-TE delay	0 us
Delay before PC scans	0 us
Single-band images	On
MB LeakBlock kernel	On
MB dual kernel	Off
MB RF phase scramble	Off
Opt. MB RF pulse BW	Off
SENSE1 coil combine	On
Invert RO/PE polarity	Off
Force Maxwell corr.	Off
PF omits higher k-space	Off
Disable B1 control loop	Off
Disable freq. update	Off
Suppress 16-bit DICOM	Off
Force equal slice timing	Off
Online multi-band recon.	Online
FFT scale factor	1.00
Fat saturation FA	110.00 deg
Fat sat. offset	0.00 Hz
Sinc exc. pulse BWTP	5.20
Physio recording	DICOM
Triggering scheme	Standard

Sequence - Assistant

SAR Assistant	Off

\\SAHMRI_Research\Head\AEP\AEP MRI ACQUISITION PROTOCOL Cima_v21\FMap_AxObl_AP- *
TA: 9 sec Coil Selection: Auto Voxel Size: 2.4×2.4×2.0 mm³ Acc:: None Rel. SNR: 1.00

Properties

Start measurement without further preparation	On
Wait for User to Start	Off
Start measurements	Single Measurement
Prio Recon	Off
Auto Open Inline Display	Off
Auto Close Inline Display	Off
Load Images to MR View&GO	On
Auto Store Images	On
Disable auto transfer to PACS	Off
Load Images to Stamp Segments	Off
Load Images to Graphic Segments	Off
Graphic segment	Default
Inline Movie	Off

Routine

Slice Group	1
Slices	72
Distance Factor	0 %
Position	R1.1 P6.9 F25.5 mm
Orientation	Transversal
Phase Encoding Dir.	A >> P
Phase Oversampling	0 %
FOV Read	264 mm
FOV Phase	100.0 %
Slice Thickness	2.0 mm
TR	8500.0 ms
TE	70.00 ms
Averages	1
Multi-band accel. factor	1
AutoAlign	---

Contrast - Common

TR	8500.0 ms
TE	70.00 ms
MTC	Off
Magn. Preparation	None
Flip Angle	90 deg
Refocus flip angle	180 deg
Fat-Water Contrast	Fat Saturation
Grad. rev. fat suppr.	Disabled
Contrasts	1
Reconstruction	Magnitude

Contrast - Dynamic

Dynamic Mode	Standard
Measurements	1
Delay in TR	0.00 ms

Resolution - Common

FOV Read	264 mm
FOV Phase	100.0 %
Slice Thickness	2.0 mm
Base Resolution	112
Phase Resolution	100 %
Interpolation	Off

Resolution - Acceleration

Acceleration Mode	None
Phase Partial Fourier	7/8

Resolution - Filter

Raw Filter	Off
Elliptical Filter	Off
Hamming	Off
Distortion Correction	Off
Static Field Correction	Off
Normalize	Prescan

Geometry - Common

Slice Group	1
Slices	72
Distance Factor	0 %
Position	R1.1 P6.9 F25.5 mm
Orientation	Transversal
Phase Encoding Dir.	A >> P
Phase Oversampling	0 %
FOV Read	264 mm
FOV Phase	100.0 %
Slice Thickness	2.0 mm
TR	8500.0 ms
Multi-Slice Mode	Interleaved
Series	Interleaved
Multi-band accel. factor	1

Geometry - AutoAlign

Slice Group	1
Position	R1.1 P6.9 F25.5 mm
Orientation	Transversal
Phase Encoding Dir.	A >> P
AutoAlign	---
Initial Position	R1.1 P6.9 F25.5
R	1.1 mm
P	6.9 mm
F	25.5 mm
Initial Orientation	Transversal
Initial Rotation	0.00 deg

Geometry - Saturation

Special Saturation	None

Geometry - Tim Planning Suite

Set-n-Go Protocol	Off
Table Position	0 mm
Table Position	H
Inline Composing	Off

System - Miscellaneous

Coil Selection	Auto Coil Select
Radial Sorting	Off
MSMA	S - C - T
Sagittal	R >> L
Coronal	A >> P
Transversal	F >> H
Coil Combination	Sum of Squares
Matrix Optimization	Off
Coil Focus	Flat

System - Adjustments

Adjustment Strategy	Standard
B0 Shim	Standard
B1 Shim	TrueForm
CoilShim	Off
Adjustment Tolerance	Auto
Adjust with Body Coil	Off
Confirm Frequency	Never
Assume Silicone	Off

System - Adjust Volume

Position	R1.1 P6.9 F25.5 mm
Orientation	Transversal
Rotation	0.00 deg
A >> P	264 mm
R >> L	264 mm
F >> H	144 mm
Reset	Off

System - pTx

B1 Shim	TrueForm
Excitation	Standard

System - Tx/Rx

Frequency 1H	123.256549 MHz
? Ref. Amplitude 1H	0.000 V
Reset	Off
Image Scaling	1.000

Physio - Signal

1st Signal/Mode	None
TR	8500.0 ms
Multi-band accel. factor	1

BOLD

GLM Statistics	Off
Ignore Meas. at Start	0
Ignore After Transition	0

BOLD

Model Transition States	On
Temp. Highpass Filter	On
Threshold	4.00
Paradigm Size	4
Meas[1]	Baseline
Meas[2]	Baseline
Meas[3]	Baseline
Meas[4]	Active
Motion Correction	Off
Spatial Filter	Off
Measurements	1
Delay in TR	0.00 ms

Inline - Open Recon

Algorithm	None

Sequence - Part 1

Sequence Name	epse
Dimension	2D
Excitation	Standard
RF Pulse Type	Normal
Gradient Mode	Performance
Bandwidth	2626 Hz/Px
Echo Spacing	0.54 ms
Free Echo Spacing	Off
EPI Factor	112

Sequence - Part 2

Introduction	Off

Sequence - Special

Min. prep scans	0
Delay before PC scans	0 us
SENSE1 coil combine	On
Invert RO/PE polarity	Off
PF omits higher k-space	Off
Disable B1 control loop	Off
Disable freq. update	Off
Suppress 16-bit DICOM	Off
Force equal slice timing	Off
FFT scale factor	1.00
Fat saturation FA	110.00 deg
Fat sat. offset	0.00 Hz
Physio recording	DICOM
Triggering scheme	Standard

Sequence - Assistant

SAR Assistant	Off

**\\SAHMRI_Research\Head\AEP\AEP MRI ACQUISITION PROTOCOL Cima_v21\FMap_AxObl_AP_InvROPE-*
**

TA: 9 sec Coil Selection: Auto Voxel Size: 2.4×2.4×2.0 mm³ Acc:: None Rel. SNR: 1.00

Properties

Start measurement without further preparation	On
Wait for User to Start	Off
Start measurements	Single Measurement
Prio Recon	Off
Auto Open Inline Display	Off
Auto Close Inline Display	Off
Load Images to MR View&GO	On
Auto Store Images	On
Disable auto transfer to PACS	Off
Load Images to Stamp Segments	Off
Load Images to Graphic Segments	Off
Graphic segment	Default
Inline Movie	Off

Routine

Slice Group	1
Slices	72
Distance Factor	0 %
Position	R1.1 P6.9 F25.5 mm
Orientation	Transversal
Phase Encoding Dir.	A >> P
Phase Oversampling	0 %
FOV Read	264 mm
FOV Phase	100.0 %
Slice Thickness	2.0 mm
TR	8500.0 ms
TE	70.00 ms
Averages	1
Multi-band accel. factor	1
AutoAlign	---

Contrast - Common

TR	8500.0 ms
TE	70.00 ms
MTC	Off
Magn. Preparation	None
Flip Angle	90 deg
Refocus flip angle	180 deg
Fat-Water Contrast	Fat Saturation
Grad. rev. fat suppr.	Disabled
Contrasts	1
Reconstruction	Magnitude

Contrast - Dynamic

Dynamic Mode	Standard
Measurements	1
Delay in TR	0.00 ms

Resolution - Common

FOV Read	264 mm
FOV Phase	100.0 %
Slice Thickness	2.0 mm
Base Resolution	112
Phase Resolution	100 %
Interpolation	Off

Resolution - Acceleration

Acceleration Mode	None
Phase Partial Fourier	7/8

Resolution - Filter

Raw Filter	Off
Elliptical Filter	Off
Hamming	Off
Distortion Correction	Off
Static Field Correction	Off
Normalize	Prescan

Geometry - Common

Slice Group	1
Slices	72
Distance Factor	0 %
Position	R1.1 P6.9 F25.5 mm
Orientation	Transversal
Phase Encoding Dir.	A >> P
Phase Oversampling	0 %
FOV Read	264 mm
FOV Phase	100.0 %
Slice Thickness	2.0 mm
TR	8500.0 ms
Multi-Slice Mode	Interleaved
Series	Interleaved
Multi-band accel. factor	1

Geometry - AutoAlign

Slice Group	1
Position	R1.1 P6.9 F25.5 mm
Orientation	Transversal
Phase Encoding Dir.	A >> P
AutoAlign	---
Initial Position	R1.1 P6.9 F25.5
R	1.1 mm
P	6.9 mm
F	25.5 mm
Initial Orientation	Transversal
Initial Rotation	0.00 deg

Geometry - Saturation

Special Saturation	None

Geometry - Tim Planning Suite

Set-n-Go Protocol	Off
Table Position	0 mm
Table Position	H
Inline Composing	Off

System - Miscellaneous

Coil Selection	Auto Coil Select
Radial Sorting	Off
MSMA	S - C - T
Sagittal	R >> L
Coronal	A >> P
Transversal	F >> H
Coil Combination	Sum of Squares
Matrix Optimization	Off
Coil Focus	Flat

System - Adjustments

Adjustment Strategy	Standard
B0 Shim	Standard
B1 Shim	TrueForm
CoilShim	Off
Adjustment Tolerance	Auto
Adjust with Body Coil	Off
Confirm Frequency	Never
Assume Silicone	Off

System - Adjust Volume

Position	R1.1 P6.9 F25.5 mm
Orientation	Transversal
Rotation	0.00 deg
A >> P	264 mm
R >> L	264 mm
F >> H	144 mm
Reset	Off

System - pTx

B1 Shim	TrueForm
Excitation	Standard

System - Tx/Rx

Frequency 1H	123.256549 MHz
? Ref. Amplitude 1H	0.000 V
Reset	Off
Image Scaling	1.000

Physio - Signal

1st Signal/Mode	None
TR	8500.0 ms
Multi-band accel. factor	1

BOLD

GLM Statistics	Off
Ignore Meas. at Start	0
Ignore After Transition	0
Model Transition States	On
Temp. Highpass Filter	On
Threshold	4.00
Paradigm Size	4
Meas[1]	Baseline
Meas[2]	Baseline
Meas[3]	Baseline
Meas[4]	Active
Motion Correction	Off
Spatial Filter	Off
Measurements	1
Delay in TR	0.00 ms

Inline - Open Recon

Algorithm	None

Sequence - Part 1

Sequence Name	epse
Dimension	2D
Excitation	Standard
RF Pulse Type	Normal
Gradient Mode	Performance
Bandwidth	2626 Hz/Px
Echo Spacing	0.54 ms
Free Echo Spacing	Off
EPI Factor	112

Sequence - Part 2

Introduction	Off

Sequence - Special

Min. prep scans	0
Delay before PC scans	0 us
SENSE1 coil combine	On
Invert RO/PE polarity	On
PF omits higher k-space	Off
Disable B1 control loop	Off
Disable freq. update	Off
Suppress 16-bit DICOM	Off
Force equal slice timing	Off
FFT scale factor	1.00
Fat saturation FA	110.00 deg
Fat sat. offset	0.00 Hz
Physio recording	DICOM
Triggering scheme	Standard

Sequence - Assistant

SAR Assistant	Off

**\\SAHMRI_Research\Head\AEP\AEP MRI ACQUISITION PROTOCOL Cima_v21\fMRI_AxObl_AP_OneBack-*

TA: 4:53 min Coil Selection: Auto Voxel Size: 3.0×3.0×3.0 mm³ Acc:: 2 Rel. SNR: 1.00

Properties

Start measurement without further preparation	Off
Wait for User to Start	Off
Start measurements	Single Measurement
Prio Recon	Off
Auto Open Inline Display	Off
Auto Close Inline Display	Off
Load Images to MR View&GO	On
Auto Store Images	On
Disable auto transfer to PACS	Off
Load Images to Stamp Segments	Off
Load Images to Graphic Segments	Off
Graphic segment	Default
Inline Movie	Off

Routine

Slice Group	1
Slices	44
Distance Factor	0 %
Position	R1.1 P6.9 F25.5 mm
Orientation	Transversal
Phase Encoding Dir.	A >> P
Phase Oversampling	0 %
FOV Read	216 mm
FOV Phase	111.1 %
Slice Thickness	3.0 mm
TR	900.0 ms
TE 1	13.40 ms
TE 2	32.96 ms
TE 3	52.52 ms
Averages	1
Multi-band accel. factor	4
AutoAlign	---

Contrast - Common

TR	900.0 ms
TE 1	13.40 ms
TE 2	32.96 ms
TE 3	52.52 ms
MTC	Off
Magn. Preparation	None
Flip Angle	30 deg
Fat-Water Contrast	Fat Saturation
Contrasts	3
Reconstruction	Magn./Phase

Contrast - Dynamic

Dynamic Mode	Standard
Measurements	305

Contrast - Dynamic

Delay in TR	0.00 ms

Resolution - Common

FOV Read	216 mm
FOV Phase	111.1 %
Slice Thickness	3.0 mm
Base Resolution	72
Phase Resolution	100 %
Interpolation	Off

Resolution - Acceleration

Acceleration Mode	GRAPPA
Reference scan mode	Single-shot
Acceleration Factor PE	2
Reference Lines PE	24
Phase Partial Fourier	7/8

Resolution - Filter

Raw Filter	Off
Elliptical Filter	Off
Hamming	Off
Distortion Correction	Off
Static Field Correction	Off
Normalize	Prescan

Geometry - Common

Slice Group	1
Slices	44
Distance Factor	0 %
Position	R1.1 P6.9 F25.5 mm
Orientation	Transversal
Phase Encoding Dir.	A >> P
Phase Oversampling	0 %
FOV Read	216 mm
FOV Phase	111.1 %
Slice Thickness	3.0 mm
TR	900.0 ms
Multi-Slice Mode	Interleaved
Series	Interleaved
Multi-band accel. factor	4

Geometry - AutoAlign

Slice Group	1
Position	R1.1 P6.9 F25.5 mm
Orientation	Transversal
Phase Encoding Dir.	A >> P
AutoAlign	---
Initial Position	R1.1 P6.9 F25.5
R	1.1 mm

Geometry - AutoAlign

P	6.9 mm
F	25.5 mm
Initial Orientation	Transversal
Initial Rotation	0.00 deg

Geometry - Saturation

Special Saturation	None

Geometry - Tim Planning Suite

Set-n-Go Protocol	Off
Table Position	0 mm
Table Position	H
Inline Composing	Off

System - Miscellaneous

Coil Selection	Auto Coil Select
Radial Sorting	Off
MSMA	S - C - T
Sagittal	R >> L
Coronal	A >> P
Transversal	F >> H
Coil Combination	Sum of Squares
Matrix Optimization	Off
Coil Focus	Flat

System - Adjustments

Adjustment Strategy	Standard
B0 Shim	Standard
B1 Shim	TrueForm
CoilShim	Off
Adjustment Tolerance	Auto
Adjust with Body Coil	Off
Confirm Frequency	Never
Assume Silicone	Off

System - Adjust Volume

Position	R1.1 P6.9 F25.5 mm
Orientation	Transversal
Rotation	90.00 deg
R >> L	216 mm
A >> P	240 mm
F >> H	132 mm
Reset	Off

System - pTx

B1 Shim	TrueForm
Excitation	Standard

System - Tx/Rx

Frequency 1H	123.256549 MHz
? Ref. Amplitude 1H	0.000 V
Reset	Off
Image Scaling	1.000

Physio - Signal

1st Signal/Mode	None
TR	900.0 ms
Multi-band accel. factor	4

BOLD

GLM Statistics	Off
Ignore Meas. at Start	0
Ignore After Transition	0
Model Transition States	Off
Temp. Highpass Filter	Off
Threshold	4.00
Paradigm Size	201
Meas[1]	Baseline
Meas[2]	Baseline
Meas[3]	Baseline
Meas[4]	Baseline
Meas[5]	Baseline
Meas[6]	Baseline
Meas[7]	Baseline
Meas[8]	Baseline
Meas[9]	Baseline
Meas[10]	Baseline
Meas[11]	Active
Meas[12]	Active
Meas[13]	Active
Meas[14]	Active
Meas[15]	Active
Meas[16]	Active
Meas[17]	Active
Meas[18]	Active
Meas[19]	Active
Meas[20]	Active
Meas[21]	Active
Meas[22]	Active
Meas[23]	Active
Meas[24]	Active
Meas[25]	Active
Meas[26]	Active
Meas[27]	Active
Meas[28]	Active
Meas[29]	Active
Meas[30]	Active
Meas[31]	Baseline
Meas[32]	Baseline
Meas[33]	Baseline
Meas[34]	Baseline
Meas[35]	Baseline
Meas[36]	Baseline
Meas[37]	Baseline
Meas[38]	Baseline
Meas[39]	Baseline
Meas[40]	Baseline
Meas[41]	Baseline
Meas[42]	Baseline

BOLD

Meas[43]	Baseline
Meas[44]	Baseline
Meas[45]	Baseline
Meas[46]	Baseline
Meas[47]	Baseline
Meas[48]	Baseline
Meas[49]	Baseline
Meas[50]	Baseline
Meas[51]	Active
Meas[52]	Active
Meas[53]	Active
Meas[54]	Active
Meas[55]	Active
Meas[56]	Active
Meas[57]	Active
Meas[58]	Active
Meas[59]	Active
Meas[60]	Active
Meas[61]	Active
Meas[62]	Active
Meas[63]	Active
Meas[64]	Active
Meas[65]	Active
Meas[66]	Active
Meas[67]	Active
Meas[68]	Active
Meas[69]	Active
Meas[70]	Active
Meas[71]	Baseline
Meas[72]	Baseline
Meas[73]	Baseline
Meas[74]	Baseline
Meas[75]	Baseline
Meas[76]	Baseline
Meas[77]	Baseline
Meas[78]	Baseline
Meas[79]	Baseline
Meas[80]	Baseline
Meas[81]	Baseline
Meas[82]	Baseline
Meas[83]	Baseline
Meas[84]	Baseline
Meas[85]	Baseline
Meas[86]	Baseline
Meas[87]	Baseline
Meas[88]	Baseline
Meas[89]	Baseline
Meas[90]	Baseline
Meas[91]	Active
Meas[92]	Active
Meas[93]	Active
Meas[94]	Active
Meas[95]	Active
Meas[96]	Active

BOLD

Meas[97]	Active
Meas[98]	Active
Meas[99]	Active
Meas[100]	Active
Meas[101]	Active
Meas[102]	Active
Meas[103]	Active
Meas[104]	Active
Meas[105]	Active
Meas[106]	Active
Meas[107]	Active
Meas[108]	Active
Meas[109]	Active
Meas[110]	Active
Meas[111]	Baseline
Meas[112]	Baseline
Meas[113]	Baseline
Meas[114]	Baseline
Meas[115]	Baseline
Meas[116]	Baseline
Meas[117]	Baseline
Meas[118]	Baseline
Meas[119]	Baseline
Meas[120]	Baseline
Meas[121]	Baseline
Meas[122]	Baseline
Meas[123]	Baseline
Meas[124]	Baseline
Meas[125]	Baseline
Meas[126]	Baseline
Meas[127]	Baseline
Meas[128]	Baseline
Meas[129]	Baseline
Meas[130]	Baseline
Meas[131]	Active
Meas[132]	Active
Meas[133]	Active
Meas[134]	Active
Meas[135]	Active
Meas[136]	Active
Meas[137]	Active
Meas[138]	Active
Meas[139]	Active
Meas[140]	Active
Meas[141]	Active
Meas[142]	Active
Meas[143]	Active
Meas[144]	Active
Meas[145]	Active
Meas[146]	Active
Meas[147]	Active
Meas[148]	Active
Meas[149]	Active
Meas[150]	Active

BOLD

Meas[151]	Baseline
Meas[152]	Baseline
Meas[153]	Baseline
Meas[154]	Baseline
Meas[155]	Baseline
Meas[156]	Baseline
Meas[157]	Baseline
Meas[158]	Baseline
Meas[159]	Baseline
Meas[160]	Baseline
Meas[161]	Baseline
Meas[162]	Baseline
Meas[163]	Baseline
Meas[164]	Baseline
Meas[165]	Baseline
Meas[166]	Baseline
Meas[167]	Baseline
Meas[168]	Baseline
Meas[169]	Baseline
Meas[170]	Baseline
Meas[171]	Active
Meas[172]	Active
Meas[173]	Active
Meas[174]	Active
Meas[175]	Active
Meas[176]	Active
Meas[177]	Active
Meas[178]	Active
Meas[179]	Active
Meas[180]	Active
Meas[181]	Active
Meas[182]	Active
Meas[183]	Active
Meas[184]	Active
Meas[185]	Active
Meas[186]	Active
Meas[187]	Active
Meas[188]	Active
Meas[189]	Active
Meas[190]	Active
Meas[191]	Baseline
Meas[192]	Baseline
Meas[193]	Baseline
Meas[194]	Baseline
Meas[195]	Baseline
Meas[196]	Baseline
Meas[197]	Baseline
Meas[198]	Baseline
Meas[199]	Baseline
Meas[200]	Baseline
Meas[201]	Baseline
Motion Correction	Off
Spatial Filter	Off
Measurements	305

BOLD

Delay in TR	0.00 ms

Inline - Open Recon

Algorithm	None

Sequence - Part 1

Sequence Name	epfid
Dimension	2D
Excitation	Standard
Gradient Mode	Performance
Flow Compensation	None
Bandwidth	2314 Hz/Px
Echo Spacing	0.54 ms
Free Echo Spacing	Off
EPI Factor	80

Sequence - Part 2

Introduction	Off
RF Spoiling	Off

Sequence - Special

Excite pulse duration	5120 us
Min. prep scans	0
Min. prep scans SB	0
Inter-TE delay	0 us
Delay before PC scans	0 us
Single-band images	On
MB LeakBlock kernel	On
MB dual kernel	Off
MB RF phase scramble	Off
Opt. MB RF pulse BW	Off
SENSE1 coil combine	On
Invert RO/PE polarity	Off
Force Maxwell corr.	Off
PF omits higher k-space	Off
Disable B1 control loop	Off
Disable freq. update	Off
Suppress 16-bit DICOM	Off
Force equal slice timing	Off
Online multi-band recon.	Online
FFT scale factor	1.00
Fat saturation FA	110.00 deg
Fat sat. offset	0.00 Hz
Sinc exc. pulse BWTP	5.20
Physio recording	DICOM
Triggering scheme	Standard

Sequence - Assistant

SAR Assistant	Off

\\SAHMRI_Research\Head\AEP\AEP MRI ACQUISITION PROTOCOL Cima_v21\FMap_AxObl_AP- *
TA: 9 sec Coil Selection: Auto Voxel Size: 2.4×2.4×2.0 mm³ Acc:: None Rel. SNR: 1.00

Properties

Start measurement without further preparation	On
Wait for User to Start	Off
Start measurements	Single Measurement
Prio Recon	Off
Auto Open Inline Display	Off
Auto Close Inline Display	Off
Load Images to MR View&GO	On
Auto Store Images	On
Disable auto transfer to PACS	Off
Load Images to Stamp Segments	Off
Load Images to Graphic Segments	Off
Graphic segment	Default
Inline Movie	Off

Routine

Slice Group	1
Slices	72
Distance Factor	0 %
Position	R1.1 P6.9 F25.5 mm
Orientation	Transversal
Phase Encoding Dir.	A >> P
Phase Oversampling	0 %
FOV Read	264 mm
FOV Phase	100.0 %
Slice Thickness	2.0 mm
TR	8500.0 ms
TE	70.00 ms
Averages	1
Multi-band accel. factor	1
AutoAlign	---

Contrast - Common

TR	8500.0 ms
TE	70.00 ms
MTC	Off
Magn. Preparation	None
Flip Angle	90 deg
Refocus flip angle	180 deg
Fat-Water Contrast	Fat Saturation
Grad. rev. fat suppr.	Disabled
Contrasts	1
Reconstruction	Magnitude

Contrast - Dynamic

Dynamic Mode	Standard
Measurements	1
Delay in TR	0.00 ms

Resolution - Common

FOV Read	264 mm
FOV Phase	100.0 %
Slice Thickness	2.0 mm
Base Resolution	112
Phase Resolution	100 %
Interpolation	Off

Resolution - Acceleration

Acceleration Mode	None
Phase Partial Fourier	7/8

Resolution - Filter

Raw Filter	Off
Elliptical Filter	Off
Hamming	Off
Distortion Correction	Off
Static Field Correction	Off
Normalize	Prescan

Geometry - Common

Slice Group	1
Slices	72
Distance Factor	0 %
Position	R1.1 P6.9 F25.5 mm
Orientation	Transversal
Phase Encoding Dir.	A >> P
Phase Oversampling	0 %
FOV Read	264 mm
FOV Phase	100.0 %
Slice Thickness	2.0 mm
TR	8500.0 ms
Multi-Slice Mode	Interleaved
Series	Interleaved
Multi-band accel. factor	1

Geometry - AutoAlign

Slice Group	1
Position	R1.1 P6.9 F25.5 mm
Orientation	Transversal
Phase Encoding Dir.	A >> P
AutoAlign	---
Initial Position	R1.1 P6.9 F25.5
R	1.1 mm
P	6.9 mm
F	25.5 mm
Initial Orientation	Transversal
Initial Rotation	0.00 deg

Geometry - Saturation

Special Saturation	None

Geometry - Tim Planning Suite

Set-n-Go Protocol	Off
Table Position	0 mm
Table Position	H
Inline Composing	Off

System - Miscellaneous

Coil Selection	Auto Coil Select
Radial Sorting	Off
MSMA	S - C - T
Sagittal	R >> L
Coronal	A >> P
Transversal	F >> H
Coil Combination	Sum of Squares
Matrix Optimization	Off
Coil Focus	Flat

System - Adjustments

Adjustment Strategy	Standard
B0 Shim	Standard
B1 Shim	TrueForm
CoilShim	Off
Adjustment Tolerance	Auto
Adjust with Body Coil	Off
Confirm Frequency	Never
Assume Silicone	Off

System - Adjust Volume

Position	R1.1 P6.9 F25.5 mm
Orientation	Transversal
Rotation	0.00 deg
A >> P	264 mm
R >> L	264 mm
F >> H	144 mm
Reset	Off

System - pTx

B1 Shim	TrueForm
Excitation	Standard

System - Tx/Rx

Frequency 1H	123.256549 MHz
? Ref. Amplitude 1H	0.000 V
Reset	Off
Image Scaling	1.000

Physio - Signal

1st Signal/Mode	None
TR	8500.0 ms
Multi-band accel. factor	1

BOLD

GLM Statistics	Off
Ignore Meas. at Start	0
Ignore After Transition	0

BOLD

Model Transition States	On
Temp. Highpass Filter	On
Threshold	4.00
Paradigm Size	4
Meas[1]	Baseline
Meas[2]	Baseline
Meas[3]	Baseline
Meas[4]	Active
Motion Correction	Off
Spatial Filter	Off
Measurements	1
Delay in TR	0.00 ms

Inline - Open Recon

Algorithm	None

Sequence - Part 1

Sequence Name	epse
Dimension	2D
Excitation	Standard
RF Pulse Type	Normal
Gradient Mode	Performance
Bandwidth	2626 Hz/Px
Echo Spacing	0.54 ms
Free Echo Spacing	Off
EPI Factor	112

Sequence - Part 2

Introduction	Off

Sequence - Special

Min. prep scans	0
Delay before PC scans	0 us
SENSE1 coil combine	On
Invert RO/PE polarity	Off
PF omits higher k-space	Off
Disable B1 control loop	Off
Disable freq. update	Off
Suppress 16-bit DICOM	Off
Force equal slice timing	Off
FFT scale factor	1.00
Fat saturation FA	110.00 deg
Fat sat. offset	0.00 Hz
Physio recording	DICOM
Triggering scheme	Standard

Sequence - Assistant

SAR Assistant	Off

**\\SAHMRI_Research\Head\AEP\AEP MRI ACQUISITION PROTOCOL Cima_v21\FMap_AxObl_AP_InvROPE-*

TA: 9 sec Coil Selection: Auto Voxel Size: 2.4×2.4×2.0 mm³ Acc:: None Rel. SNR: 1.00

Properties

Start measurement without further preparation	On
Wait for User to Start	Off
Start measurements	Single Measurement
Prio Recon	Off
Auto Open Inline Display	Off
Auto Close Inline Display	Off
Load Images to MR View&GO	On
Auto Store Images	On
Disable auto transfer to PACS	Off
Load Images to Stamp Segments	Off
Load Images to Graphic Segments	Off
Graphic segment	Default
Inline Movie	Off

Routine

Slice Group	1
Slices	72
Distance Factor	0 %
Position	R1.1 P6.9 F25.5 mm
Orientation	Transversal
Phase Encoding Dir.	A >> P
Phase Oversampling	0 %
FOV Read	264 mm
FOV Phase	100.0 %
Slice Thickness	2.0 mm
TR	8500.0 ms
TE	70.00 ms
Averages	1
Multi-band accel. factor	1
AutoAlign	---

Contrast - Common

TR	8500.0 ms
TE	70.00 ms
MTC	Off
Magn. Preparation	None
Flip Angle	90 deg
Refocus flip angle	180 deg
Fat-Water Contrast	Fat Saturation
Grad. rev. fat suppr.	Disabled
Contrasts	1
Reconstruction	Magnitude

Contrast - Dynamic

Dynamic Mode	Standard
Measurements	1
Delay in TR	0.00 ms

Resolution - Common

FOV Read	264 mm
FOV Phase	100.0 %
Slice Thickness	2.0 mm
Base Resolution	112
Phase Resolution	100 %
Interpolation	Off

Resolution - Acceleration

Acceleration Mode	None
Phase Partial Fourier	7/8

Resolution - Filter

Raw Filter	Off
Elliptical Filter	Off
Hamming	Off
Distortion Correction	Off
Static Field Correction	Off
Normalize	Prescan

Geometry - Common

Slice Group	1
Slices	72
Distance Factor	0 %
Position	R1.1 P6.9 F25.5 mm
Orientation	Transversal
Phase Encoding Dir.	A >> P
Phase Oversampling	0 %
FOV Read	264 mm
FOV Phase	100.0 %
Slice Thickness	2.0 mm
TR	8500.0 ms
Multi-Slice Mode	Interleaved
Series	Interleaved
Multi-band accel. factor	1

Geometry - AutoAlign

Slice Group	1
Position	R1.1 P6.9 F25.5 mm
Orientation	Transversal
Phase Encoding Dir.	A >> P
AutoAlign	---
Initial Position	R1.1 P6.9 F25.5
R	1.1 mm
P	6.9 mm
F	25.5 mm
Initial Orientation	Transversal
Initial Rotation	0.00 deg

Geometry - Saturation

Special Saturation	None

Geometry - Tim Planning Suite

Set-n-Go Protocol	Off
Table Position	0 mm
Table Position	H
Inline Composing	Off

System - Miscellaneous

Coil Selection	Auto Coil Select
Radial Sorting	Off
MSMA	S - C - T
Sagittal	R >> L
Coronal	A >> P
Transversal	F >> H
Coil Combination	Sum of Squares
Matrix Optimization	Off
Coil Focus	Flat

System - Adjustments

Adjustment Strategy	Standard
B0 Shim	Standard
B1 Shim	TrueForm
CoilShim	Off
Adjustment Tolerance	Auto
Adjust with Body Coil	Off
Confirm Frequency	Never
Assume Silicone	Off

System - Adjust Volume

Position	R1.1 P6.9 F25.5 mm
Orientation	Transversal
Rotation	0.00 deg
A >> P	264 mm
R >> L	264 mm
F >> H	144 mm
Reset	Off

System - pTx

B1 Shim	TrueForm
Excitation	Standard

System - Tx/Rx

Frequency 1H	123.256549 MHz
? Ref. Amplitude 1H	0.000 V
Reset	Off
Image Scaling	1.000

Physio - Signal

1st Signal/Mode	None
TR	8500.0 ms
Multi-band accel. factor	1

BOLD

GLM Statistics	Off
Ignore Meas. at Start	0
Ignore After Transition	0
Model Transition States	On
Temp. Highpass Filter	On
Threshold	4.00
Paradigm Size	4
Meas[1]	Baseline
Meas[2]	Baseline
Meas[3]	Baseline
Meas[4]	Active
Motion Correction	Off
Spatial Filter	Off
Measurements	1
Delay in TR	0.00 ms

Inline - Open Recon

Algorithm	None

Sequence - Part 1

Sequence Name	epse
Dimension	2D
Excitation	Standard
RF Pulse Type	Normal
Gradient Mode	Performance
Bandwidth	2626 Hz/Px
Echo Spacing	0.54 ms
Free Echo Spacing	Off
EPI Factor	112

Sequence - Part 2

Introduction	Off

Sequence - Special

Min. prep scans	0
Delay before PC scans	0 us
SENSE1 coil combine	On
Invert RO/PE polarity	On
PF omits higher k-space	Off
Disable B1 control loop	Off
Disable freq. update	Off
Suppress 16-bit DICOM	Off
Force equal slice timing	Off
FFT scale factor	1.00
Fat saturation FA	110.00 deg
Fat sat. offset	0.00 Hz
Physio recording	DICOM
Triggering scheme	Standard

Sequence - Assistant

SAR Assistant	Off

\\SAHMRI_Research\Head\AEP\AEP MRI ACQUISITION PROTOCOL Cima_v21\fMRI_AxObl_AP_Movie- *
TA: 13:54 min Coil Selection: Auto Voxel Size: 3.0×3.0×3.0 mm³ Acc:: 2 Rel. SNR: 1.00

Properties

Start measurement without further preparation	Off
Wait for User to Start	Off
Start measurements	Single Measurement
Prio Recon	Off
Auto Open Inline Display	Off
Auto Close Inline Display	Off
Load Images to MR View&GO	On
Auto Store Images	On
Disable auto transfer to PACS	Off
Load Images to Stamp Segments	Off
Load Images to Graphic Segments	Off
Graphic segment	Default
Inline Movie	Off

Routine

Slice Group	1
Slices	44
Distance Factor	0 %
Position	R1.1 P6.9 F25.5 mm
Orientation	Transversal
Phase Encoding Dir.	A >> P
Phase Oversampling	0 %
FOV Read	216 mm
FOV Phase	111.1 %
Slice Thickness	3.0 mm
TR	900.0 ms
TE 1	13.40 ms
TE 2	32.96 ms
TE 3	52.52 ms
Averages	1
Multi-band accel. factor	4
AutoAlign	---

Contrast - Common

TR	900.0 ms
TE 1	13.40 ms
TE 2	32.96 ms
TE 3	52.52 ms
MTC	Off
Magn. Preparation	None
Flip Angle	30 deg
Fat-Water Contrast	Fat Saturation
Contrasts	3
Reconstruction	Magn./Phase

Contrast - Dynamic

Dynamic Mode	Standard
Measurements	907
Delay in TR	0.00 ms

Resolution - Common

FOV Read	216 mm
FOV Phase	111.1 %
Slice Thickness	3.0 mm
Base Resolution	72
Phase Resolution	100 %
Interpolation	Off

Resolution - Acceleration

Acceleration Mode	GRAPPA
Reference scan mode	Single-shot
Acceleration Factor PE	2
Reference Lines PE	24
Phase Partial Fourier	7/8

Resolution - Filter

Raw Filter	Off
Elliptical Filter	Off
Hamming	Off
Distortion Correction	Off
Static Field Correction	Off
Normalize	Prescan

Geometry - Common

Slice Group	1
Slices	44
Distance Factor	0 %
Position	R1.1 P6.9 F25.5 mm
Orientation	Transversal
Phase Encoding Dir.	A >> P
Phase Oversampling	0 %
FOV Read	216 mm
FOV Phase	111.1 %
Slice Thickness	3.0 mm
TR	900.0 ms
Multi-Slice Mode	Interleaved
Series	Interleaved
Multi-band accel. factor	4

Geometry - AutoAlign

Slice Group	1
Position	R1.1 P6.9 F25.5 mm
Orientation	Transversal
Phase Encoding Dir.	A >> P
AutoAlign	---
Initial Position	R1.1 P6.9 F25.5
R	1.1 mm
P	6.9 mm
F	25.5 mm
Initial Orientation	Transversal
Initial Rotation	0.00 deg

Geometry - Saturation

Special Saturation	None

Geometry - Tim Planning Suite

Set-n-Go Protocol	Off
Table Position	0 mm
Table Position	H
Inline Composing	Off

System - Miscellaneous

Coil Selection	Auto Coil Select
Radial Sorting	Off
MSMA	S - C - T
Sagittal	R >> L
Coronal	A >> P
Transversal	F >> H
Coil Combination	Sum of Squares
Matrix Optimization	Off
Coil Focus	Flat

System - Adjustments

Adjustment Strategy	Standard
B0 Shim	Standard
B1 Shim	TrueForm
CoilShim	Off
Adjustment Tolerance	Auto
Adjust with Body Coil	Off
Confirm Frequency	Never
Assume Silicone	Off

System - Adjust Volume

Position	R1.1 P6.9 F25.5 mm
Orientation	Transversal
Rotation	90.00 deg
R >> L	216 mm
A >> P	240 mm
F >> H	132 mm
Reset	Off

System - pTx

B1 Shim	TrueForm
Excitation	Standard

System - Tx/Rx

Frequency 1H	123.256549 MHz
? Ref. Amplitude 1H	0.000 V
Reset	Off
Image Scaling	1.000

Physio - Signal

1st Signal/Mode	None
TR	900.0 ms
Multi-band accel. factor	4

BOLD

GLM Statistics	Off
Ignore Meas. at Start	0
Ignore After Transition	0
Model Transition States	Off
Temp. Highpass Filter	Off
Threshold	4.00
Paradigm Size	201
Meas[1]	Baseline
Meas[2]	Baseline
Meas[3]	Baseline
Meas[4]	Baseline
Meas[5]	Baseline
Meas[6]	Baseline
Meas[7]	Baseline
Meas[8]	Baseline
Meas[9]	Baseline
Meas[10]	Baseline
Meas[11]	Active
Meas[12]	Active
Meas[13]	Active
Meas[14]	Active
Meas[15]	Active
Meas[16]	Active
Meas[17]	Active
Meas[18]	Active
Meas[19]	Active
Meas[20]	Active
Meas[21]	Active
Meas[22]	Active
Meas[23]	Active
Meas[24]	Active
Meas[25]	Active
Meas[26]	Active
Meas[27]	Active
Meas[28]	Active
Meas[29]	Active
Meas[30]	Active
Meas[31]	Baseline
Meas[32]	Baseline
Meas[33]	Baseline
Meas[34]	Baseline
Meas[35]	Baseline
Meas[36]	Baseline
Meas[37]	Baseline
Meas[38]	Baseline
Meas[39]	Baseline
Meas[40]	Baseline
Meas[41]	Baseline
Meas[42]	Baseline
Meas[43]	Baseline
Meas[44]	Baseline
Meas[45]	Baseline
Meas[46]	Baseline
Meas[47]	Baseline

BOLD

Meas[48]	Baseline
Meas[49]	Baseline
Meas[50]	Baseline
Meas[51]	Active
Meas[52]	Active
Meas[53]	Active
Meas[54]	Active
Meas[55]	Active
Meas[56]	Active
Meas[57]	Active
Meas[58]	Active
Meas[59]	Active
Meas[60]	Active
Meas[61]	Active
Meas[62]	Active
Meas[63]	Active
Meas[64]	Active
Meas[65]	Active
Meas[66]	Active
Meas[67]	Active
Meas[68]	Active
Meas[69]	Active
Meas[70]	Active
Meas[71]	Baseline
Meas[72]	Baseline
Meas[73]	Baseline
Meas[74]	Baseline
Meas[75]	Baseline
Meas[76]	Baseline
Meas[77]	Baseline
Meas[78]	Baseline
Meas[79]	Baseline
Meas[80]	Baseline
Meas[81]	Baseline
Meas[82]	Baseline
Meas[83]	Baseline
Meas[84]	Baseline
Meas[85]	Baseline
Meas[86]	Baseline
Meas[87]	Baseline
Meas[88]	Baseline
Meas[89]	Baseline
Meas[90]	Baseline
Meas[91]	Active
Meas[92]	Active
Meas[93]	Active
Meas[94]	Active
Meas[95]	Active
Meas[96]	Active
Meas[97]	Active
Meas[98]	Active
Meas[99]	Active
Meas[100]	Active
Meas[101]	Active

BOLD

Meas[102]	Active
Meas[103]	Active
Meas[104]	Active
Meas[105]	Active
Meas[106]	Active
Meas[107]	Active
Meas[108]	Active
Meas[109]	Active
Meas[110]	Active
Meas[111]	Baseline
Meas[112]	Baseline
Meas[113]	Baseline
Meas[114]	Baseline
Meas[115]	Baseline
Meas[116]	Baseline
Meas[117]	Baseline
Meas[118]	Baseline
Meas[119]	Baseline
Meas[120]	Baseline
Meas[121]	Baseline
Meas[122]	Baseline
Meas[123]	Baseline
Meas[124]	Baseline
Meas[125]	Baseline
Meas[126]	Baseline
Meas[127]	Baseline
Meas[128]	Baseline
Meas[129]	Baseline
Meas[130]	Baseline
Meas[131]	Active
Meas[132]	Active
Meas[133]	Active
Meas[134]	Active
Meas[135]	Active
Meas[136]	Active
Meas[137]	Active
Meas[138]	Active
Meas[139]	Active
Meas[140]	Active
Meas[141]	Active
Meas[142]	Active
Meas[143]	Active
Meas[144]	Active
Meas[145]	Active
Meas[146]	Active
Meas[147]	Active
Meas[148]	Active
Meas[149]	Active
Meas[150]	Active
Meas[151]	Baseline
Meas[152]	Baseline
Meas[153]	Baseline
Meas[154]	Baseline
Meas[155]	Baseline

BOLD

Meas[156]	Baseline
Meas[157]	Baseline
Meas[158]	Baseline
Meas[159]	Baseline
Meas[160]	Baseline
Meas[161]	Baseline
Meas[162]	Baseline
Meas[163]	Baseline
Meas[164]	Baseline
Meas[165]	Baseline
Meas[166]	Baseline
Meas[167]	Baseline
Meas[168]	Baseline
Meas[169]	Baseline
Meas[170]	Baseline
Meas[171]	Active
Meas[172]	Active
Meas[173]	Active
Meas[174]	Active
Meas[175]	Active
Meas[176]	Active
Meas[177]	Active
Meas[178]	Active
Meas[179]	Active
Meas[180]	Active
Meas[181]	Active
Meas[182]	Active
Meas[183]	Active
Meas[184]	Active
Meas[185]	Active
Meas[186]	Active
Meas[187]	Active
Meas[188]	Active
Meas[189]	Active
Meas[190]	Active
Meas[191]	Baseline
Meas[192]	Baseline
Meas[193]	Baseline
Meas[194]	Baseline
Meas[195]	Baseline
Meas[196]	Baseline
Meas[197]	Baseline
Meas[198]	Baseline
Meas[199]	Baseline
Meas[200]	Baseline
Meas[201]	Baseline
Motion Correction	Off
Spatial Filter	Off
Measurements	907
Delay in TR	0.00 ms

Inline - Open Recon

Algorithm	None

Sequence - Part 1

Sequence Name	epfid
Dimension	2D
Excitation	Standard
Gradient Mode	Performance
Flow Compensation	None
Bandwidth	2314 Hz/Px
Echo Spacing	0.54 ms
Free Echo Spacing	Off
EPI Factor	80

Sequence - Part 2

Introduction	Off
RF Spoiling	Off

Sequence - Special

Excite pulse duration	5120 us
Min. prep scans	0
Min. prep scans SB	0
Inter-TE delay	0 us
Delay before PC scans	0 us
Single-band images	On
MB LeakBlock kernel	On
MB dual kernel	Off
MB RF phase scramble	Off
Opt. MB RF pulse BW	Off
SENSE1 coil combine	On
Invert RO/PE polarity	Off
Force Maxwell corr.	Off
PF omits higher k-space	Off
Disable B1 control loop	Off
Disable freq. update	Off
Suppress 16-bit DICOM	Off
Force equal slice timing	Off
Online multi-band recon.	Online
FFT scale factor	1.00
Fat saturation FA	110.00 deg
Fat sat. offset	0.00 Hz
Sinc exc. pulse BWTP	5.20
Physio recording	DICOM
Triggering scheme	Standard

Sequence - Assistant

SAR Assistant	Off

\\SAHMRI_Research\Head\AEP\AEP MRI ACQUISITION PROTOCOL Cima_v21\FMap_AxObl_AP- *
TA: 9 sec Coil Selection: Auto Voxel Size: 2.4×2.4×2.0 mm³ Acc:: None Rel. SNR: 1.00

Properties

Start measurement without further preparation	On
Wait for User to Start	Off
Start measurements	Single Measurement
Prio Recon	Off
Auto Open Inline Display	Off
Auto Close Inline Display	Off
Load Images to MR View&GO	On
Auto Store Images	On
Disable auto transfer to PACS	Off
Load Images to Stamp Segments	Off
Load Images to Graphic Segments	Off
Graphic segment	Default
Inline Movie	Off

Routine

Slice Group	1
Slices	72
Distance Factor	0 %
Position	R1.1 P6.9 F25.5 mm
Orientation	Transversal
Phase Encoding Dir.	A >> P
Phase Oversampling	0 %
FOV Read	264 mm
FOV Phase	100.0 %
Slice Thickness	2.0 mm
TR	8500.0 ms
TE	70.00 ms
Averages	1
Multi-band accel. factor	1
AutoAlign	---

Contrast - Common

TR	8500.0 ms
TE	70.00 ms
MTC	Off
Magn. Preparation	None
Flip Angle	90 deg
Refocus flip angle	180 deg
Fat-Water Contrast	Fat Saturation
Grad. rev. fat suppr.	Disabled
Contrasts	1
Reconstruction	Magnitude

Contrast - Dynamic

Dynamic Mode	Standard
Measurements	1
Delay in TR	0.00 ms

Resolution - Common

FOV Read	264 mm
FOV Phase	100.0 %
Slice Thickness	2.0 mm
Base Resolution	112
Phase Resolution	100 %
Interpolation	Off

Resolution - Acceleration

Acceleration Mode	None
Phase Partial Fourier	7/8

Resolution - Filter

Raw Filter	Off
Elliptical Filter	Off
Hamming	Off
Distortion Correction	Off
Static Field Correction	Off
Normalize	Prescan

Geometry - Common

Slice Group	1
Slices	72
Distance Factor	0 %
Position	R1.1 P6.9 F25.5 mm
Orientation	Transversal
Phase Encoding Dir.	A >> P
Phase Oversampling	0 %
FOV Read	264 mm
FOV Phase	100.0 %
Slice Thickness	2.0 mm
TR	8500.0 ms
Multi-Slice Mode	Interleaved
Series	Interleaved
Multi-band accel. factor	1

Geometry - AutoAlign

Slice Group	1
Position	R1.1 P6.9 F25.5 mm
Orientation	Transversal
Phase Encoding Dir.	A >> P
AutoAlign	---
Initial Position	R1.1 P6.9 F25.5
R	1.1 mm
P	6.9 mm
F	25.5 mm
Initial Orientation	Transversal
Initial Rotation	0.00 deg

Geometry - Saturation

Special Saturation	None

Geometry - Tim Planning Suite

Set-n-Go Protocol	Off
Table Position	0 mm
Table Position	H
Inline Composing	Off

System - Miscellaneous

Coil Selection	Auto Coil Select
Radial Sorting	Off
MSMA	S - C - T
Sagittal	R >> L
Coronal	A >> P
Transversal	F >> H
Coil Combination	Sum of Squares
Matrix Optimization	Off
Coil Focus	Flat

System - Adjustments

Adjustment Strategy	Standard
B0 Shim	Standard
B1 Shim	TrueForm
CoilShim	Off
Adjustment Tolerance	Auto
Adjust with Body Coil	Off
Confirm Frequency	Never
Assume Silicone	Off

System - Adjust Volume

Position	R1.1 P6.9 F25.5 mm
Orientation	Transversal
Rotation	0.00 deg
A >> P	264 mm
R >> L	264 mm
F >> H	144 mm
Reset	Off

System - pTx

B1 Shim	TrueForm
Excitation	Standard

System - Tx/Rx

Frequency 1H	123.256549 MHz
? Ref. Amplitude 1H	0.000 V
Reset	Off
Image Scaling	1.000

Physio - Signal

1st Signal/Mode	None
TR	8500.0 ms
Multi-band accel. factor	1

BOLD

GLM Statistics	Off
Ignore Meas. at Start	0
Ignore After Transition	0

BOLD

Model Transition States	On
Temp. Highpass Filter	On
Threshold	4.00
Paradigm Size	4
Meas[1]	Baseline
Meas[2]	Baseline
Meas[3]	Baseline
Meas[4]	Active
Motion Correction	Off
Spatial Filter	Off
Measurements	1
Delay in TR	0.00 ms

Inline - Open Recon

Algorithm	None

Sequence - Part 1

Sequence Name	epse
Dimension	2D
Excitation	Standard
RF Pulse Type	Normal
Gradient Mode	Performance
Bandwidth	2626 Hz/Px
Echo Spacing	0.54 ms
Free Echo Spacing	Off
EPI Factor	112

Sequence - Part 2

Introduction	Off

Sequence - Special

Min. prep scans	0
Delay before PC scans	0 us
SENSE1 coil combine	On
Invert RO/PE polarity	Off
PF omits higher k-space	Off
Disable B1 control loop	Off
Disable freq. update	Off
Suppress 16-bit DICOM	Off
Force equal slice timing	Off
FFT scale factor	1.00
Fat saturation FA	110.00 deg
Fat sat. offset	0.00 Hz
Physio recording	DICOM
Triggering scheme	Standard

Sequence - Assistant

SAR Assistant	Off

\\SAHMRI_Research\Head\AEP\AEP MRI ACQUISITION PROTOCOL Cima_v21\FMap_AxObl_AP_InvROPE-*

TA: 9 sec Coil Selection: Auto Voxel Size: 2.4×2.4×2.0 mm³ Acc:: None Rel. SNR: 1.00

Properties

Start measurement without further preparation	On
Wait for User to Start	Off
Start measurements	Single Measurement
Prio Recon	Off
Auto Open Inline Display	Off
Auto Close Inline Display	Off
Load Images to MR View&GO	On
Auto Store Images	On
Disable auto transfer to PACS	Off
Load Images to Stamp Segments	Off
Load Images to Graphic Segments	Off
Graphic segment	Default
Inline Movie	Off

Routine

Slice Group	1
Slices	72
Distance Factor	0 %
Position	R1.1 P6.9 F25.5 mm
Orientation	Transversal
Phase Encoding Dir.	A >> P
Phase Oversampling	0 %
FOV Read	264 mm
FOV Phase	100.0 %
Slice Thickness	2.0 mm
TR	8500.0 ms
TE	70.00 ms
Averages	1
Multi-band accel. factor	1
AutoAlign	---

Contrast - Common

TR	8500.0 ms
TE	70.00 ms
MTC	Off
Magn. Preparation	None
Flip Angle	90 deg
Refocus flip angle	180 deg
Fat-Water Contrast	Fat Saturation
Grad. rev. fat suppr.	Disabled
Contrasts	1
Reconstruction	Magnitude

Contrast - Dynamic

Dynamic Mode	Standard
Measurements	1
Delay in TR	0.00 ms

Resolution - Common

FOV Read	264 mm
FOV Phase	100.0 %
Slice Thickness	2.0 mm
Base Resolution	112
Phase Resolution	100 %
Interpolation	Off

Resolution - Acceleration

Acceleration Mode	None
Phase Partial Fourier	7/8

Resolution - Filter

Raw Filter	Off
Elliptical Filter	Off
Hamming	Off
Distortion Correction	Off
Static Field Correction	Off
Normalize	Prescan

Geometry - Common

Slice Group	1
Slices	72
Distance Factor	0 %
Position	R1.1 P6.9 F25.5 mm
Orientation	Transversal
Phase Encoding Dir.	A >> P
Phase Oversampling	0 %
FOV Read	264 mm
FOV Phase	100.0 %
Slice Thickness	2.0 mm
TR	8500.0 ms
Multi-Slice Mode	Interleaved
Series	Interleaved
Multi-band accel. factor	1

Geometry - AutoAlign

Slice Group	1
Position	R1.1 P6.9 F25.5 mm
Orientation	Transversal
Phase Encoding Dir.	A >> P
AutoAlign	---
Initial Position	R1.1 P6.9 F25.5
R	1.1 mm
P	6.9 mm
F	25.5 mm
Initial Orientation	Transversal
Initial Rotation	0.00 deg

Geometry - Saturation

Special Saturation	None

Geometry - Tim Planning Suite

Set-n-Go Protocol	Off
Table Position	0 mm
Table Position	H
Inline Composing	Off

System - Miscellaneous

Coil Selection	Auto Coil Select
Radial Sorting	Off
MSMA	S - C - T
Sagittal	R >> L
Coronal	A >> P
Transversal	F >> H
Coil Combination	Sum of Squares
Matrix Optimization	Off
Coil Focus	Flat

System - Adjustments

Adjustment Strategy	Standard
B0 Shim	Standard
B1 Shim	TrueForm
CoilShim	Off
Adjustment Tolerance	Auto
Adjust with Body Coil	Off
Confirm Frequency	Never
Assume Silicone	Off

System - Adjust Volume

Position	R1.1 P6.9 F25.5 mm
Orientation	Transversal
Rotation	0.00 deg
A >> P	264 mm
R >> L	264 mm
F >> H	144 mm
Reset	Off

System - pTx

B1 Shim	TrueForm
Excitation	Standard

System - Tx/Rx

Frequency 1H	123.256549 MHz
? Ref. Amplitude 1H	0.000 V
Reset	Off
Image Scaling	1.000

Physio - Signal

1st Signal/Mode	None
TR	8500.0 ms
Multi-band accel. factor	1

BOLD

GLM Statistics	Off
Ignore Meas. at Start	0
Ignore After Transition	0
Model Transition States	On
Temp. Highpass Filter	On
Threshold	4.00
Paradigm Size	4
Meas[1]	Baseline
Meas[2]	Baseline
Meas[3]	Baseline
Meas[4]	Active
Motion Correction	Off
Spatial Filter	Off
Measurements	1
Delay in TR	0.00 ms

Inline - Open Recon

Algorithm	None

Sequence - Part 1

Sequence Name	epse
Dimension	2D
Excitation	Standard
RF Pulse Type	Normal
Gradient Mode	Performance
Bandwidth	2626 Hz/Px
Echo Spacing	0.54 ms
Free Echo Spacing	Off
EPI Factor	112

Sequence - Part 2

Introduction	Off

Sequence - Special

Min. prep scans	0
Delay before PC scans	0 us
SENSE1 coil combine	On
Invert RO/PE polarity	On
PF omits higher k-space	Off
Disable B1 control loop	Off
Disable freq. update	Off
Suppress 16-bit DICOM	Off
Force equal slice timing	Off
FFT scale factor	1.00
Fat saturation FA	110.00 deg
Fat sat. offset	0.00 Hz
Physio recording	DICOM
Triggering scheme	Standard

Sequence - Assistant

SAR Assistant	Off
